# Development of a TSR-based method for understanding structural relationships of cofactors and local environments in photosystem I

**DOI:** 10.1186/s12859-025-06038-y

**Published:** 2025-01-14

**Authors:** Lujun Luo, Tarikul I. Milon, Elijah K. Tandoh, Walter J. Galdamez, Andrei Y. Chistoserdov, Jianping Yu, Jan Kern, Yingchun Wang, Wu Xu

**Affiliations:** 1 Department of Chemistry, University of Louisiana at Lafayette, Lafayette, LA 70504 USA; 2 Department of Biology, University of Louisiana at Lafayette, Lafayette, LA 70504 USA; 3 Biosciences Center, National Renewable Energy Laboratory, Golden, CO 80401 USA; 4 Bioenergetics Department, MBIB Division, Lawrence Berkeley National Laboratory, Berkeley, CA 94720 USA; 5 Institute of Genetics and Developmental Biology, Chinese Academy of Sciences, Beijing, 100101 China

**Keywords:** TSR-based method, Photosystem I, Representation of cofactor 3D structures, Cofactor and protein interaction, Cofactor binding site and A and B branches

## Abstract

**Background:**

All chemical forms of energy and oxygen on Earth are generated via photosynthesis where light energy is converted into redox energy by two photosystems (PS I and PS II). There is an increasing number of PS I 3D structures deposited in the Protein Data Bank (PDB). The Triangular Spatial Relationship (TSR)-based algorithm converts 3D structures into integers (TSR keys). A comprehensive study was conducted, by taking advantage of the PS I 3D structures and the TSR-based algorithm, to answer three questions: (i) Are electron cofactors including P700, A_-1_ and A_0_, which are chemically identical chlorophylls, structurally different? (ii) There are two electron transfer chains (A and B branches) in PS I. Are the cofactors on both branches structurally different? (iii) Are the amino acids in cofactor binding sites structurally different from those not in cofactor binding sites?

**Results:**

The key contributions and important findings include: (i) a novel TSR-based method for representing 3D structures of pigments as well as for quantifying pigment structures was developed; (ii) the results revealed that the redox cofactor, P700, are structurally conserved and different from other redox factors. Similar situations were also observed for both A_-1_ and A_0_; (iii) the results demonstrated structural differences between A and B branches for the redox cofactors P700, A_-1_, A_0_ and A_1_ as well as their cofactor binding sites; (iv) the tryptophan residues close to A_0_ and A_1_ are structurally conserved; (v) The TSR-based method outperforms the Root Mean Square Deviation (RMSD) and the Ultrafast Shape Recognition (USR) methods.

**Conclusions:**

The structural analyses of redox cofactors and their binding sites provide a foundation for understanding the unique chemical and physical properties of each redox cofactor in PS I, which are essential for modulating the rate and direction of energy and electron transfers.

**Supplementary Information:**

The online version contains supplementary material available at 10.1186/s12859-025-06038-y.

## Introduction

Life on planet Earth is sustained largely by oxygenic photosynthesis. Oxygenic photosynthesis is a process in which higher plants, eukaryotic algae, and cyanobacteria convert CO_2_ to chemical forms of energy, produce O_2_ using sunlight and through that, they power the entire biological world [1, 2]. Virtually all oxygen in the atmosphere is thought to be generated through the photosynthetic process [3, 4]. This process can be divided into two chains of coordinated reactions: light reactions and dark reactions. In light reactions, sunlight is harnessed to synthesize ATP and NADPH from splitting H_2_O into H^+^, *e*^−^ and O_2_. ATP and NADPH are utilized in the dark (i.e., light-independent) reactions to drive the synthesis of carbohydrates from CO_2_. During light reactions, the four membrane-protein complexes, photosystem II (PS II), cytochrome *b*_6_*f*, photosystem I (PS I) and ATP synthase, function in a coordinated way to initiate the photosynthetic process. PS I and PS II are involved in capturing sunlight and converting the absorbed energy into the energy of charge separation, i.e., act as natural “solar cells” that convert light into electrical current. The PS I complex of cyanobacteria contains twelve subunits (PsaA, B, C, D, E, F, I, J, K, L, M and X), chlorophylls (Chls) and carotenoid cofactors [5]. The electron transfer chain of PS I consists of six Chls, two phylloquinones and three [4Fe-4S] clusters. PsaA and PsaB are the core subunits that harbor the most antenna Chls, the primary electron donor P700 (a dimer of Chls), and a chain of electron acceptors A_−1_ (a Chl *a*), A_0_ (a Chl *a*), A_1_ (a phylloquinone) and F_X_ (a [4Fe-4S] cluster). The peripheral subunit PsaC binds the terminal electron acceptors F_A_ and F_B_, two [4Fe-4S] clusters. Each individual electron transfer cofactor is labeled with a respective structural and spectroscopic name since they are located on both PsaA and PsaB sides of a pseudo-C2 axis of symmetry. There are two electron transfer chains starting from P700 (P700_A_/P700_B_), through the A branch (A_−1A_, A_0A_ and A_1A_) or the B branch (A_−1B_, A_0B_ and A_1B_) and converging at F_X_. The antenna contains ~ 100 Chls [5].

Electron transfer is a fundamental process required for energy conversion in biological systems. Essential for electron transfer is the fine-tuning of the redox potentials of the electron acceptors and donors through interactions with the protein in which they are embedded [6] and the precise arrangement of cofactors with respect to each other. Therefore, it is critical to obtain a mechanistic understanding of interactions between cofactors, e.g., Chl and quinone, and between cofactors and their protein environments. The Triangular Spatial Relationship (TSR)-based method was developed for comparing molecular 3D structures [7] and probing drug and target interactions [8]. The input data for the TSR-based method are experimentally determined 3D structures from the Protein Data Bank (PDB) [9]. The first version of the TSR algorithm creates triangles with the C_α_ atoms of proteins as vertices. Triangles are constructed for every combination of three amino acids of a protein structure. A TSR key (an integer) is computed using geometric features such as length, angle, and vertex labels. Labels are determined by a rule-based assignment, which ensures consistent assignment of keys to identical TSRs across proteins, hence allowing a simpler but exact representation of protein structures [7]. Representation of 3D structures by TSR keys has its unique advantage of searching for similar substructures across structure datasets. In this study, we have developed a new version of the TSR-based method for understanding structural relationships of Chls and quinones as well as structural relationships of Chl and quinone binding sites. The examples of electron cofactors (Chl and phylloquinone) used in this study are from PS I.

The crystal structure of PS I complex from the cyanobacterium *Thermosynechccocus elongatus* (thereafter *T. elongatus,* recently renamed to *T. vestitus*) was solved at 2.5 Å resolution [5]. This structure has been known for a long time and has had therefore a significant positive impact on functional studies of PS I. Plant and other cyanobacterial PS I structures were solved at 4.4 Å resolution [10], 3.4 Å resolution [11], 3.3 Å resolution [12], 2.8 Å resolution [13–15] and 2.6 Å resolution [16]. Over the last six years, the structural knowledge greatly increased with a large number of published structures (2018 [17, 18], 2019 [19–24], 2020 [25–32], 2021 [33–44], 2022 [45–52], 2023 [53–60], 2024 [61, 62]) from cyanobacteria and algae, some of them obtained under different light conditions and in different oligomeric states (monomer, trimer and tetramer forms). This wealth of information allows the architecture of pigments, cofactors and proteins to be accurately modeled at the atomic level. This study, by taking advantage of the available PS I 3D structures and the TSR-based algorithm, aims to answer three questions: (i) Cofactors of P700, A_−1_ and A_0_ are Chl molecules. What are structural differences among P700, A_−1_ and A_0_? (ii) What are structural differences between A-branch (P700_A_, A_−1A_, A_0A_ and A_1A_) and B-branch (P700_B_, A_−1B_, A_0B_ and A_1B_) cofactors and their corresponding binding sites? (iii) Are the amino acids in cofactor binding sites structurally different from the amino acids not in cofactor binding sites?

This study is organized into four sections. First, we discuss structural relationships of PsaA and PsaB polypeptides. Second, we report a method for representing 3D structures of Chl and phylloquinone and discuss the structural relationships of the pigments using such method. Third, we present the structural relationships of cofactor binding sites. Finally, we evaluate the TSR algorithm by comparing it with popular structural comparison methods. The main contribution to the method development includes a new representation of Chl and phylloquinone 3D structures. Key findings include correlations of cofactor structures or structures of cofactor binding sites with their functions. In summary, this work introduces a new computational method with advantages in understanding the structural foundation for determining the redox potentials of electron donors and acceptors. Through this extensive study of cofactor conformations and cofactor local protein environments, we have discovered unique substructures exclusively belonging to a certain type of cofactors or a specific binding site for a cofactor.

## Experimental procedures

### Key generation

Key generation method using C_α_ atoms, MaxDist and Theta was reported before [7]. Three vertices of triangle *i* are labeled as ${l}_{i1}$, ${l}_{i2}$ and ${l}_{i3}$ that are determined using a rule-based formula. MaxDist is defined as the distance of the longest edge of a triangle. Theta is defined as the angle that is < 90° between the line from the midpoint of the edge of ${l}_{i1}$ and ${l}_{i2}$ to the opposite vertex ${l}_{i3}$ and half of the ${l}_{i1}$—${l}_{i2}$ edge. The Python code for C_α_ key generation is available in the supplementary document.

### Protein structural similarity and distance calculation

The Generalized Jaccard coefficient measure [63] was used for calculating pairwise similarity between any two protein structures in a dataset [7]. The distance matrix is derived from the similarity matrix [7]. Protein structure clustering is visualized based on Average Linkage Clustering [64]. The complexity of the multiple dimensional relations among 3D structures is reduced and represented by the Multidimensional Scaling (MDS) method [65]. Structural images were prepared using the Visual Molecular Dynamics (VMD) package [66].

### Development of a new version of the TSR-based method for pigments

To quantify the structures of pigments including Chls and quinones, a new version of the TSR-based method has been developed where every possible triangle is constituted from all the atoms except hydrogen atoms in a pigment. The bin boundaries used for Theta were the same as those we reported for the TSR algorithm using C_α_ atoms [7]. Seventeen bins, about half the number of the MaxDist bins for the C_α_ TSR algorithm [8], with one angstrom as an interval were used for MaxDist. To generate TSR keys for pigments, information on PDB ID, chain and pigment name and ID is needed. Each cofactor of PS I complexes from different species was annotated by examining structures using VMD. Each type of atoms was assigned an integer. An atom filtering algorithm was developed to select specific atoms for TSR key generation.

### Development of a TSR algorithm for quantifying structures of amino acids

The TSR concept was used to develop an algorithm for quantifying the structures of different amino acids and same amino acids at the different positions. All atoms except hydrogen atoms of every amino acid were used for TSR key generation. The bin boundaries used for Theta were the same as those we reported for the TSR algorithm using C_α_ atoms [7]. Fifty-eight bins with one angstrom as an interval were used for MaxDist. Normalized Jaccard coefficient measure is used for calculation of similarity between two amino acids.

### Sequence alignment

The MUSCLE module of SnapGene was applied to conduct multiple sequence alignments. Phylogenetic studies of protein sequences were conducted using the MEGA software [67].

### Dataset preparation

The datasets containing 3D structures of PS I complexes from plants, cyanobacteria and algae cultured under white or red light, normal or high light, and normal or high temperature conditions were prepared. All pigments and proteins in the datasets were selected from the PDB [9]. The PDB IDs, chains, pigment names and IDs can be found in Supplementary File 1.

### Output files from key generation code and definition of different types of TSR keys

Two output files were generated from the key generation step for every molecule, either a protein, a cofactor or an amino acid. One output is named “key file” and data structure of a “key file” is an integer (TSR key) vector for representing a 3D structure of protein, cofactor or amino acid. The other output is referred as “triplet files” containing the details for three amino acids and their positions, MaxDist and Theta values and the key for each protein. If for cofactor or amino acids, a “triplet file” contains the details for three atoms, MaxDist and Theta values and the key. The keys using C_α_ atoms are called *CA* TSR keys. The keys for a pigment are called *Cofactor* TSR keys. The keys for amino acids are called *AA* TSR keys. For *CA, Cofactor and AA* TSR keys*,* they can be further divided into *distinct*, *total*, *distinct* and *total common*, and *distinct* and *total specific TSR keys* that were reported before [68] to reveal structural relationships. Calculations of every type of TSR key (*distinct*, *total*, *distinct common, total common*, *distinct specific* or *total specific*) is accomplished through integer search using “key files”. A TSR key is an integer and only the integer is not biologically and chemically meaningful. If a key of interest is identified through key search, the key needs to be mapped into the triangle(s) with the details of three C_α_ atoms and MaxDist and Theta values for *CA* TSR or with the details of three atoms and MaxDist and Theta values for *Cofactor* or *AA* TSR through searching the key in the “triplet files”.

### Ultrafast shape recognition method

The Ultrafast Shape Recognition (USR) method was developed by Ballester’s group [69]. In this method, the set of all atomic distances from four molecular locations are considered: the molecular centroid (ctd), the closest atom to ctd (cst), the farthest atom to ctd (fct), and the farthest atom to fct (ftf). Each set of four distances can be regarded as a distribution. The first three moments are used for each distribution. Therefore, USR encodes the shape of a molecule and creates pairwise similarity output using 12 descriptors. The pairwise similarity output is the input file for hierarchical cluster analyses. The Python codes to compare structures of proteins, cofactors and amino acids using the USR method are available in the supplementary document.

### Root mean square deviation method

The Root Mean Square Deviation (RMSD) method calculates the minimum value of the root-mean-square distance between all possible one-to-one matchings between the atoms in the superimposed structures [70]. Pairwise structural differences for protein C_α_ atoms were calculated using the TM-align method [71]. The Python codes to compare structures of cofactors and amino acids using the RMSD method are available in the supplementary document. The pairwise distance output files from the TM-align method are the input files for hierarchical cluster analyses.

### Statistical analyses

*T*-test was used to identify statistical differences between the different feature engineering methods’ similarity values. A threshold of *p* < 0.05 was used to determine significance.

## Results

### 3.1 The analysis of PsaA and PsaB structures using CA TSR keys has identified the specific substructures exclusively belonging to a certain organism or a particular cell-culture condition

#### The analysis of the entire PsaA and PsaB structures reveals high structural similarities of PsaA, PsaB and between PsaA and PsaB from different photosynthetic organisms

**Fig. 1:**
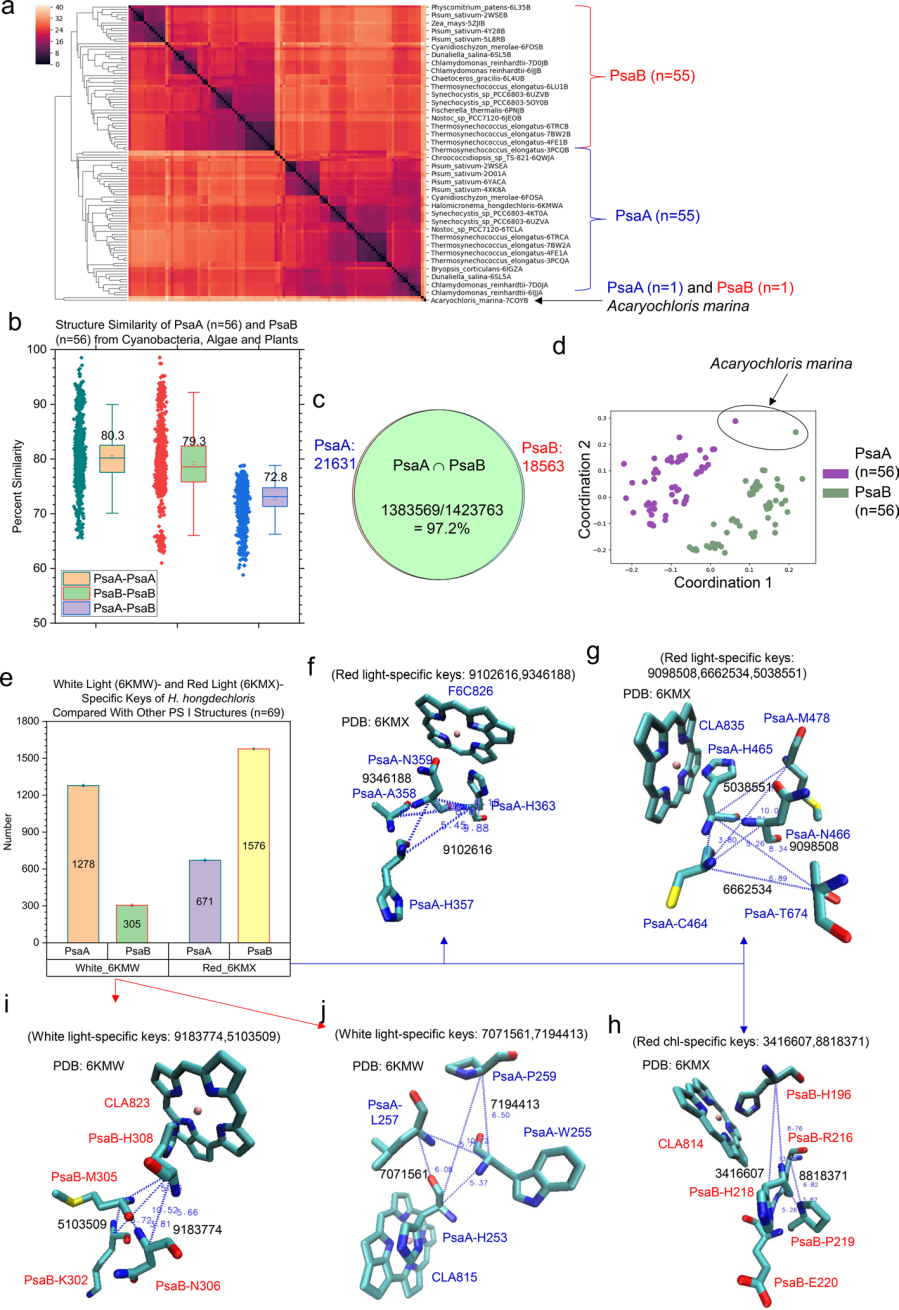
Hierarchical cluster and MDS analyses of PsaA and PsaB from diverse organisms demonstrate the capacity of the TSR algorithm for distinguishing their 3D structural differences and the substructures of PsaA or PsaB exclusively belonging to either red or white light cultural conditions were identified. Panel **a**, the hierarchical cluster analysis shows 3D structural relationships of PsaA and PsaB. The numbers of PsaA and PsaB sequences used in the analysis are labeled; panel **b**, calculations of the overall structural similarity of PsaA and PsaB and structural similarities for individual PsaA and PsaB structures. The average similarity values, SDs, and 25/75 percentiles are indicated; panel **c**, the Venn diagram of the numbers of TSR keys exclusively belonging to the PsaA protein class or the PsaB protein class and commonly shared between the PsaA and PsaB protein classes; panel **d**, the same PsaA and PsaB structures, presented in panel (**a**)*,* analyzed using the MDS method. The numbers of PsaA and PsaB structures and the PsaA and PsaB structures for the organism of *Acaryochloris marina* are labeled; panel **e**, the *specific* TSR keys identified for PsaA and PsaB of *H. hongdechloris* cultured under both red and white light culture conditions. Average values are labeled; panel **f**, the 3D substructures corresponding to the two *specific* TSR keys (9102616 and 9346188) that exclusively belong to PsaA of *H. hongdechloris* cultured under the red-light conditions. The triangle corresponding to the key 9102616 is constructed from the three C_α_ atoms from PsaA-H357, PsaA-N359 and PsaA-H363. The triangle corresponding to the key 9346188 is constructed from the three C_α_ atoms from PsaA-A358, PsaA-N359 and PsaA-H363; panel **g**, the 3D substructures corresponding to the three *specific* TSR keys (5038551, 6662534 and 9098508) that exclusively belong to PsaA of *H. hongdechloris* cultured under the red-light conditions. The triangle corresponding to the key 5038551 is constructed from the three C_α_ atoms from PsaA-C464, PsaA-H465 and PsaA-M478. The triangle corresponding to the key 6662534 is constructed from the three C_α_ atoms from PsaA-C464, PsaA-H465 and PsaA-T674. The triangle corresponding to the key 9098508 is constructed from the three C_α_ atoms from PsaA-C464, PsaA-H465 and PsaA-N466; panel **h**, the 3D substructures corresponding to the two *specific* TSR keys (3416607 and 8818371) which exclusively belong to PsaB of *H. hongdechloris* cultured under the red-light conditions. The triangle corresponding to the key 3416607 is constructed from the three C_α_ atoms from PsaB-H196, PsaB-H218 and PsaB-E220. The triangle corresponding to the key 8818371 is constructed from the three C_α_ atoms from PsaB-R216, PsaB-H218 and PsaB-P219; panel **i**, the 3D substructures corresponding to the two *specific* TSR keys (5103509 and 9183774) that exclusively belong to PsaB of *H. hongdechloris* cultured under the white light conditions. The triangle corresponding to the key 5103509 is constructed from the three C_α_ atoms from PsaB-K302, PsaB-M305 and PsaB-H308. The triangle corresponding to the key 9183774 is constructed from the three C_α_ atoms from PsaB-M305, PsaB-N306 and PsaB-H308; panel **j**, the 3D substructures corresponding to the two *specific* TSR keys (7071561 and 7194413) that exclusively belong to PsaA of *H. hongdechloris* cultured under the white-light conditions. The triangle corresponding to the key 7071561 is constructed from the three C_α_ atoms from PsaA-H253, PsaA-W255 and PsaA-L257. The triangle corresponding to the key 7194413 is constructed from the three C_α_ atoms from PsaA-H253, PsaA-W255 and PsaA-P259; For panels **f**–**j**, the IDs of the PDB and chlorophylls are labeled

The hierarchical cluster analysis demonstrates that the TSR-based method can distinguish PsaA structures from PsaB structures and vice versa. One exception was observed where PsaA and PsaB from *Acaryochloris marina* form a separated small cluster besides a large PsaA cluster and a large PsaB cluster (Fig. 1a). As expected, both PsaA (an average of 80.3% among different structures) and PsaB (an average of 79.3% among different structures) have high structural similarities as well as those between PsaA and PsaB (an average of 72.8%) (Fig. 1b). Such high structural similarities are supported by a high percentage of distinct (78.1% = 9.76 × 10^5^/1.25 × 10^6^ for PsaA, 77.9% = 9.66 × 10^5^/1.24 × 10^6^ for PsaB, 72.3% = 8.97 × 10^5^/1.24 × 10^6^ for PsaA and PsaB,) and total (98.5% = 6.59 × 10^7^/6.69 × 10^7^ for PsaA, 97.6% = 6.43 × 10^7^/6.59 × 10^7^ for PsaB, 97.6% = 6.44 × 10^7^/6.60 × 10^7^ for PsaA and PsaB) *common* keys (Supplementary Fig. 1). The Venn diagram provides additional evidence of a high structural similarity between PsaA and PsaB (Fig. 1c). The result from the MDS analysis of PsaA and PsaB structures supports that from the hierarchical cluster analysis (Fig. 1d). Distinct and total *specific* keys were identified for PsaA and PsaB (Supplementary Fig. 2). Those keys represent unique substructures exclusively belonging to PsaA or PsaB.

#### The structural analyses identified substructures exclusively belonging to a certain oligomer form of PS I, a certain culture condition and a certain type of pigment containing organisms

In cyanobacteria, PS I exists as a trimer or monomer, and possibly a tetramer [72]. Depending on environmental conditions, trimer may be shifted to monomer and vice versa, suggesting that each form functions slightly differently, which may also translate into structural changes [73]. Specific substructures represented by *specific* TSR keys exclusively belonging to a trimer (PDB: 5OY0) [17] or a monomer (PDB: 6HQB) [73] (Supplementary Fig. 3a) were identified. One example of three trimer-*specific* keys is shown in Supplementary Fig. 3b. Those trimer-*specific* keys-associated triangles are close to three Chl molecules (CLA1218, CLA1219 and CLA1220) (Supplementary Fig. 3b) that could be a part of red pigments. Two examples of monomer-*specific* keys are shown in Supplementary Fig. 3c and 3d. One example containing two monomer-*specific* keys and their associated triangles are close to two Chl molecules (CLA1108 and CLA1109) (Supplementary Fig. 3c). The other monomer-specific triangles are not close to any Chl molecules (Supplementary Fig. 3d). As expected, the PsaA polypeptides from trimer and monomer have identical amino acid sequences (Supplementary Fig. 4). This is the case for PsaB as well (Supplementary Fig. 4). The *specific* keys identified for trimer or monomer demonstrate that the TSR keys can be used to quantify conformational changes induced by oligomerization or de-oligomerization. Because the conformational changes are close to Chls, it may explain absorption differences between trimers and monomers of PS I.

The data for cyanobacterium *Halomicronema hongdechloris* indicated that its Chl *f* functions to harvest the far-red light. This resulted in changes of the PS I gene expression favoring PsaA and PsaB for binding of Chl *f* [31]. The sequence alignment analysis of PsaA and PsaB from *Halomicronema hongdechloris* (6KMW: white light and 6KMX: far-red light) shows the difference in amino acid sequences under different light conditions (Supplementary Fig. 5). To understand the structural changes induced or partially induced by white or far-red light, we further investigated the structures of PsaA and PsaB from this cyanobacterium grown under these light conditions*.* The *specific* TSR keys exclusively for white light or far-red light conditions were identified for both PsaA and PsaB (Fig. 1e). The details of seven far-red-light-*specific* keys and four white-light-*specific* keys were analyzed. Two far-red-*specific* keys are close to a Chl *f* (F6C826) (Fig. 1f), three *specific* keys are close to a Chl *a* (CLA835) (Fig. 1g) and two *specific* keys are close to a Chl *a* (CLA814) (Fig. 1h). Two similar examples were identified for white-light-*specific* keys. One example shows two keys that are close to CLA823 (Fig. 1i) and another example shows that two different keys are close to CLA815 (Fig. 1j). Cyanobacterium *Acaryochloris marina* also has the ability in absorbing far-red light. The special pair in this cyanobacterium is a dimer of Chl *d* and its epimer Chl *d*’ [34] rather than a dimer of Chl *a* and its epimer Chl *a*’ found in other species. Also, the primary electron acceptor is pheophytin *a* [34] instead of Chl *a*. Like the situations for trimer vs. monomer and white light vs. far-red light, we were able to identify the substructures exclusively belonging to only Chl *a*-containing, Chl *f*-containing and Chl *d*-containing organisms (Supplementary Fig. 6). The specific TSR keys discussed in this section reveal cofactor-specific protein environments that may contribute to absorption of a specific wavelength of light.

### Development of the TSR algorithm for representing 3D structures of electron transfer cofactors and the hierarchical cluster analysis revealed that the clusters of the electron donor or acceptor generally match with their functions

#### Development of the TSR algorithm for representing 3D structures of electron transfer cofactors

There are ~ 100 Chl molecules and 2 phylloquinone molecules based on a high-resolution crystal structure of cyanobacterial PS I [74]. Six out of these Chl molecules function as either an electron donor or acceptor and they form the reaction center. Two out of 96 Chl molecules may function as the linkers to connect the reaction center with the rest of the antenna Chl molecules. These two Chl molecules are called connecting Chls (A_C_). One is on PsaA side named A_CA_ and the other is on PsaB side named A_CB_ in this study. Similarly for the electron donors and acceptors, P700, A_−1_, A_0_ and A_1_ on the PsaA side are named P700_A_, A_0A_ and A_1A_ whereas they are named P700_B_, A_0B_ and A_1B_ if they are on the PsaB side. A_−1A_ and A_−1B_ are the only electron transfer cofactors, in which PsaA binds A_−1B_ and PsaB binds A_−1A_. P700_A_, A_−1A_, A_0A_ and A_1A_ are called A-branch electron transfer cofactors whereas P700_B_, A_−1B_, A_0B_ and A_1B_ are called B-branch electron transfer cofactors. To understand whether the electron donors, electron acceptors and connecting Chls have their specific structural characteristics, a novel method to represent their 3D structures was developed.

**Fig. 2:**
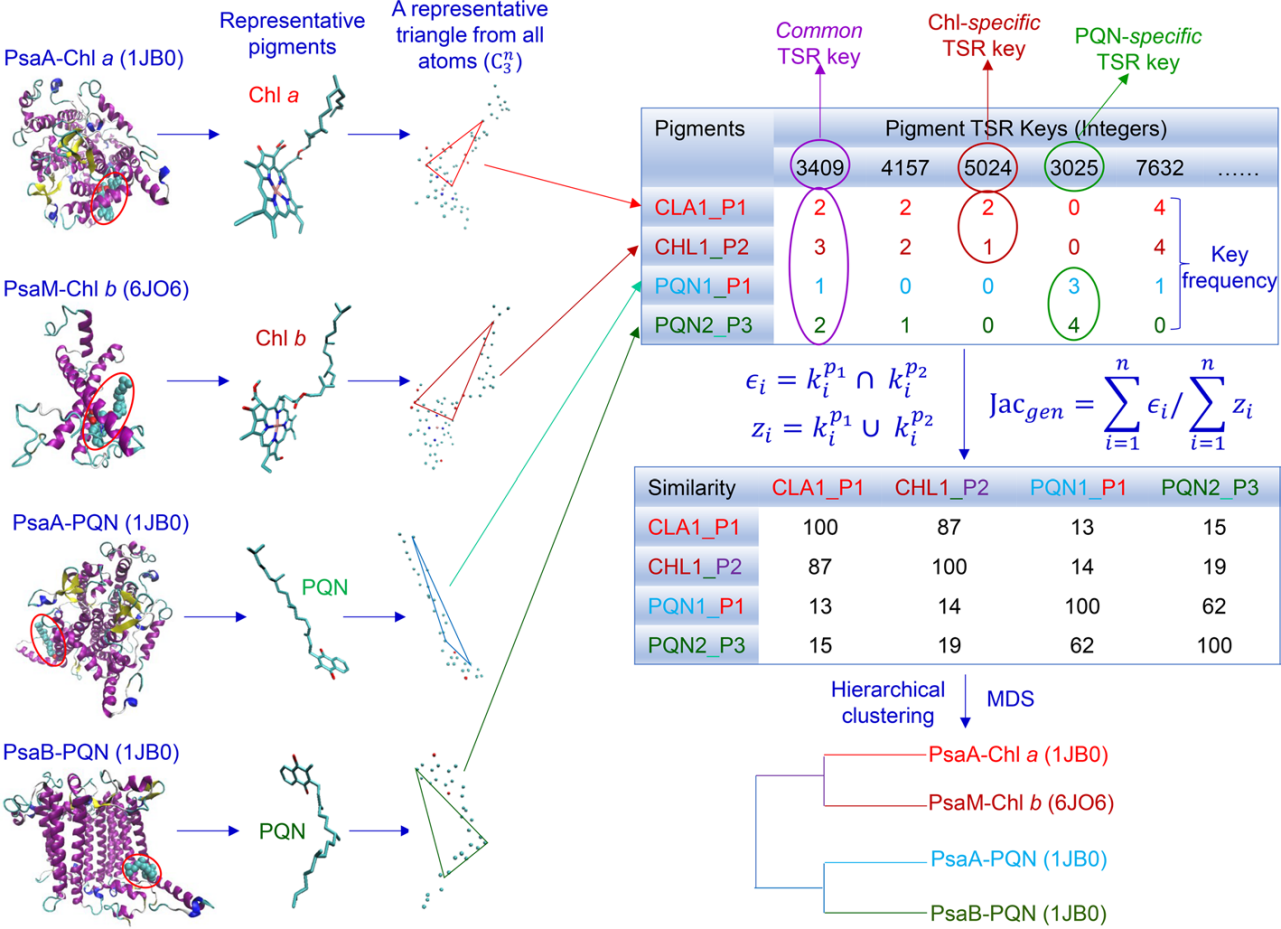
The design of the TSR algorithm for representing 3D structures of chlorophyll and phylloquinone molecules. It illustrates the schema of how to decode chlorophyll and phylloquinone 3D structures to integers (TSR keys) and how to calculate pairwise structural similarities using the calculated TSR keys and the Generalized Jaccard coefficient approach. An example of *common* and *specific* keys is shown. A hypothetical hierarchical cluster result is also shown

In this method, first, all atoms except hydrogen atoms of a pigment 3D structure are selected and all possible triangles constructed by the atoms are identified (Fig. 2). Second, three vertex labels are determined using the rule-based assignment. Third, TSR keys (integers) and key occurrence frequencies are calculated. Fourth, pairwise similarities between pigment 3D structures are calculated using the Generalized Jaccard similarity through computing identical and nonidentical keys, and their frequencies (Fig. 2). The 3D structures of pigments are represented by a vector of integers (*Cofactor* TSR keys). Such a representation for pigments is unique. The important objectives of this algorithm are to quantify structural similarities of pigments and provide insight into structural relationships through identifying *specific* and *common Cofactor* TSR keys (Fig. 2).

#### The clustering analysis has demonstrated that the electron donors and electron acceptors have unique structural characteristics

The selection of the structures of the electron donors and electron acceptors is based on the criteria: (i) whether it is a model structure of PS I; (ii) whether it comes from a model organism for photosynthesis research; (iii) whether PS I structures from a diversity of organisms are available and (iv) whether a high-resolution PS I structure is available. The crystal structure from *T. vestitus*, a type of thermophilic cyanobacteria, is the model PS I structure (PDB: 1JB0). *Chlamydomonas reinhardtii* (PDB: 6JO6)*,* a eukaryotic green alga, and *Synechocystis* sp. PCC 6803 (hereafter *Synechocystis*) (PDB: 5OY0), a strain of unicellular and freshwater cyanobacteria, are the model organisms for photosynthesis research and numerous functional studies were conducted in these two organisms. PDB 6PNJ contains a PS I structure from filamentous true-branching cyanobacterium *Fischerella thermalis* and PDB 5ZJI contains a PS I structure from a plant (*Zea mays*). Therefore, five PDBs (1JB0, 5OY0, 6JO6, 6PNJ and 5ZJI) were selected for the structural study of cofactors.

**Fig. 3:**
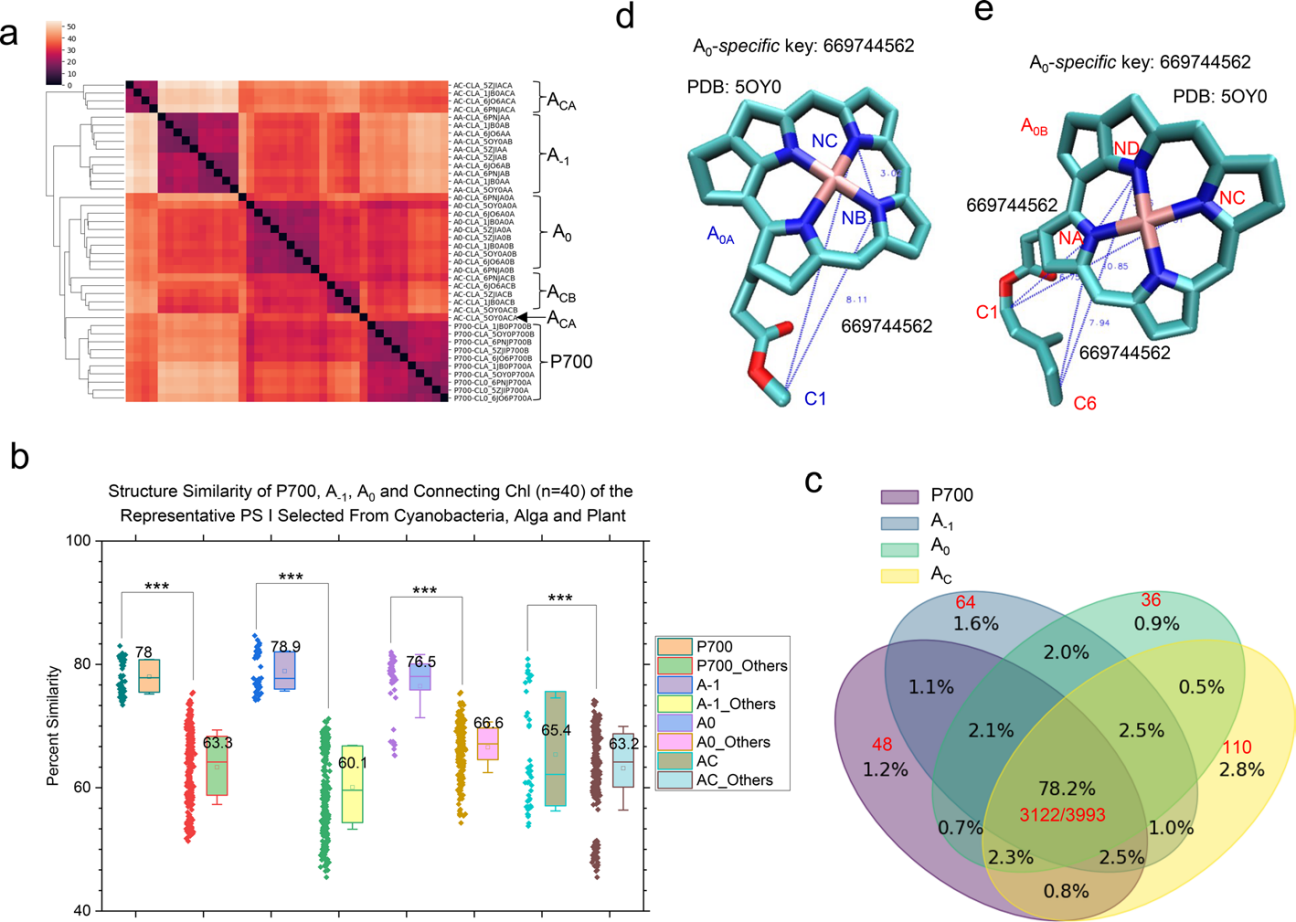
Hierarchical cluster analysis of electron cofactors P700, A_−1_, A_0_ and connecting chlorophyll molecules demonstrating the capacity of the TSR algorithm, which can distinguish their 3D structural differences. Panel **a**, the hierarchical cluster analysis of 3D structural relationships between P700, A_−1_, A_0_ and connecting chlorophyll molecules. A_CA_ (A_CB_) represents the connecting chlorophyll molecule on the PsaA (PsaB) side; panel **b**, the side-by-side structural comparisons between one type of cofactor pairs and between that type of cofactor and other types of cofactor pairs. The average similarity values, SDs, and 25/75 percentiles are indicated. *** means a *p* value is less than 0.001 using a *t*-test; panel **c**, the Venn diagram showing the numbers of TSR keys exclusively belonging to each type of cofactor class (the P700 cofactor class, A_−1_ cofactor class, A_0_ cofactor class and the connecting chlorophyll (A_C_) class), and the regions between any two, three and four cofactor classes; panels **d** and **e**, one A_0_ *specific* TSR key (669744562). The triangle corresponding to this key is constructed from three atoms (C1, NB and NC) of A_0A_ and is shown in panel (**d**), and the triangle corresponding to the key 669,744,562 constructed from three atoms (C1, NA and NC or C6, NA and ND) of A_0B_ is shown in panel (**e**). The PDB ID is labeled

The hierarchical cluster analysis shows that P700, A_−1_ and A_0_ form their own clusters, indicating they have their individual structural characteristics. More structural diversity was observed for A_C_. A_CA_ chlorophylls form their own cluster with an exception that one A_CA_ molecule is joined with the P700 cluster (Fig. 3a). A_CB_ structures also form their own cluster. However, the A_CB_ cluster is separated from the A_CA_ cluster (Fig. 3a). P700, A_−1_ and A_0_ have similar structural similarities, but they have higher structural similarities than A_C_ (Fig. 3b). P700s have higher structural similarities among themselves than those when P700s were compared with A_−1_, A_0_ and A_C_ (Fig. 3b). It is true also for A_−1_, A_0_ and A_C_ (Fig. 3b). All these (Fig. 3b) support the clustering result obtained for P700, A_−1_ and A_0_ (Fig. 3a). In addition, the analysis of structural similarity (Fig. 3b) demonstrates that A_C_ chlorophylls also have their structural characteristics. If we consider ten molecules of each type of cofactors as a group, P700, A_−1_, A_0_ and A_C_ groups share 78.2% of identical *Cofactor* TSR keys (Fig. 3c), suggesting that they have a high similarity percentage among four groups of cofactors as expected. If we consider individual cofactors, all forty P700, A_−1_, A_0_ and A_C_ pigments have 1350 distinct *common* keys (without considering key occurrence frequency) and 39,600 total *common* keys (with considering key occurrence frequency) on average (Supplementary Fig. 7). Those *common* keys can be found in each pigment of the 40 pigments. The 40 pigments have 2700 distinct and 43,400 total keys on average (Supplementary Fig. 7). P700, A_−1_, A_0_ and A_C_ share roughly 50% (1350/2700) to 91.2% (39,600/43,400) of common substructures. One *specific* key, 669,744,562, exclusively identified for A_0_, is found in all A_0_ molecules but not in P700, A_−1_ and A_C_. This A_0_-*specific* key is shown in Fig. 3d for A_0A_ with one occurrence frequency and in Fig. 3e for A_0B_ with two occurrence frequencies. To further examine the structural differences of the cofactors between the PsaA side and the PsaB side, we performed cluster analyses of each type of cofactors. The P700_A_ cluster and the P700_B_ cluster are distinct (Supplementary Fig. 8). In contrast, the A_−1A_ and A_−1B_ structures tend to cluster together (Supplementary Fig. 9). Two A_−1_ clusters were observed. However, each cluster is a mix of A_−1A_ and A_−1B_, suggesting A_−1_ structural diversity among different species. The situations for A_0A_ and A_0B_ (Supplementary Fig. 10) as well as for A_CA_ and A_CB_ (Supplementary Fig. 11) are found to lie between P700 and A_−1_.

**Fig. 4:**
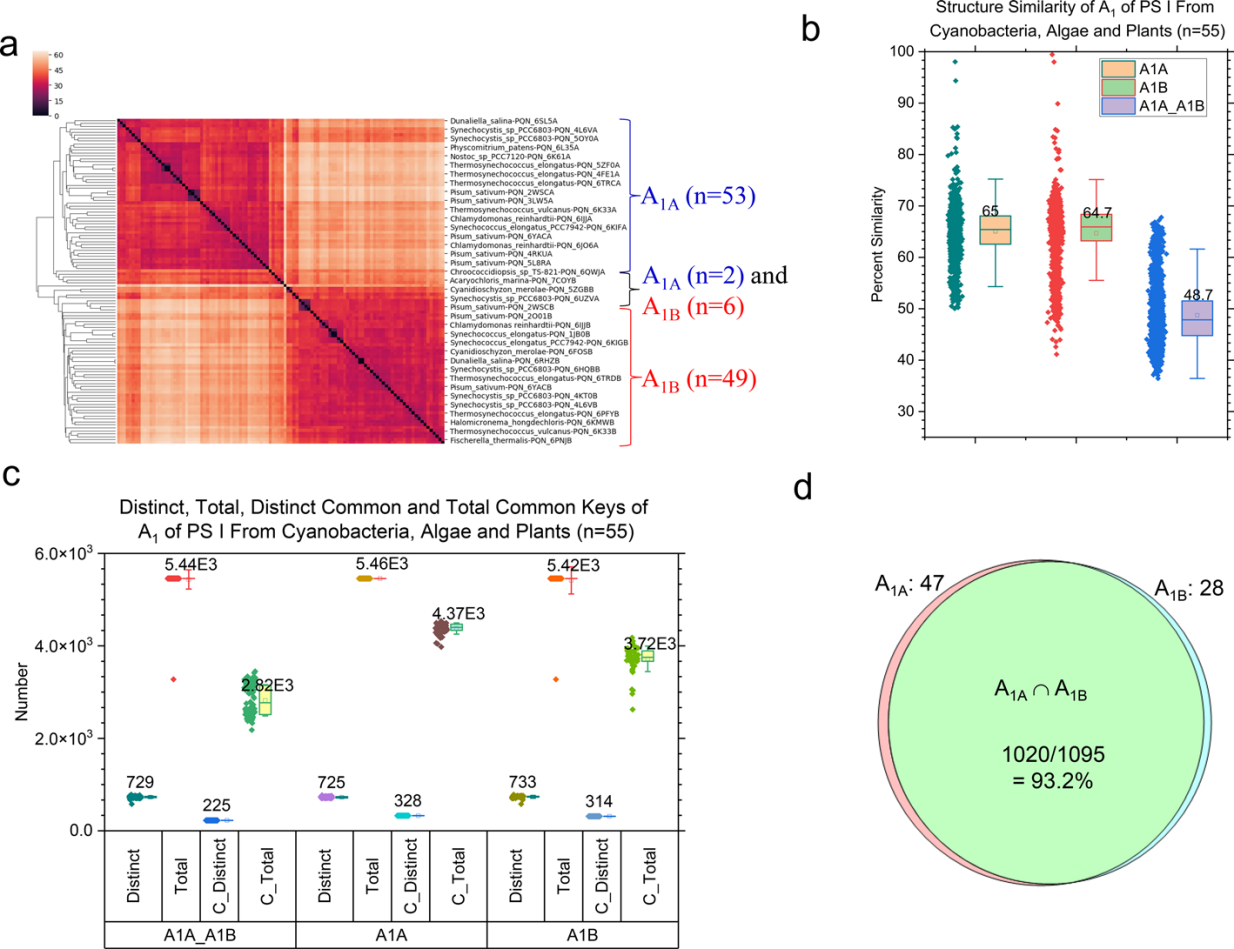
Hierarchical cluster analysis of electron cofactors A_1_ demonstrating the capacity of the TSR algorithm for distinguishing of their 3D structural differences. Panel **a**, the hierarchical cluster analysis of 3D structural relationships of A_1A_ and A_1B_. The numbers of A_1A_ and A_1B_ chlorophylls used in the analysis are labeled; panel **b**, calculated overall structural similarities of A_1A_ and A_1B_ and structural similarities for individual A_1A_ and A_1B_ structures. The average similarity values, SDs, and 25/75 percentiles are indicated; panel **c**, the combined distinct, total, distinct common and total common TSR keys for A_1A_, A_1B_ and A_1A_ and A_1B_. The average numbers, SDs, and 25/75 percentiles are indicated; panel **d**, the Venn diagram showing the numbers of TSR keys exclusively belonging to the A_1A_ cofactor class or the A_1B_ cofactor class and commonly shared between the A_1A_ and A_1B_ cofactor classes

We also performed the hierarchical cluster analysis of A_1_ molecules. Most A_1A_ structures group together. It is also the case for most A_1B_ structures. However, two A_1A_ and six A_1B_ structures cluster together (Fig. 4a). The average structural similarities of A_1A_ and A_1B_ are nearly the same. The average structural similarity between A_1A_ and A_1B_ are lower than that of A_1A_ as well as that of A_1B_ as expected (Fig. 4b). Taken together, the results suggest that A_1A_ and A_1B_ have their structural characteristics with their structural diversities. A_1A_ and A_1B_ share 30.9% (225/729) of distinct *common* keys and 51.8% (2820/5440) of total *common* keys (Fig. 4c). It indicates that A_1A_ and A_1B_ have 30.9% to 51.9% similar substructures. If we consider the keys from all A_1A_ molecules as a group and the keys from all A_1B_ molecules as another group, both groups share a high percentage of the same keys (Fig. 4d). Only very small portions of the keys were found exclusively belonging to either group (Fig. 4d).

### A role of local environments for understanding the mechanisms underlying cofactor–protein interactions

#### Distance calculations reveal the difference in the arrangement of overall Chl molecules between cyanobacterial and eukaryotic photosynthetic organisms and specific arrangement of electron transfer cofactors

**Fig. 5:**
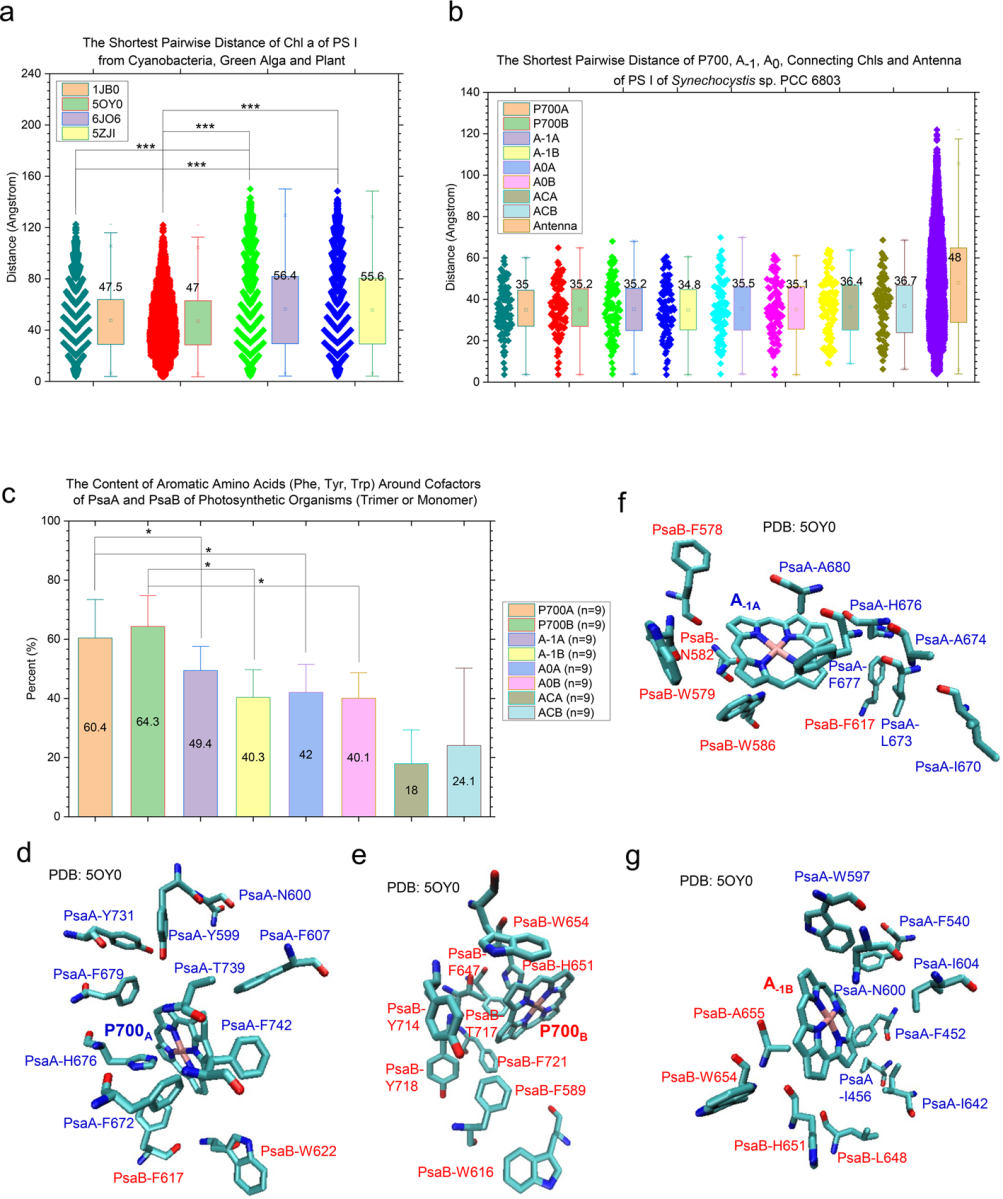
The difference in global arrangement of chlorophyll molecules between representative cyanobacteria and alga/plant, structural characteristic of the reaction centers and more aromatic residues closely interacting with P700. Panel **a**, he shortest distances between chlorophyll—chlorophyll pairs were calculated and are present. The PDB IDs are labeled. *** means a *p* value is less than 0.001 in a *t*-test; panel (b), the shortest distances between P700_A_—other chlorophyll pairs, P700_B_—other chlorophyll pairs, A_−1A_—other chlorophyll pairs, A_−1B_—other chlorophyll pairs, A_0A_—other chlorophyll pairs, A_0B_—other chlorophyll pairs, A_CA_—other chlorophyll pairs, A_CB_—other chlorophyll pairs and chlorophyll pairs (including antenna). In panels **a** and **b**, the average values, SDs, and 25/75 percentiles are indicated; panel **c**, the numbers of aromatic residues (phenylalanine, tyrosine and tryptophan) that have close interactions with P700_A_, P700_B_, A_−1A_, A_−1B_, A_0A_, A_0B_, A_CA_ and A_CB_. The percentages of those aromatic residues are present. The cutoff value for the close interactions is 3.5 Å. The average values and SDs are indicated. * means a *p* value is less than 0.05 using a *t*-test; Panels **d** through **g**, the representative surrounding amino acids of P700_A_ **d**, P700_B_ (**e**), A_−1A_ (**f**) and A_−1B_ **(g**) are illustrated. The PDB is 5OY0. The numbers of aromatic residues that have closely interactions with P700 is larger than those that closely interact with A_−1_ or A_0_

Both the chromophore—chromophore interaction strength and the chromophore—environment interaction coupling are important for modulating energy and electron transfers in PS I. Therefore, the shortest distances between each Chl pair were calculated and described herein. The description of the chromophore environment is discussed in the next section. These calculations show that eukaryotic photosynthetic organisms (green alga and plant) have larger pairwise distances between Chl pairs than those of cyanobacteria (Fig. 5a), suggesting differences in the antenna arrangement between cyanobacterial and higher plant systems which agree with the data described earlier in the literature [75]. The reaction centers including P700, A_−1_ and A_0_ of oxygenic organisms exhibit a common general architecture and share the same basic functional principles. Two connecting Chl molecules (A_CA_ and A_CB_) are special as they structurally and perhaps functionally connect A_−1_ and A_0_ of the electron transfer chains to the antenna. Therefore, we include the connecting Chl molecules in the analysis. The result reveals that P700, A_−1_, A_0_ and A_C_ have similar pairwise distances with the antenna Chls (Fig. 5b), suggesting unique positions of the redox factors in the cofactor—protein complex. It also suggests the special locations of two A_C_ molecules for connecting antenna Chls to the reaction center. In summary, the global arrangement of Chls is different between prokaryotic PS I and eukaryotic PS I and the locations of two A_C_ molecules are special that may indicate their specific functions.

#### Calculations of number and type of amino acids surrounding the cofactors reveal differences in local environments among different cofactors and between the A branch and the B branch

PS I are characterized by optimized structures where the protein scaffold acts on the energy and electron transfer cofactors, finely tuning their surroundings and modulating their properties and functionalities [76]. Numerous studies have addressed the contributions of individual amino acids to modulating the spectroscopic properties of bound redox cofactors. The subtle structural differences in cofactor binding sites have not been reported. First, the number of surrounding residues for the cofactors was investigated. Diverse cyanobacteria have similar average numbers of surrounding amino acids for Chl molecules while eukaryotic organisms have slightly lower numbers of surrounding amino acids (Supplementary Fig. 12). Significant numbers of Chl *a* and Chl *d* molecules were identified. Chl *d* molecules have slightly more surrounding amino acids than Chl *a* molecules (Supplementary Fig. 13). Second, the differences in cofactor binding sites between the A branch and the B branch were examined. The result shows that A_−1A_ has more surrounding amino acids than A_−1B_ (Supplementary Fig. 14). It is also true for the A_CA_ and A_CB_ binding sites (Supplementary Fig. 14). The redox cofactors (P700, A_−1_ and A_0_) have more amino acids than A_C_ and the rest of Chls (Supplementary Fig. 14). For the P700 and A_0_ binding sites, there is no difference between two branches (Supplementary Fig. 14). We found that the P700 binding sites have more aromatic residues than those of A_−1_ and A_0_ (Fig. 5c). In contrast, A_CA_ and A_CB_ have less numbers of aromatic residues than the cofactors in the reaction center (Fig. 5c). Figure 5d, e, f and g, illustrate the representative examples of the binding sites of P700_A_, P700_B_, A_−1A_ and A_−1B_, respectively. For the A_1_ binding sites, the B branch has more amino acids than the A branch (Supplementary Fig. 15). As the references, we calculated the amino acid compositions including aromatic residues for PsaA and PsaB (Supplementary Figs. 16 and 17). The top three most abundant amino acids for PsaA (Supplementary Fig. 16) and for PsaB (Supplementary Fig. 17) are Leu, Gly and Ala. Interestingly, we observed more surrounding aromatic residues for P700 than A_−1_ and A_0_ and a difference in the number of surrounding amino acids between the A branch and the B branch for A_−1_, A_C_ and A_1_.

#### The structural analysis using CA TSR keys demonstrates the differences of the redox cofactors between the A branch and the B branch

**Fig. 6:**
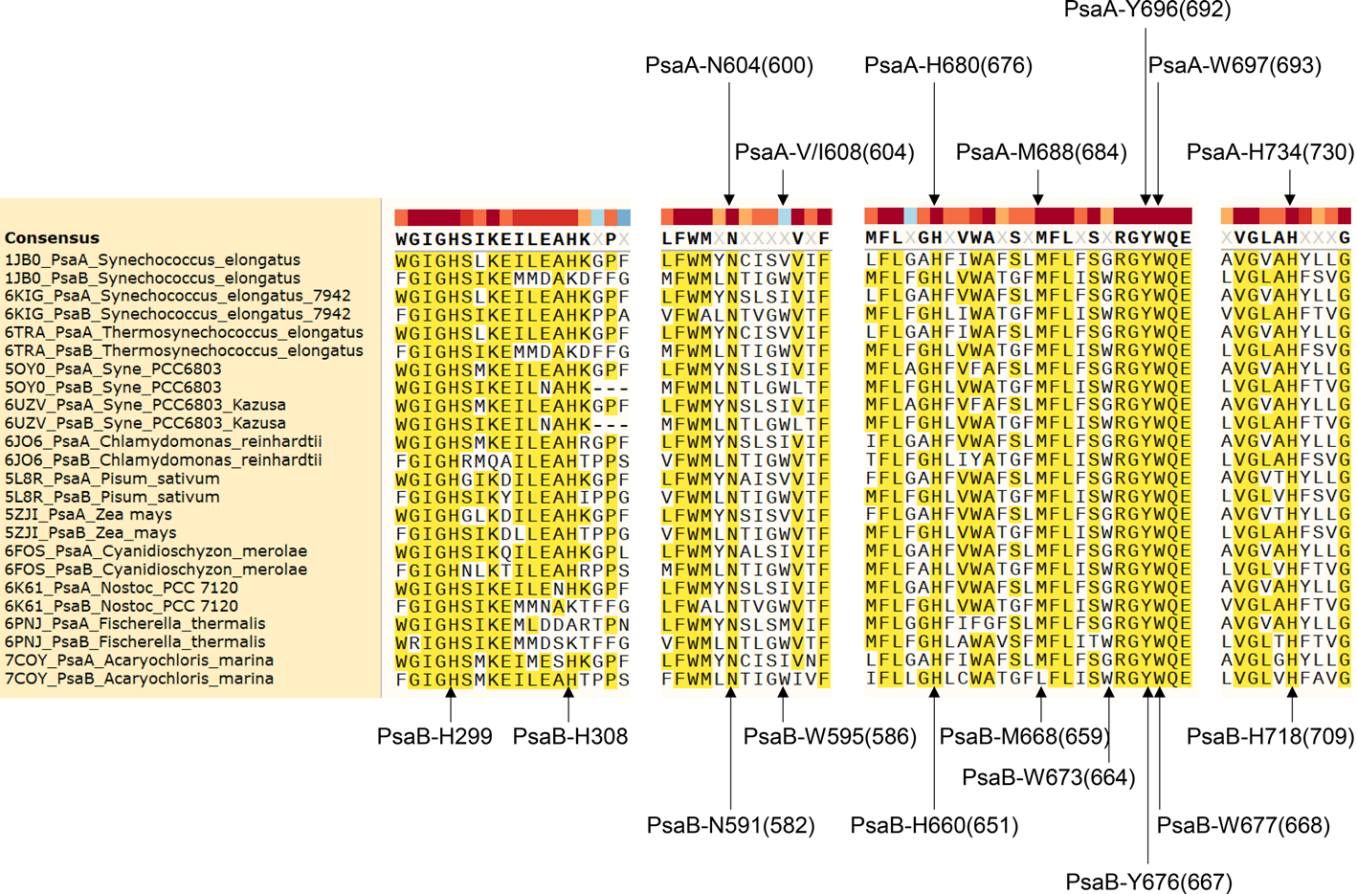
The multiple sequence alignment of PsaA and PsaB from the representative organisms showing the conserved residues for closely interacting with P700, A_−1_, A_0_, A_C_, A_1_ and possibly red chlorophyll molecules. The critical residues for interacting P700, A_−1_, A_0_, A_C_, A_1_ and possibly red chlorophyll molecules are labeled. The nomenclatures for *Synechococcus elongatus* and *Synechocystis* sp. PCC 6803 (in the parenthesis) are used

To study the binding sites of the redox cofactors, we included four more PS I structures for increasing diversity of cell-culture conditions, different oligomeric forms and the reaction centers. Two structures, 6KMW (white light) and 6KMX (far-red light) from *Halomicronema hongdechloris*, a cyanobacterium that produces Chl *f*; one monomeric PS I structure, 6HQB from the model photosynthetic organism *Synechocystis* and one structure, 7COY from far-red light utilizing PS I of *Acaryochloris marina* where Chl *d* and pheophytin are in the reaction center, are included in the study. The amino acids and their positions in PsaA and PsaB were labeled for different species. Because the amino acid positions for closely contacting the cofactors could be different for different species, for labeling the amino acid positions, the multiple sequence alignment analysis was performed (Fig. 6). The amino acids and their positions for P700_A_ and P700_B_ are summarized in Table 1 and those for A_−1_, A_0_ and A_1_ are listed in Supplementary Tables 1, 2 and 3, respectively. The pigment numbers for P700_A_, P700_B_, A_−1A_, A_−1B_, A_0A_, A_0B_, A_CA_ and A_CB_ from different species are shown in Table 2. For the rest of the sections in this study, the nomenclature of *Synechocystis* is used.

**Table 1:** The Amino Acids that Interact with P700_A_ and P700_B_ and Their Positions

PDB	Organism and resolution	Cofactors	Axial ligand	Interacting amino acids		Comments
				PsaA	PsaB	
1JB0	*Synechococcus elongatus* (2.5 Å)	P700A	PsaA-H680	Y603, N604, F611, F676, W683, Y735, T743, F746	L626, W631	CyanobacteriumTrimer
		P700B	PsaB-H660		F598, W625, F656, W663, Y723, T726, Y727, F730	
5OY0	*Synechocystis sp. PCC 6803* (2.5 Å)	P700A	PsaA-H676	Y599, N600, F607, F672, F679, Y731, T739, F742	F617, W622	CyanobacteriumTrimer
		P700B	PsaB-H651		F589, W616, F647, W654, Y714, T717, Y718, F721	
6HQB	*Synechocystis sp. PCC 6803* (4.0 Å)	P700A	PsaA-H676	Y599, N600, F607, F672, F679, Y731, T739, F742	F617, W622	CyanobacteriumMonomer
		P700B	PsaB-H651		F589, W616, F647, W654, Y714, T717, Y718, F721	
6JO6	*Chlamydomonas reinhardtii* (2.9 Å)	P700A	PsaA-H676	Y600, N601, F608, F672, W679, Y731, T739, F742	L621, W626	Green Alga Trimer
		P700B	PsaB-H655		F593, W620, F651, W658, Y718, T721, Y722, F725	
6KMW	*Halomicronema hongdechloris C2206* (2.35 Å)	P700A	PsaA-H689	Y612, N613, F620, F685, W692, F744, T752, F755	L628, W633	CyanobacteriumTrimer, Chl *f,* White light
		P700B	PsaB-H662		F600, W627, F658, W665, Y725, T728, Y729, F732	
6KMX	*Halomicronema hongdechloris C2206* (2.41 Å)	P700A	PsaA-H709	Y632, N633, F640, F705, F712, Y764, T772, F775	F630, W635	CyanobacteriumTrimer, Chl *f,* Far-red light
		A0B	PsaB-H664		F602, W629, F660, W667, Y726, T729, Y730, F733	
6PNJ	*Fischerella thermalis PCC 7521* (3.19 Å)	P700A	PsaA-H713	Y636, N637, F644, F709, F716, Y768, T776, F779	F627, W632	CyanobacteriumTrimer, Far-red light
		P700B	PsaB-H661		F599, W626, F657, W664, Y723, T726, Y727, F730	
7COY	*Acaryochloris marina MBIC11017* (2.5 Å)	P700A	PsaA-H678	Y601, N602, F609, F674, W681, Y733, S741, F744	L623, W628	CyanobacteriumTrimer, Far-red light
		P700B	PsaB-H657		F595, W622, F653, W660, Y720, T723, Y724, F727	
5ZJI	*Zea mays* (3.3 Å)	P700A	PsaA-H675	Y598, N599, F606, F671, W678, Y730, T738, F741	F620, W625	Plants, Trimer
		P700B	PsaB-H654		F592, W619, F650, W657, Y717, T720, Y721, F724

**Table 2:** The Chl Chains and Numbers and the Amino Acids that Provide Axial Ligands to P700, A_−1_, A_0_ and A_C_

PDB	Organism and resolution	P700		A-1		A0		AC	
		P700A	P700B	A-1A	A-1B	A0A	A0B	ACA	ACB
1JB0	*Synechococcus elongatus* (2.5 Å)	A, 1011, CLA, PsaA-H680	B, 1021, CLA, PsaB-H660	B, 1012, CLA, PsaB-N591	A, 1022, CLA, PsaA-N604	A, 1013, CLA, PsaA-M688	B, 1023, CLA, PsaB-M668	A, 1140, CLA, PsaA-H734	B, 1239, CLA, PsaB-H718
5OY0	*Synechocystis sp. PCC 6803* (2.5 Å)	A, 1011, CLA, PsaA-H676	B, 1021, CLA, PsaB-H651	A, 1012, CLA, PsaB-N582	B, 1022, CLA, PsaA-N600	A, 1013, CLA, PsaA-M684	B, 1023, CLA, PsaB-M659	A, 1140, CLA, PsaA-H730	B, 1239, CLA, PsaB-H709
6HQB	*Synechocystis sp. PCC 6803* (4.0 Å)	A, 1011, CLA, PsaA-H676	B, 1021, CLA, PsaB-H651	A, 1012, CLA, PsaB-N582	B, 1022, CLA, PsaA-N600	A, 1013, CLA, PsaA-M684	B, 1023, CLA, PsaB-M659	A, 1140, CLA, PsaA-H730	B, 1239, CLA, PsaB-H709
6JO6	*Chlamydomonas reinhardtii* (2.9 Å)	A, 801, CL0 PsaA-H676	B, 802, CLA, PsaB-H655	A, 803, CLA, PsaB-N586	A, 854, CLA, PsaA-N601	A, 802, CLA, PsaA-M684	B, 803, CLA, PsaB-M663	A, 842, CLA, PsaA-H730	B, 840, CLA, PsaB-H713
6KMW	*Halomicronema hongdechloris C2206* (2.35 Å)	A, 801, CL0, PsaA-H689	B, 803, CLA, PsaB-H662	B, 804, CLA, PsaB-N593	B, 801, CLA, PsaA-N613	B, 802, CLA, PsaA-M697	B, 805, CLA, PsaB-M670	A, 841, CLA, PsaA-H743	B, 841, CLA, PsaB-H720
6KMX	*Halomicronema hongdechloris C2206* (2.41 Å)	A, 801, CL0, PsaA-H709	B, 801, CLA, PsaB-H664	B, 802, CLA, PsaB-N595	A, 802, CLA, PsaA-N633	A, 803, CLA, PsaA-M717	B, 803, CLA, PsaB-M672	A, 843, CLA, PsaA-H763	B, 840, CLA, PsaB-H721
6PNJ	*Fischerella thermalis PCC 7521* (3.19 Å)	A, 1011, CL0, PsaA-H713	B, 1021, CLA, PsaB-H661	A, 1012, CLA, PsaB-N592	B, 1022, CLA, PsaA-N637	A, 1013, CLA, PsaA-M721	B, 1023, CLA, PsaB-M669	A, 1140, CLA, PsaA-H767	B, 1239, CLA, PsaB-H718
7COY	*Acaryochloris marina MBIC11017* (2.5 Å)	A, 3101, G9R, PsaA-H678	B, 3003, CL7, PsaB-H657	B, 3002, CL7, PsaB-N588	A, 3103, CL7, PsaA-N602	A, 3102, PHO, PsaA-M686	B, 3004, PHO, PsaB-L665	A, 3143, CL7, PsaA-H732	B, 3026, CL7, PsaB-H715
5ZJI	*Zea mays* (3.3 Å)	A, 801, CL0, PsaA-H675	B, 802, CLA, PsaB-H654	A, 803, CLA, PsaB-N585	A, 854, CLA, PsaA-N599	A, 802, CLA, PsaA-M683	B, 803, CLA, PsaB-M662	A, 842, CLA, PsaA-H729	B, 840, CLA, PsaB-H712

**Fig. 7:**
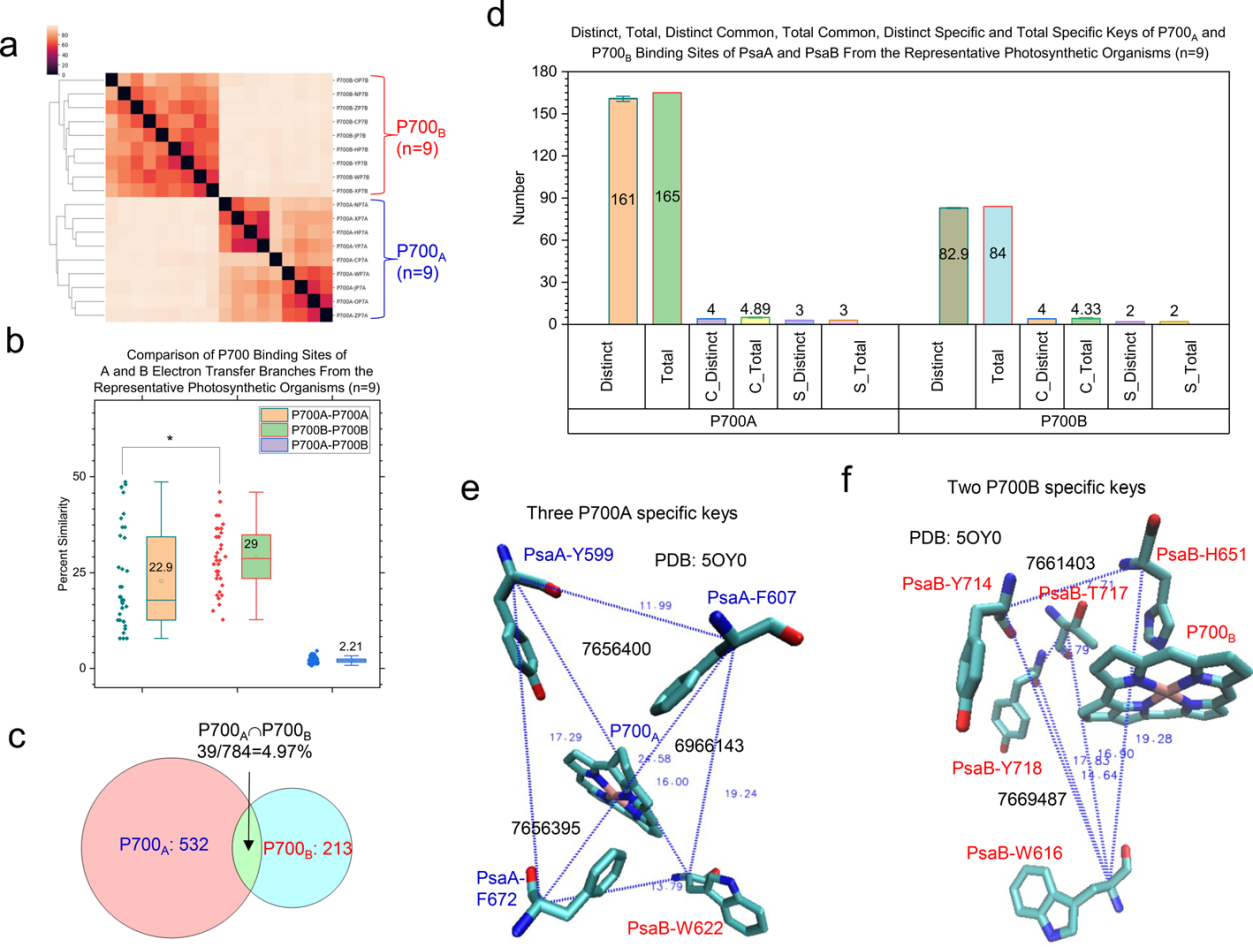
Hierarchical cluster analysis of P700_A_ and P700_B_ molecules demonstrating the capacity of the TSR algorithm for distinguishing their 3D structural differences. Panel **a**, the hierarchical cluster analysis shows 3D structural relationships of P700_A_ and P700_B_. The numbers of P700_A_ and P700_B_ are labeled; panel **b**, structural similarities between P700_A_—P700_A_ pairs, P700_B_—P700_B_ pairs and P700_A_—P700_B_ pairs. The average similarity values, SDs, and 25/75 percentiles are indicated; panel **c**, the Venn diagram showing the numbers of TSR keys exclusively belonging to the P700_A_ group, the P700_B_ group and the intersection between the P700_A_ group and the P700_B_ group; panel **d**, the distinct, total, distinct *common* (C_Distinct), total *common* (C_Total), distinct *specific* (S_Distinct) and total *specific* (S_Total) TSR keys for P700_A_ and P700_B_. The average values and SDs are indicated; panel **e**, the three P700_A_ *specific* TSR keys (6966143, 7656395, 7656400). The triangle corresponding to 6966143 is constructed from the three C_α_ atoms from PsaA-F607, PsaB-W622 and PsaA-F672. The triangle corresponding to the key 7656395 is constructed from the three C_α_ atoms from PsaA-Y599, PsaA-F607 and PsaB-W622. The triangle corresponding to the key 7656400 is constructed from the three C_α_ atoms from PsaA-Y599, PsaB-W622 and PsaA-F672; panel **f**, the two P700_B_ *specific* TSR keys (7661403, 7669487) are shown. The triangle corresponding to the key 7661403 is constructed from the three C_α_ atoms from PsaB-W616, PsaB-H651 and PsaB-Y714. The triangle corresponding to the key 7669487 is constructed from the three C_α_ atoms from PsaB-W616, PsaB-T717 and PsaB-Y718. **e**–**f**, The PDB is 5OY0

**Fig. 8:**
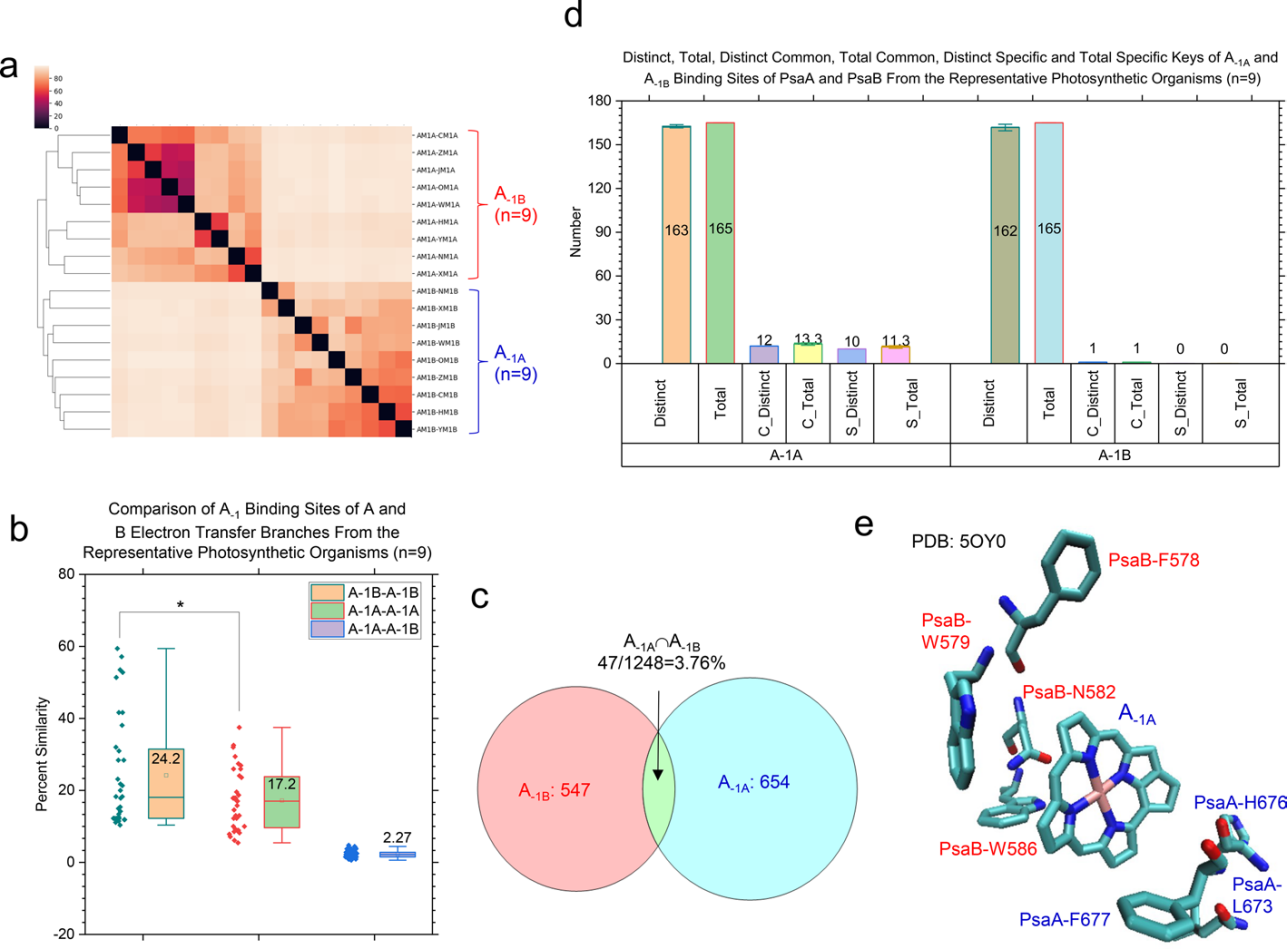
Hierarchical cluster analysis of different types of A_−1A_ and A_−1B_ demonstrating the capacity of the TSR algorithm, which can distinguish their 3D structural differences. Panel **a**, the hierarchical cluster analysis of 3D structural relationships of A_−1A_ and A_−1B_. The numbers of A_−1A_ and A_−1B_ are labeled; panel **b**, the structural similarity between A_−1A_—A_−1A_ pairs, A_−1B_—A_−1B_ pairs and A_−1A_—A_−1B_ pairs. The average similarity values, SDs, and 25/75 percentiles are indicated; panel **c**, the Venn diagram showing the numbers of TSR keys exclusively belonging to the A_−1A_ group, the A_−1B_ group and the intersection between the A_−1A_ group and the A_−1B_ group; panel **d**, the distinct, total, distinct *common* (C_Distinct), total *common* (C_Total), distinct *specific* (S_Distinct) and total *specific* (S_Total) TSR keys for A_−1A_ and A_−1B_. The average values and SDs are indicated; panel **e**, the ten A_−1A_ specific TSR keys. The amino acids associated with these ten keys are PsaA-L673, PsaA-H676, PsaA-F677, PsaB-F578, PsaB-W579, PsaB-N582 and PsaB-W586. The PDB is 5OY0

**Fig. 9:**
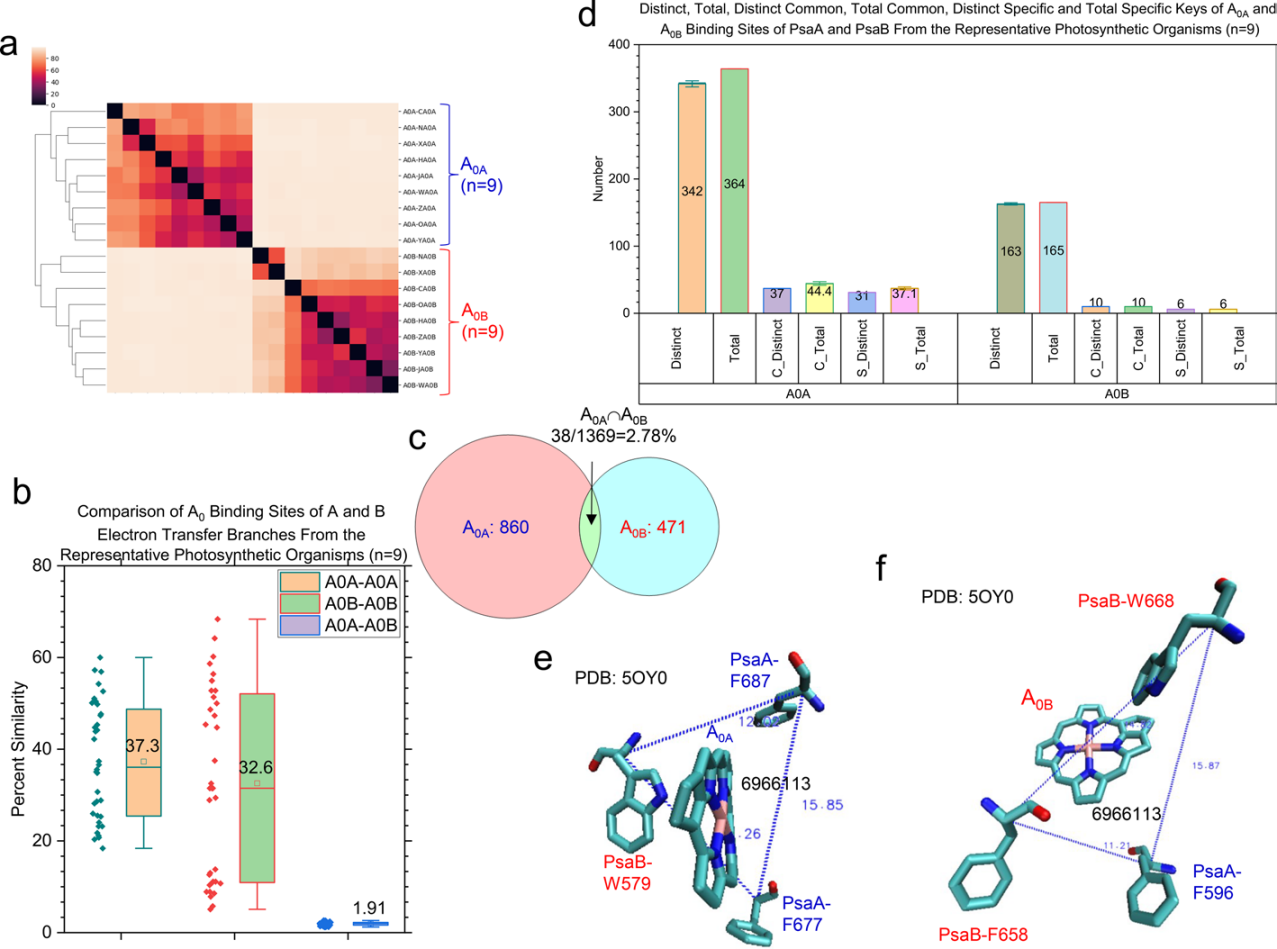
Hierarchical cluster analysis of different types of A_0A_ and A_0B_ demonstrating the capacity of the TSR algorithm for distinguishing their 3D structural differences. Panel **a**, the hierarchical cluster analysis showing 3D structural relationships of A_0A_ and A_0A_. The numbers of A_0A_ and A_0B_ are labeled; panel **b**, the structural similarity between A_0A_—A_0A_ pairs, A_0B_—A_0B_ pairs and A_0A_—A_0B_ pairs. The average similarity values, SDs, and 25/75 percentiles are indicated; panel **c**, the Venn diagram showing the numbers of TSR keys exclusively belonging to the A_0A_ group, the A_0B_ group and the intersection between the A_0A_ group and the A_0B_ group; panel **d**, the distinct, total, distinct *common* (C_Distinct), total *common* (C_Total), distinct *specific* (S_Distinct) and total *specific* (S_Total) TSR keys for A_0A_ and A_0B_. The average values and SDs are indicated; panels **e** and **f**, the A_0_ *common* TSR key (6,966,113). The triangle corresponding to the key 6,966,113 for A_0A_ (**e**) is constructed from the three C_α_ atoms from PsaB-W579, PsaA-F677 and PsaA-F687. The triangle corresponding to the key 6,966,113 for A_0B_ **(f)** is constructed from the three C_α_ atoms from PsaA-F596, PsaB-F658 and PsaB-W668. The PDB is 5OY0

**Fig. 10:**
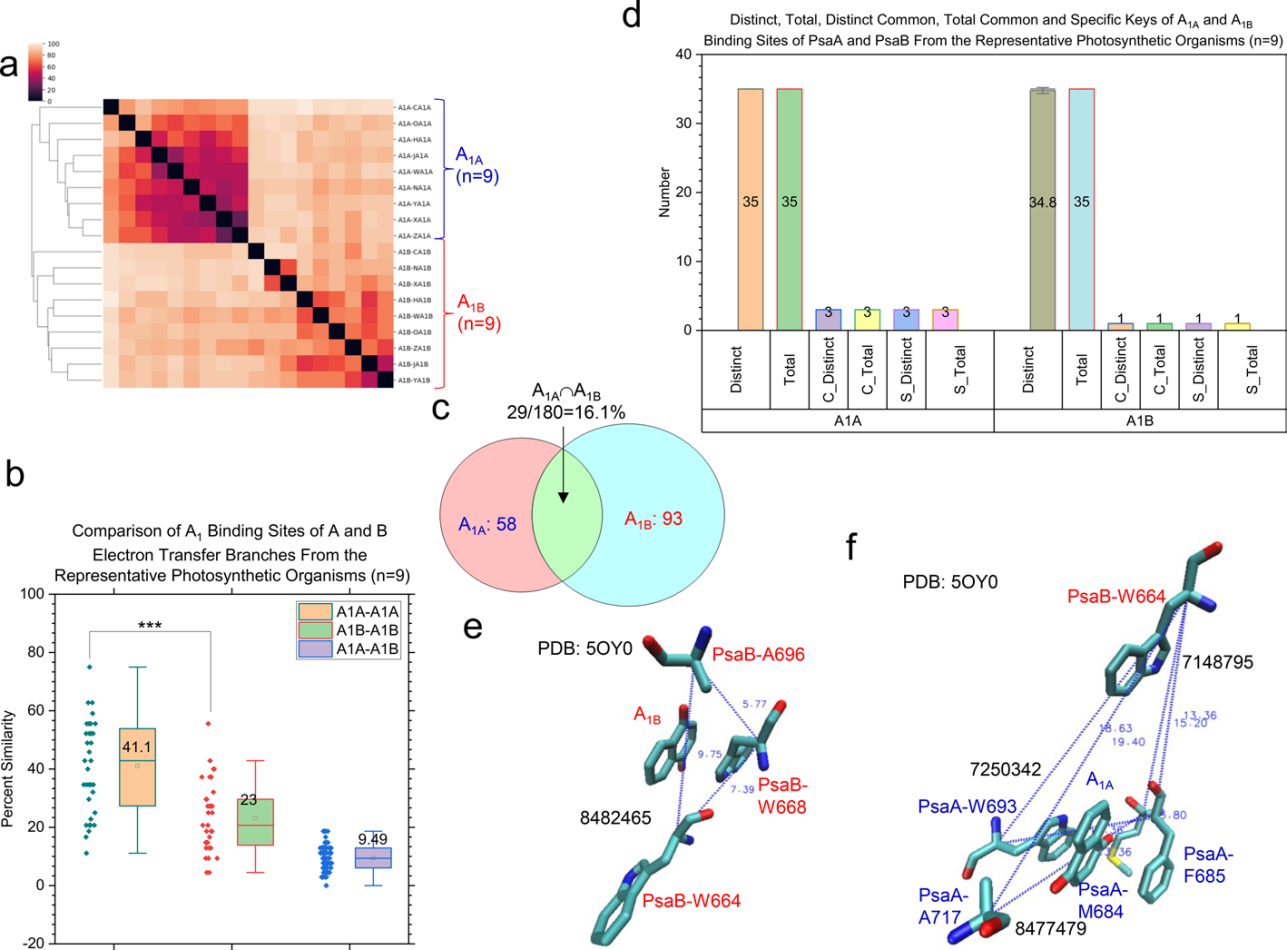
Hierarchical cluster analysis of different types of A_1A_ and A_1B_ demonstrating the capacity of the TSR algorithm for distinguishing their 3D structural differences. Panel **a**, the hierarchical cluster analysis showing 3D structural relationships of A_1A_ and A_1A_. The numbers of A_1A_ and A_1B_ are labeled; panel **b**, the structural similarity between A_1A_—A_1A_ pairs, A_1B_—A_1B_ pairs and A_1A_—A_1B_ pairs. The average similarity values, SDs, and 25/75 percentiles are indicated; panel **c**, the Venn diagram showing the numbers of TSR keys exclusively belonging to the A_1A_ group, the A_1B_ group and the intersection between the A_1A_ group and the A_1B_ group; panel **d**, the distinct, total, distinct *common* (C_Distinct), total *common* (C_Total), distinct *specific* (S_Distinct) and total *specific* (S_Total) TSR keys for A_1A_ and A_1B_. The average values and SDs are indicated; panel **e**, the A_1B_ *specific* TSR key (8482465) is shown. The triangle corresponding to the key 8482465 is constructed from the three C_α_ atoms from PsaB-W664, PsaB-W668 and PsaB-F696; panel **f**, the three A_1A_ *specific* TSR keys (7148795, 7250342, 8477479). The triangle corresponding to the key 7148795 is constructed from the three C_α_ atoms from PsaB-W664, PsaA-M684 and PsaA-F685. The triangle corresponding to the key 7250342 is constructed from the three C_α_ atoms from PsaB-W664, PsaA-F685 and PsaA-W693. The triangle corresponding to the key 8477479 is constructed from the three C_α_ atoms from PsaB-W664, PsaA-M684 and PsaA-A717; panels **e**–**f**, The PDB is 5OY0

The hierarchical cluster analysis clearly shows that the binding sites of each type of redox cofactors form their own clusters. The resulting four clusters: P700, A_−1_, A_0_ and A_1_ can further be divided into two distinct subclusters: one for the binding sites in PsaA and the other for their corresponding sites in PsaB (Supplementary Fig. 18a). This result demonstrates the structural characteristics of the binding sites of each type of redox cofactors from either PsaA side or PsaB side and suggests a difference in redox potential of the cofactors between both sides. The pairwise structural similarities of the binding sites of all redox cofactors are shown in Supplementary Fig. 18b. The P700_B_ binding sites are more conserved (Fig. 7a) and have a higher structural similarity (Fig. 7b) than the P700_A_ binding sites. The P700_A_ group and the P700_B_ group share a small portion of the keys, suggesting a great difference between P700_A_ and P700_B_ environments (Fig. 7c). The *common* and *specific* keys for the binding sites of P700_A_ and P700_B_ were identified (Fig. 7d). Three P700_A_-*specific* keys were shown in Fig. 7e (details in Supplementary 19a) whereas two P700_B_-*specific* keys were illustrated in Fig. 7f (details in Supplementary 19b). The results obtained from the A_−1A_ and A_−1B_ binding sites (Fig. 8a-d) are similar to those from the P700_A_ and P700_B_ binding sites (Fig. 7a-d). Ten A_−1A_-*specific* keys were identified and those ten triangles are from three residues (L673, H676 and F677) of PsaA and four residues (F578, W579, N582 and W586) of PsaB (Fig. 8e and Supplementary Fig. 20). No A_−1B_-*specific* keys were found (Fig. 8d), suggesting more structural diversity for the binding sites of A_−1A_ than those of A_−1B_. No *common* keys were identified for the binding sites of P700 (both P700_A_ and P700_B_) and for those of A_−1_ (both A_−1A_ and A_−1B_). The results from the A_0_ binding sites (Fig. 9a-d) are similar to the binding sites of P700 and A_−1_. One *common* key was identified for the binding sites of both A_0A_ (Fig. 9e) and A_0B_ (Fig. 9f). The A_0A_-*specific* and A_0B_-*specific* keys are shown in Supplementary Figs. 21 and 22, respectively. The binding sites of A_1A_ are more conserved (Fig. 10a) and have a higher structural similarity (Fig. 10b) than those of A_1B_. The structural relationships of the binding site of the A_1A_ and A_1B_ groups combined as well as the individual A_1A_ and A_1B_ binding site groups are shown in Fig. 10c-d, respectively. One *specific* key (Fig. 10e) and three *specific* keys (Fig. 10f) (details in Supplementary Fig. 23) were identified for the binding sites of A_1B_ and A_1A_, respectively. Therefore, the hierarchical clustering results demonstrate the difference of the cofactor binding sites between the A and B branches.

### Evaluation of the TSR-based method for quantifying molecular 3D structures

A common approach to understand the functions of a protein is to compare it to other proteins [77]. The existing 3D structure comparison methods can be roughly divided into five categories [7]: sequence-, distance-, secondary structure-, geometry-, and network-based methods. The TSR algorithm is categorized as a geometry-based method. Therefore, we evaluate the TSR-based method in comparison with the RMSD [70] and USR [69] methods. RMSD is a popular measure of structural similarity between protein or drug 3D structures and involves alignment and optimal superposition between matched pairs of atoms [78]. It searches for the lowest RMSD result for both structures. Alignment or superposition is a complex problem because it is challenging to simultaneously optimize the number of equivalent residues and the global differences due to the fact that one may have to be optimized at the expense of the other [79]. An additional challenge can arise when two different global structures are similar in small local regions (e.g., Triad between chymotrypsin and subtilisin) that can be overlooked. USR is a shape similarity technique that is characterized as a non-superposition-based method [80, 81]. To provide a spectrum of comparisons, we decided to compare the methods for proteins at global and local structural levels as well as for electron redox cofactors.

#### Comparison of the TSR-based method with the RMSD and the USR methods for global structures

PsaA and PsaB show a strong sequence homology [82] and have been suggested to evolve via gene duplication [83]. PsaA and PsaB are well preserved in the membrane integral parts while large differences between the two subunits are visible in the loop regions [84]. The TM-align software [71] was used to generate pairwise RMSD scores that were further used as an input for hierarchical clustering. The result clearly shows two clusters. One cluster contains nine PsaA structures and the other cluster contains nine PsaB structures (Supplementary Fig. 24). The result agrees with their functional classification as well as the protein sequence-based phylogenetic study (Supplementary Fig. 25). Adjusted Rand index (ARI) is frequently used in cluster validation, which measures agreement between two partitions: one partition is given by the clustering process and the other is defined by an external criterion. We used the functional classification as the external criterion in this study. The ARI values lie between 0 and 1 and should be interpreted as follows: ARI ≥ 0.90 excellent recovery; 0.80 ≤ ARI < 0.90 good recovery; 0.65 ≤ ARI < 0.80 moderate recovery; ARI < 0.65 poor recovery. As expected, the ARI for the clustering analysis of PsaA and PsaB using the RMSD method is 1.0. The clustering analysis of the same structures using the USR method reveals that the PsaA structures cannot completely separated from the PsaB structures (Supplementary Fig. 26). The ARI value obtained from the USR method is 0.

**Fig. 11:**
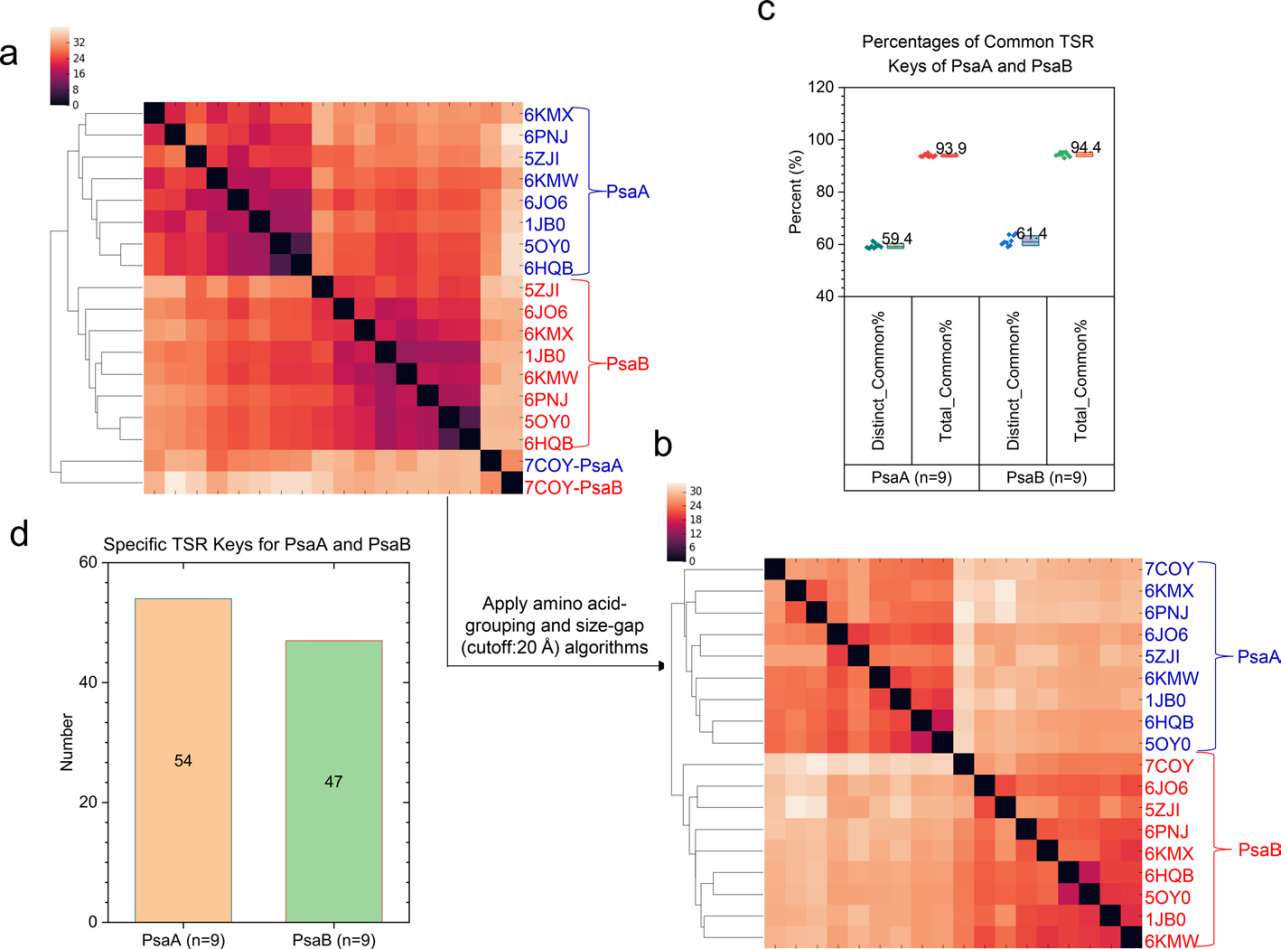
Hierarchical cluster analysis of the representative PsaA and PsaB structures demonstrated the importance of applying the amino acid grouping algorithm and the size-gap algorithm in the TSR-based method. Panel **a**, the hierarchical clustering without applying amino acid-grouping algorithm and the size-gap algorithm; panel **b**, the hierarchical clustering with applying amino acid-grouping algorithm and the size-gap algorithm. The cutoff value for the size-gap algorithm is 20 Å; panels **a–b**, the PDB IDs, PsaA and PsaB are labeled. Blue represents PsaA structures and red represents PsaB structures; panel **c**, the distinct *common*, total *common*, distinct and total TSR keys for each structure of PsaA and PsaB were calculated and the percentages of distinct *common* and total *common* TSR keys are present. Percentage of distinct *common* TSR keys = No. of distinct *common* TSR keys/No. of distinct TSR keys * 100%. Percentage of total *common* TSR keys = No. of total *common* TSR keys/No. total TSR keys * 100%. The average values are labeled and the SDs are shown; panel **d**, the specific keys exclusively belonging to PsaA or PsaB were calculated and are shown; panels **c–d**, number of the structures for PsaA and PsaB are labeled

The same PsaA and PsaB structures as those used in the RMSD and USR studies were used for the TSR-based analysis. The hierarchical clustering shows that eight PsaA structures are clustered together and eight PsaB structures are clustered together. However, the PsaA and PsaB structures from *Acaryochloris marina* are grouped into one cluster (Fig. 11a). The result does not perfectly match with their taxa classification (cyanobacterial PS I vs. green algal PS I vs. plant PS I) probably because the structures of PsaA (685 aa) and PsaB (658 aa) from *Acaryochloris marina* are smaller than the rest of PsaA (717–750 aa) and PsaB (727–740 aa) structures. It was reported that applying the amino acid-grouping algorithm improves the clustering result when two amino acids with similar structures and chemical properties are grouped together [85]. Applying the size-gap algorithm also improves clustering results when a small structure is compared with a large structure [86]. To improve the clustering for the PsaA and PsaB structures, we have applied both the amino acid-grouping and the size-gap algorithms together, we observed an improvement of the cluster analysis (Fig. 11b) and the ARI value achieves 1.0. One of the uniqueness of the TSR algorithm lies in its ability to interpret clustering results using *common* and *specific* TSR keys and to offer valuable insights into the underlying hierarchical relationships of molecular structures within the dataset. It was reported that the common substructure motifs among different protein folds are of critical importance for biological function predictions [87]. Specific substructures exclusively belonging to a particular protein family can be considered as structural characteristics and could be structural foundation for drug development. *Common* (Fig. 11c) and *specific* (Fig. 11d) TSR keys were identified for PsaA and PsaB for a deeper understanding of their relationships.

#### Comparison of the TSR-based method with the RMSD and USR methods for amino acid structures

For the reaction center of PS I, the Mg^2+^ ion, a relatively hard acid, of each monomer of P700 is axially coordinated by a nitrogen atom of a histidine residue whereas A_−1_ and A_0_ are coordinated to a water ligand and soft base sulfur ligand from a methionine residue, respectively [74]. In the X-ray crystal structure of PS I from *Synechocystis*, a water molecule serving as an axial ligand for A_−1A_ is bonded by two hydrogen bonds with PsaB-N582 and with PsaB-W586, which tightly arrange the A_−1A_ binding pocket. In contrast, the PsaB-W586 corresponding residue in PsaA, depending on the species, is Val or Ile and a water molecule that serves as an axial ligand for the A_−1B_ bonded only by one hydrogen bond with PsaA-N600 [17]. Upon the inspections of the structures of PS I [17], we found that PsaA-W597 is close to A_0B_ whereas PsaB-W579 is close to A_0A_. To understand whether the amino acids that directly or indirectly participate in coordination bonds with the redox cofactors have their unique structural characteristics, we focused on His, Asn, Trp and Met. The structural similarities of those amino acids in PsaA and PsaB from different species are shown in Supplementary Figs. 27 (His), 28 (Asn), 29 (Trp) and 30 (Met). A trial analysis of one structure (PDB: 5OY0) indicates that the histidine residues (PsaA-H676 and PsaB-H651) coordinated to P700_A_ or P700_B_ are separated and are in different clusters (Supplementary Fig. 31). The asparagine residues (PsaB-N582 and PsaA-N600) hydrogen bonded to the water with the coordination to A_−1A_ or A_−1B_ are not next to each other (Supplementary Fig. 32). The methionine residues (PsaA-M684 and PsaB-M659) coordinated to A_0A_ or A_0B_ are next to each other and are in the same cluster (Supplementary Fig. 33), whereas tryptophan residues (PsaB-W579 and PsaA-W597) close to A_0A_ or A_0B_ are also next to each other and are in the same cluster (Supplementary Fig. 34). The trial analysis suggests that the amino acids that have close contact with the redox cofactors may (Met and Trp) or may not (His and Asn) have their unique structural characteristics. Therefore, histidine and asparagine residues were excluded from the follow-up analyses.

**Fig. 12:**
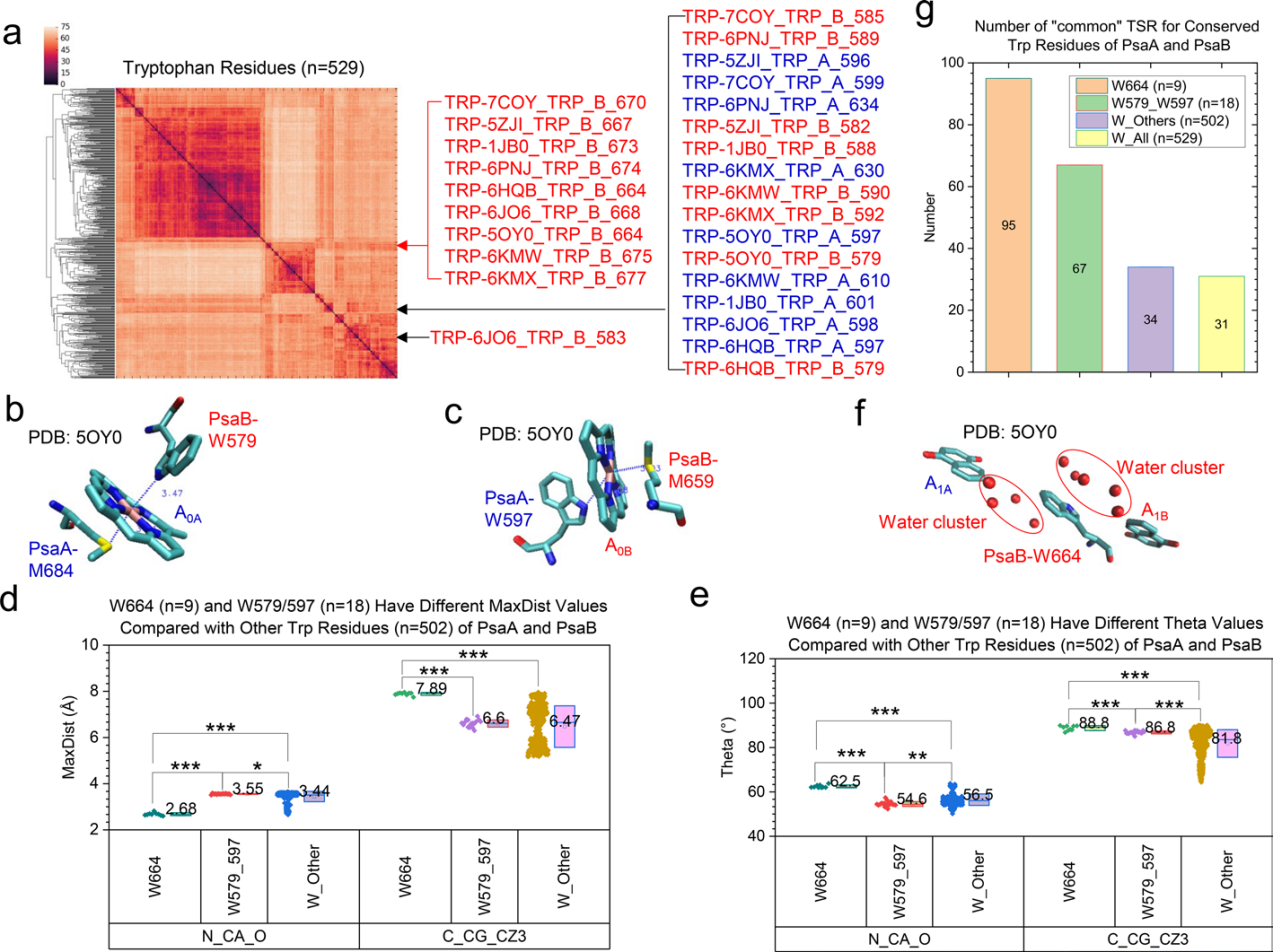
The tryptophan residues closely interacting with A_0_ and A_1_ and their structural characteristics. Panel **a**, the hierarchical cluster analysis shows 3D structural relationships of the tryptophan residues of PsaA and PsaB from diverse organisms. The PDB IDs, the number of tryptophan and nine adjacent tryptophan residues as well as seventeen adjacent tryptophan residues are labeled. One tryptophan residue that is close to A_0_ and separated from the remaining seventeen tryptophan is labeled too; panel **b**, the residues closely interacting with A_0A_; panel **c**, the residues closely interacting with A_0B_; panel **d**, the MaxDist values for the tryptophan residues that are close to A_0_ and A_1_ and for the rest of tryptophan residues were calculated; panel **e**, the Theta values for the tryptophan residues that are close to A_0_ and A_1_ and for the rest of tryptophan residues were calculated; panels **d**–**e**, * means a *p* value is less than 0.05 using a *t*-test, ** means a *p* value is less than 0.01 and *** means a *p* value is less than 0.001 using a *t*-test; panel **f**, the residues close to A_1A_, A_1B_ and the water cluster; panels **b**, **c**, **f**, the PDB is 5OY0; panel **g**, numbers of common TSR keys for the tryptophan residues that are close to A_1_, A_0_, and the rest of tryptophan residues and all tryptophan residues were calculated. Average numbers are labeled

To verify the structural characteristics of Met and Trp, eight more PDB structures were included in the subsequent analyses. The hierarchical clustering result shows that fourteen out of seventeen methionine residues that coordinated with A_0_ are grouped together. However, the rest three methionine residues are separated from the fourteen methionine residues. Therefore, we focus on the rest of the discussions only on tryptophan residues. Eighteen tryptophan residues are close to either A_0A_ or A_0B_. Seventeen of all these 18 tryptophan residues are next to each other in the hierarchical cluster analysis (Fig. 12a). The examples of the close interactions between Trp and A_0_ are illustrated in Fig. 12b (close interaction between PsaB-W579 and A_0A_) and **12c** (close interaction between PsaA-W597 and A_0B_). These 18 tryptophan residues have different MaxDist (Fig. 12d) and Theta (Fig. 12e) values compared with other tryptophan residues in PsaA and PsaB. Interestingly, we also found that the particular tryptophan residues from nine PDB structures group together. PsaB-Trp664 (PDB: 5OY0) separates two water clusters between A_1A_ and A_1B_ (Fig. 12f). These 9 tryptophan residues have their structural characteristics because they have unique MaxDist (Fig. 12d) and Theta (Fig. 12e) values. The Trp664 residues from different species have more *common* TSR keys than the tryptophan residues of 579 and 597 (Fig. 12g), suggesting that the Trp664 residues are structurally more conserved.

The corresponding residue of PsaB-Trp664 in PsaA is Gly689. PsaB-W664 has been suggested to play a role in the electron transfer acting as an electron acceptor between A_1B_ and F_X_ [88]_._ The functional studies demonstrated that (i) PS I with doubly protonated quinone in the A_1_ binding site of the mutant with PsaB-W664F are not functional in electron transfer. However, the electron transfer functionality can be restored by incubating the light-treated mutant PS I sample in the presence of added phylloquinone [89]. (ii) PsaB‑Trp664 is essential for the high‑efficiency electron transfer between the phylloquinones and the iron‑sulfur clusters [90]. Considering the structural analysis in this study and the published functional studies, we can link the structural characteristics of Try664 to their functions. As the result, we conclude that tryptophan residues, closely contacting with A_0_, have their specific structural characteristics and the tryptophan residues, separating two water clusters between A_1A_ and A_1B_, are structurally conserved.

To evaluate the performance of the TSR-based method, we used the RMSD and USR methods to study the same tryptophan residues that were analyzed by the TSR algorithm. The cluster analysis using the RMSD method shows that eight of 9 Trp664 are grouped together and thirteen of 18 Trp579—Trp597 are grouped next together (Supplementary Fig. 35). Based on the fact that all 9 Trp664 are grouped together and seventeen out of 18 Trp579—Trp597 are grouped when the TSR algorithm is used (Fig. 12a), we conclude that the TSR-base method is better for the cluster analysis of the tryptophan residues than the RMSD method. The performance of the USR method on the tryptophan cluster analysis is worse (Supplementary Fig. 36) than the performances of the RMSD (Supplementary Fig. 35) and the TSR (Fig. 12a) algorithms.

#### Comparison of the TSR-based method with the RMSD and USR methods for structures of redox cofactors

**Fig. 13:**
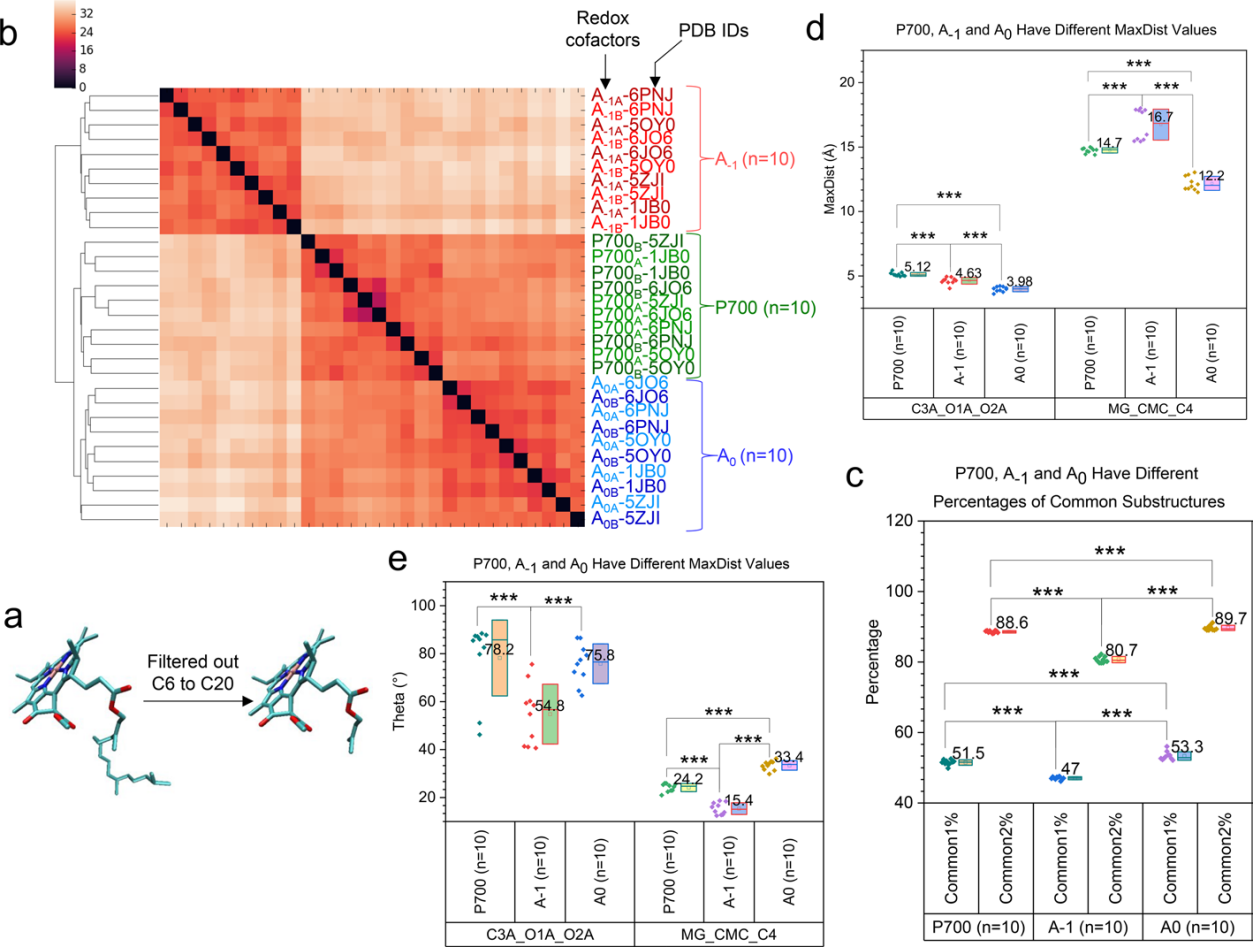
Hierarchical cluster analysis of P700, A_−1_ and A_0_ from different species. Panel **a**, the tails of chlorophyll molecules from carbon 6 to carbon 20 are not included in the study; panel **b**, the hierarchical cluster analysis of the redox cofactors: P700, A_−1_ and A_0_. Redox cofactors, PDB IDs and numbers of structures are labeled; panel **c**, percentages of distinct *common* (Common1%) and total *common* (Common2%) TSR keys were calculated and are present. The definitions for percentage of distinct and total *common* TSR keys were defined in Fig. 13 panel **c**; panels **d**–**e**, the MaxDist (**d**) and Theta (**e**) values for two triangles (C3A-O1A-O2A and MG-CMC-C4) of P700, A_−1_ and A_0_ were calculated and are present; panels **c**–**e**, *** means a *p* value is less than 0.001 using a *t*-test

Phytol tail of chlorophyll anchors the pigment to membranes of thylakoids and maintains the orientation of the pigment. The chlorin ring of redox cofactors, not the phytol tail, is directly involved in electron and energy transfer. The clustering result shows that the redox cofactors are grouped into the P700 cluster, the A_−1_ cluster and the A_0_ cluster (Fig. 3a). In this study up to now, all the atoms of the cofactors have been used in generating the TSR keys. Chl *a*’s tail has 20 carbons. To further study the structural characteristics of the redox cofactors, we have developed a feature selection method in the TSR key generation step to filter out the carbons 6 to 20 of the phytol tails (Fig. 13a). The new hierarchical clustering result shows three clusters (ARI: 1.0): P700, A_−1_ and A_0_ (Fig. 13b). The P700 cluster and A_0_ cluster are joined into the P700—A_0_ cluster that is then merged with the A_−1_ cluster (Fig. 13b). It reveals that P700 and A_0_ from different species are structurally similar and A_−1_ are structurally different from either P700 or A_0_. It is unclear whether the structural similarity is related to their functions. P700_A_ and P700_B_ do not form their own clusters (Fig. 13b), suggesting P700_A_ are not structurally different from P700_B_ among different species. The same situation is observed for A_−1A_ and A_−1B_ as well as A_0A_ and A_0B_ (Fig. 13b). Interestingly, we have observed that A_0A_ and A_0B_ are grouped into a single cluster for each species (each PDB entry) (Fig. 13b). The hierarchical structural relationships of the redox cofactors can be described as (i) two A_0_ clusters of thermophilic cyanobacterium *T. vestitus* and cyanobacterium *Synechocystis* are joined into a large cluster, and two A_0_ clusters of filamentous true-branching cyanobacterium *Fischerella thermalis* and a single-cell green alga *Chlamydomonas reinhardtii* are joined into another large cluster; (ii) the two large clusters from (i) are merged into a large cluster; (iii) The larger cluster is merged with the A_0_ cluster of the plant (Fig. 13b). The result indicates A_0_ structures are species-specific. It is not the case for P700 and A_−1_ (Fig. 13b). To interpret the clustering results, we have calculated *common* and *specific* TSR keys for P700, A_−1_ and A_0_. The percentages of *common* TSR keys are different for P700, A_−1_ and A_0_ (Fig. 13c), suggesting different structural similarities for the redox cofactors. In addition, two triangles (C3A-O1A-O2A and MG-CMC-C4) have different MaxDist (Fig. 13d) and Theta (Fig. 13e) values for P700, A_−1_ and A_0_, demonstrating the geometrical differences of different cofactors. Taken together, the *common* and *specific* TSR keys explain the clustering result (P700 cluster, A_−1_ cluster and A_0_ cluster).

The cluster analysis of the redox cofactors using the RMSD method clearly shows three clusters: P700, A_−1_ and A_0_ clusters (Supplementary Fig. 37) that agree with their functional classification (ARI: 1.00) and match with the three clusters from the TSR algorithm (ARI: 1.00). In contrast, the USR method can distinguish A_−1_ structures from those of P700 and A_0_ (Supplementary Fig. 38). However, it cannot distinguish P700 structures from A_0_ structures (Supplementary Fig. 38) (ARI: 0.544) (Supplementary Table 4). If we look closer at the subclusters from the RMSD method, there are two subclusters for the A_0_ clusters: one is for A_0A_ (A branch) and the other is for A_0B_ (B branch) (Supplementary Fig. 37). The A_0_ structures are branch-specific (A branch and B branch) for the RMSD method (Supplementary Fig. 37), not species-specific observed from the TSR algorithm (Fig. 13b). The P700 structures are also branch-specific for the RMSD method (Supplementary Fig. 37). However, it is not the case for the A_−1_ structures (Supplementary Fig. 37).

## Discussions

Significant numbers of aromatic residues are found in the binding sites of P700, A_−1_, A_0_ and A_1_. The exact functions of aromatic residues in the binding sites of the cofactors are unclear. We discovered that the tryptophan residues of PsaA and PsaB close to A_0B_ and A_0A_, respectively, are structural conserved among different photosynthetic organisms. Trp has been reported to be involved in light-triggered electron transfer [91]. The studies highlight the generality of Trp-porphyrin electron transfer events in heme proteins [92]. The exact function of the tryptophan residues (PsaA-Trp597 and PsaB-Trp579) that are close to A_0_ has not been studied by an experimental approach.

The early version of the TSR-based method has its limitation that can only quantify backbone structures of proteins. This new version allows studying structural complementarity between electron cofactors and their surrounding amino acids. This version of the TSR algorithm was evaluated by comparing it with the RMSD and USR methods. For the quantitative comparisons of the global and local protein structures and the redox factor structures, the TSR-based and RMSD methods outperformed the USR method. For protein global structural and redox cofactor structural comparisons, the results of the hierarchical clustering using the TSR-based method or the RMSD method match with their functional classification. For tryptophan structural comparisons, the TSR-based method outperforms the RMSD method. In addition, the TSR-based method can interpret clustering results using *common* and *specific* TSR keys. In contrast, the RMSD and USR methods have their limitations in interpreting clustering results. Besides the advantages of interpreting results, the TSR-based method has two additional advantages. First, the RMSD method requires pre-alignment or pre-determination of equivalent residues for proteins or equivalent atoms for redox factors. Therefore, the RMSD method has its limitation in comparing two nonhomologous proteins (PsaA vs. PsaL) and two different types of redox cofactors (e.g., chlorophyll vs. phylloquinone). In contrast, the TSR-based method is an alignment-free algorithm. It can be used to quantify two completely different structures. Second, the unique representation of molecular 3D structures by TSR keys (integers) makes substructure search easy and effective. It would be useful if a computational method is able to search for functional substructures similar to catalytic sites, ligand binding sites and other interfacing residues [93]. Such an endeavor requires the availability of a method encoding molecular structures that are indicative of biological activity. Structural complementarity in molecular recognition events is an important indicator of a molecule’s activity because favorable molecular interactions require such complementarity. The TSR algorithm has its uniqueness for quantifying structural complementarity (e.g., cofactor and cofactor binding sites).

## Conclusions and future directions

### Conclusions

A comprehensive study of PS I 3D structures brought the following main findings.

(i) A new version of the TSR-based method was developed to represent 3D structures of pigments and to quantify pigment structures.
(ii) The hierarchical clustering results using *Cofactor* TSR keys reveal that the redox cofactors, P700, A_−1_ and A_0_ form their distinct clusters, suggesting their specific structural characteristics. For example, the two triangles (C3A-O1A-O2A and MG-CMC-C4) have different geometries for P700, A_−1_ and A_0_.
(iii) The results using *Cofactor* and *CA* TSR keys demonstrate the structural differences of the redox cofactors, P700, A_−1_, A_0_ and A_1_, as well as their binding sites between A branch and B branch.
(iv) Different types of TSR keys were used to show common substructures shared by different types of redox cofactors or their binding sites as well as unique substructures exclusively belonging to a certain type of cofactors or their binding sites.
(v) The hierarchical clustering results show that the tryptophan residues close to A_0_ from different species were clustered together as well as the tryptophan residues splitting the water cluster near A_1A_ and A_1B_ binding sites were grouped together. The results demonstrate that the tryptophan residues close to A_0_ are structurally conserved. The tryptophan residues splitting the water cluster are also structurally conserved (e.g., N-CA-O triangle and C-CG-CZ3 triangle have different geometries between Trp664 and Trp579-Trp.). These structurally conserved residues imply their specific functional roles.
(vi) In term of hierarchical clustering results, the TSR-based method outperforms the RMSD and USR methods. In term of computational cost, the USR method runs faster than the RMSD and TSR methods for global protein structural comparisons.

In summary, this study of structural relationships of pigments and protein local environments provides new evidence for their unique chemical and physical properties of each redox cofactor that modulate the rate and direction of energy and electron transfer. This study builds a solid foundation for future functional studies of PS I complex using experimental approach as well as theoretical analyses, e.g., molecular dynamics simulations or QM/MM calculations. Understanding of the mechanisms underlying energy and electron transfer is essential for developing novel approaches for addressing two challenges being faced by the world: a need for energy sources, and a reduction of greenhouse gas emissions.

### Future directions

The mechanism underlying the interactions between cofactors and protein environments is not fully understood. Thus, how to replicate the same mechanisms in artificial systems is still open to investigation [76]. > 50 PS I structures were included in this study. More PS I structures can be included in the future studies. The reaction centers of PS II have the arrangements similar to those of PS I. PS I and PS II structures can be studied together. In this study, we manually labeled the numbers of each redox factors and A_CA_ and A_CB_, and numbers of the residues that coordinate with Mg^2+^ ions of the cofactors or the water molecules that have the coordination bond with the cofactors. PsaA and PsaB amino acid sequences and residue number assignment of each Chl molecules may not be the same across different species. Therefore, the manual labeling process for each cofactor and their corresponding residues is time-consuming. An algorithm needs to be developed for labeling each cofactor and their coordinating residues. We have developed a method for representing 3D structures of all twenty amino acids and quantifying their structures. Studies showed that His [94–99], Asn [100–103], Trp [88–90, 104, 105] and Met [106–113] play critical roles in modulating properties of the redox cofactors in PS I. Therefore, we discussed four amino acids (His, Asn, Trp and Met) with a focus on Trp in this study. As stated earlier, more structures and all other amino acids (Supplementary Fig. 39) can be included in the future studies.

## Supplementary Information

Additional file1.

## Data Availability

These data were derived from the following resource available in the public domain: https://www.rcsb.org/. The authors confirm that the data supporting the findings of this study are available within the article and its supplementary materials. The source Python codes for this study are available for academic users on GitHub: https://github.com/WuXu26/PSI_TSR. Other TSR-related Python codes can be found from https://github.com/tarikulislammilon/TSR and https://github.com/WuXu26/Protein-3D-TSR.
