# Supplementary material for: Development of a TSR-based method for understanding structural relationships of cofactors and local environments in photosystem I
Source: BMC Bioinformatics. 2025 Jan 14;26:15. doi: 10.1186/s12859-025-06038-y (PMC11731568; doi:10.1186/s12859-025-06038-y)
Supplement: Supplementary file 1 — Additional file1. [file 12859_2025_6038_MOESM1_ESM.pdf]

Supplementary Figure Legend

Supplementary Figure 1. The different types of TSR keys of PsaA and PsaB from diverse organisms. Distinct, total, distinct *common* (C_Distinct) and total *common* (C_Total) TSR keys were calculated for PsaA, PsaB as well as for PsaA and PsaB combined. The average values, SDs, and 25/75 percentiles are indicated. The number of the structures is labeled.

Supplementary Figure 2. The specific TSR keys of PsaA and PsaB from diverse organisms. Distinct *specific* (S_Distinct) and total *specific* (S_Total) TSR keys were calculated for PsaA and PsaB. The average values, SDs, and 25/75 percentiles are indicated. The number of the structures is` labeled.

Supplementary Figure 3. The trimer and monomer *specific* TSR keys for PsaA and PsaB from diverse organisms including the representative *specific* keys. Panel (a), the *specific* keys for trimer and monomer forms of PS I from *Synechocystis* sp. PCC 6803. The average values and SDs are indicated. The number of the structures are labeled; panel (b), the three timer-*specific* TSR keys (9102576, 9264965, 9386804). The triangle corresponding to the key 9102576 is constructed from the three C_α atoms of PsaB-N306, PsaB-H308 and PsaB-H317. The triangle corresponding to the key 9264965 is constructed from the three C_α atoms of PsaB-N306, PsaB-H317 and PsaB-T318. The triangle corresponding to she key 9386804 is constructed from the three C_α atoms of PsaB-H317, PsaB-N319 and PsaB-N404. The surrounding chlorophylls (CLA1218, CLA1219, CLA1220) corresponding to the triangles of PsaB trimer-*specific* keys are also shown; panel (b), the two monomer-*specific* TSR keys (4636575, 7641001). The triangle corresponding to the key 4636575 is constructed from the three C_α atoms of PsaA-H179, PsaA-

H181 and PsaA-K183. The triangle corresponding to the key 7641001 is constructed from the three C_α atoms of PsaA-T2, PsaA-Y180 and PsaA-H181. The surrounding chlorophylls (CLA1108, CLA1109) corresponding to triangles of PsaA monomer-*specific* keys are shown; panel (d), the one monomer-*specific* TSR keys (3372925, 3819483) is shown. The triangle corresponding to the key 3372925 is constructed from the three C_α atoms of PsaB-H242, PsaB-F244 and PsaB-E248. The triangle corresponding to the key 3819483 is constructed from the three C_α atoms of PsaB-H242, PsaB-I243 and PsaB-F244. b-d, The PDB IDs are labeled.

Supplementary Figure 4. The multiple sequence alignment analysis of the part of the aligned sequences of PsaA and PsaB from the trimer and monomer of *Synechocystis* sp. PCC 6803.

Supplementary Figure 5. The multiple sequence alignment analysis of the part of the aligned sequences of PsaA and PsaB from *H. hongdechloris* cells which were cultured under white and red light conditions.

Supplementary Figure 6. The chlorophyll-type *specific* TSR keys of PsaA and PsaB from diverse organisms including the representative *specific* keys.. Panel (a), the *specific* keys for chlorophyll *a*, chlorophyll *d* and chlorophyll *f* of PS I. The average values are indicated and the number of the structures are labeled; panel (b), the two chlorophyll *f-specific* TSR keys (3374957,7984039) of PsaB. The triangle corresponding to the key 3374957 is constructed from the three C_α atoms of PsaB-H443, PsaB-E447 and PsaB-Q456. The triangle corresponding to the key 7984039 is constructed from the three C_α atoms of PsaB-D445, PsaB-E447 and PsaB-V448;

panel (c), one chlorophyll *f-specific* TSR key (5126891) of PsaA is shown. The triangle corresponding to the key 5126891 is constructed from the three C_α atoms of PsaA-M313, PsaA-K325 and PsaA-M327; panel (d), the two chlorophyll *d-specific* TSR keys (7681569, 7681616) of PsaA. The triangle corresponding to the key 7681569 is constructed from the three C_α atoms of PsaA-Y178, PsaA-Y181 and PsaA-H182. The triangle corresponding to the key 7681616 is constructed from the three C_α atoms of PsaA-Y175, PsaA-Y179 and PsaA-H182; panel (e), the two chlorophyll *d-specific* TSR keys (7474587, 8087610) of PsaB. The triangle corresponding to the key 7474587 is constructed from the three C_α atoms of PsaB-C659, PsaB-H715 and PsaB-Y724. The triangle corresponding to the key 8087610 is constructed from the three C_α atoms of PsaB-H715, PsaB-V718 and PsaB-Y721. In panels (b-e) the PDB IDs and chlorophyll identities are labeled.

Supplementary Figure 7. Distinct, total, distinct common and total common TSR keys of a dataset containing P700, A_{-1}, A_0 and A_C. The average values, SDs and the numbers of each type of cofactors are indicated. Note: only A_{0A} from PDB 6PNJ has sixty atoms and the rest of the cofactors have sixty-five atoms.

Supplementary Figure 8. Hierarchical cluster and MDS analyses of $P700_A$ and $P700_B$. Panel ((a), the result of the hierarchical cluster analysis; panel (b), the result from the MDS analysis. These analyses demonstrate that **$P700_A$ and $P700_B$** have their unique structural characteristics. Cofactor TSR keys were used in both analyses.

Supplementary Figure 9. Hierarchical cluster and MDS analyses of the structural

relationships between A_{-1A} and A_{-1B}. Panel (a), the result of the hierarchical cluster analysis; panel (b), the result of the MDS analysis. Cofactor TSR keys were used in both analyses.

Supplementary Figure 10. Hierarchical cluster and MDS analyses of A_{0A} and A_{0B} Panel (a), the result of the hierarchical cluster analysis; panel (b), the result from the MDS analysis. These analyses demonstrate that A_{0A} and A_{0B} have their unique structural characteristics. Cofactor TSR keys were used in both analyses

Supplementary Figure 11. Hierarchical cluster and MDS analyses of A_{CA} and A_{CB}. demonstrate that they likely have their structural characteristics. Panel (a), the result of the hierarchical cluster analysis; b, the result of the MDS analysis. These analyses demonstrate that A_{CA} and A_{CB} have their unique structural characteristics. Cofactor TSR keys were used in both analyses

Supplementary Figure 12. Calculated numbers of the residues of PS I that closely interact with chlorophyll molecules/ The chlorophyll molecules include chlorophyll *a*, chlorophyll *d* and chlorophyll *f*. The average values, SDs, and 25/75 percentiles are indicated. * means a *p* value is less than 0.05 and ** means a *p* value is less than 0.01 using *t*-test. The cutoff value for close interactions is 3.5 Å.

Supplementary Figure 13. The numbers of the residues of PS I that closely interact with different types of chlorophyll molecules. The chlorophyll molecules include chlorophyll *a*, chlorophyll *b*, chlorophyll *d*, chlorophyll *f* and pheophytin. The average values and SDs are

indicated. * means a *p* value is less than 0.05 using *t*-test. The cutoff value for close interactions is 3.5 Å.

Supplementary Figure 14. The numbers of the residues of PsaA and PsaB that closely interact with different cofactors. The cofactors include $P700_A$, $P700_B$, A_{-1A}, A_{-1B}, A_{0A}, A_{0B}, A_{CA}, A_{CB} and the rest of the chlorophyll molecules. The average values and SDs are indicated. * means a *p* value is less than 0.05 and ** means a *p* value is less than 0.01 using *t*-test. The cutoff value for close interactions is 3.5 Å.

Supplementary Figure 15. The numbers of the residues of PsaA and PsaB that closely interact with A_{1A} and A_{1B}. The average values and SDs are indicated. *** means a *p* value is less than 0.001 using *t*-test. The cutoff value for close interactions is 3.5 Å.

Supplementary Figure 16. The amino acid content of PsaA from diverse organisms. The average values and SDs are indicated.

Supplementary Figure 17. The amino acid content of PsaB from diverse organisms. The average values and SDs are indicated.

Supplementary Figure 18. Hierarchical cluster analysis of electron cofactor binding sites. Panel (a), the result of the hierarchical cluster analysis. The numbers of the binding sites in the analysis are labeled; panel (b), the structural similarity of electron binding sites. The average value and SD are labeled. C_α TSR keys are used for data presented in both panels. The cutoff

value for a binding site is 3.5 Å. Hierarchical cluster analysis revealed the structural relationships of electron cofactor binding sites/

Supplementary Figure 19. The three amino acids and their sequence positions for the P700 *specific* TSR keys. Panel (a), the three amino acids and their sequence positions for the three $P700_A$ *specific* TSR keys; panel (b), the three amino acids and their sequence positions for the two P700A *specific* TSR keys.

Supplementary Figure 20. The three amino acids and their sequence positions for the ten A_{-1A} *specific* TSR keys.

Supplementary Figure 21. The three amino acids and their sequence positions for the A_{0A} *specific* TSR keys. Panel (a), the structures of the six amino acids of PsaA and six amino acids of PsaB corresponding to the thirty-one A_{0A} *specific* TSR keys. The PDB ID, amino acids and their sequence positions, and A_{0A} are labeled; panel (b), the three amino acids, their sequence positions for the thirty-one A_{0A} *specific* TSR keys.

Supplementary Figure 22. The three amino acids and their sequence positions for the A_{0B} *specific* TSR keys. Panel (a), the structures of the three amino acids of PsaA and three amino acids of PsaB corresponding to the six A_{0B} *specific* TSR keys. The PDB ID, amino acids and their sequence positions, and A_{0B} are labeled; panel (b), the three amino acids, their sequence positions for the six A_{0B} *specific* TSR keys.

Supplementary Figure 23. The three amino acids and their sequence positions for the three A_{1A} *specific* TSR keys.

Supplementary Figure 24. The hierarchical cluster analysis of PsaA and PsaB from different species using the RMSD method. Pairwise RMSD values were calculated using the TM-align software package. PDB IDs, PsaA, PsaB and numbers of structures are labeled.

Supplementary Figure 25. The phylogenetic analysis of PsaA and PsaB sequences from different species using MEGA. The ClustalW algorithm and Neighbor-Joining method were used for the phylogenetic study of PsaA and PsaB sequences. PDB IDs, PsaA and PsaB are labeled.

Supplementary Figure 26. The hierarchical cluster analysis of PsaA and PsaB from different species using the USR method. PDB IDs, PsaA, PsaB and numbers of structures are labeled.

Supplementary Figure 27. The structural similarity of histidine residues of PsaA and PsaB in diverse organisms. The average values, SDs, 25/75 percentiles, and PDB ID are labeled.

Supplementary Figure 28. The structural similarity of asparagine residues of PsaA and PsaB in diverse organisms. The average values, SDs, 25/75 percentiles, and PDB ID are labeled.

Supplementary Figure 29. The structural similarity of tryptophan residues of PsaA and PsaB in diverse organisms. The average values, SDs, 25/75 percentiles, and PDB ID are labeled.

Supplementary Figure 30. The structural similarity of methionine residues of PsaA and PsaB from diverse organisms. The average values, SDs, 25/75 percentiles, and PDB ID are labeled.

Supplementary Figure 31. Hierarchical cluster analysis of the structural relationships of histidine from the PS I of *Synechocystis* sp. PCC 6803. The PDB is 5OY0 and six histidine residues are labeled. Three histidine residues are adjacent and the other three are also adjacent.

Supplementary Figure 32. Hierarchical cluster analysis of the structural relationships of asparagine from the PS I of *Synechocystis* sp. PCC 6803. The PDB is 5OY0 and six adjacent asparagine residues are labeled.

Supplementary Figure 33. Hierarchical cluster analysis of the structural relationships of methionine from the PS I of *Synechocystis* sp. PCC 6803. The PDB is 5OY0 and two adjacent methionine residues are labeled.

Supplementary Figure 34. Hierarchical cluster analysis of the structural relationships of tryptophan from the PS I of *Synechocystis* sp. PCC 6803. The PDB is 5OY0 and thirteen tryptophan residues are labeled.

Supplementary Figure 35. Hierarchical cluster analysis of the tryptophan residues of PsaA and PsaB from different species using the RMSD method. The tryptophan residues that are close to A_0 and A_1 are labeled.

Supplementary Figure 36. Hierarchical cluster analysis of the tryptophan residues of PsaA and PsaB from different species using the USR method. The tryptophan residues that are close to A_0 and A_1 are labeled.

Supplementary Figure 37. Hierarchical cluster analysis of P700, A_{-1} and A_0 of PsaA and PsaB from different species using the RMSD method. Redox cofactors and PDB IDs are labeled.

Supplementary Figure 38. Hierarchical cluster analysis of P700, A_{-1} and A_0 of PsaA and PsaB from different species using the USR method. Redox cofactors and PDB IDs are labeled.

Supplementary Figure 39. Hierarchical cluster analysis of the structural relationships of the amino acids from PsaA and PsaB from diverse organisms in the binding sites of the electron transfer cofactors. Panel (a), the result of the hierarchical cluster analysis. The amino acids and their numbers are labeled; panel (b), the overall structural similarity of the binding sites of the electron transfer cofactors. In both analyses, the cutoff value for a binding site is 3.5 Å.

Supplementary Figure 1

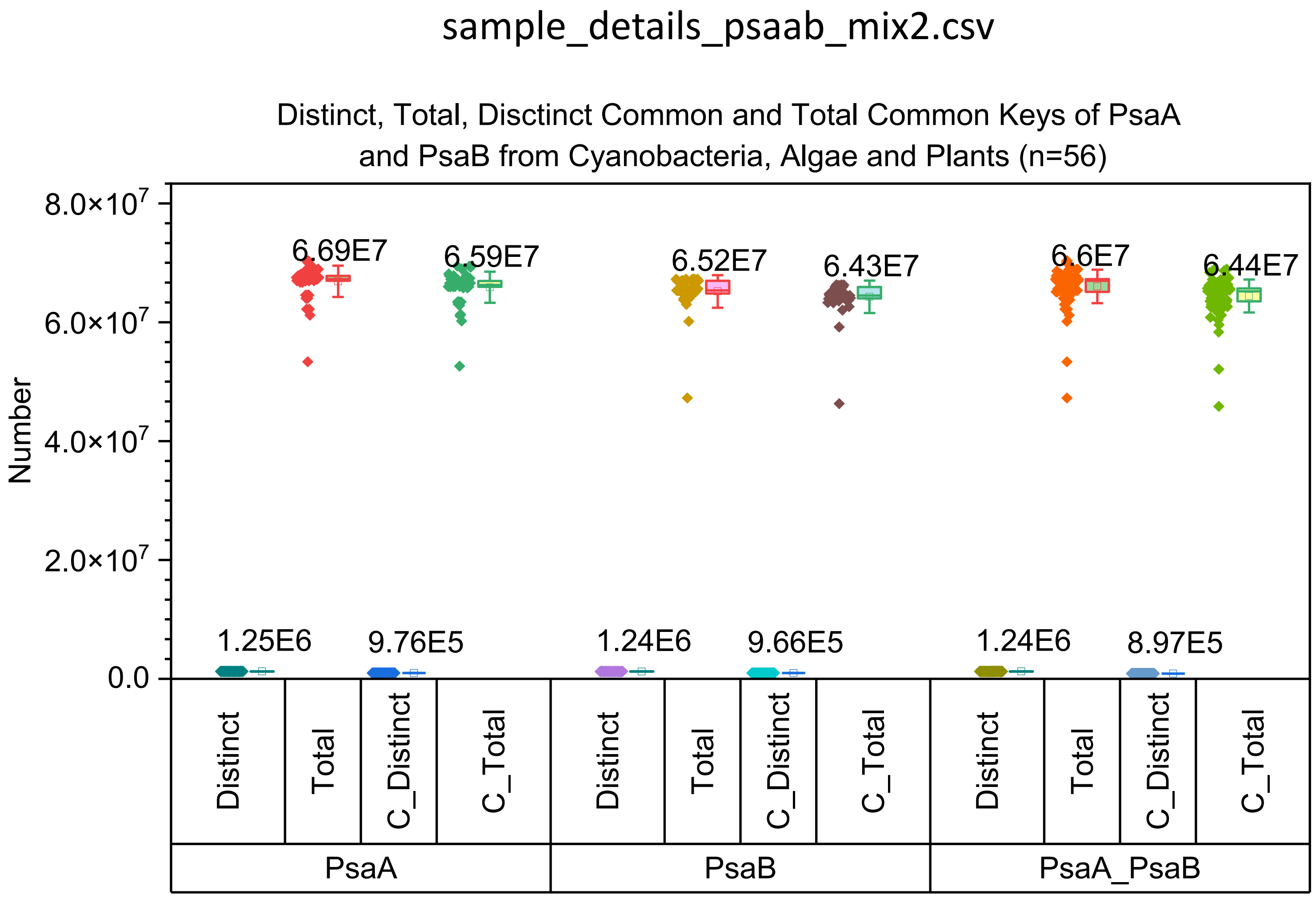

Supplementary Figure 2

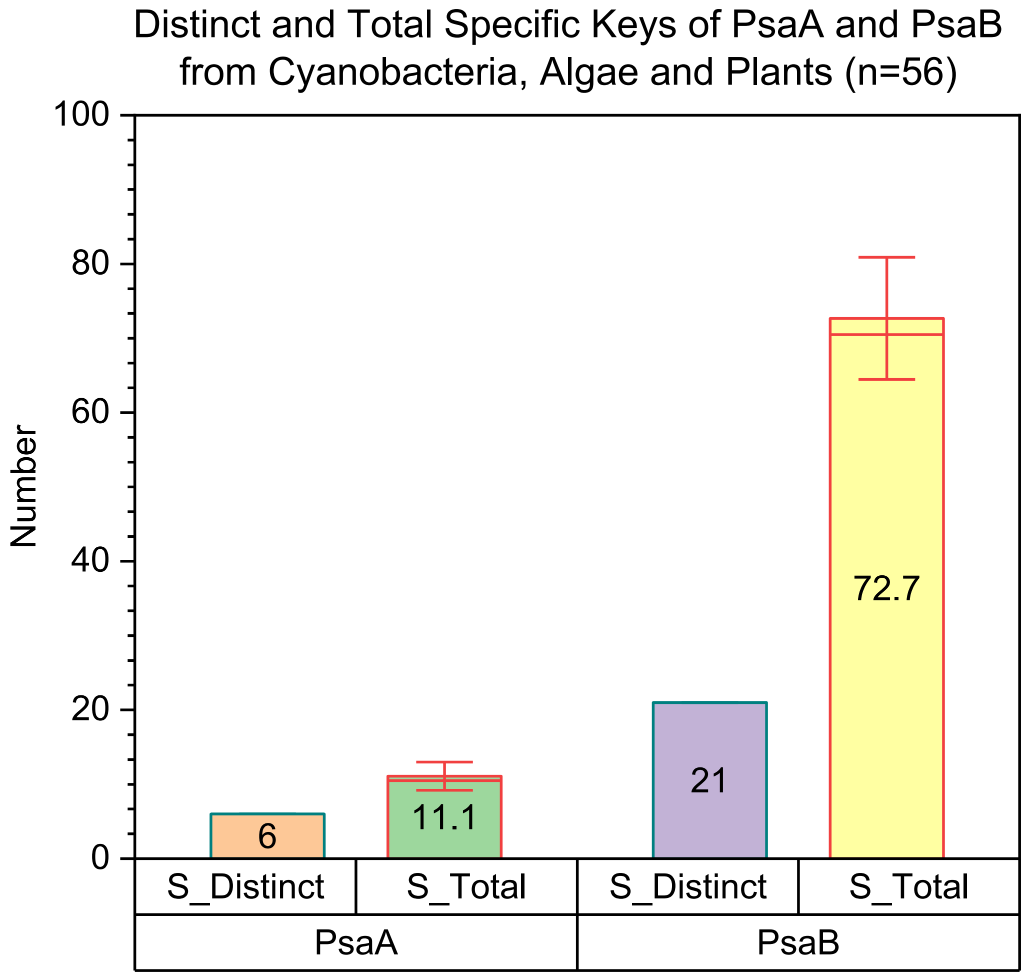

Supplementary Figure 3

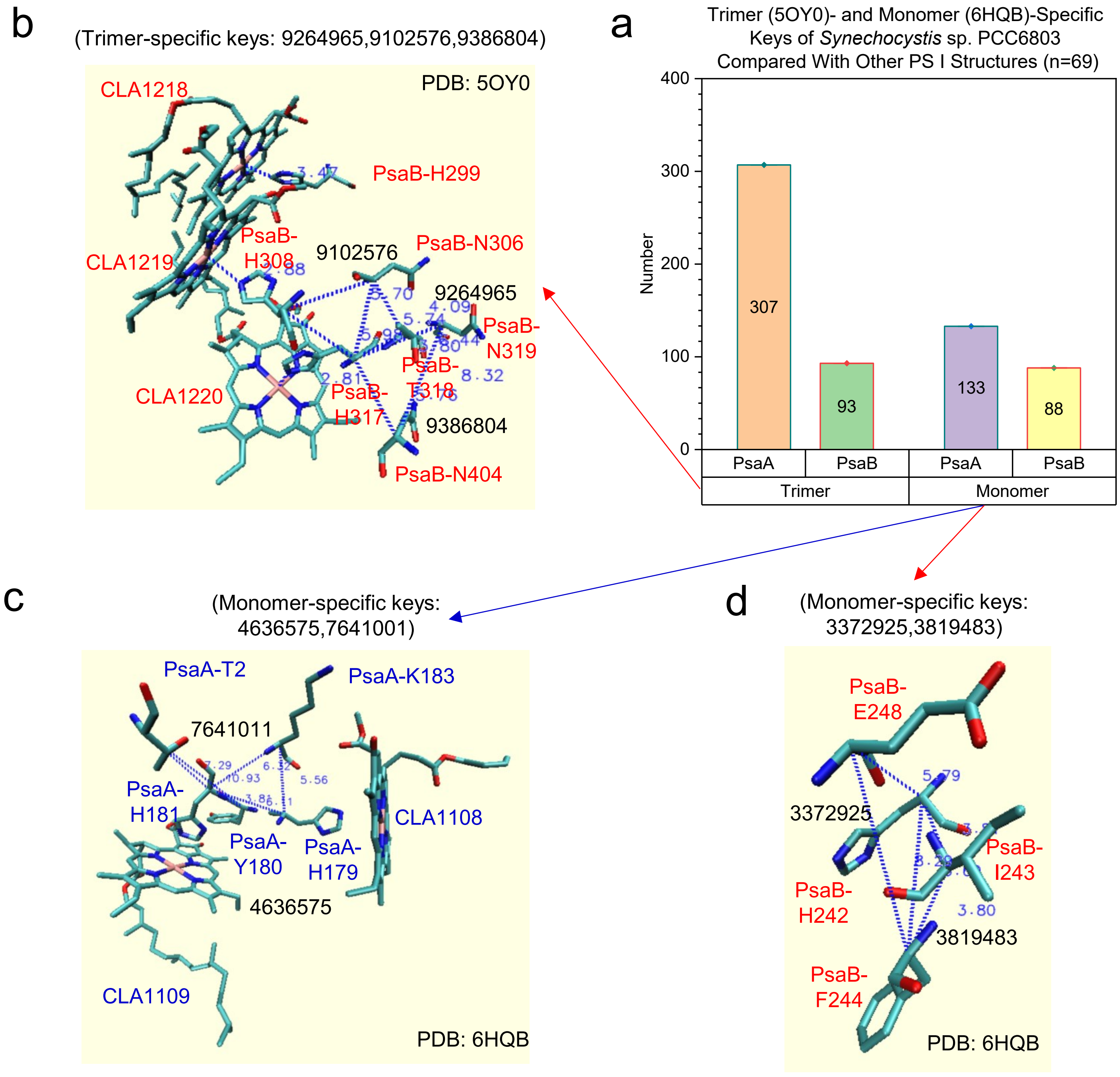

Supplementary Figure 4

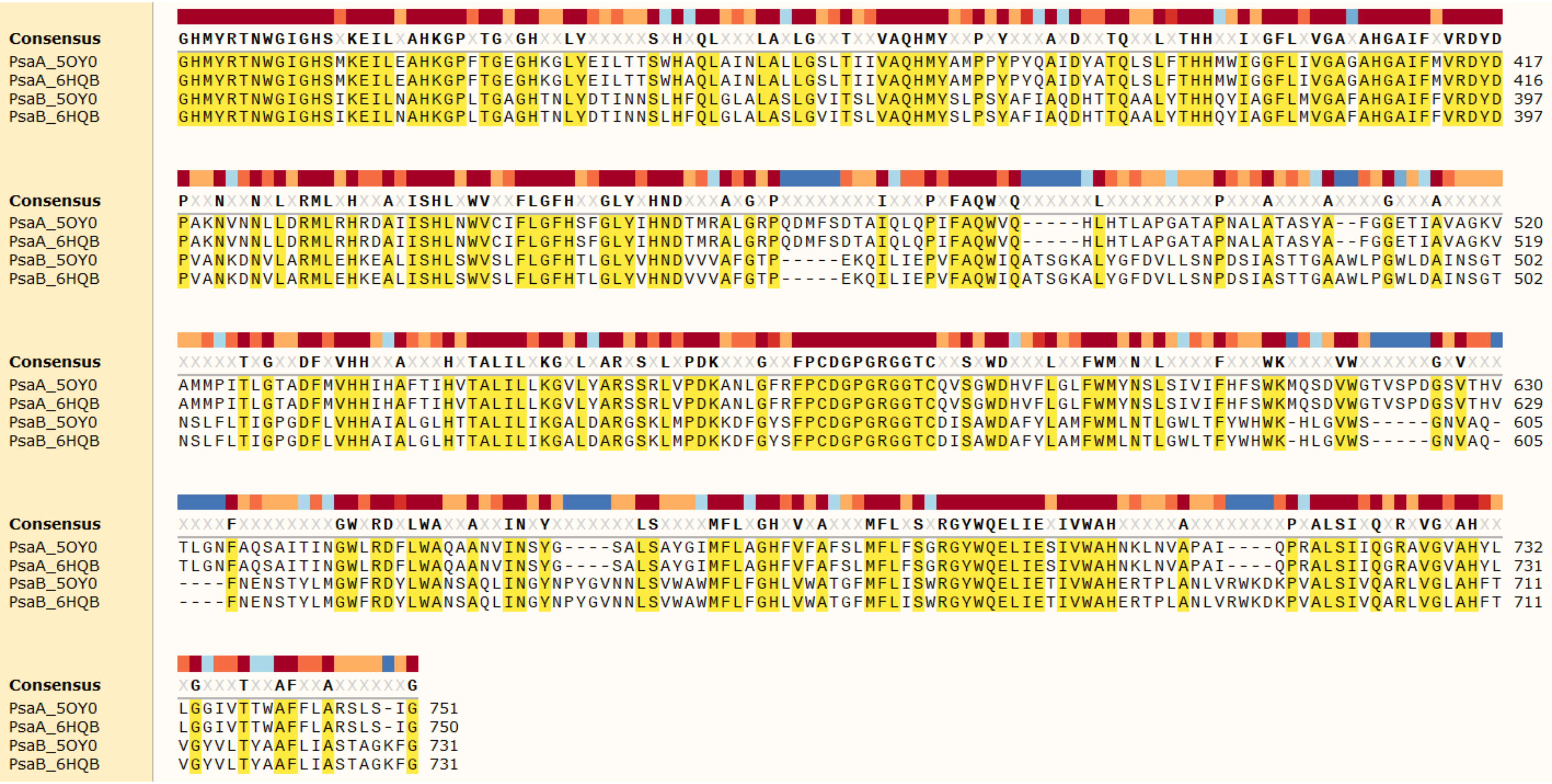

Supplementary Figure 5

```
Consensus   ---XXXXXXXXWXXXXXLXXXXXPXXXEGLXPFFTXNWXXYAXXXXXXXXXXXXXXXXXXXXLTFXGGXXPQTXXLWLTDXAHHHLAIAVLFIVAGHXYRTNXGIGHXXK
PsaA_6KMW   DSVAAIPLPHEWILNPSLMTDIYPSFAEGLKPFFTLNWSVYAD------------------FLTFNGGLNPQTGGLWLTDTAHHHLALAVLFIVAGHFYRTNWGIGHSFK 292
PsaA_6KMX   ---EDIPLPHEFILNKSLMVDLYPSFAEGVKPFFTLNWSAYAD------------------FLTFKGGLNPVTGGLWMTDIAHHHVAIAVLFIIAGHFYRTNWGIGHSFR 279
PsaB_6KMW   ----------GW--DNFLFTPPHP---AGLTPFFTGNWGVYAQNPDTASHVFGSSTGAGSAILTFLGGFHPQTESLWLTDMAHHHLAIAVLFIVAGHMYRTNFGIGHNMK 301
PsaB_6KMX   ----------GW--DNFLSIRPHP---EGLAPLFTGNWGAYAQNPDTAEHAFGTAQGAGSAILTFLGGFHPQTESLWLTDMAHHHLAIAVIFIVAGHMYRTNFGIGHNIK 301

Consensus   EXXXAXXXPXXXXXXXXXGX-GHKGXYXXXXXSXHXQLXWHLAXXGXXXXLVAXHMYXMPPYXXXAXDYXTXXXLXTHHXXIAGFXXVGAXAHXAIFLXRDYDPAXXXNN
PsaA_6KMW   EVLEAHKGP-------VTGE-GHKGMYEIFTTSWHCQLSWNLAWIGSLSILVAHHMYSMPPYPYIATDYPTQLSLFTHHMWIGGFLIVGAGAHAAIFMVRDYDPATHINN 394
PsaA_6KMX   ELLDDARTPKMLPIFNFIGPVGHRGLDKIFETSWHANLAIHLVQFGTASLLVAHHMYAMPPYPYLATDYATVTSLFTHHVWIAGFCIVGGAAHAAIFLVRDYNPADHVNN 389
PsaB_6KMW   EIMNAHNPPQGTPFGGMIGE-GHKGMYDTYNNSLHFQLGWHLACLGVITSLVAQHMYSLPPYAFLAKSYTTQAALYTHHQYIAGFIMVGAFAHGAIFLIRDYDPASNSNN 410
PsaB_6KMX   EMTEALQG-------------GHKGIYDTYNNSLHFQLGWHLACLGVITSLVAQHMYAMPPYAFMARDYTTMSALYTHHQYIAGFLMIGAFAHGAIFLIRDYDPEANRDN 398

Consensus   VLDRXLXHXXAIISHLXWVXXFLGFHXXGLYVHNDXMXAFGXPXXXXXXXXIXXXPVFAQWXQXXHXXAXXGXXXXXXXPXXXAXXXXPXXGXXXXXGXXXAXXXXXXXX
PsaA_6KMW   LLDRVIRHRDAIISHLNWVCIFLGFHSFGLYVHNDTMRAFGRPQDMFSDTGIQLQPVFAQWVQNLHAAAAGG-----TAPNAAAGVS-PAFG-----GDILAVVGKVAMM 493
PsaA_6KMX   VLDRTLRHRDTVVSHLAWVCQFLGFHSFAMYCHNDTMRAFGRPQDMFSDTGIQLQPIFAQWVQQIQTMAVGGV----TAPGLGGPVS-HAFG-----GGVVAIGDKIAMM 489
PsaB_6KMW   VLDRVLQHKEAIISHLSWVSLFLGFHTLGLYVHNDVMVAFGTPEKQ-----ILVEPVFAQWIQAAHGKLLYGFDTLLSNPGSIASTAWPNYGNVWLSGWLDAINSGDNSL 515
PsaB_6KMX   VLARMLAHKEAIISHLSWVSLFLGFHTLGLYVHNDCEVALGSPEKQ-----ILIEPVFAQWTQAFHGKALYGINSLLSNPDSVASTAWPNYGNVWLSGWLEAVNNGANSL 503

Consensus   XXTXGXXDFLVHHXXAXXXHVTXLILXKGXLXARXSXLXPDKXXXGXXFPCDGPGRGGTCXXSAWDXXXLXXFWMXNXLXXVXFXXXWKXXXXVWXXXXXXGXVXXXXXX
PsaA_6KMW   PITLGTADFLVHHIHAFTIHVTVLILLKGVLFARNSRLIPDKGELGFRFPCDGPGRGGTCQVSGWDHVFLGLFWMYNSLSIVIFHFSWKMQSDVWGSVSPDGSVSHITAG 603
PsaA_6KMX   PIQLGTADFLIHHIHAFTIHVTVLVLLKGVLFSRNSRLIPDKGELGFRFPCDGPGRGGTCQVSAWDHVFLGLFWMYNSLSIVIFHFFWKMQSDVWGTVGADGTISHITGG 599
PsaB_6KMW   FLTIGPGDFLVHHAIALGLHTTTLILVKGALDARGSKLMPDKKDFGYSFPCDGPGRGGTCDISAWDAFYLAVFWMLNTIGWVTFYWHWK-HLAIW-----QGNVAQ---- 615
PsaB_6KMX   FLTIGPGDLLVHHAIALGLHVTTLILVKGALDARGSKLMPDKKDFGYSFPCDGPGRGGTCDISAWDAFYLATFWMLNTLGWVTFYWHWK-HLSVW-----SGNVAQ---- 603

Consensus   XFXXSSXXXXGWLRDXLWAXXSQXIXXYXXXXXXXLXXXXXXFLXGHXXWAXSXMFLXSXRGYWQELIEXXVWAHXXXXXX-XXXXXXXPXALSIXQXRXVGXAHFXXGX
PsaA_6KMW   NFAQSAITINGWLRDFLWAQASQVIGSYG----SALSAYGLLFLGAHFVWAFSLMFLFSGRGYWQELIESIVWAHNKLKVA----PAIQPRALSITQGRAVGVAHFLLGG 705
PsaA_6KMX   NFAQASITNNGWLRDFLWAQASQVIGSYG----SALSAYGLFFLAGHFIFGFSLMFLFSGRGYWQELIESIVWAHNKLKIT----TAIQPRALSITQGRAVGVAHYLLGG 701
PsaB_6KMW   -FNESSTYLMGWLRDYLWLNSSQLINGYNPYGMNNLAVWAWMFLLGHLVWATGFMFLISWRGYWQELIETLVWAHERTPLANLVRWKDKPVALSIVQARLVGLAHFSVGY 724
PsaB_6KMX   -FNESSTYLMGWFRDYLWANSAQLINGYSPAGTNSLAVWAWMFLFGHLAWAVSFMFLITWRGYWQELIETLMWAHENTPLS-FGYPKDKPVALSIVQARLVGLTHFTVGY 711
```

Supplementary Figure 6

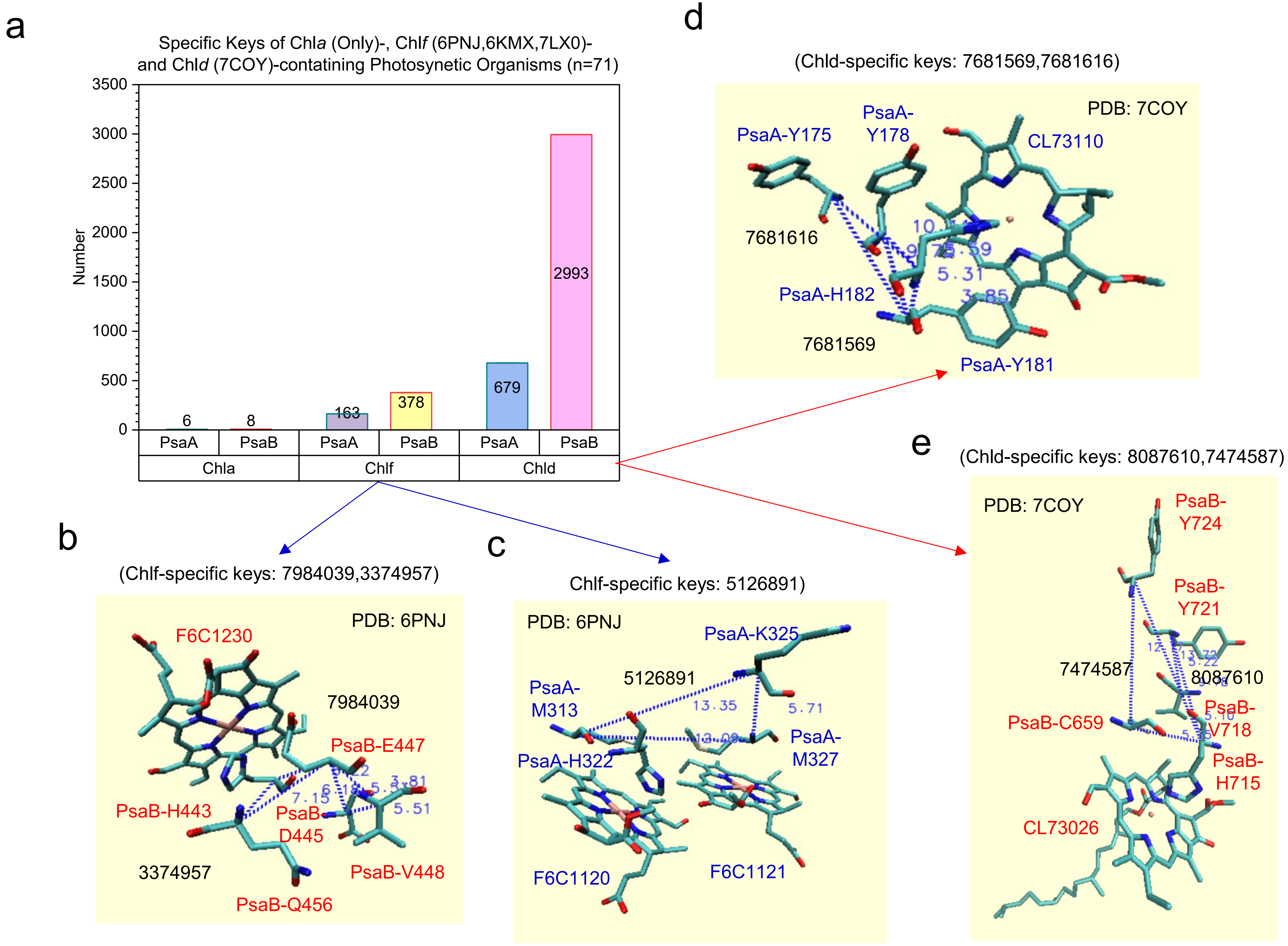

Supplementary Figure 7

CLA_6JO6ACA	65	2709	43680
CLA_6JO6ACB	65	2622	43680
CLA_6JO6P700B	65	2698	43680
CLA_6PNJA0A	60	2642	34220
CLA_6PNJA0B	65	2764	43680
CLA_6PNJAA	65	2772	43680

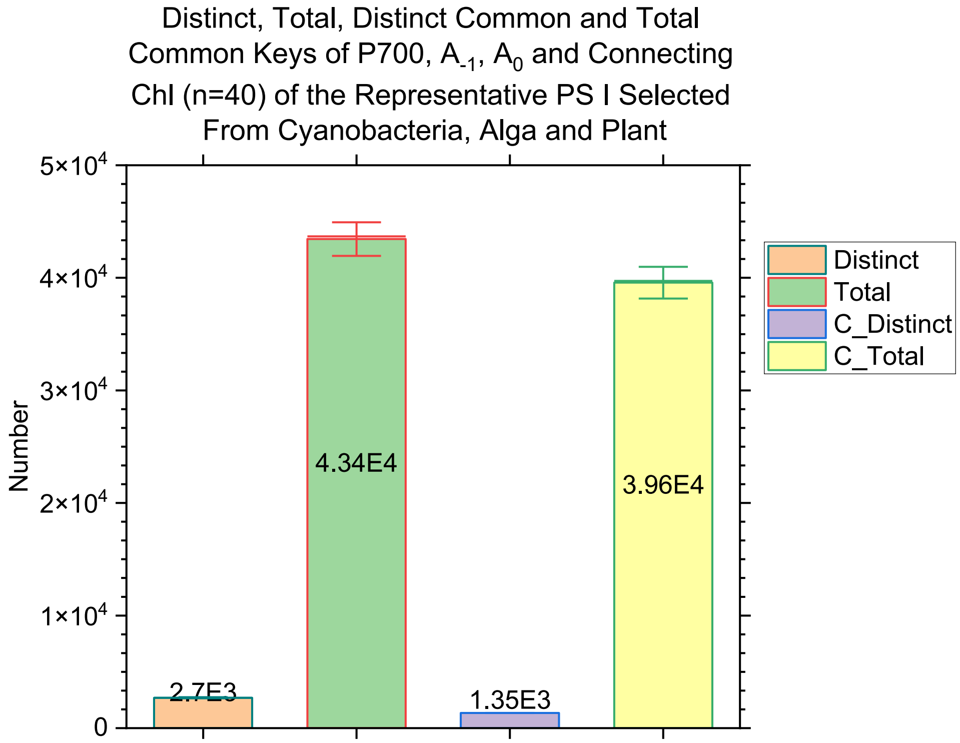

sample_et_mix5

Only 6PNJA0A is 60 atoms and the rest are 65 atoms

Supplementary Figure 8

a

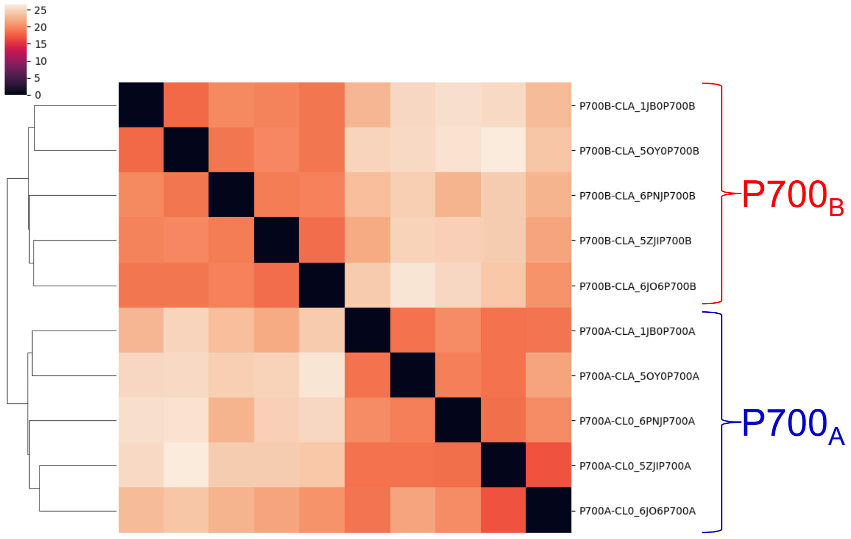

b

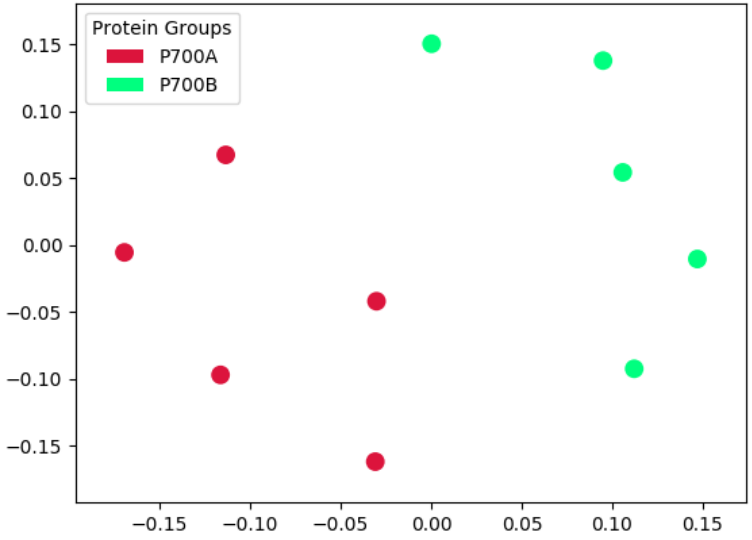

Supplementary Figure 9

a

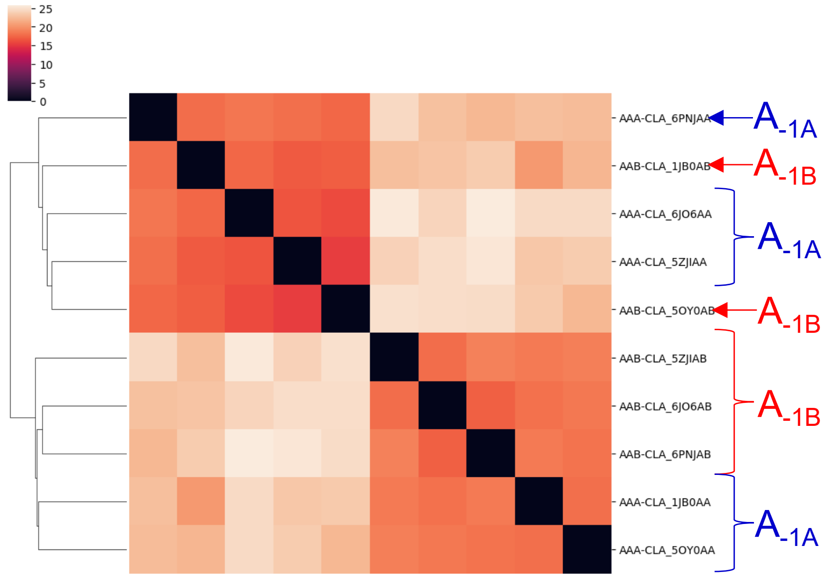

b

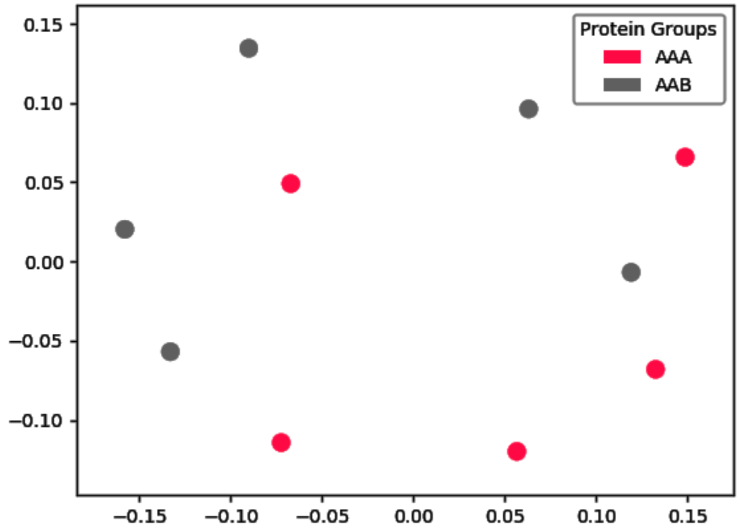

Supplementary Figure 10

a

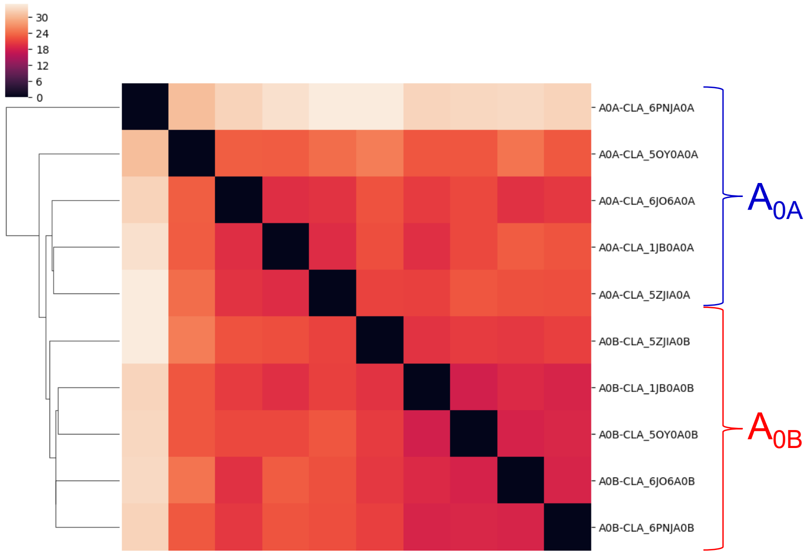

b

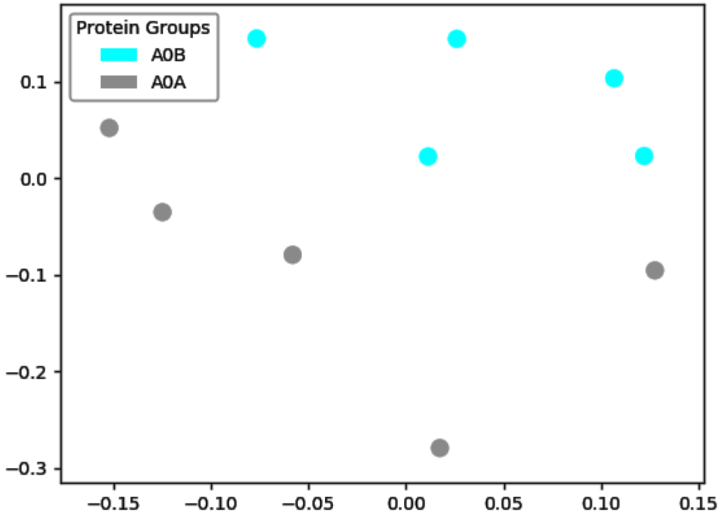

Supplementary Figure 11

a

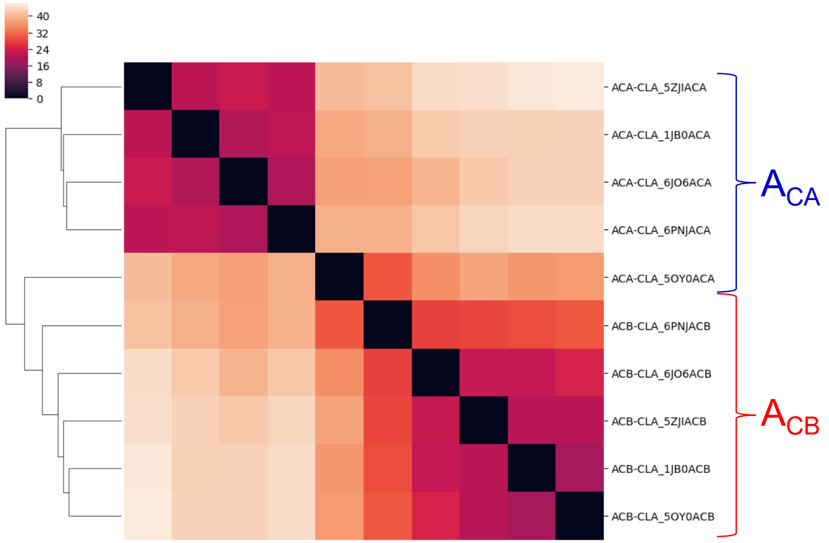

b

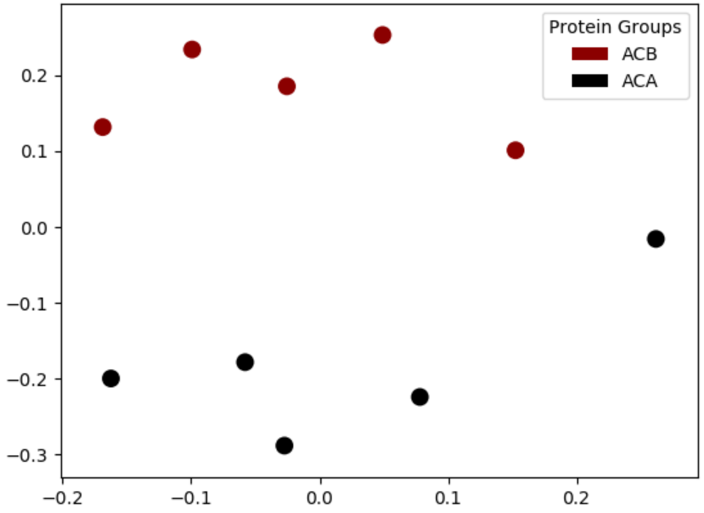

Supplementary Figure 12

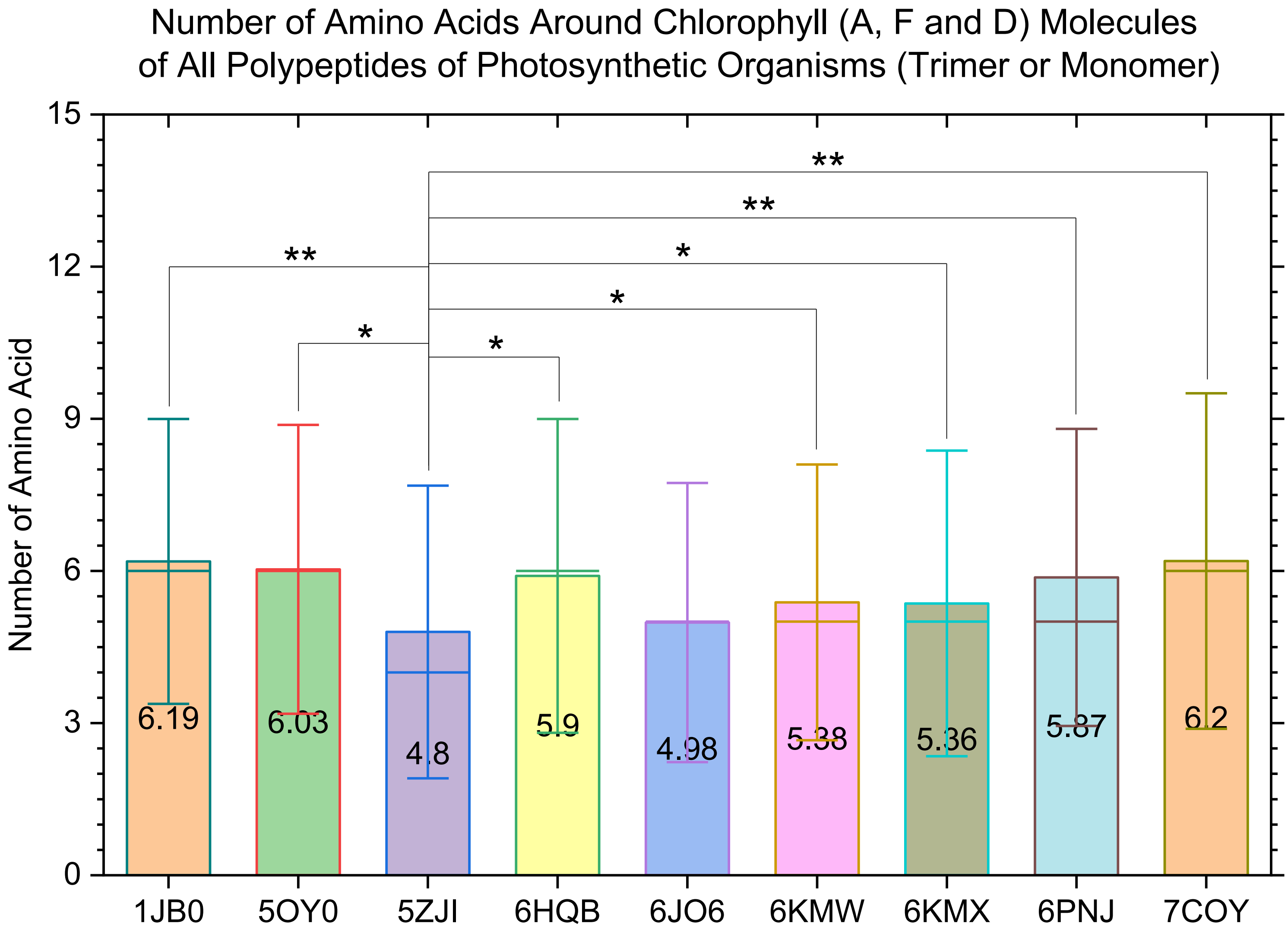

Supplementary Figure 13

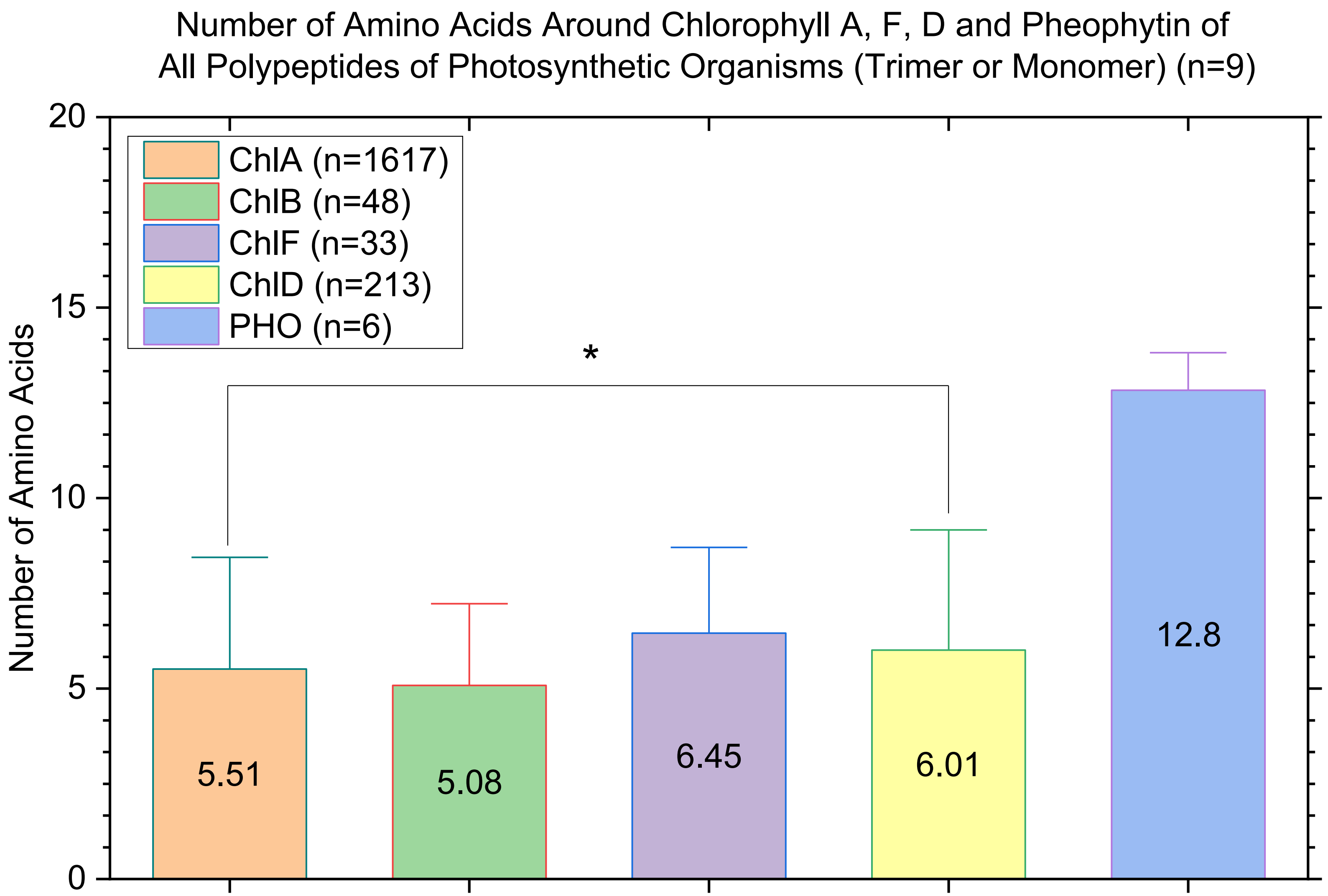

Supplementary Figure 14

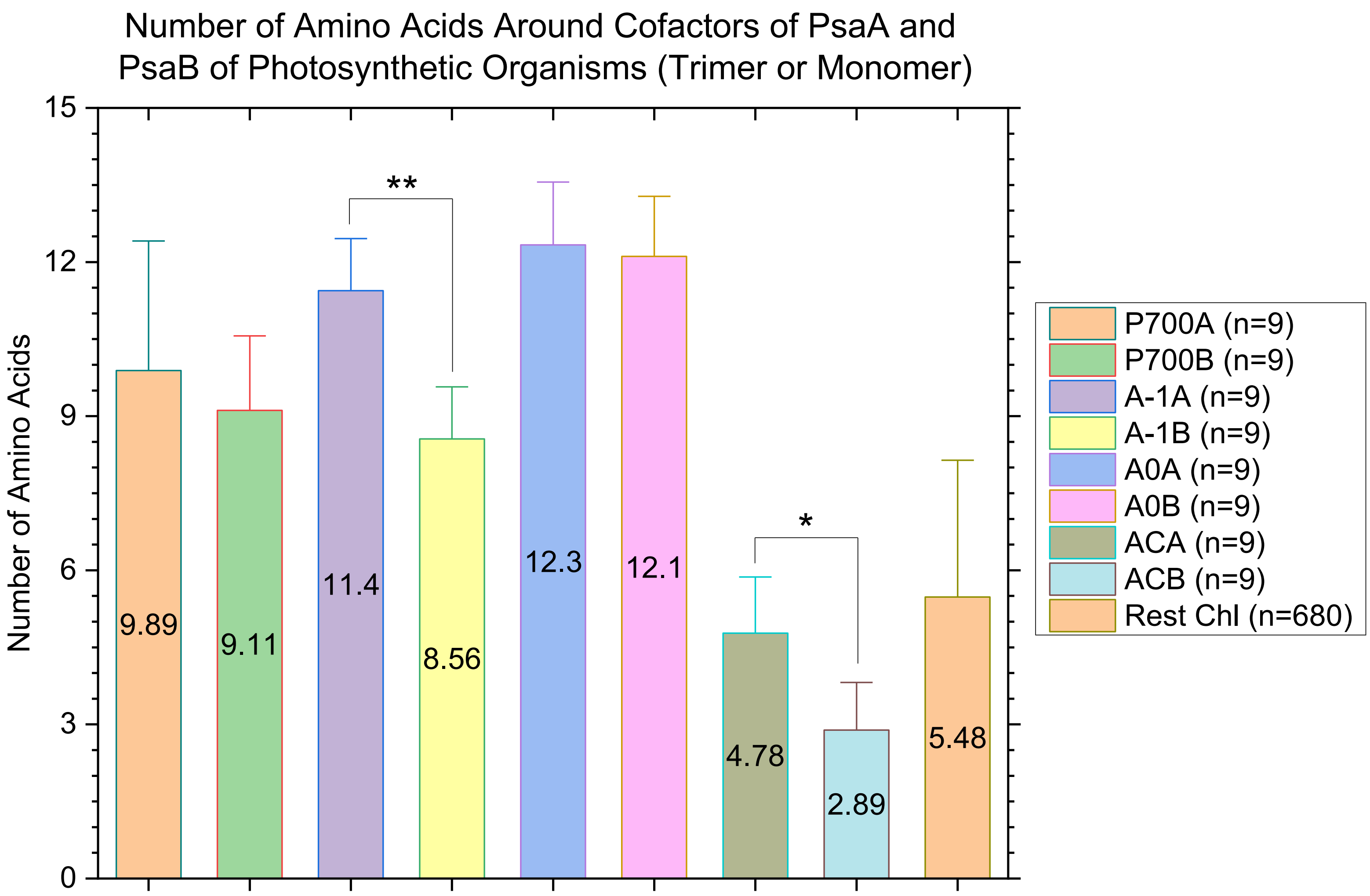

Supplementary Figure 15

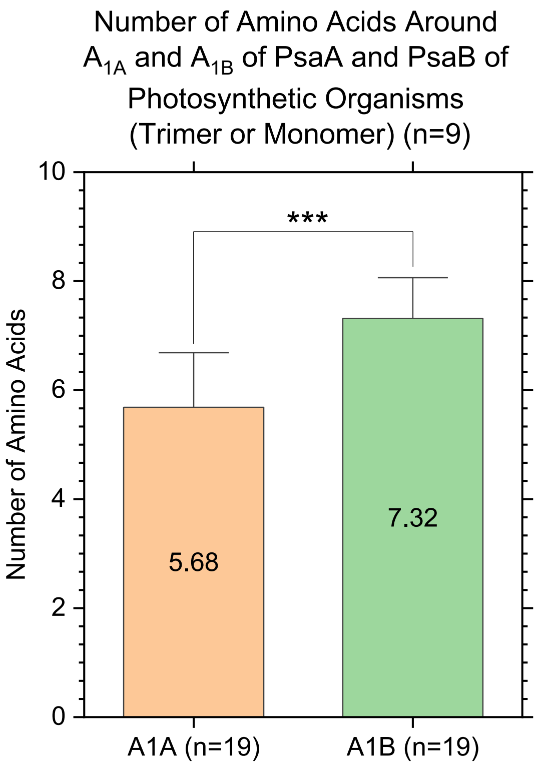

Supplementary Figure 16

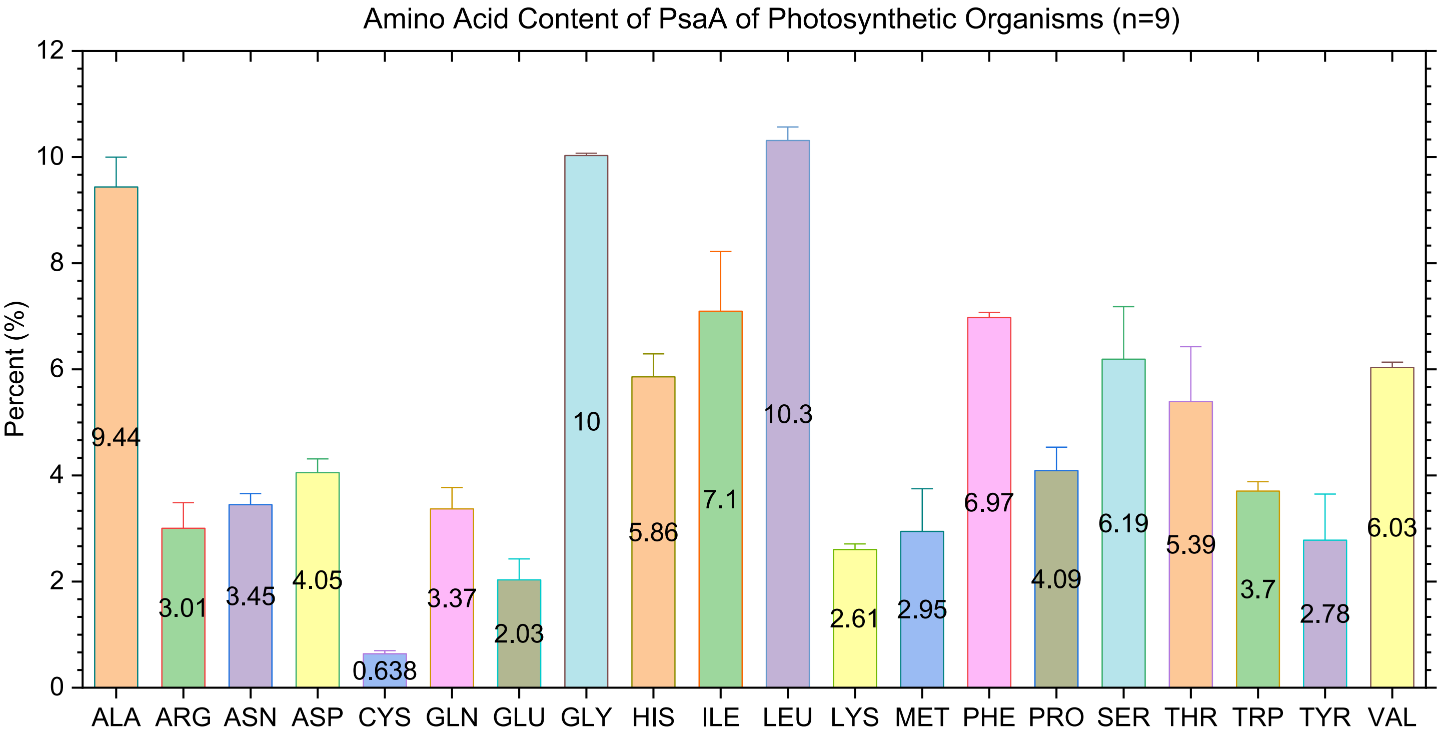

Supplementary Figure 17

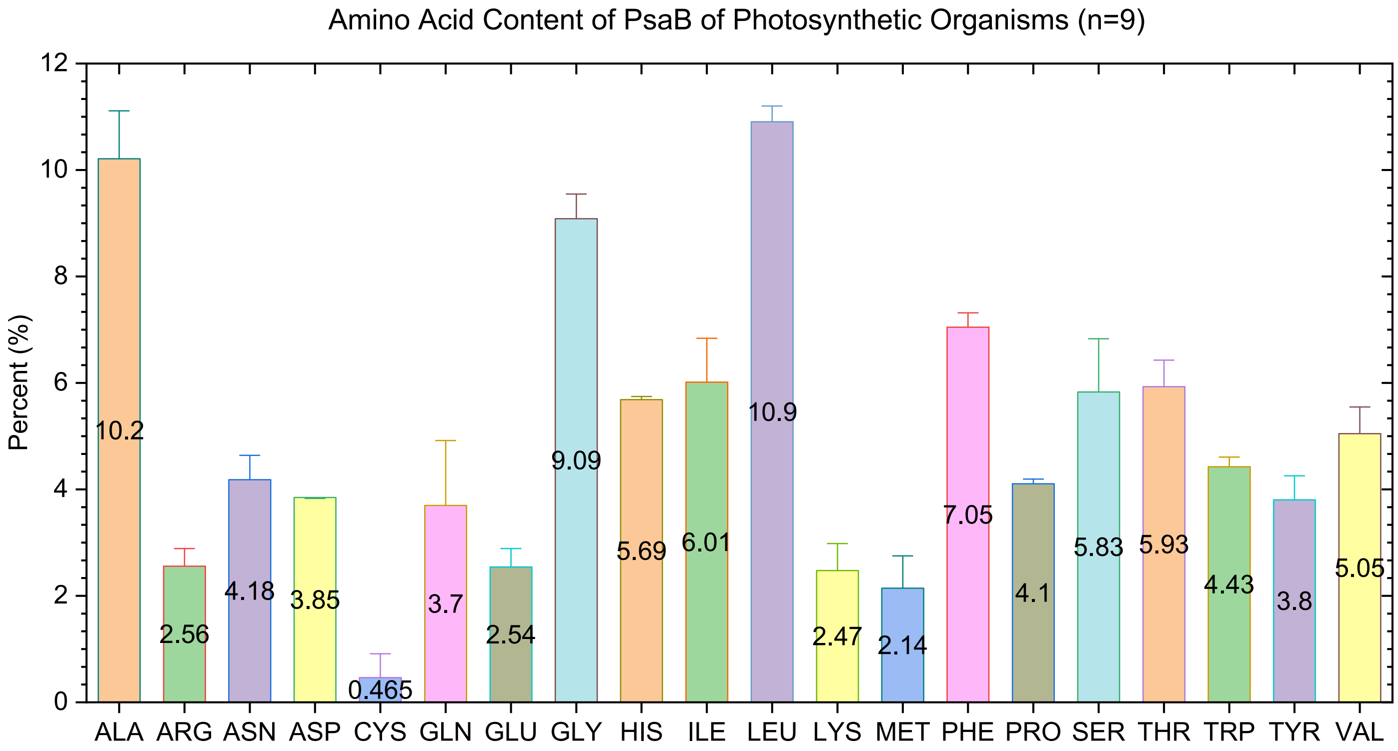

Supplementary Figure 18

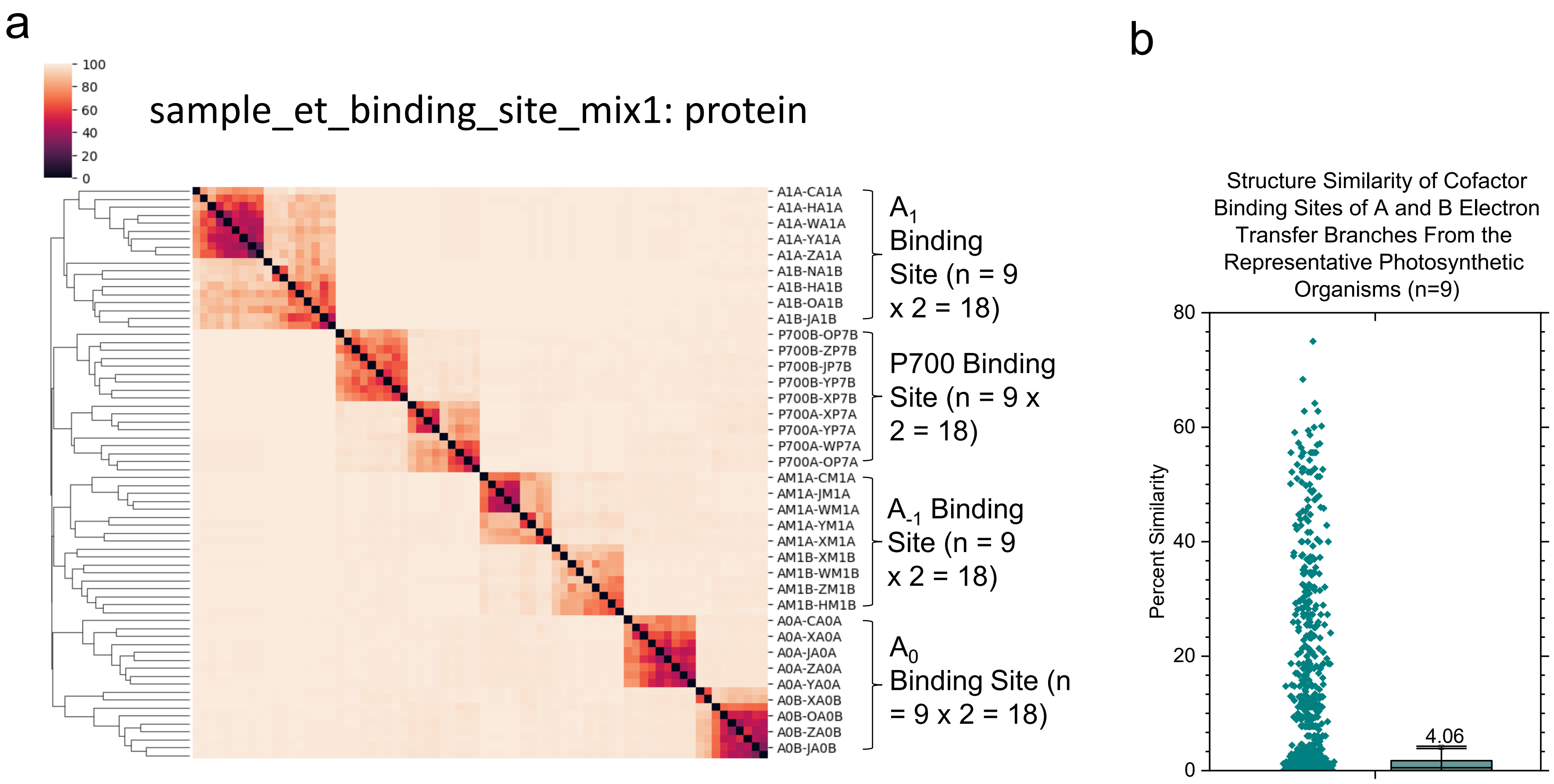

Supplementary Figure 19

a

Three $P700_A$-*specific* keys

7656395 TYR	599 TRP	622 PHE	607
7656400 TYR	599 TRP	622 PHE	672
6966143 TRP	622 PHE	607 PHE	672

b

Two $P700_B$-*specific* keys

7661403 TYR	714 TRP	616 HIS	651
7669487 TYR	718 TRP	616 THR	717

Supplementary Figure 20

Ten A_{-1A}-*specific* keys

key	aa0	pos0	aa1	pos1	aa2	pos2
7047321	TRP	579	LEU	673	PHE	578
9077323	ASN	582	LEU	673	PHE	578
3312094	HIS	676	PHE	578	PHE	677
7067594	TRP	579	HIS	676	PHE	677
9097595	ASN	582	HIS	676	PHE	677
7067593	TRP	579	HIS	676	PHE	578
9097597	ASN	582	HIS	676	PHE	578
6966094	TRP	579	PHE	677	PHE	578
8996098	ASN	582	PHE	677	PHE	578
9280261	ASN	582	TRP	586	PHE	578

Supplementary Figure 21

a

31 A_{0A}-*specific* keys

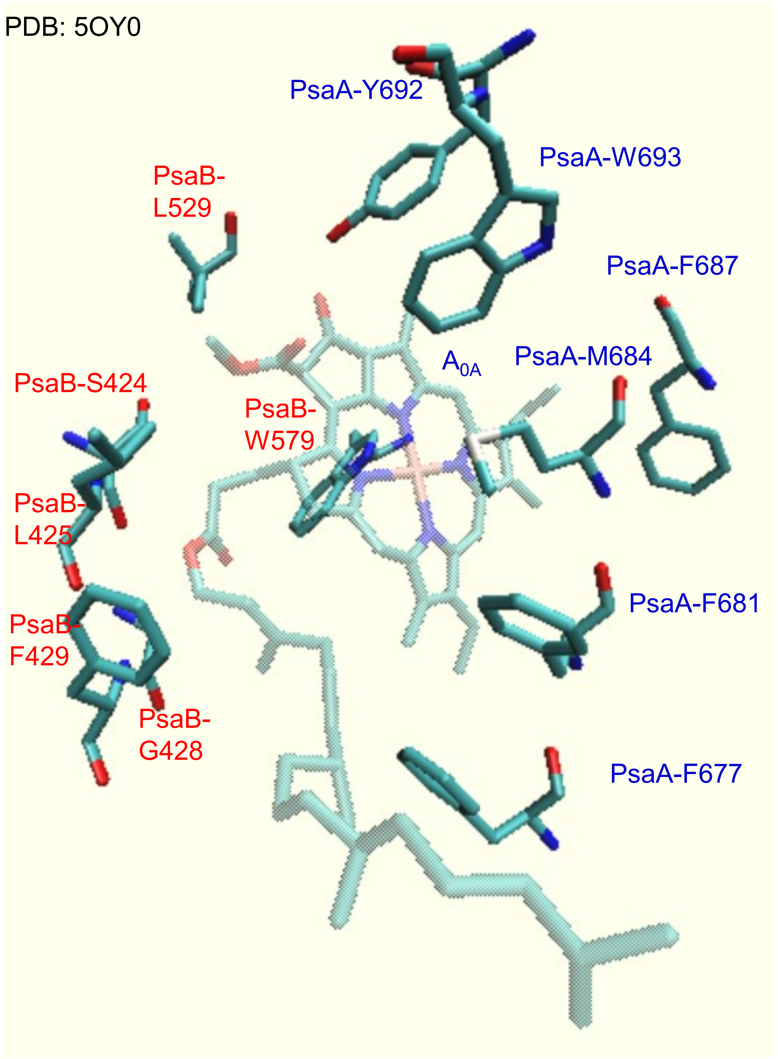

b

key	aa0	pos0	aa1	pos1	aa2	pos2
7372149	TYR	692	PHE	677	PHE	681
6154144	SER	424	PHE	687	PHE	677
2906174	LEU	529	PHE	677	PHE	687
7209742	TRP	693	SER	424	PHE	677
6966116	TRP	579	PHE	429	PHE	677
6336798	SER	424	MET	684	PHE	681
7372116	TYR	692	PHE	681	PHE	687
4124142	GLY	428	PHE	687	PHE	681
7514242	TYR	692	GLY	428	PHE	681
6235342	SER	424	LEU	696	PHE	681
6336821	SER	424	MET	684	PHE	687
4936148	MET	684	PHE	429	PHE	687
7148772	TRP	579	MET	684	PHE	687
7624848	TYR	692	SER	424	MET	684
5082273	MET	684	GLY	428	LEU	696
5021370	MET	684	LEU	529	LEU	696
5017297	MET	684	LEU	425	PHE	429
5017318	MET	684	LEU	529	PHE	429
7148818	TRP	579	MET	684	PHE	429
7250318	TRP	693	TRP	579	PHE	687
6235342	SER	424	LEU	696	PHE	687
4205342	GLY	428	LEU	696	PHE	687
6154156	SER	424	PHE	687	PHE	429
4124149	GLY	428	PHE	687	PHE	429
4205342	GLY	428	LEU	529	PHE	687
7518287	TYR	692	GLY	428	LEU	696
7622828	TYR	692	SER	424	GLY	428
7663441	TYR	692	TRP	579	GLY	428
7047344	TRP	693	LEU	696	PHE	429
7262493	TRP	693	TRP	579	SER	424
7112276	TRP	693	GLY	428	LEU	529
7047344	TRP	579	LEU	696	PHE	429
7213744	TRP	579	SER	424	LEU	529
4205291	GLY	428	LEU	529	PHE	429

Supplementary Figure 22

a

6 A_{0B}-*specific* keys

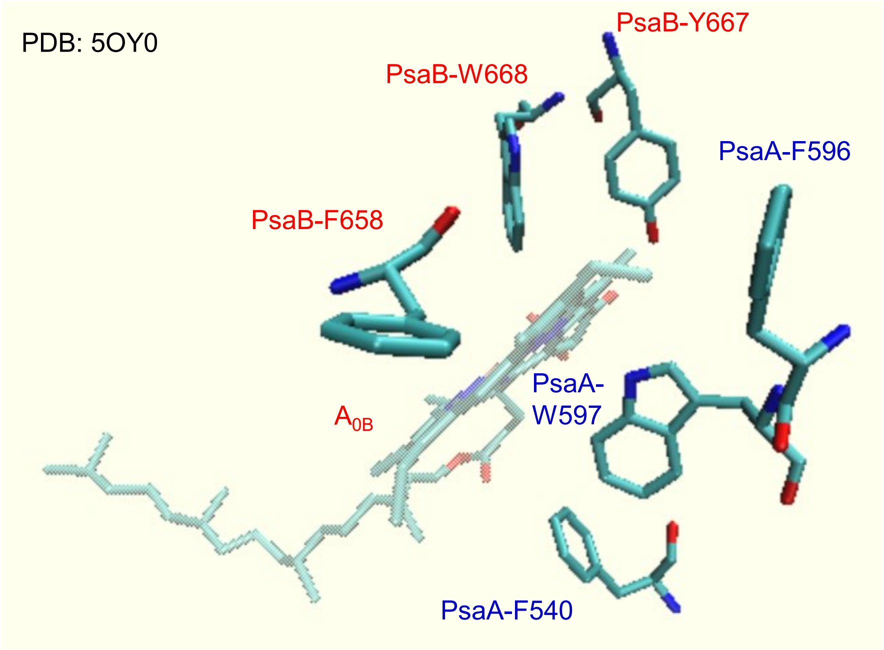

b

key	aa0	pos0	aa1	pos1	aa2	pos2
6966090	TRP	597	PHE	540	PHE	596
1282142	PHE	540	PHE	658	PHE	596
7372147	TYR	667	PHE	540	PHE	596
7372142	TYR	667	PHE	540	PHE	658
7250294	TRP	668	TRP	597	PHE	596
7372113	TYR	667	PHE	658	PHE	596

Supplementary Figure 23

3 A_{1A}-*specific* key

key	aa0	pos0	aa1	pos1	aa2	pos2
7148795	TRP	664	MET	684	PHE	685
8477479	ALA	717	TRP	664	MET	684
7250342	TRP	664	TRP	693	PHE	685

Supplementary Figure 24

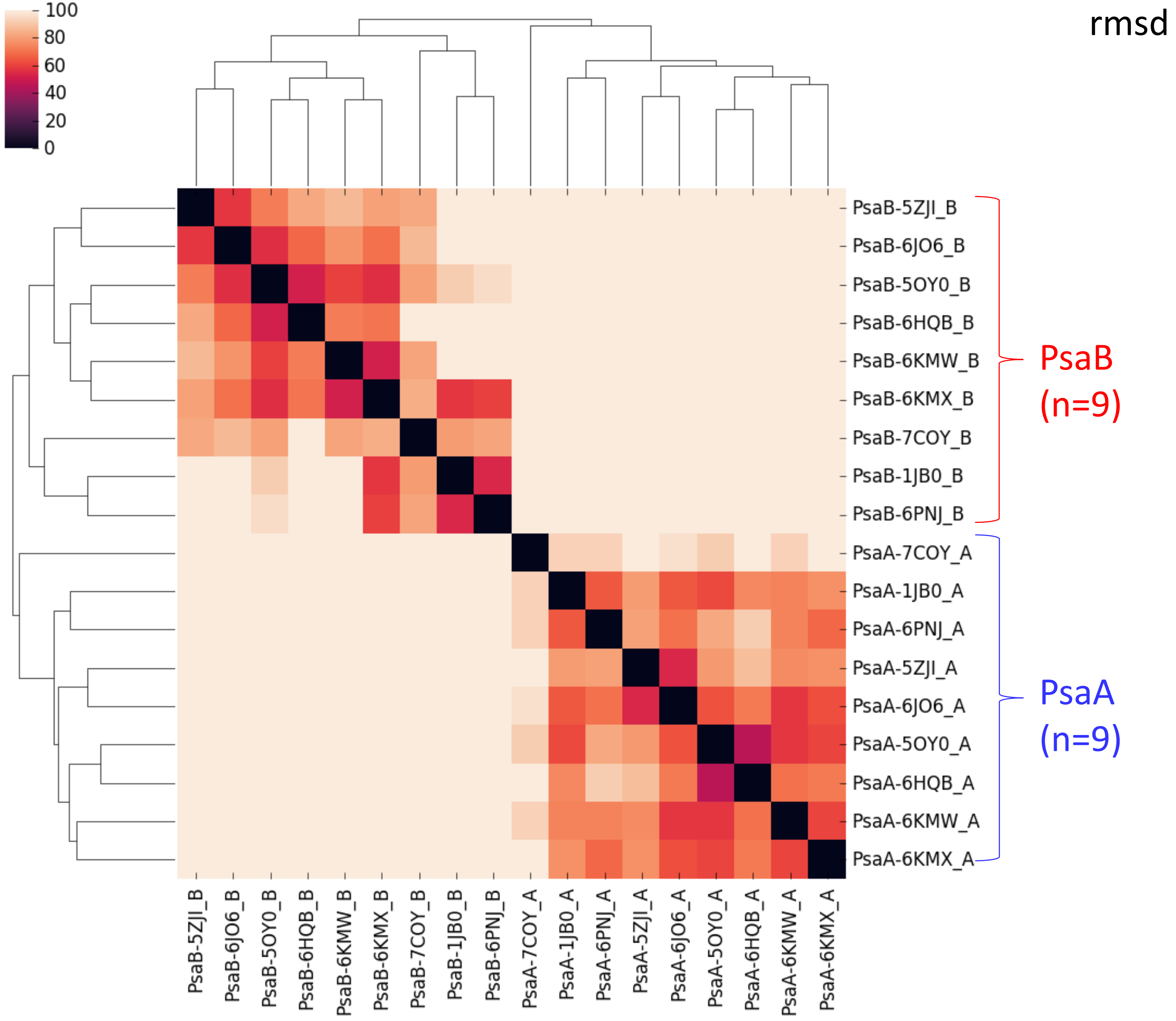

Supplementary Figure 25

ClustalW

Neighbor-Joining

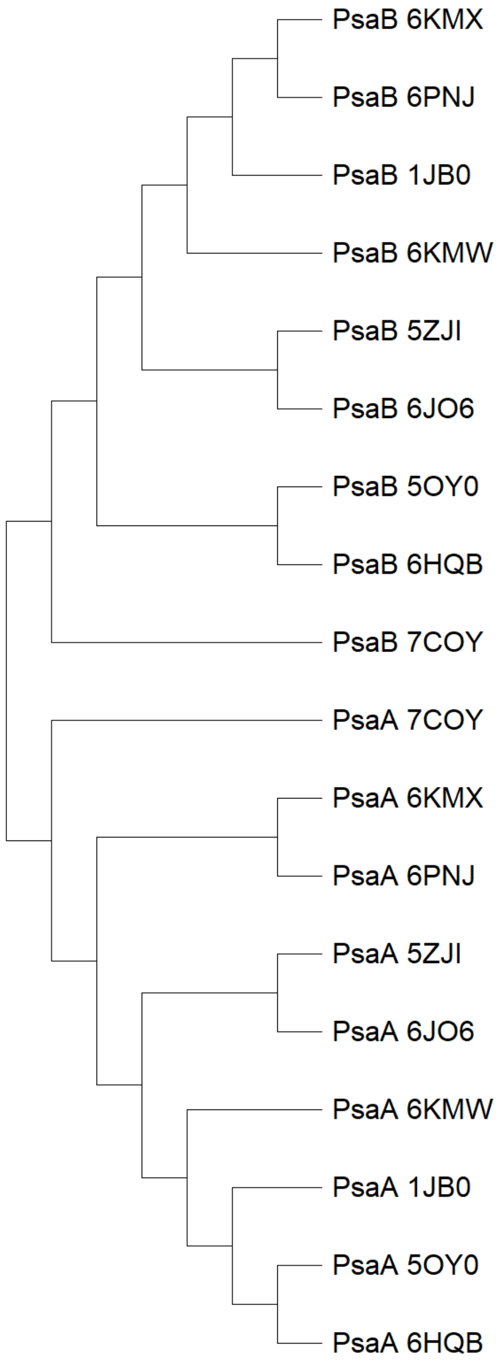

Supplementary Figure 26

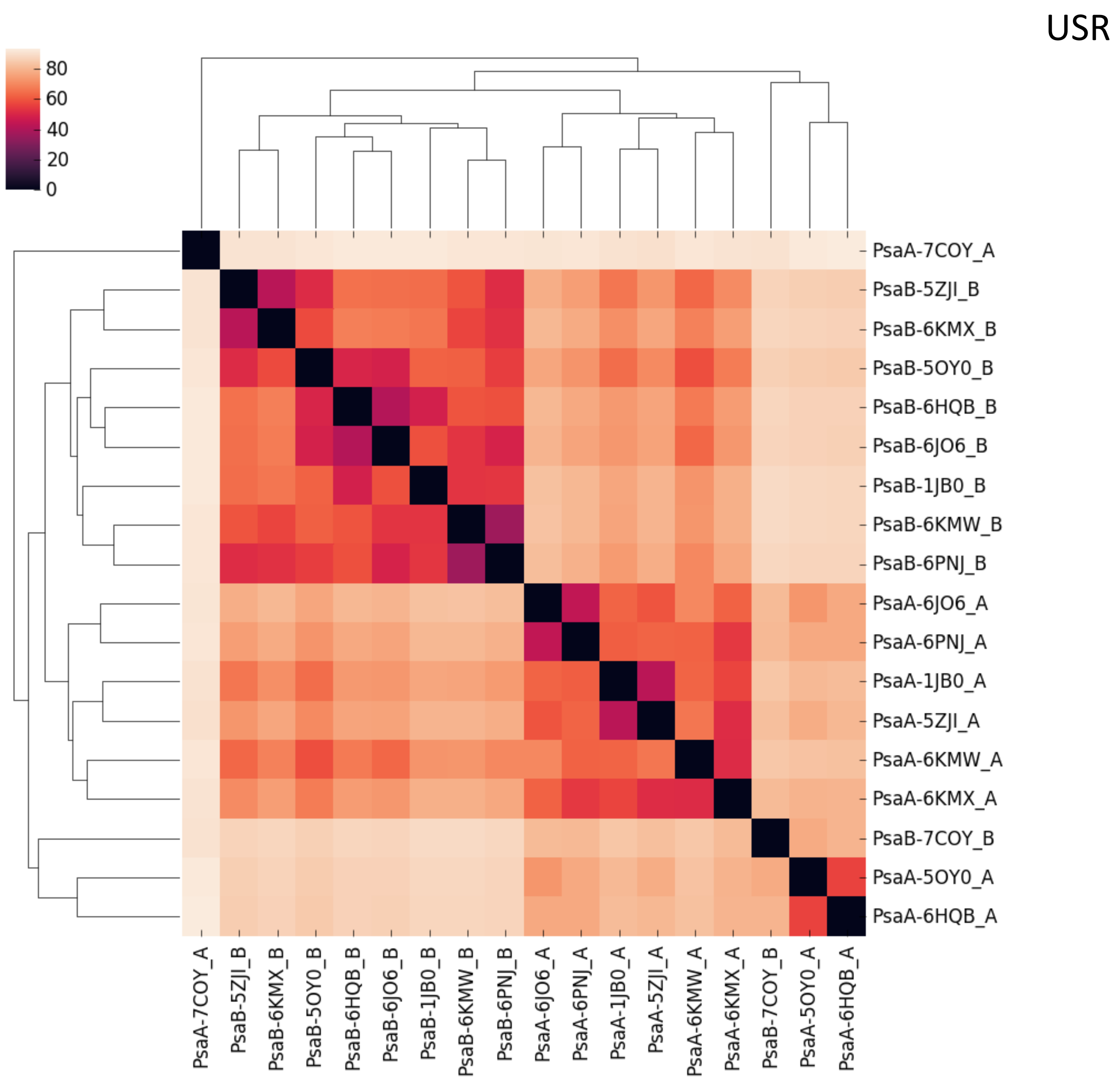

Supplementary Figure 27

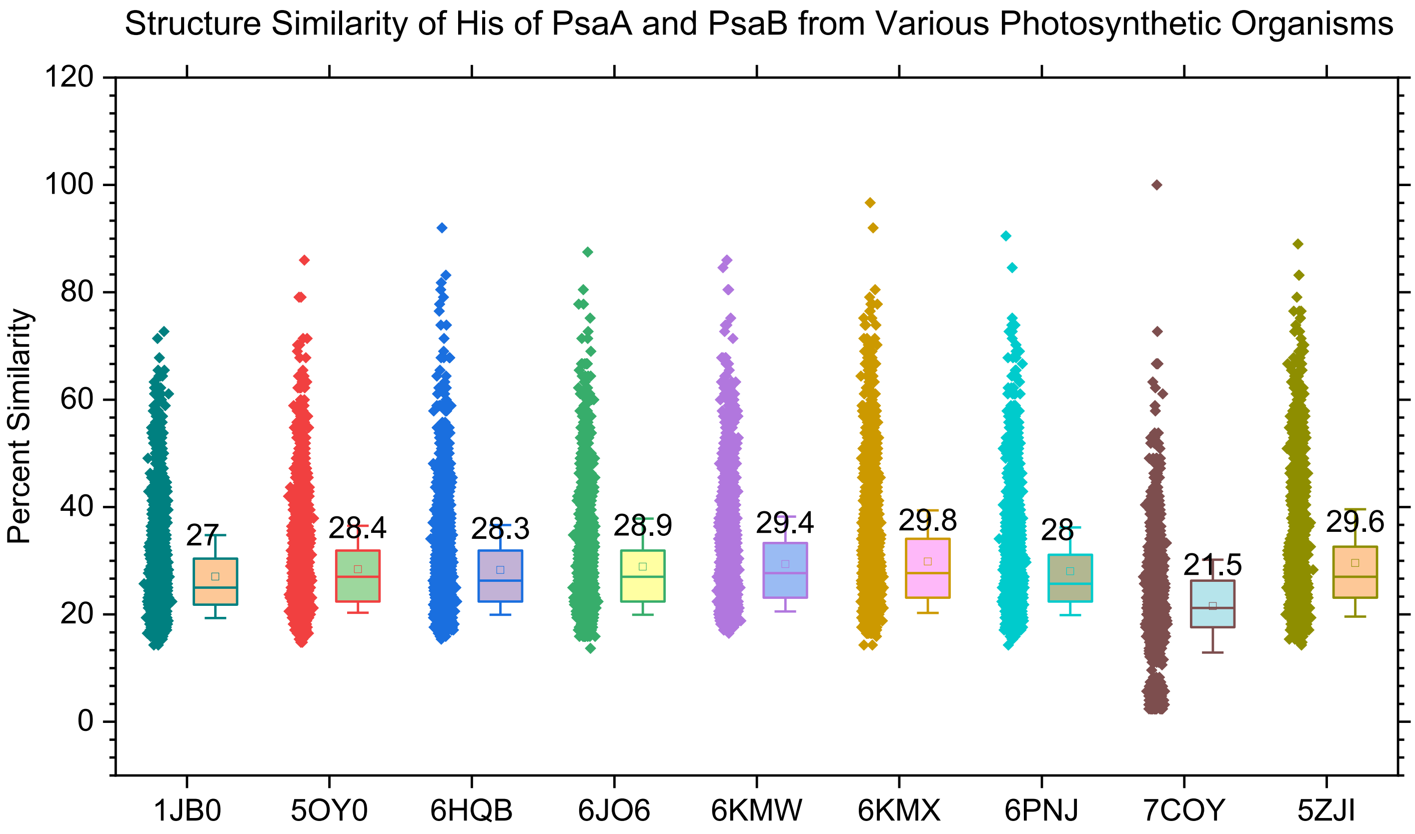

Supplementary Figure 28

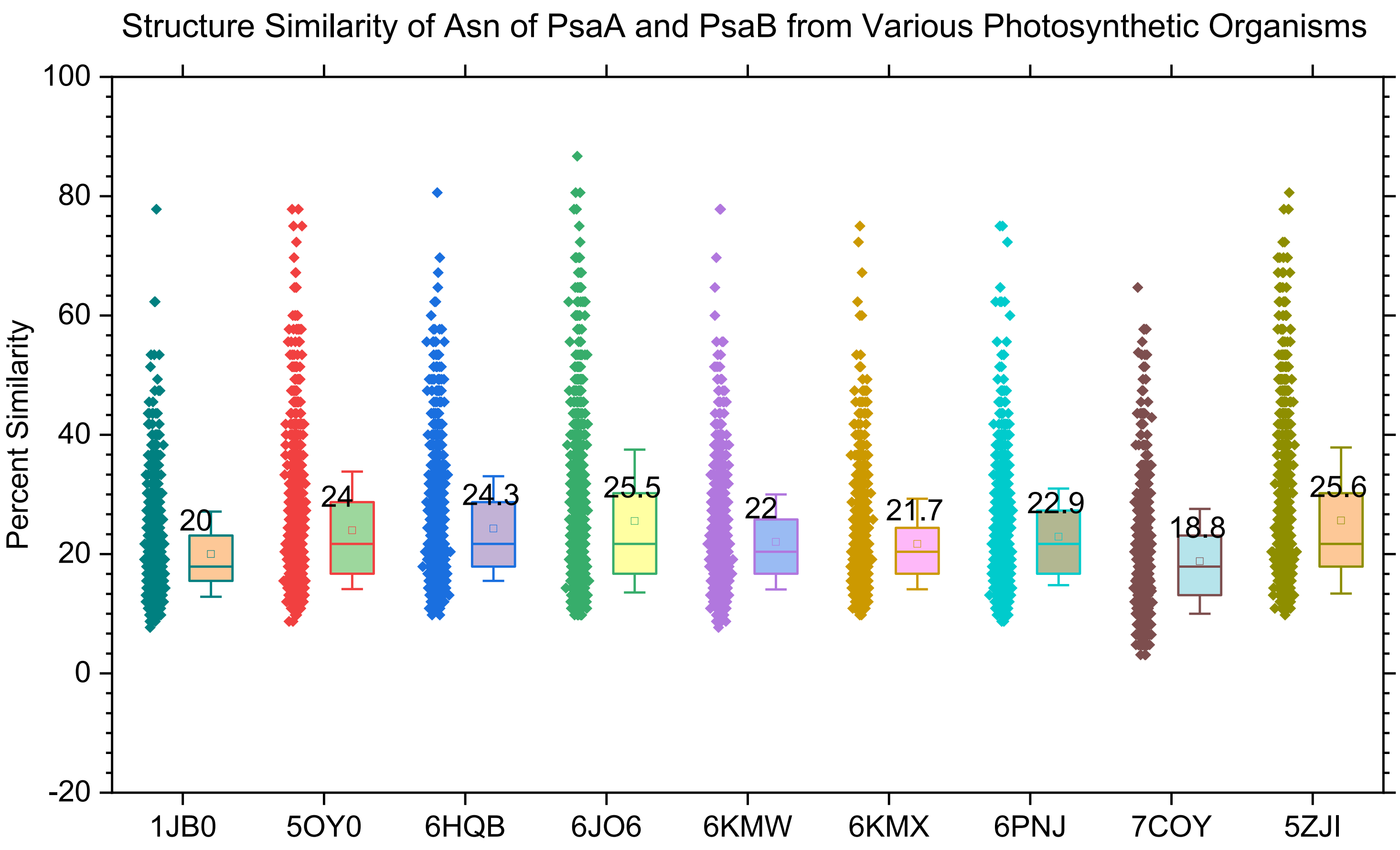

Supplementary Figure 29

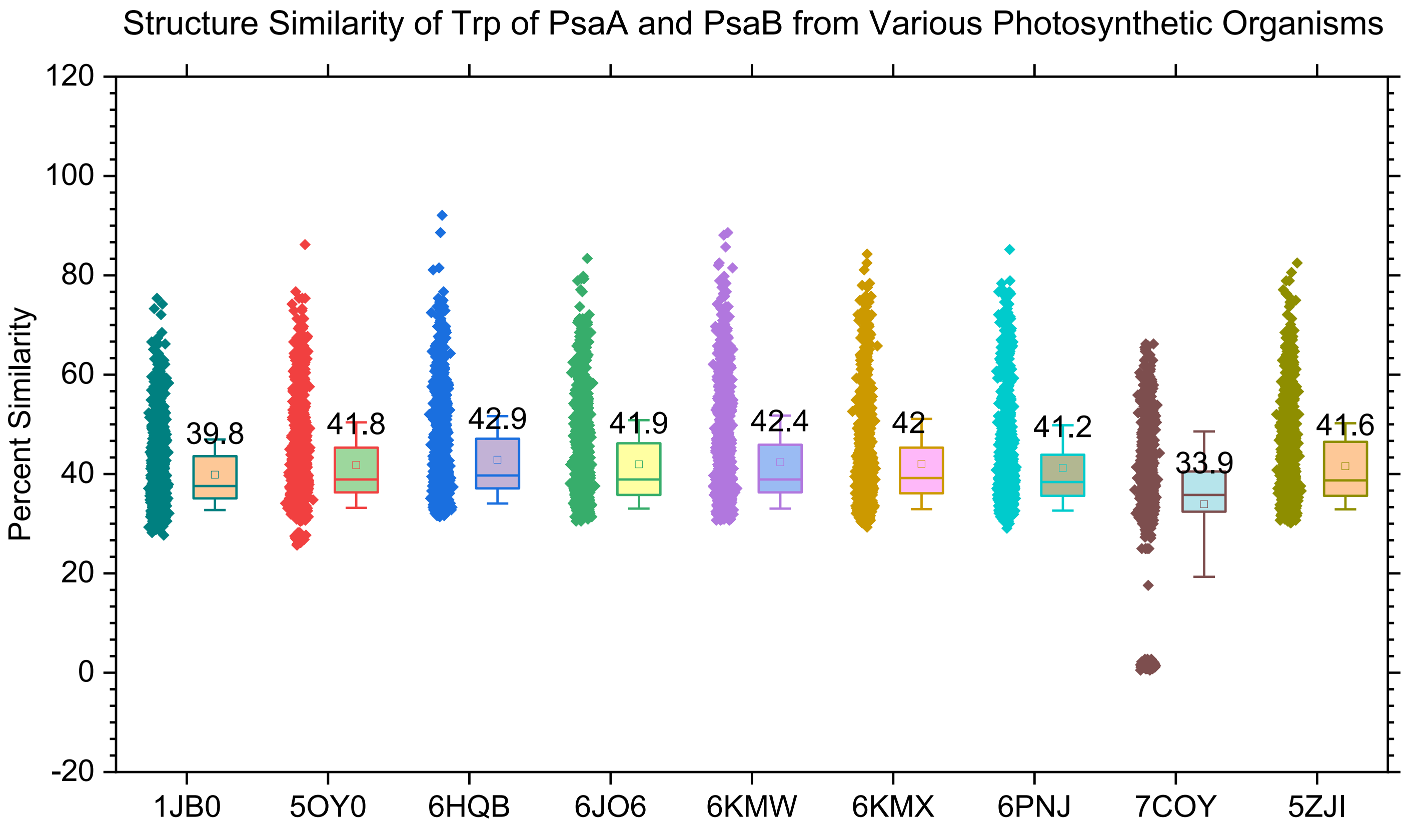

Supplementary Figure 30

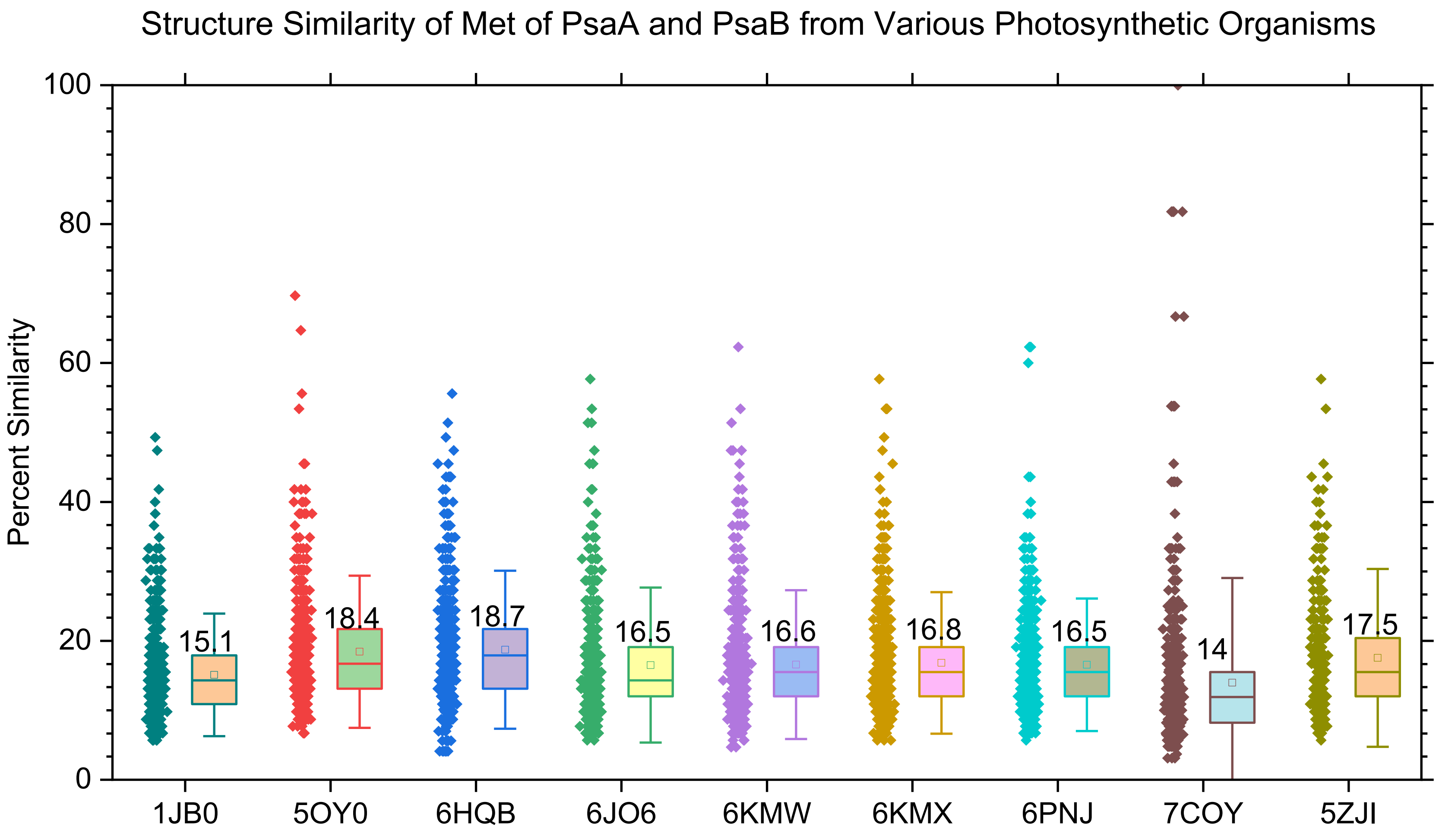

Supplementary Figure 31

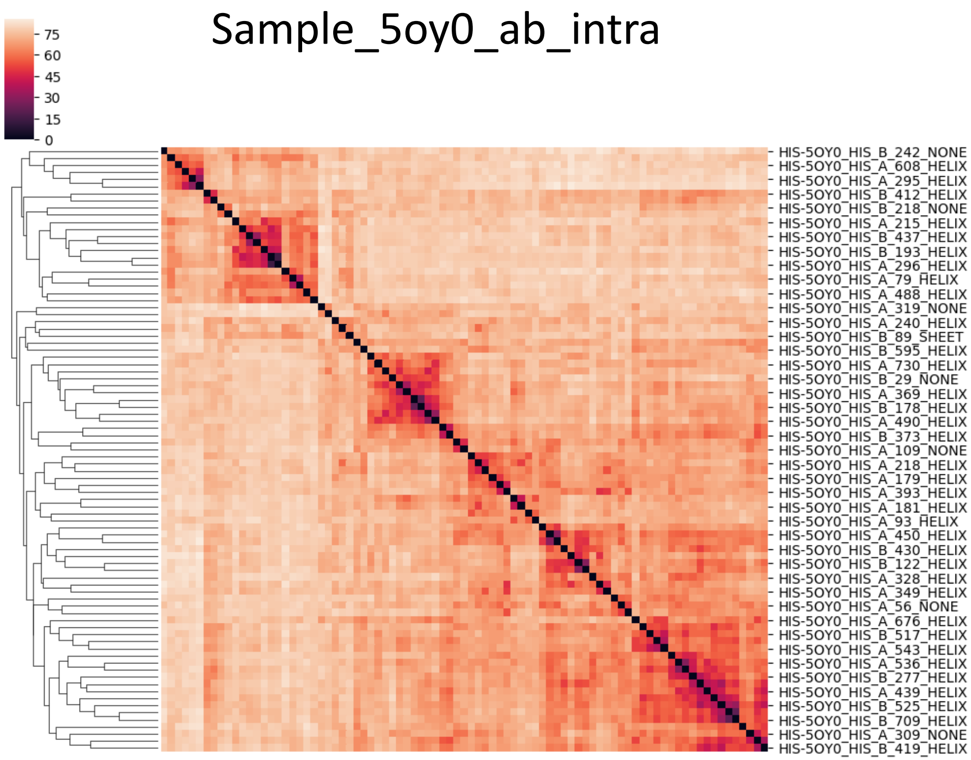

HIS-5OY0_HIS_A_538_HELIX
HIS-5OY0_HIS_A_676_HELIX
HIS-5OY0_HIS_B_308_NONE
HIS-5OY0_HIS_B_525_HELIX
HIS-5OY0_HIS_B_651_HELIX
HIS-5OY0_HIS_B_709_HELIX

Supplementary Figure 32

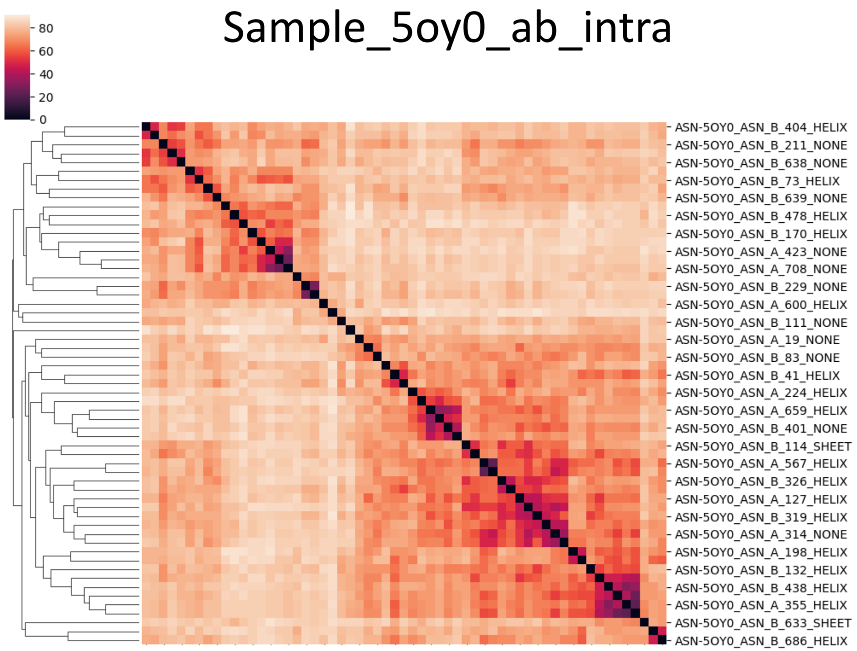

ASN-5OY0_ASN_A_708_NONE
ASN-5OY0_ASN_B_582_HELIX
ASN-5OY0_ASN_B_229_NONE
ASN-5OY0_ASN_A_282_NONE
ASN-5OY0_ASN_A_600_HELIX
ASN-5OY0_ASN_A_441_HELIX

Supplementary Figure 33

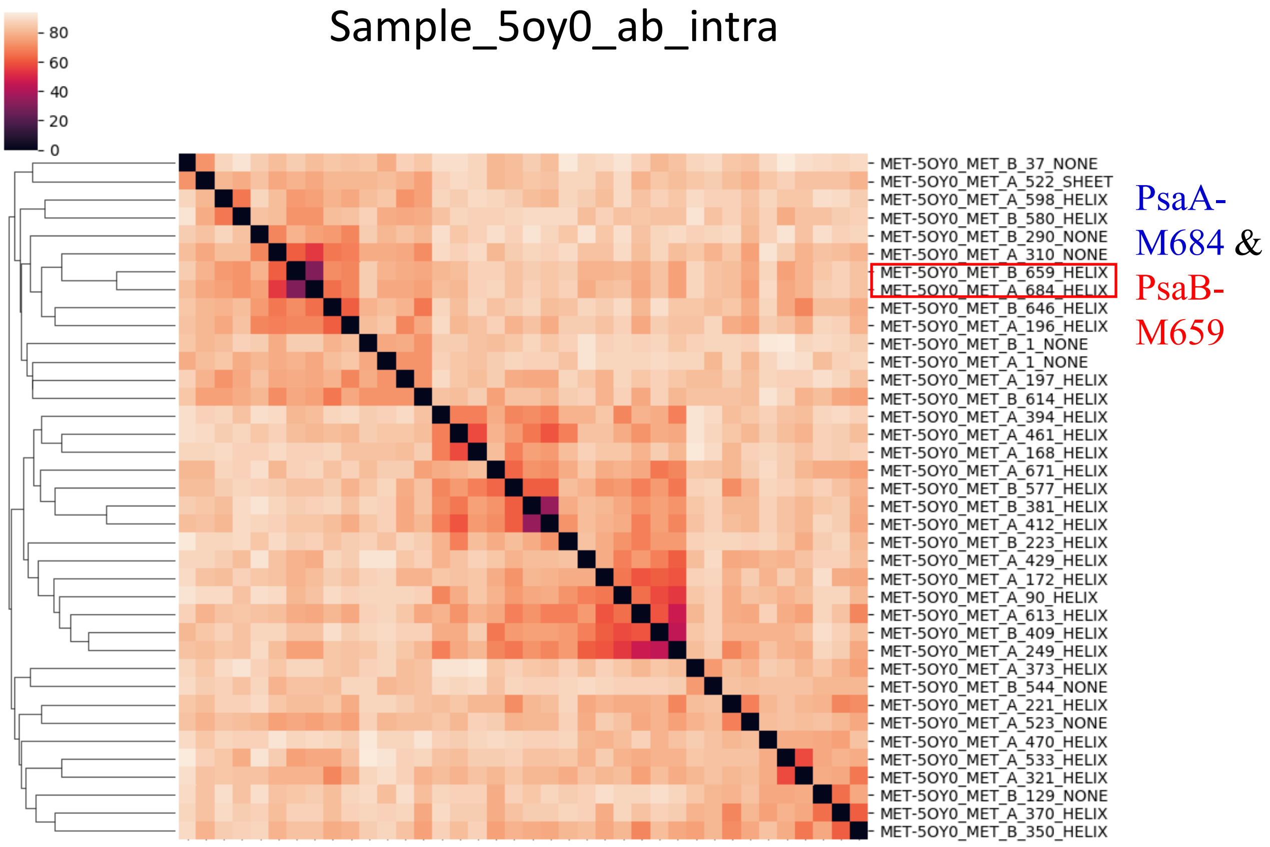

Supplementary Figure 34

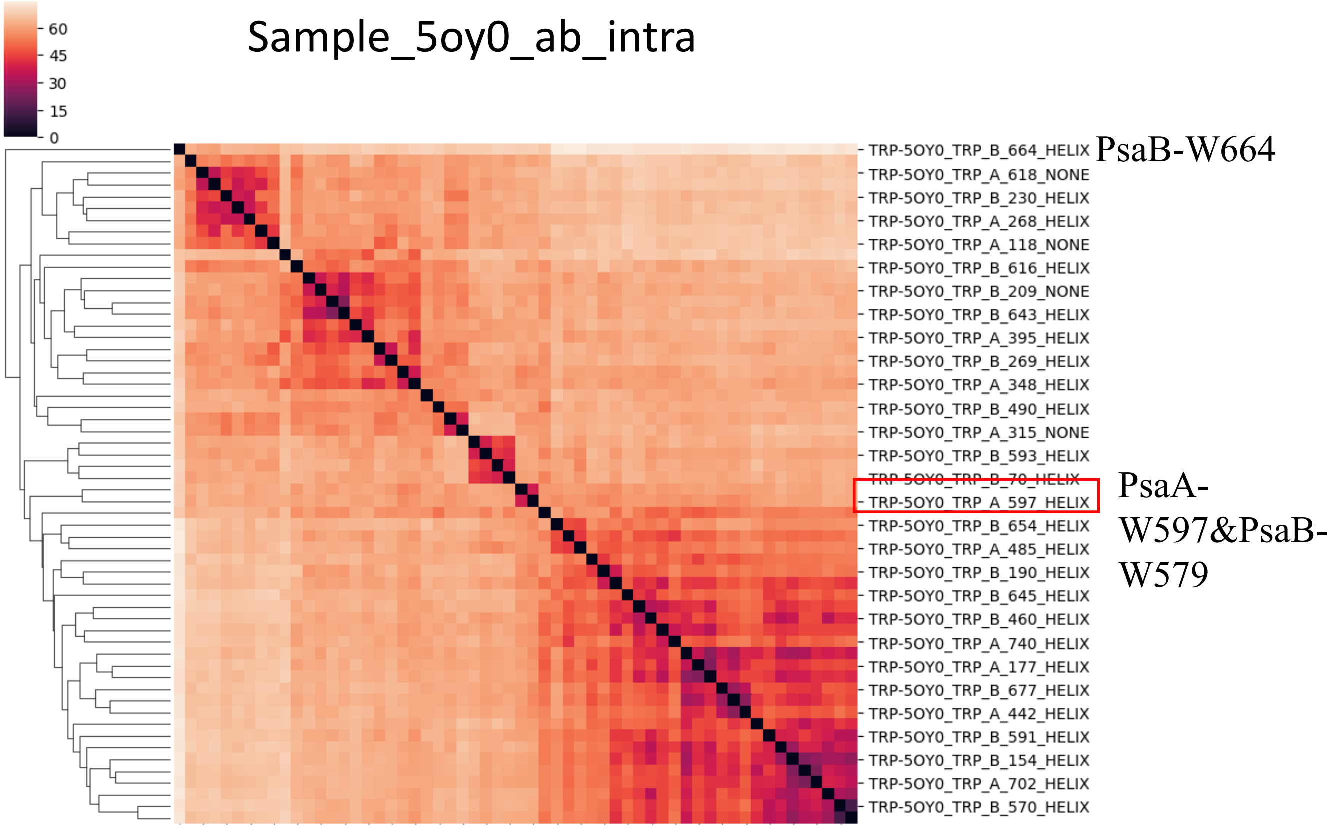

TRP-5OY0_TRP_B_230_HELIX
TRP-5OY0_TRP_B_586_HELIX
TRP-5OY0_TRP_A_268_HELIX

TRP-5OY0_TRP_A_118_NONE
TRP-5OY0_TRP_B_668_HELIX
TRP-5OY0_TRP_B_616_HELIX
TRP-5OY0_TRP_A_49_HELIX
TRP-5OY0_TRP_B_209_NONE
TRP-5OY0_TRP_A_693_HELIX
TRP-5OY0_TRP_B_643_HELIX

Supplementary Figure 35

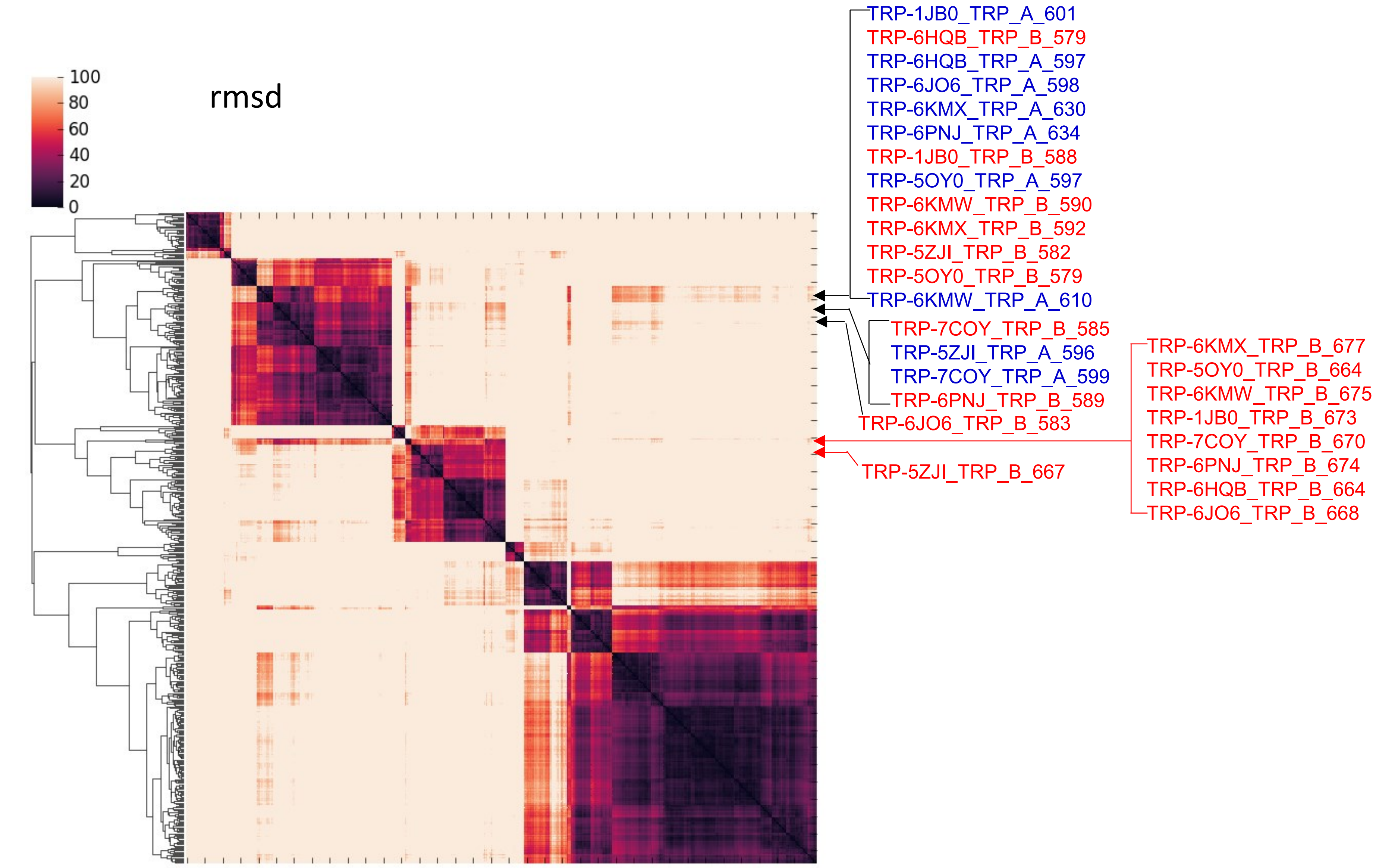

Supplementary Figure 36

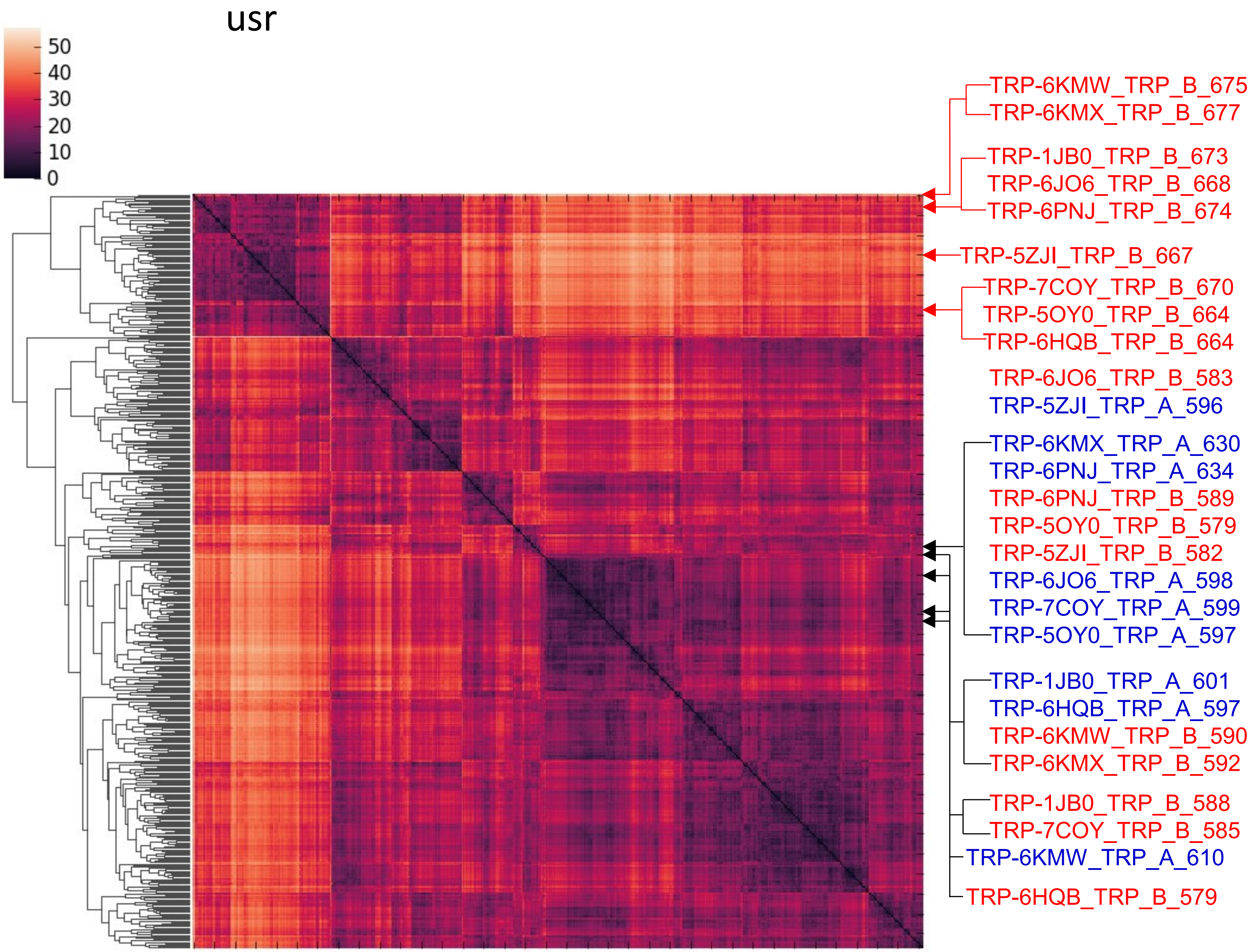

Supplementary Figure 37

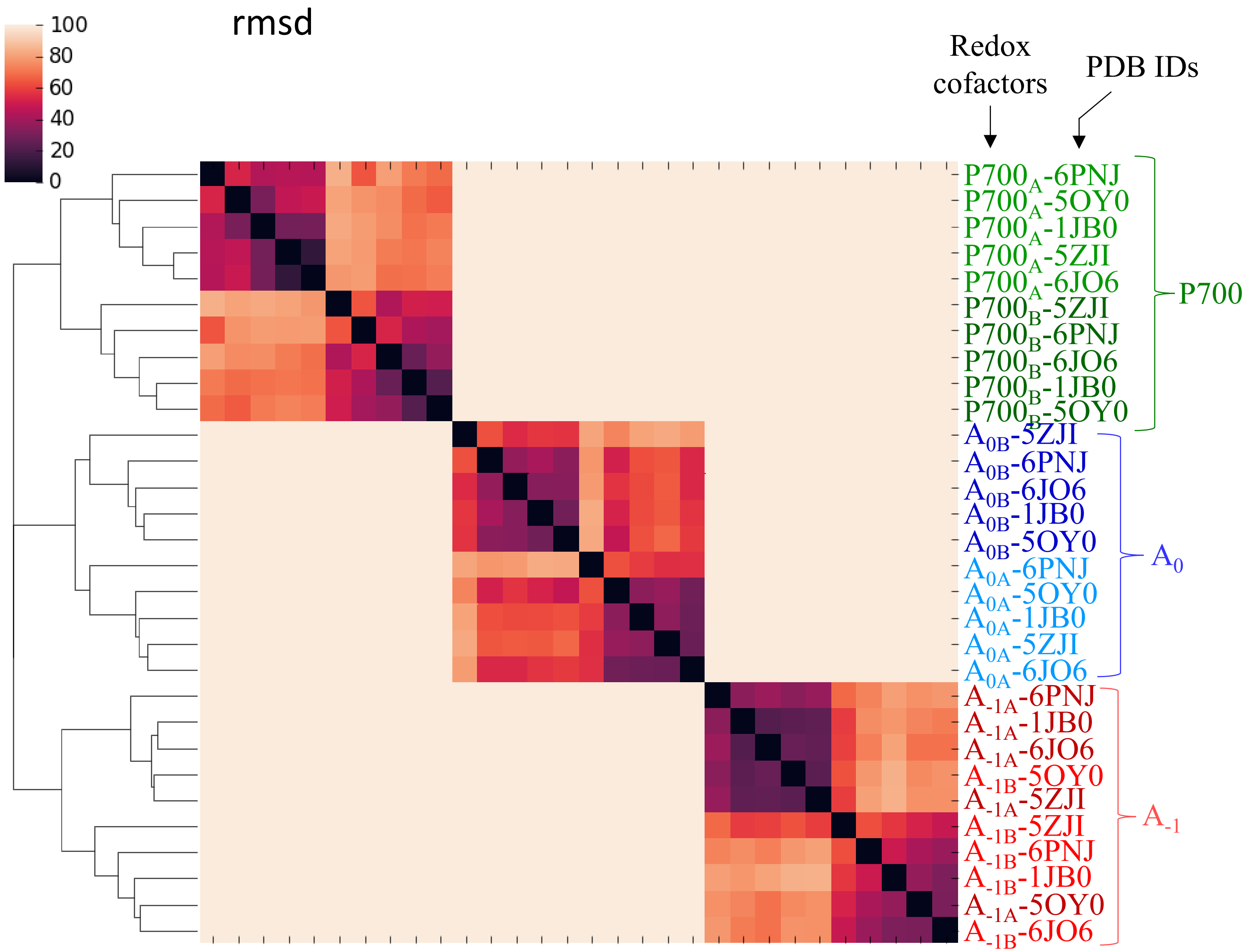

Supplementary Figure 38

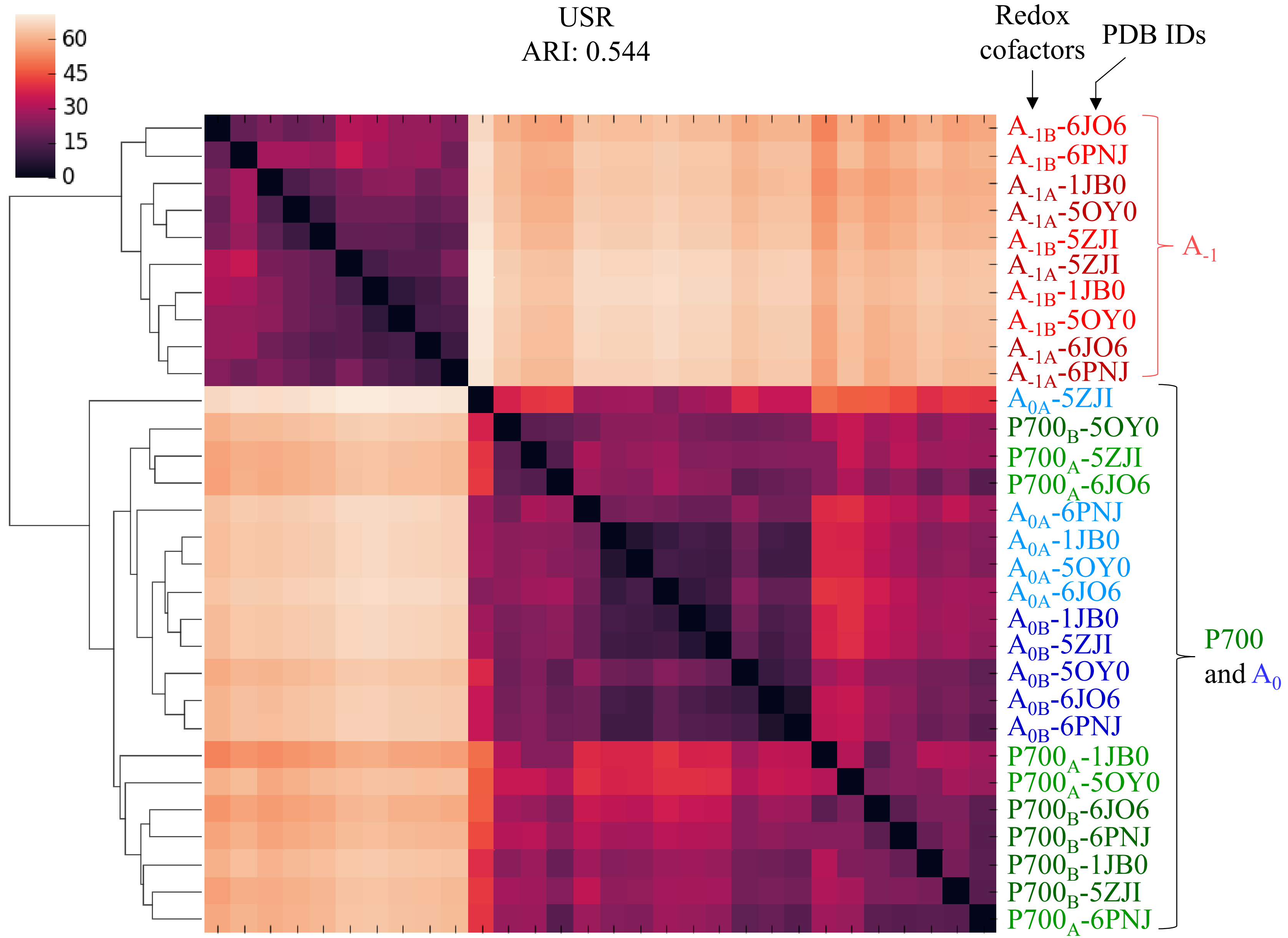

Supplementary Figure 32

a

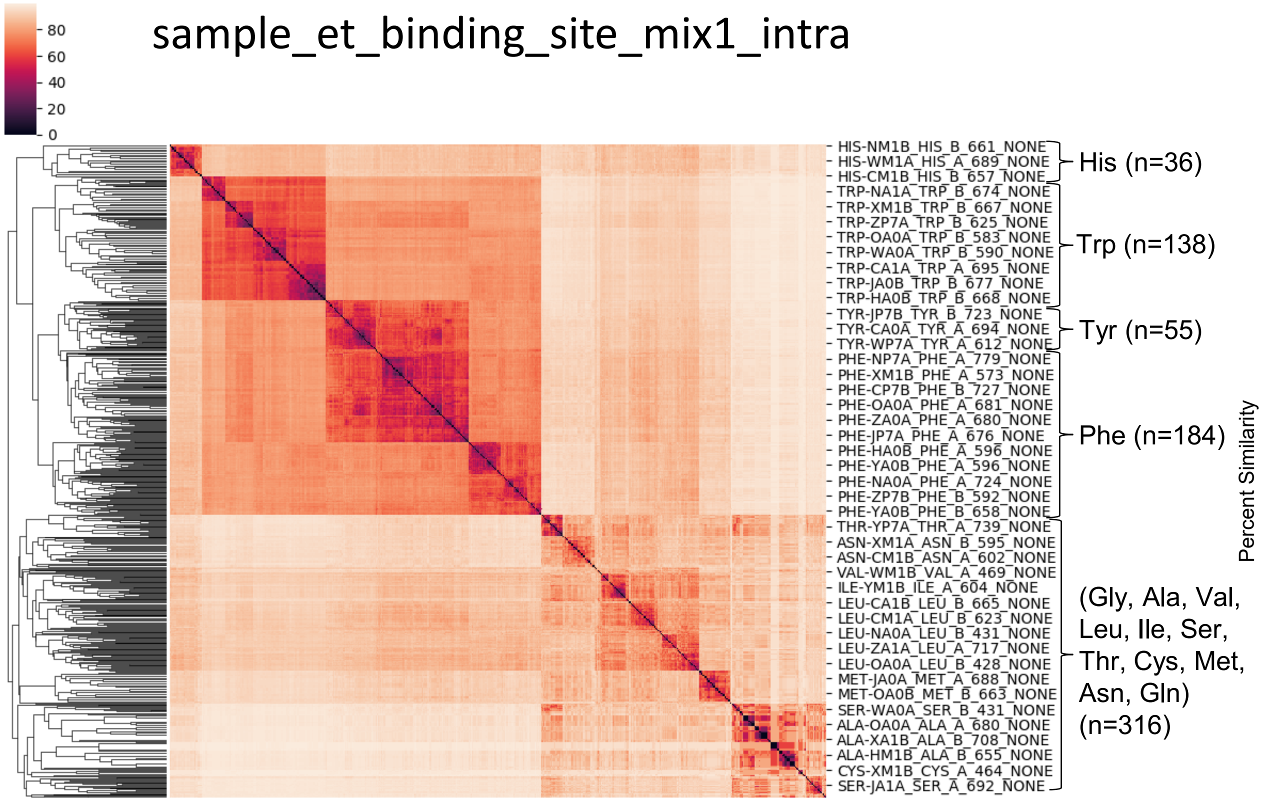

b

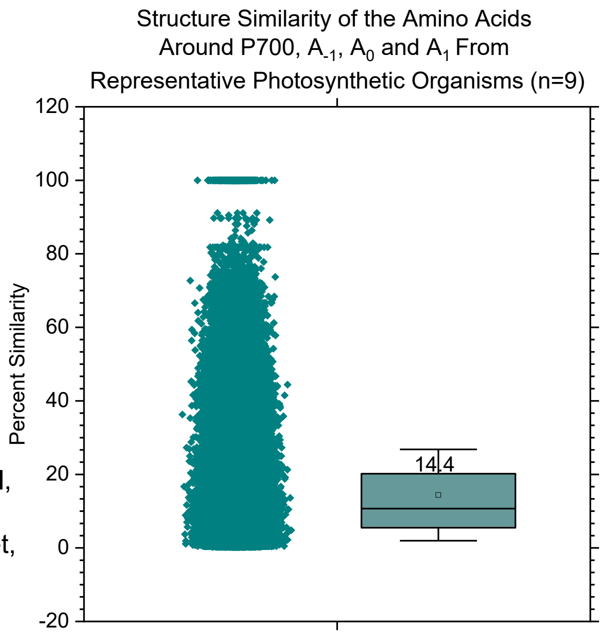

Supplementary Table 1. The Amino Acids that Interact with A_{-1A} and A_{-1B} and Their Positions.

PDB	Organism	Cofactors	H-Bond to the Water	Interacting Amino Acids		Comments
				PsaA	PsaB	
1JB0	*Synechococcus elongatus*	A-1A	PsaB-N591	L674, L677, G678, H680, F681, A684	F587, W588, W595, L626	Cyanobacterium Trimer
		A-1B	PsaA-N604	F456, V460, F544, W601, V608, I646	L657, H660, W663, A664	
5OY0	*Synechocystis sp. PCC 6803*	A-1A	PsaB-N582	I670, L673, A674, H676, F677, A680	F578, W579, W586, F617	Cyanobacterium Trimer
		A-1B	PsaA-N600	F452, I456, F540, W597, I604, I642	L648, H651, W654, A655	
6HQB	*Synechocystis sp. PCC 6803*	A-1A	PsaB-N582	I670, L673, A674, H676, F677, A680	F578, W579, W586, F617	Cyanobacterium Monomer
		A-1B	PsaA-N600	F452, I456, F540, W597, I604, I642	L648, H651, W654, A655	
6JO6	*Chlamydomonas reinhardtii*	A-1A	PsaB-N586	L670, L673, G674, H676, F677, A680	F582, W583, W590, L621	Green Alga Trimer
		A-1B	PsaA-N601	F453, I457, F541, W598, I605, I642	L652, H655, W658, A659	
6KMW	*Halomicronema hongdechloris C2206*	A-1A	PsaB-N593	L683, L686, G687, H689, F690, A693	F589, W590, W597, L628	Cyanobacterium Trimer, Chl *f*, White light
		A-1B	PsaA-N613	F465, V469, F553, W610, I617, I665	L659, H662, W665, A666	
6KMX	*Halomicronema hongdechloris C2206*	A-1A	PsaB-N595	L703, L706, A707, H709, F710, G713	F591, W592, W599, F630	Cyanobacterium Trimer, Chl *f*, Far-red light
		A-1B	PsaA-N633	F460, C464, F573, W630, I637, N675	L661, H664, W667, A668	
6PNJ	*Fischerella thermalis PCC 7521*	A-1A	PsaB-N592	L707, L710, G711, H713, F714, G717	F588, W589, W596, F627	Cyanobacterium Trimer, Far-red light
		A-1B	PsaA-N637	F463, C467, F577, W634, M641, N679	L658, H661, W664, A665	
7COY	*Acaryochloris marina MBIC11017*	A-1A	PsaB-N588	I672, L675, G676, H678, F679, A682	F584, W585, W592, L623	Cyanobacterium Trimer, Far-red light
		A-1B	PsaA-N602	F453, I457, F542, W599, I606, I644	L654, H657, W660, A661	
5ZJI	*Zea mays*	A-1A	PsaB-N585	L669, L672, G673, H675, F676, A679	F581, W582, W589, L620	Plants, Trimer
		A-1B	PsaA-N599	F450, I454, F539, W596, V603, I641	L651, H654, W657, A658	

Supplementary Table 2. The Amino Acids that Interact with A_{0A} and A_{0B} and Their Positions.

PDB	Organism	Cofactors	Axial Ligand	Interacting Amino Acids		Comments
				PsaA	PsaB	
1JB0	*Synechococcus elongatus*	A0A	PsaA-M688	F681, A684, F685, F691, Y696, W697, L700	S429, L430, G433, F434, L538, W588	Cyanobacterium Trimer
		A0B	PsaB-M668	N445, I449, F544, F600, W601	A664, T665, F667, Y676, W677	
5OY0	*Synechocystis sp. PCC 6803*	A0A	PsaA-M684	F677, A680, F681, F687, Y692, W693, L696	S424, L425, G428, F429, L529, W579	Cyanobacterium Trimer
		A0B	PsaB-M659	N441, I445, F540, F596, W597	A655, T656, F658, Y667, W668	
6HQB	*Synechocystis sp. PCC 6803*	A0A	PsaA-M684	F677, A680, F681, F687, Y692, W693, L696	S424, L425, G428, F429, L529, W579	Cyanobacterium Monomer
		A0B	PsaB-M659	N441, I445, F540, F596, W597	A655, T656, F658, Y667, W668	
6JO6	*Chlamydomonas reinhardtii*	A0A	PsaA-M684	F677, A680, F681, F687, Y692, W693, L696	S427, L428, G431, F432, L533, W583	Green Alga Trimer
		A0B	PsaB-M663	N442, I446, F541, F597, W598	A659, T660, F662, Y671, W672	
6KMW	*Halomicronema hongdechloris C2206*	A0A	PsaA-M697	F690, A693, F694, F700, Y705, W706, L709	S431, L432, G435, F436, L540, W590	Cyanobacterium Trimer, Chl *f*, White light
		A0B	PsaB-M670	N454, I458, F553, F609, W610	A666, T667, F669, Y678, W679	
6KMX	*Halomicronema hongdechloris C2206*	A0A	PsaA-M717	F710, G713, F714, F720, Y725, W726, L729	S433, L434, G437, F438, L542, W592	Cyanobacterium Trimer, Chl *f*, Far-red light
		A0B	PsaB-M672	A449, Q453, F573, F629, W630	A668, V669, F671, Y680, W681	
6PNJ	*Fischerella thermalis PCC 7521*	A0A	PsaA-M721	F714, G717, F718, F724, Y729, W730, L733	S430, L431, G434, F435, L539, W589	Cyanobacterium Trimer, Far-red light
		A0B	PsaB-M669	A452, Q456, F577, F633, W634	A665, V666, F668, Y677, W678	
7COY	*Acaryochloris marina MBIC11017*	A0A	PsaA-M686	F679, A682, F683, F689, Y694, W695, L698	S426, M427, G430, F431, L535, W585	Cyanobacterium Trimer, Far-red light
		A0B	PsaB-L665	N442, I446, F542, F598, W599	A661, T662, F664, Y673, W674	
5ZJI	*Zea mays*	A0A	PsaA-M683	F676, A679, F680, F686, Y691, W692, L695	S426, L427, G430, F431, L532, W582	Plants, Trimer
		A0B	PsaB-M662	N439, I443, F539, F595, W596	A658, T659, F669, Y670, W671	

Supplementary Table 3. The Amino Acids that Interact with A_{1A} and A_{1B} and Their Positions.

PDB	Organism	Cofactors	π-stacking	Interacting Amino Acids		Comments
				PsaA	PsaB	
1JB0	*Synechococcus elongatus*	A1A	PsaA-W697	M688, F689, S692, A721, L722	W673	Cyanobacterium Trimer
		A1B	PsaB-W677		M668, F669, S672, W673, A705, L706	
5OY0	*Synechocystis sp. PCC 6803*	A1A	PsaA-W693	M684, F685, S688, A717, L718	W664	Cyanobacterium Trimer
		A1B	PsaB-W668		M659, F660, S663, W664, A696, L697	
6HQB	*Synechocystis sp. PCC 6803*	A1A	PsaA-W693	M684, F685, S688, A717, L718	W664	Cyanobacterium Monomer
		A1B	PsaB-W668		M659, F660, S663, W664, A696, L697	
6JO6	*Chlamydomonas reinhardtii*	A1A	PsaA-W693	M684, F685, S688, A717, L718	W668	Green Alga Trimer
		A1B	PsaB-W672		M663, F684, S667, W668, A700, L701	
6KMW	*Halomicronema hongdechloris C2206*	A1A	PsaA-W706	M697, F698, S701, A730, L731	W675	Cyanobacterium Trimer, Chl *f*, White light
		A1B	PsaB-W679		M670, F671, S674, W675, A707, L708	
6KMX	*Halomicronema hongdechloris C2206*	A1A	PsaA-W726	M717, F718, S721, A750, L751	W677	Cyanobacterium Trimer, Chl *f*, Far-red light
		A1B	PsaB-W681		M672, F673, T676, W677, A708, L709	
6PNJ	*Fischerella thermalis PCC 7521*	A1A	PsaA-W730	M721, F722, S725, A754, L755	W674	Cyanobacterium Trimer, Far-red light
		A1B	PsaB-W678		M669, F670, T673, W674, A705, L706	
7COY	*Acaryochloris marina MBIC11017*	A1A	PsaA-W695	M686, F687, S690, A719, M720	W670	Cyanobacterium Trimer, Far-red light
		A1B	PsaB-W674		L665, F666, S669, W670, G702, L703	
5ZJI	*Zea mays*	A1A	PsaA-W692	M683, F684, S687, A716, L717	W667	Plants, Trimer
		A1B	PsaB-W671		M662, F663, S666, W667, A699, L700	

Supplementary Table 4. Evaluation of the Clustering Results of Protein and Cofactor Structures Using the USR, RMSD and TSR Methods.

**ARI Values**	**Global Protein Structures**	**Cofactor Structures**
**USR**	0	0.544
**RMSD**	1.0	1.0
**TSR**	1.0	1.0

Supplementary_File1

protein	chain_1	chain_2	chain_1_a	chain_2_a	group1	group1	group1	group1	group	group1	group1	group1	group1	percent_th	percent_th	percent_th	percent_threshold1
1JB0	A	B	Unknown	Unknown	Others	Others	Cyanobact	High_Temp	Chl_a	White	Normal_Li	Synechocc	PsaA	100	95	90	85
2O01	A	B	Unknown	Unknown	Others	Others	Plant	Normal_Te	Chl_a	White	Normal_Li	Pisum_sati	PsaA	100	95	90	85
2WSC	A	B	Unknown	Unknown	Others	Others	Plant	Normal_Te	Chl_a	White	Normal_Li	Pisum_sati	PsaA	100	95	90	85
2WSE	A	B	Unknown	Unknown	Others	Others	Plant	Normal_Te	Chl_a	White	Normal_Li	Pisum_sati	PsaA	100	95	90	85
2WSF	A	B	Unknown	Unknown	Others	Others	Plant	Normal_Te	Chl_a	White	Normal_Li	Pisum_sati	PsaA	100	95	90	85
3LW5	A	B	Unknown	Unknown	Others	Others	Plant	Normal_Te	Chl_a	White	Normal_Li	Pisum_sati	PsaA	100	95	90	85
3PCQ	A	B	Unknown	Unknown	Others	Others	Cyanobact	High_Temp	Chl_a	White	Normal_Li	Thermosyn	PsaA	100	95	90	85
4FE1	A	B	Unknown	Unknown	Others	Others	Cyanobact	High_Temp	Chl_a	White	Normal_Li	Thermosyn	PsaA	100	95	90	85
4KT0	A	B	Unknown	Unknown	Others	Others	Cyanobact	Normal_Te	Chl_a	White	Normal_Li	Synechocy	PsaA	100	95	90	85
4L6V	A	B	Unknown	Unknown	Others	Others	Cyanobact	Normal_Te	Chl_a	White	Normal_Li	Synechocy	PsaA	100	95	90	85
4RKU	A	B	Unknown	Unknown	Others	Others	Plant	Normal_Te	Chl_a	White	Normal_Li	Pisum_sati	PsaA	100	95	90	85
4XK8	A	B	Unknown	Unknown	Others	Others	Plant	Normal_Te	Chl_a	White	Normal_Li	Pisum_sati	PsaA	100	95	90	85
4Y28	A	B	Unknown	Unknown	Others	Others	Plant	Normal_Te	Chl_a	White	Normal_Li	Pisum_sati	PsaA	100	95	90	85
5L8R	A	B	Unknown	Unknown	Others	Others	Plant	Normal_Te	Chl_a	White	Normal_Li	Pisum_sati	PsaA	100	95	90	85
5OY0	A	B	Unknown	Unknown	Trimer	Others	Cyanobact	Normal_Te	Chl_a	White	Normal_Li	Synechocy	PsaA	100	95	90	85
5ZF0	A	B	Unknown	Unknown	Others	Others	Cyanobact	High_Temp	Chl_a	White	Normal_Li	Thermosyn	PsaA	100	95	90	85
5ZGB	A	B	Unknown	Unknown	Others	Others	Alga	High_Temp	Chl_a	White	Normal_Li	Cyanidiosc	PsaA	100	95	90	85
5ZGH	A	B	Unknown	Unknown	Others	Others	Alga	High_Temp	Chl_a	White	Normal_Li	Cyanidiosc	PsaA	100	95	90	85
5ZJI	A	B	Unknown	Unknown	Others	Others	Plant	Normal_Te	Chl_a	White	Normal_Li	Zea_mays	PsaA	100	95	90	85
6FOS	A	B	Unknown	Unknown	Others	Others	Alga	High_Temp	Chl_a	White	Normal_Li	Cyanidiosc	PsaA	100	95	90	85
6HQB	A	B	Unknown	Unknown	Monomer	Others	Cyanobact	Normal_Te	Chl_a	White	Normal_Li	Synechocy	PsaA	100	95	90	85
6IGZ	A	B	Unknown	Unknown	Others	Others	Alga	Normal_Te	Chl_a	White	Normal_Li	Bryopsis_c	PsaA	100	95	90	85
6IJJ	A	B	Unknown	Unknown	Others	Others	Alga	Normal_Te	Chl_a	White	Normal_Li	Chlamydo	PsaA	100	95	90	85
6IJO	A	B	Unknown	Unknown	Others	Others	Alga	Normal_Te	Chl_a	White	Normal_Li	Chlamydo	PsaA	100	95	90	85
6JEO	A	B	Unknown	Unknown	Others	Others	Cyanobact	Normal_Te	Chl_a	White	Normal_Li	Nostoc_sp	PsaA	100	95	90	85
6JO5	A	B	Unknown	Unknown	Others	Others	Alga	Normal_Te	Chl_a	White	Normal_Li	Chlamydo	PsaA	100	95	90	85
6JO6	A	B	Unknown	Unknown	Others	Others	Alga	Normal_Te	Chl_a	White	Normal_Li	Chlamydo	PsaA	100	95	90	85
6K33	A	B	Unknown	Unknown	Others	Others	Cyanobact	High_Temp	Chl_a	White	Normal_Li	Thermosyn	PsaA	100	95	90	85
6K61	A	B	Unknown	Unknown	Others	Others	Cyanobact	Normal_Te	Chl_a	White	Normal_Li	Nostoc_sp	PsaA	100	95	90	85
6KIF	A	B	Unknown	Unknown	Others	Others	Cyanobact	High_Temp	Chl_a	White	Normal_Li	Synechocc	PsaA	100	95	90	85
6KIG	A	B	Unknown	Unknown	Others	Others	Cyanobact	High_Temp	Chl_a	White	Normal_Li	Synechocc	PsaA	100	95	90	85
6KMW	A	B	Unknown	Unknown	Others	White	Cyanobact	Normal_Te	Chl_a	White	Normal_Li	Halomicro	PsaA	100	95	90	85
6KMX	A	B	Unknown	Unknown	Others	Red	Cyanobact	Normal_Te	Chl_f	Red	Normal_Li	Halomicro	PsaA	100	95	90	85
6L35	A	B	Unknown	Unknown	Others	Others	Plant	Normal_Te	Chl_a	White	Normal_Li	Physcomit	PsaA	100	95	90	85
6L4U	A	B	Unknown	Unknown	Others	Others	Alga	Normal_Te	Chl_a	White	Normal_Li	Chaetocer	PsaA	100	95	90	85
6LU1	A	B	Unknown	Unknown	Others	Others	Cyanobact	High_Temp	Chl_a	White	Normal_Li	Thermosyn	PsaA	100	95	90	85
6NWA	A	B	Unknown	Unknown	Others	Others	Cyanobact	Normal_Te	Chl_a	White	Normal_Li	Synechocy	PsaA	100	95	90	85
6PFY	A	B	Unknown	Unknown	Others	Others	Cyanobact	High_Temp	Chl_a	White	Normal_Li	Thermosyn	PsaA	100	95	90	85
6PGK	A	B	Unknown	Unknown	Others	Others	Cyanobact	High_Temp	Chl_a	White	Normal_Li	Thermosyn	PsaA	100	95	90	85
6PNJ	A	B	Unknown	Unknown	Others	Others	Cyanobact	High_Temp	Chl_f	Red	Normal_Li	Fischerella	PsaA	100	95	90	85
6QPH	A	B	Unknown	Unknown	Others	Others	Alga	Normal_Te	Chl_a	White	Normal_Li	Dunaliella_	PsaA	100	95	90	85
6QWJ	A	B	Unknown	Unknown	Others	Others	Cyanobact	Normal_Te	Chl_a	White	Normal_Li	Chroococc	PsaA	100	95	90	85
6RHZ	A	B	Unknown	Unknown	Others	Others	Alga	Normal_Te	Chl_a	White	Normal_Li	Dunaliella_	PsaA	100	95	90	85
6SL5	A	B	Unknown	Unknown	Others	Others	Alga	Normal_Te	Chl_a	White	Normal_Li	Dunaliella_	PsaA	100	95	90	85
6TCL	A	B	Unknown	Unknown	Others	Others	Cyanobact	Normal_Te	Chl_a	White	Normal_Li	Nostoc_sp	PsaA	100	95	90	85

6TRA	A	B	Unknown	Unknown	Others	Others	Cyanobact	Normal_Te	Chl_a	White	Normal_Lig	Thermosyn	PsaA	100	95	90	85
6TRC	A	B	Unknown	Unknown	Others	Others	Cyanobact	Normal_Te	Chl_a	White	Normal_Lig	Thermosyn	PsaA	100	95	90	85
6TRD	A	B	Unknown	Unknown	Others	Others	Cyanobact	Normal_Te	Chl_a	White	Normal_Lig	Thermosyn	PsaA	100	95	90	85
6UZV	A	B	Unknown	Unknown	Others	Others	Cyanobact	Normal_Te	Chl_a	White	Normal_Lig	Synechocy	PsaA	100	95	90	85
6VPV	A	B	Unknown	Unknown	Others	Others	Cyanobact	Normal_Te	Chl_a	White	High_Light	Cyanobact	PsaA	100	95	90	85
6YAC	A	B	Unknown	Unknown	Others	Others	Plant	Normal_Te	Chl_a	White	Normal_Lig	Pisum_sati	PsaA	100	95	90	85
6YEZ	A	B	Unknown	Unknown	Others	Others	Plant	Normal_Te	Chl_a	White	Normal_Lig	Pisum_sati	PsaA	100	95	90	85
6YXR	A	B	Unknown	Unknown	Others	Others	Alga	Normal_Te	Chl_a	White	Normal_Lig	Dunaliella_	PsaA	100	95	90	85
7BLZ	A	B	Unknown	Unknown	Others	Others	Alga	Normal_Te	Chl_a	White	Normal_Lig	Cyanidiosc	PsaA	100	95	90	85
7BW2	A	B	Unknown	Unknown	Others	Others	Cyanobact	Normal_Te	Chl_a	White	Normal_Lig	Thermosyn	PsaA	100	95	90	85
7COY	A	B	Unknown	Unknown	Others	Others	Cyanobact	Normal_Te	Chl_d	White	Normal_Lig	Acaryochlc	PsaA	100	95	90	85
7D0J	A	B	Unknown	Unknown	Others	Others	Alga	Normal_Te	Chl_a	White	Normal_Lig	Chlamydor	PsaA	100	95	90	85
7DKZ	A	B	Unknown	Unknown	Others	Others	Plant	Normal_Te	Chl_a	White	Normal_Lig	Pisum_sati	PsaA	100	95	90	85
7DR0	A	B	Unknown	Unknown	Others	Others	Alga	Normal_Te	Chl_a	White	Normal_Lig	Cyanophor	PsaA	100	95	90	85
7DR1	A	B	Unknown	Unknown	Others	Others	Alga	Normal_Te	Chl_a	White	Normal_Lig	Cyanophor	PsaA	100	95	90	85
7DR2	A	B	Unknown	Unknown	Others	Others	Alga	Normal_Te	Chl_a	White	Normal_Lig	Cyanophor	PsaA	100	95	90	85
7EW6	A	B	Unknown	Unknown	Others	Others	Plant	Normal_Te	Chl_a	White	Normal_Lig	Hordeum_v	PsaA	100	95	90	85
7EWK	A	B	Unknown	Unknown	Others	Others	Plant	Normal_Te	Chl_a	White	Normal_Lig	Hordeum_v	PsaA	100	95	90	85
7F4V	A	B	Unknown	Unknown	Others	Others	Cyanobact	Normal_Te	Chl_a	White	Normal_Lig	Gloeobact	PsaA	100	95	90	85
7KSQ	A	B	Unknown	Unknown	Others	Others	Plant	Normal_Te	Chl_a	White	Normal_Lig	Physcomit	PsaA	100	95	90	85
7KUX	A	B	Unknown	Unknown	Others	Others	Plant	Normal_Te	Chl_a	White	Normal_Lig	Physcomit	PsaA	100	95	90	85
7LX0	A	B	Unknown	Unknown	Others	Others	Cyanobact	Normal_Te	Chl_f	White	Normal_Lig	Fischerella	PsaA	100	95	90	85
7QCO	A	B	Unknown	Unknown	Others	Others	Cyanobact	High_Temp	Chl_a	White	Normal_Lig	Chroococc	PsaA	100	95	90	85
7WFD	A	B	Unknown	Unknown	Others	Others	Plant	Normal_Te	Chl_a	White	Normal_Lig	Arabidopsi	PsaA	100	95	90	85
7WFE	A	B	Unknown	Unknown	Others	Others	Plant	Normal_Te	Chl_a	White	Normal_Lig	Arabidopsi	PsaA	100	95	90	85
7WG5	A	B	Unknown	Unknown	Others	Others	Plant	Normal_Te	Chl_a	White	Normal_Lig	Arabidopsi	PsaA	100	95	90	85

Python codes and input files

(i) TSR key generation code for proteins using C alpha atoms
(ii) TSR key generation code for pigments using all atoms except hydrogen atoms
(iii) TSR key generation code for tryptophan using all atoms except hydrogen atoms
(iv) USR code for proteins using C alpha atoms
(v) USR code for pigments using all atoms except hydrogen atoms
(vi) USR code for tryptophan using all atoms except hydrogen atoms
(vii) RMSD code for pigments using all atoms except hydrogen atoms
(viii) RMSD code for tryptophan using all atoms except hydrogen atoms
(ix) aminoAcidCode_lexicographic_new.txt
(x) drug_atom_lexical_txt.csv
(xi) sample_details_psi_ab_mix1.csv
(xii) sample_details_et_mix5_name_id_atom.csv

The source code is available for academic users on GitHub: https://github.com/tarikulislammilon/TSR and https://github.com/WuXu26/Protein-3D-TSR. If you have any questions about the Python codes, please contact at wxx6941@louisiana.edu. If you need supercomputer access to run any of the Python codes, please contact us. We can provide access to the supercomputers up to for the ten scientists who want to use the TSR method at the same time.

(i) TSR key generation code for proteins using C alpha atoms

#Python code: to calculate "key files" and "triplet file" for protein 3D structures
#Input files: (i) aminoAcidCode_lexicographic_new.csv; (ii) sample_details_psi_ab_mix1.csv;
#(iii) pdb
#Output files: "key files" and "triplet files" will be generated for protein

```
# Author:Tarikul Islam Milon
#Created on: 10/27/2024


import csv
import math
import Bio.PDB
from Bio.PDB import PDBParser
import pandas as pd
import os

dTheta = 29
dLen = 35
numOfLabels = 20


df = pd.read_csv('sample_details_psi_ab_mix1.csv')
PDB_list = df['protein'].to_list()
Chain = df['chain'].to_list()
Group_Infor = df['group'].to_list()

atomSeq = {}
atomSeqNumber = open("aminoAcidCode_lexicographic_new.txt", 'r')
lines = atomSeqNumber.readlines()
for line in lines:
    aa_Res = line.split()[0]
    aa_Res_No =line.split()[1]
    atomSeq[aa_Res]=aa_Res_No
atomSeqNumber.close()


# Theta Bin for 3D
def thetaClass_(Theta):
    # classT=0
    if Theta >= 0 and Theta < 12.11:
        classT = 1
    elif Theta >= 12.11 and Theta < 17.32:
        classT = 2
    elif Theta >= 17.32 and Theta < 21.53:
        classT = 3
    elif Theta >= 21.53 and Theta < 25.21:
        classT = 4
    elif Theta >= 25.21 and Theta < 28.54:
        classT = 5
    elif Theta >= 28.54 and Theta < 31.64:
        classT = 6
    elif Theta >= 31.64 and Theta < 34.55:
```

```
        classT = 7
    elif Theta >= 34.55 and Theta < 37.34:
        classT = 8
    elif Theta >= 37.34 and Theta < 40.03:
        classT = 9
    elif Theta >= 40.03 and Theta < 42.64:
        classT = 10
    elif Theta >= 42.64 and Theta < 45.17:
        classT = 11
    elif Theta >= 45.17 and Theta < 47.64:
        classT = 12
    elif Theta >= 47.64 and Theta < 50.05:
        classT = 13
    elif Theta >= 50.05 and Theta < 52.43:
        classT = 14
    elif Theta >= 52.43 and Theta < 54.77:
        classT = 15
    elif Theta >= 54.77 and Theta < 57.08:
        classT = 16
    elif Theta >= 57.08 and Theta < 59.38:
        classT = 17
    elif Theta >= 59.38 and Theta < 61.64:
        classT = 18
    elif Theta >= 61.64 and Theta < 63.87:
        classT = 19
    elif Theta >= 63.87 and Theta < 66.09:
        classT = 20
    elif Theta >= 66.09 and Theta < 68.30:
        classT = 21
    elif Theta >= 68.30 and Theta < 70.5:
        classT = 22
    elif Theta >= 70.5 and Theta < 72.69:
        classT = 23
    elif Theta >= 72.69 and Theta < 79.2:
        classT = 24
    elif Theta >= 79.2 and Theta < 81.36:
        classT = 25
    elif Theta >= 81.36 and Theta < 83.51:
        classT = 26
    elif Theta >= 83.51 and Theta < 85.67:
        classT = 27
    elif Theta >= 85.67 and Theta < 87.80:
        classT = 28
    elif Theta >= 87.80 and Theta <= 90.00:
        classT = 29
    return classT

# maxDist bin for 3D
def dist12Class_(dist12):
    #classL=0
    if (dist12<3.83):
        classL=1
    elif dist12>=3.83 and dist12<7.00:
        classL=2
```

```
elif dist12>=7.00 and dist12<9.00:
    classL=3
elif dist12>=9.00 and dist12<11.00:
    classL=4
elif dist12>=11.00 and dist12<14.00:
    classL=5
elif dist12>=14.00 and dist12<17.99:
    classL=6
elif dist12>=17.99 and dist12<21.25:
    classL=7
elif dist12>=21.25 and dist12<23.19:
    classL=8
elif dist12>=23.19 and dist12<24.8:
    classL=9
elif dist12>=24.8 and dist12<26.26:
    classL=10
elif dist12>=26.26 and dist12<27.72:
    classL=11
elif dist12>=27.72 and dist12<28.9:
    classL=12
elif dist12>=28.9 and dist12<30.36:
    classL=13
elif dist12>=30.36 and dist12<31.62:
    classL=14
elif dist12>=31.62 and dist12<32.76:
    classL=15
elif dist12>=32.76 and dist12<33.84:
    classL=16
elif dist12>=33.84 and dist12<35.13:
    classL=17
elif dist12>=35.13 and dist12<36.26:
    classL=18
elif dist12>=36.26 and dist12<37.62:
    classL=19
elif dist12>=37.62 and dist12<38.73:
    classL=20
elif dist12>=38.73 and dist12<40.12:
    classL=21
elif dist12>=40.12 and dist12<41.8:
    classL=22
elif dist12>=41.8 and dist12<43.41:
    classL=23
elif dist12>=43.41 and dist12<45.55:
    classL=24
elif dist12>=45.55 and dist12<47.46:
    classL=25
elif dist12>=47.46 and dist12<49.69:
    classL=26
elif dist12>=49.69 and dist12<52.65:
    classL=27
elif dist12>=52.65 and dist12<55.81:
    classL=28
elif dist12>=55.81 and dist12<60.2:
    classL=29
```

```
    elif dist12>=60.2 and dist12<64.63:
        classL=30
    elif dist12>=64.63 and dist12<70.04:
        classL=31
    elif dist12>=70.04 and dist12<76.15:
        classL=32
    elif dist12>=76.15 and dist12<83.26:
        classL=33
    elif dist12>=83.26 and dist12<132.45:
        classL=34
    elif dist12>=132.45:
        classL=35
    return classL



def calDist(x1, y1, z1, x2, y2, z2):
    return math.sqrt((x2 - x1) ** 2 + (y2 - y1) ** 2 + (z2 - z1) ** 2)


Key_Dict_Total = ()
DataFrame_Index = []
Group_Information = []

Output_Folder_Path =
'/ddnB/work/wxx6941/TSR/code/code/psi_revision/9pdb/ca_tsr_a_b/output_ca_
all'
outputFile3=open('Sample_details2_output.txt','w')
for i in range(len(PDB_list)):
    PDB_ID = PDB_list[i]
    Chain_Name = Chain[i]
    Group = Group_Infor[i]

    PDB_File_Path =
"/ddnB/work/wxx6941/TSR/code/code/psi_revision/9pdb/ca_tsr_a_b/pdb/{}.pdb
".format(PDB_ID)

    keyDict3D = {}
    xCoord = {}
    yCoord = {}
    zCoord = {}
    Atom = {}
    Res={}
    res_Id={}

    counter2 = 0
    File=open(PDB_File_Path,'r')
    lines_ = File.readlines()
    outputFile1 = open(
f'{Output_Folder_Path}/{PDB_ID}_{Chain_Name}.triplets_theta29_dist35',
'w')
    outputFile2 =
open(f'{Output_Folder_Path}/key_{PDB_ID}_{Chain_Name}.keys_theta29_dist35
', 'w')
```

```
    # header
    outputFile1.writelines( 'Residue1   Residue2   Residue3   Edge1
Edge2  Edge3\t   Coor_R1\t           Coor_R2\t
CoorR3\tTheta\tmax_dist\td_3\tkey3D\tLabel1\tLabel2\tLabel3\n')
    outputFile2.writelines('key\t\tfreq\n')

    counter = 0
    for line in lines_:
        if line.startswith('ATOM') or line.startswith('HETATM'):
            if line[21].strip() == Chain_Name:
                if line[13:15].strip() == 'CA':
                    xCoord[counter] = float(line[30:38])
                    yCoord[counter] = float(line[38:46])
                    zCoord[counter] = float(line[46:54])

                    Res[counter] = line[17:20].strip()

                    res_Id[counter] = line[22:27].strip()
                    Atom[counter] = line[13:15].strip()
                    counter += 1

                    print(line[22:27].strip())
        if line.startswith('TER') and line[21].strip() == Chain_Name:
            break
    Max_Dist={}
    Min_Dist={}
    Number_of_Triangles=0
    for i in range(len(xCoord)):
        for j in range(i + 1, len(yCoord)):
            for k in range(j + 1, len(zCoord)):
                L1 = calDist(xCoord[i], yCoord[i], zCoord[i], xCoord[j],
yCoord[j], zCoord[j])
                L2 = calDist(xCoord[j], yCoord[j], zCoord[j], xCoord[k],
yCoord[k], zCoord[k])
                L3 = calDist(xCoord[i], yCoord[i], zCoord[i], xCoord[k],
yCoord[k], zCoord[k])

                l1 = int(atomSeq[Res[j]])
                l2 = int(atomSeq[Res[k]])
                l3 = int(atomSeq[Res[i]])

                Med1 = (1 / 2) * math.sqrt(2 * (L1 ** 2) + 2 * (L2 ** 2)
- L3 ** 2)
                Med2 = (1 / 2) * math.sqrt(2 * (L2 ** 2) + 2 * (L3 ** 2)
- L1 ** 2)
                Med3 = (1 / 2) * math.sqrt(2 * (L3 ** 2) + 2 * (L1 ** 2)
- L2 ** 2)
                Median = [Med1, Med2, Med3]
                Label = [l1, l2, l3]
                index1 = [L3, L1, L2]

                # 1st Condition
                if l1 != l2 != l3:
                    X = [l1, l2, l3]
```

```
                    b3 = Median[Label.index(min(l1, l2, l3))]
                    d12 = index1[Label.index(min(l1, l2, l3))]
                    if d12 == L3 and max(l1, l2, l3) == l2:
                        d13 = L2
                    elif d12 == L3 and max(l1, l2, l3) == l3:
                        d13 = L1

                    elif d12 == L2 and max(l1, l2, l3) == l1:
                        d13 = L1
                    elif d12 == L2 and max(l1, l2, l3) == l2:
                        d13 = L3
                    elif d12 == L1 and max(l1, l2, l3) == l1:
                        d13 = L2
                    elif d12 == L1 and max(l1, l2, l3) == l3:
                        d13 = L3
                    X.remove(max(X))
                    X.remove(min(X))
                    Label1 = max(l1, l2, l3)
                    Label2 = X[0]
                    Label3 = min(l1, l2, l3)
                    #outputFile1.writelines(f'{Label1}  {Label2}
{Label3} {max(l1, l2, l3)} {l1} {l2} {l3}')

                # 2nd condition
                elif l1 > l2 == l3:
                    Label1 = l1
                    if L2 > L1:
                        b3 = Med3
                        d13 = L1
                        d12 = L2
                        Label2 = l2
                        Label3 = l3
                    else:
                        b3 = Med2
                        d13 = L2
                        d12 = L1
                        Label2 = l3
                        Label3 = l2

                elif l2 > l1 == l3:
                    Label1 = l2
                    if L3 > L2:
                        b3 = Med1
                        d13 = L2
                        d12 = L3
                        Label2 = l3
                        Label3 = l1
                    else:
                        b3 = Med3
                        d13 = L3
                        d12 = L2
                        Label2 = l1
                        Label3 = l3
```

```
elif l3 > l1 == l2:
    Label1 = l3
    if L1 > L3:
        b3 = Med2
        d13 = L3
        d12 = L1
        Label2 = l1
        Label3 = l2
    else:
        b3 = Med1
        d13 = L1
        d12 = L3
        Label2 = l2
        Label3 = l1
# 3rd condition
elif l1 == l2 > l3:
    b3 = Med3
    Label3 = l3
    if L1 > L3:
        d13 = L1
        d12 = L2
        Label1 = l1
        Label2 = l2
    else:
        d13 = L3
        d12 = L2
        Label1 = l2
        Label2 = l1

elif l1 == l3 > l2:
    Label3 = l2
    b3 = Med2
    if L2 > L3:
        d13 = L2
        d12 = L1
        Label1 = l1
        Label2 = l3
    else:
        d13 = L3
        d12 = L1
        Label1 = l3
        Label2 = l1
elif l2 == l3 > l1:
    Label3 = l1
    b3 = Med1
    if L2 > L1:
        d13 = L2
        d12 = L3
        Label1 = l2
        Label2 = l3
    else:
        d13 = L1
        d12 = L3
        Label1 = l3
```

```
                Label2 = l2

        # 4th condition
        if l1 == l2 == l3:
            if L2 >= max(L1, L2, L3):
                b3 = Med3
                d13 = L1
                d12 = L2
                Label1 = l1
                Label2 = l2
                Label3 = l3
            if L1 >= max(L1, L2, L3):
                b3 = Med2
                d13 = L2
                d12 = L1
                Label1 = l1
                Label2 = l3
                Label3 = l2

            if L3 >= max(L1, L2, L3):
                # b3=Med3
                # d13 =L1
                # d12 =L2
                # Corrected
                b3 = Med1
                d13 = L1
                d12 = L3
                Label1 = l3
                Label2 = l2
                Label3 = l1

        a = (d13 ** 2 - (d12 / 2) ** 2 - b3 ** 2)
        b = (2 * (d12 / 2) * b3)
        if L1!=0 and L2!=0 and L3!=0:
            Theta1 = (math.acos(a / b)) * (180 / math.pi)

            if Theta1 <= 90:
                Theta = Theta1
            else:
                Theta = abs(180 - Theta1)
            maxDist = max(L1, L2, L3)
            minDist = min(L1, L2, L3)

            Max_Dist[Number_of_Triangles] = maxDist
            Min_Dist[Number_of_Triangles] = minDist
            Number_of_Triangles += 1

            ClassT1 = thetaClass_(Theta)
            ClassL1 = dist12Class_(maxDist)
            if maxDist >= 0 and maxDist <= 5000:
                key3D = dLen * dTheta * (numOfLabels ** 2) *
(int(Label1) - 1) + \
                        dLen * dTheta * (numOfLabels) *
(int(Label2) - 1) + \
```

```
                                dLen * dTheta * (int(Label3) - 1) + \
                                dTheta * (ClassL1 - 1) + \
                                (ClassT1 - 1)

                        if key3D in keyDict3D:
                            keyDict3D[key3D] += 1
                        else:
                            keyDict3D[key3D] = 1

                        outputFile1.write("{}_{}_{}_{}  ".format(Res[i],
Chain_Name, res_Id[i], Atom[i]))
                        outputFile1.write("{}_{}_{}_{}  ".format(Res[j],
Chain_Name, res_Id[j], Atom[j]))
                        outputFile1.write("{}_{}_{}_{}  ".format(Res[k],
Chain_Name, res_Id[k], Atom[k]))
                        outputFile1.write(" {:.2f}  {:.2f}  {:.2f}
".format(L1, L2, L3))
                        outputFile1.write(
                            "  {:.2f},{:.2f},{:.2f}
{:.2f},{:.2f},{:.2f}  ".format(xCoord[i], yCoord[i], zCoord[i],

xCoord[j], yCoord[j], zCoord[j]))

                        outputFile1.write(" {:.2f},{:.2f},{:.2f}
".format(xCoord[k], yCoord[k], zCoord[k]))
                        outputFile1.write(
                            "{:.2f}   {:.2f}   {:.2f}   {:.0f} {} {} {}
\n".format(Theta, maxDist, b3, key3D,

int(Label1),

int(Label2), int(Label3)))
    for value_ in keyDict3D:
        outputFile2.writelines([str(value_), '\t',
str(keyDict3D[value_]), '\n'])

    Key_Dict_Total += (keyDict3D,)

    DataFrame_Index.append(f'{PDB_ID}_{Chain_Name}')
    Group_Information.append(Group)

    outputFile3.writelines(

f'{PDB_ID}\t{Chain_Name}\t{len(Res)}\t{len(keyDict3D)}\t{sum(keyDict3D.va
lues())}\t{max(Max_Dist.values())}\t{min(Max_Dist.values())}\n')

    outputFile1.close()
    outputFile2.close()



outputFile3.close()
df=pd.DataFrame(Key_Dict_Total,index=Group_Information)
df=df.rename_axis('group')
```

```
df=df.fillna(0)
df = df.astype('int')
df.insert(0,'protein',DataFrame_Index)
df.to_csv(f"{Output_Folder_Path}/feature_map_with_header.csv",header=True
,index=True)

df_Group=pd.DataFrame(columns=['group'],index=DataFrame_Index)
df_Group=df_Group.rename_axis('protein')
df_Group['group']=Group_Information
df_Group.to_csv(f"{Output_Folder_Path}/sample_details.csv",header=True,in
dex=True)

df_Clustering=pd.DataFrame(Key_Dict_Total,index=DataFrame_Index)
df_Clustering=df_Clustering.fillna(0)
df_Clustering = df_Clustering.astype('int')

first_column = list(df_Clustering.iloc[:, 0])
DataFrame_Index_2=[]
for i in range(len(df_Clustering)):

DataFrame_Index_2.append(DataFrame_Index[i]+";"+str(int(first_column[i]))
)
df_Clustering.iloc[:, 0]=DataFrame_Index_2
df_Clustering.to_csv(f"{Output_Folder_Path}/localFeatureVect_theta29_dist
35_NoFeatureSelection_keyCombine0.csv",header=False,index=False)
print('completed Successfully')
```

(ii) TSR key generation code for pigments using all atoms except hydrogen atoms

#Python code: to calculate “key files” and “triplet file” for chlorophyll 3D structures
#Input files: (i) drug_atom_lexical_txt.csv; (ii) sample_details_et_mix5_name_id_atom.csv;
#(iii) pdb
#Output files: “key files” and “triplet files” will be generated for each chlorophyll

```
# Program to calculate only drug triplets file and key freq
# Author:Tarikul Islam Milon
# Created on: 10/21/2024


import csv
import math
import Bio.PDB
from Bio.PDB import PDBParser
import pandas as pd
import os

dTheta = 29
dLen = 17
numOfLabels = 112

df = pd.read_csv('sample_details_et_mix5_name_id_atom.csv')
PDB_list = df['protein'].to_list()
Chain = df['chain'].to_list()
drug_name = df['drug_name'].to_list()
drug_id = df['drug_id'].to_list()
Group = df['group'].to_list()
Atom_list=df['ligand_atom'].to_list()

print(drug_name)
print(drug_id)
print(PDB_list)
print(Chain)

atomSeq = {}
atomSeqNumber = open("drug_atom_lexical_txt.csv", 'r')
reader_ = csv.reader(atomSeqNumber)
next(reader_)
for row in reader_:
    atomSeq[row[2]] = row[1]
atomSeqNumber.close()


# for i in drug_name:
# print(type(i),i)

# Theta Bin for 3D
def thetaClass_(Theta):
    # classT=0
    if Theta >= 0 and Theta < 12.11:
        classT = 1
    elif Theta >= 12.11 and Theta < 17.32:
        classT = 2
```

```
elif Theta >= 17.32 and Theta < 21.53:
    classT = 3
elif Theta >= 21.53 and Theta < 25.21:
    classT = 4
elif Theta >= 25.21 and Theta < 28.54:
    classT = 5
elif Theta >= 28.54 and Theta < 31.64:
    classT = 6
elif Theta >= 31.64 and Theta < 34.55:
    classT = 7
elif Theta >= 34.55 and Theta < 37.34:
    classT = 8
elif Theta >= 37.34 and Theta < 40.03:
    classT = 9
elif Theta >= 40.03 and Theta < 42.64:
    classT = 10
elif Theta >= 42.64 and Theta < 45.17:
    classT = 11
elif Theta >= 45.17 and Theta < 47.64:
    classT = 12
elif Theta >= 47.64 and Theta < 50.05:
    classT = 13
elif Theta >= 50.05 and Theta < 52.43:
    classT = 14
elif Theta >= 52.43 and Theta < 54.77:
    classT = 15
elif Theta >= 54.77 and Theta < 57.08:
    classT = 16
elif Theta >= 57.08 and Theta < 59.38:
    classT = 17
elif Theta >= 59.38 and Theta < 61.64:
    classT = 18
elif Theta >= 61.64 and Theta < 63.87:
    classT = 19
elif Theta >= 63.87 and Theta < 66.09:
    classT = 20
elif Theta >= 66.09 and Theta < 68.30:
    classT = 21
elif Theta >= 68.30 and Theta < 70.5:
    classT = 22
elif Theta >= 70.5 and Theta < 72.69:
    classT = 23
elif Theta >= 72.69 and Theta < 79.2:
    classT = 24
elif Theta >= 79.2 and Theta < 81.36:
    classT = 25
elif Theta >= 81.36 and Theta < 83.51:
    classT = 26
elif Theta >= 83.51 and Theta < 85.67:
    classT = 27
elif Theta >= 85.67 and Theta < 87.80:
    classT = 28
elif Theta >= 87.80 and Theta <= 90.00:
    classT = 29
```

```
    return classT


# MaxDist bin for 3D
def dist12Class_(dist12):
    if dist12 >= 0 and dist12 < 1:
        classL = 1
    elif dist12 >= 1 and dist12 < 2:
        classL = 2
    elif dist12 >= 2 and dist12 < 3:
        classL = 3
    elif dist12 >= 3 and dist12 < 4:
        classL = 4
    elif dist12 >= 4 and dist12 < 5:
        classL = 5
    elif dist12 >= 5 and dist12 < 6:
        classL = 6
    elif dist12 >= 6 and dist12 < 7:
        classL = 7
    elif dist12 >= 7 and dist12 < 8:
        classL = 8
    elif dist12 >= 8 and dist12 < 9:
        classL = 9
    elif dist12 >= 9 and dist12 < 10:
        classL = 10
    elif dist12 >= 10 and dist12 < 11:
        classL = 11
    elif dist12 >= 11 and dist12 < 12:
        classL = 12
    elif dist12 >= 12 and dist12 < 13:
        classL = 13
    elif dist12 >= 13 and dist12 < 14:
        classL = 14
    elif dist12 >= 14 and dist12 < 15:
        classL = 15
    elif dist12 >= 15 and dist12 < 16:
        classL = 16
    elif dist12 >= 16 and dist12 < 1000:
        classL = 17

    return classL


def calDist(x1, y1, z1, x2, y2, z2):
    return math.sqrt((x2 - x1) ** 2 + (y2 - y1) ** 2 + (z2 - z1) ** 2)


Key_Dict_Total = ()
DataFrame_Index = []
Group_Information = []

Output_Folder_Path =
'/ddnB/work/wxx6941/TSR/code/code/psi_revision/5pdb/output_chl_atom_selec
tion_tsr'
```

```
outputFile3 = open(f'{Output_Folder_Path}/sample_details_file2.txt', 'w')
outputFile3.writelines('File_ID\t\t\tTotal atom\tTotal key\tTotal
Distinct key\n')


for i in range(len(PDB_list)):
    PDB_ID = PDB_list[i]
    Chain_Name = Chain[i]
    drug_Name = drug_name[i]
    drug_Id = str(drug_id[i])
    #print(drug_Name)
    Group_ = Group[i]
    atoms=Atom_list[i].split(";")
    #print(atoms)
    if drug_Name == 'CL0':
        #drug_Name='017'
        drug_Id=f'0{drug_Id}'
    #print(PDB_ID)


    PDB_File_Path =
"/ddnB/work/wxx6941/TSR/code/code/psi_revision/5pdb/PDB_datadir_HRemoved/
{}.pdb".format(
        PDB_ID)
    p = Bio.PDB.PDBParser()
    Structure = p.get_structure('PrimaryStructureChain', PDB_File_Path)
    model = Structure[0]
    for chain in model:
        if chain.id == Chain_Name:
            for residue in chain:
                drug_Identifier = str(residue)[17:18].strip()
                resName = str(residue)[9:12].strip()  # residue Name

                numeric_filter = filter(str.isdigit, str(residue.id))
                Res_Id = "".join(numeric_filter)  # Residue ID
                print(resName,Res_Id)

                if drug_Identifier == 'H' and resName == drug_Name and
Res_Id == drug_Id:
                    for atom0 in residue:
                        Coord_end = atom0.get_vector()
                        X1_end = Coord_end[0]
                    X1_end = '{:.2f}'.format(X1_end)

                    outputFile1 = open(

f'{Output_Folder_Path}/{PDB_ID}_{Chain_Name}_{drug_Name}_{drug_Id}_{X1_en
d}.triplets_theta29_dist17',
                        'w')
                    outputFile2 = open(

f'{Output_Folder_Path}/key_{PDB_ID}_{Chain_Name}_{drug_Name}_{drug_Id}_{X
1_end}.keys_theta29_dist17',
                        'w')
```

```
                    # header
                    outputFile1.writelines(
                        'Residue1   Residue2   Residue3   Edge1  Edge2
Edge3\t   Coor_R1\t           Coor_R2\t
CoorR3\tTheta\tmax_dist\td_3\tkey3D\n')
                    outputFile2.writelines('key\t\tfreq\n')

                    keyDict3D = {}
                    xCoord = {}
                    yCoord = {}
                    zCoord = {}
                    Atom = {}
                    counter2 = 0
                    for atom1 in residue:
                        #print(atom1)
                        if atom1.get_name() in atoms:
                            #print('done')
                            atomCoord = atom1.get_vector()

                            Atom[counter2] = atom1.get_name()
                            xCoord[counter2] = atomCoord[0]
                            yCoord[counter2] = atomCoord[1]
                            zCoord[counter2] = atomCoord[2]

                            counter2 += 1



                    Total_key = 0
                    for i in range(len(xCoord)):
                        for j in range(i + 1, len(yCoord)):
                            for k in range(j + 1, len(zCoord)):
                                L1 = calDist(xCoord[i], yCoord[i],
zCoord[i], xCoord[j], yCoord[j], zCoord[j])
                                L2 = calDist(xCoord[j], yCoord[j],
zCoord[j], xCoord[k], yCoord[k], zCoord[k])
                                L3 = calDist(xCoord[i], yCoord[i],
zCoord[i], xCoord[k], yCoord[k], zCoord[k])

                                l1 = atomSeq[Atom[j]]
                                l2 = atomSeq[Atom[k]]
                                l3 = atomSeq[Atom[i]]

                                Med1 = (1 / 2) * math.sqrt(2 * (L1 ** 2)
+ 2 * (L2 ** 2) - L3 ** 2)
                                Med2 = (1 / 2) * math.sqrt(2 * (L2 ** 2)
+ 2 * (L3 ** 2) - L1 ** 2)
                                Med3 = (1 / 2) * math.sqrt(2 * (L3 ** 2)
+ 2 * (L1 ** 2) - L2 ** 2)
                                Median = [Med1, Med2, Med3]
                                Label = [l1, l2, l3]
                                index1 = [L3, L1, L2]

                                # 1st Condition
```

```
                if l1 != l2 != l3:
                    X = [l1, l2, l3]
                    b3 = Median[Label.index(min(l1, l2,
l3))]
                    d12 = index1[Label.index(min(l1, l2,
l3))]
                    if d12 == L3 and max(l1, l2, l3) ==
l2:
                        d13 = L2
                    elif d12 == L3 and max(l1, l2, l3) ==
l3:
                        d13 = L1

                    elif d12 == L2 and max(l1, l2, l3) ==
l1:
                        d13 = L1
                    elif d12 == L2 and max(l1, l2, l3) ==
l2:
                        d13 = L3
                    elif d12 == L1 and max(l1, l2, l3) ==
l1:
                        d13 = L2
                    elif d12 == L1 and max(l1, l2, l3) ==
l3:
                        d13 = L3
                    X.remove(max(X))
                    X.remove(min(X))
                    Label1 = max(l1, l2, l3)
                    Label2 = X[0]
                    Label3 = min(l1, l2, l3)

                # 2nd condition
                elif l1 > l2 == l3:
                    Label1 = l1
                    if L2 > L1:
                        b3 = Med3
                        d13 = L1
                        d12 = L2
                        Label2 = l2
                        Label3 = l3
                    else:
                        b3 = Med2
                        d13 = L2
                        d12 = L1
                        Label2 = l3
                        Label3 = l2

                elif l2 > l1 == l3:
                    Label1 = l2
                    if L3 > L2:
                        b3 = Med1
                        d13 = L2
                        d12 = L3
                        Label2 = l3
```

```
            Label3 = l1
        else:
            b3 = Med3
            d13 = L3
            d12 = L2
            Label2 = l1
            Label3 = l3

    elif l3 > l1 == l2:
        Label1 = l3
        if L1 > L3:
            b3 = Med2
            d13 = L3
            d12 = L1
            Label2 = l1
            Label3 = l2
        else:
            b3 = Med1
            d13 = L1
            d12 = L3
            Label2 = l2
            Label3 = l1
    # 3rd condition
    elif l1 == l2 > l3:
        b3 = Med3
        Label3 = l3
        if L1 > L3:
            d13 = L1
            d12 = L2
            Label1 = l1
            Label2 = l2
        else:
            d13 = L3
            d12 = L2
            Label1 = l2
            Label2 = l1

    elif l1 == l3 > l2:
        Label3 = l2
        b3 = Med2
        if L2 > L3:
            d13 = L2
            d12 = L1
            Label1 = l1
            Label2 = l3
        else:
            d13 = L3
            d12 = L1
            Label1 = l3
            Label2 = l1
    elif l2 == l3 > l1:
        Label3 = l1
        b3 = Med1
        if L2 > L1:
```

```
                    d13 = L2
                    d12 = L3
                    Label1 = l2
                    Label2 = l3
                else:
                    d13 = L1
                    d12 = L3
                    Label1 = l3
                    Label2 = l2

            # 4th condition
            if l1 == l2 == l3:
                if L2 >= max(L1, L2, L3):
                    b3 = Med3
                    d13 = L1
                    d12 = L2
                    Label1 = l1
                    Label2 = l2
                    Label3 = l3
                if L1 >= max(L1, L2, L3):
                    b3 = Med2
                    d13 = L2
                    d12 = L1
                    Label1 = l1
                    Label2 = l3
                    Label3 = l2

                if L3 >= max(L1, L2, L3):
                    # b3=Med3
                    # d13 =L1
                    # d12 =L2
                    # Corrected
                    b3 = Med1
                    d13 = L1
                    d12 = L3
                    Label1 = l3
                    Label2 = l2
                    Label3 = l1

            a = (d13 ** 2 - (d12 / 2) ** 2 - b3 ** 2)
            b = (2 * (d12 / 2) * b3)
            Theta1 = (math.acos(a / b)) * (180 /
math.pi)

            if Theta1 <= 90:
                Theta = Theta1
            else:
                Theta = abs(180 - Theta1)
            maxDist = max(L1, L2, L3)
            ClassT1 = thetaClass_(Theta)
            ClassL1 = dist12Class_(maxDist)
            key3D = dLen * dTheta * (numOfLabels **
2) * (int(Label1) - 1) + \
```

```
                                        dLen * dTheta * (numOfLabels) *
(int(Label2) - 1) + \
                                        dLen * dTheta * (int(Label3) - 1)
+ \
                                        dTheta * (ClassL1 - 1) + \
                                        (ClassT1 - 1)

                                if key3D in keyDict3D:
                                    keyDict3D[key3D] += 1
                                else:
                                    keyDict3D[key3D] = 1

                                outputFile1.write("{}_{}_{}_{}
".format(drug_Name, Chain_Name, drug_Id, Atom[i]))
                                outputFile1.write("{}_{}_{}_{}
".format(drug_Name, Chain_Name, drug_Id, Atom[j]))
                                outputFile1.write("{}_{}_{}_{}
".format(drug_Name, Chain_Name, drug_Id, Atom[k]))
                                outputFile1.write(" {:.2f}  {:.2f}
{:.2f} ".format(L1, L2, L3))
                                outputFile1.write(
                                    "  {:.2f},{:.2f},{:.2f}
{:.2f},{:.2f},{:.2f}  ".format(xCoord[i], yCoord[i],

zCoord[i], xCoord[j],

yCoord[j],

zCoord[j]))

                                outputFile1.write(" {:.2f},{:.2f},{:.2f}
".format(xCoord[k], yCoord[k], zCoord[k]))
                                outputFile1.write(
                                    "{:.2f}   {:.2f}   {:.2f}
{:.0f}\n".format(Theta, maxDist, b3, key3D))
                                Total_key += 1

                    for value_ in keyDict3D:
                        outputFile2.writelines([str(value_), '\t',
str(keyDict3D[value_]), '\n'])

                    Key_Dict_Total += (keyDict3D,)

DataFrame_Index.append(f'{PDB_ID}_{Chain_Name}_{drug_Name}_{drug_Id}_{X1_
end}')
                    Group_Information.append(Group_)
                    outputFile3.write(

f'{PDB_ID}_{Chain_Name}_{drug_Name}_{drug_Id}_{X1_end}\t{counter2}\t\t{To
tal_key}\t\t{len(keyDict3D)}\n')

                    outputFile1.close()
                    outputFile2.close()
```

```
outputFile3.close()
df = pd.DataFrame(Key_Dict_Total, index=Group_Information)
df = df.rename_axis('group')
df = df.fillna(0)
df = df.astype('int')
df.insert(0, 'protein', DataFrame_Index)
df.to_csv(f"{Output_Folder_Path}/feature_map_with_header.csv",
header=True, index=True)

df_Group = pd.DataFrame(columns=['group'], index=DataFrame_Index)
df_Group = df_Group.rename_axis('protein')
df_Group['group'] = Group_Information
df_Group.to_csv(f"{Output_Folder_Path}/sample_details_drug.csv",
header=True, index=True)

df_Clustering = pd.DataFrame(Key_Dict_Total, index=DataFrame_Index)
df_Clustering = df_Clustering.fillna(0)
df_Clustering = df_Clustering.astype('int')

first_column = list(df_Clustering.iloc[:, 0])
DataFrame_Index_2 = []
for i in range(len(df_Clustering)):
    DataFrame_Index_2.append(DataFrame_Index[i] + ";" +
str(int(first_column[i])))
df_Clustering.iloc[:, 0] = DataFrame_Index_2
df_Clustering.to_csv(f"{Output_Folder_Path}/localFeatureVect_theta29_dist
35_NoFeatureSelection_keyCombine0.csv",
                     header=False, index=False)
print('completed Successfully')
```

(iii) TSR key generation code for tryptophan using all atoms except hydrogen atoms

#Python code: to calculate “key files” and “triplet file” for tryptophan 3D structures
#Input files: (i) drug_atom_lexical_txt.csv; (ii) sample_details_psi_ab_mix1.csv; (iii) pdb
#Output files: “key files” and “triplet files” will be generated for each tryptophan reside

```
# Program to calculate the amino acid triplets and key Frequency
# Author: Tarikul Islam Milon


import csv
import math
import Bio.PDB
from Bio.PDB import PDBParser
import pandas as pd
import os

dTheta = 29
dLen = 58
numOfLabels = 112

# AminoAcidName=input('Enter the Amino Acid Name:')
#aa_list = ['LYS', 'HIS', 'GLU', 'ARG', 'VAL', 'SER', 'PRO', 'PHE',
'MET', 'LEU', 'ILE', 'GLY', 'CYS', 'ASP', 'ALA',
           #'TRP', 'GLN', 'ASN', 'TYR', 'THR', 'TPO', 'SEP', 'PTR']
aa_list =['TRP']
Incomplete_residue = {'LYS': 84, 'HIS': 120, 'GLU': 84, 'ARG': 165,
'VAL': 35, 'SER': 20, 'PRO': 35, 'PHE': 165,
                      'MET': 56, 'LEU': 56, 'ILE': 56, 'GLY': 4, 'CYS':
20, 'ASP': 56, 'ALA': 10, 'TRP': 364, 'GLN': 84,
                      'ASN': 56, 'TYR': 220, 'THR': 35, 'TPO': 165,
'SEP': 120, 'PTR': 560}
# We can chnage amino acid
df = pd.read_csv('sample_details_psi_ab_mix1.csv')
PDB_list = df['protein'].to_list()
Chain = df['chain'].to_list()

atomSeq = {}
atomSeqNumber = open("drug_atom_lexical_txt.csv", 'r')
reader2 = csv.reader(atomSeqNumber)
next(reader2)
for row in reader2:
    atomSeq[row[2]] = row[1]
atomSeqNumber.close()


# Theta Bin for 3D

def thetaClass_(Theta):
    # classT=0
    if Theta >= 0 and Theta < 12.11:
        classT = 1
    elif Theta >= 12.11 and Theta < 17.32:
        classT = 2
    elif Theta >= 17.32 and Theta < 21.53:
```

```
        classT = 3
    elif Theta >= 21.53 and Theta < 25.21:
        classT = 4
    elif Theta >= 25.21 and Theta < 28.54:
        classT = 5
    elif Theta >= 28.54 and Theta < 31.64:
        classT = 6
    elif Theta >= 31.64 and Theta < 34.55:
        classT = 7
    elif Theta >= 34.55 and Theta < 37.34:
        classT = 8
    elif Theta >= 37.34 and Theta < 40.03:
        classT = 9
    elif Theta >= 40.03 and Theta < 42.64:
        classT = 10
    elif Theta >= 42.64 and Theta < 45.17:
        classT = 11
    elif Theta >= 45.17 and Theta < 47.64:
        classT = 12
    elif Theta >= 47.64 and Theta < 50.05:
        classT = 13
    elif Theta >= 50.05 and Theta < 52.43:
        classT = 14
    elif Theta >= 52.43 and Theta < 54.77:
        classT = 15
    elif Theta >= 54.77 and Theta < 57.08:
        classT = 16
    elif Theta >= 57.08 and Theta < 59.38:
        classT = 17
    elif Theta >= 59.38 and Theta < 61.64:
        classT = 18
    elif Theta >= 61.64 and Theta < 63.87:
        classT = 19
    elif Theta >= 63.87 and Theta < 66.09:
        classT = 20
    elif Theta >= 66.09 and Theta < 68.30:
        classT = 21
    elif Theta >= 68.30 and Theta < 70.5:
        classT = 22
    elif Theta >= 70.5 and Theta < 72.69:
        classT = 23
    elif Theta >= 72.69 and Theta < 79.2:
        classT = 24
    elif Theta >= 79.2 and Theta < 81.36:
        classT = 25
    elif Theta >= 81.36 and Theta < 83.51:
        classT = 26
    elif Theta >= 83.51 and Theta < 85.67:
        classT = 27
    elif Theta >= 85.67 and Theta < 87.80:
        classT = 28
    elif Theta >= 87.80 and Theta <= 90.00:
        classT = 29
    return classT
```

```
# MaxDist bin for 3D
def dist12Class_(dist12):
    if dist12 >= 0 and dist12 < 1:
        classL = 1
    elif dist12 >= 1 and dist12 < 2:
        classL = 2
    elif dist12 >= 2 and dist12 < 3:
        classL = 3
    elif dist12 >= 3 and dist12 < 4:
        classL = 4
    elif dist12 >= 4 and dist12 < 5:
        classL = 5
    elif dist12 >= 5 and dist12 < 6:
        classL = 6
    elif dist12 >= 6 and dist12 < 7:
        classL = 7
    elif dist12 >= 7 and dist12 < 8:
        classL = 8
    elif dist12 >= 8 and dist12 < 9:
        classL = 9
    elif dist12 >= 9 and dist12 < 10:
        classL = 10
    elif dist12 >= 10 and dist12 < 11:
        classL = 11
    elif dist12 >= 11 and dist12 < 12:
        classL = 12
    elif dist12 >= 12 and dist12 < 13:
        classL = 13
    elif dist12 >= 13 and dist12 < 14:
        classL = 14
    elif dist12 >= 14 and dist12 < 15:
        classL = 15
    elif dist12 >= 15 and dist12 < 16:
        classL = 16
    elif dist12 >= 16 and dist12 < 17:
        classL = 17
    elif dist12 >= 17 and dist12 < 18:
        classL = 18
    elif dist12 >= 18 and dist12 < 19:
        classL = 19
    elif dist12 >= 19 and dist12 < 20:
        classL = 20
    elif dist12 >= 20 and dist12 < 21:
        classL = 21
    elif dist12 >= 21 and dist12 < 22:
        classL = 22
    elif dist12 >= 22 and dist12 < 23:
        classL = 23
    elif dist12 >= 23 and dist12 < 24:
        classL = 24
    elif dist12 >= 24 and dist12 < 25:
        classL = 25
```

```
elif dist12 >= 25 and dist12 < 26:
    classL = 26
elif dist12 >= 26 and dist12 < 27:
    classL = 27
elif dist12 >= 27 and dist12 < 28:
    classL = 28
elif dist12 >= 28 and dist12 < 29:
    classL = 29
elif dist12 >= 29 and dist12 < 30:
    classL = 30
elif dist12 >= 30 and dist12 < 31:
    classL = 31
elif dist12 >= 31 and dist12 < 32:
    classL = 32
elif dist12 >= 32 and dist12 < 33:
    classL = 33
elif dist12 >= 33 and dist12 < 34:
    classL = 34
elif dist12 >= 34 and dist12 < 35:
    classL = 35
elif dist12 >= 35 and dist12 < 36:
    classL = 36
elif dist12 >= 36 and dist12 < 37:
    classL = 37
elif dist12 >= 37 and dist12 < 38:
    classL = 38
elif dist12 >= 38 and dist12 < 39:
    classL = 39
elif dist12 >= 39 and dist12 < 40:
    classL = 40
elif dist12 >= 40 and dist12 < 41:
    classL = 41
elif dist12 >= 41 and dist12 < 42:
    classL = 42
elif dist12 >= 42 and dist12 < 43:
    classL = 43
elif dist12 >= 43 and dist12 < 44:
    classL = 44
elif dist12 >= 44 and dist12 < 45:
    classL = 45
elif dist12 >= 45 and dist12 < 46:
    classL = 46
elif dist12 >= 46 and dist12 < 47:
    classL = 47
elif dist12 >= 47 and dist12 < 48:
    classL = 48
elif dist12 >= 48 and dist12 < 49:
    classL = 49
elif dist12 >= 49 and dist12 < 50:
    classL = 50
elif dist12 >= 50 and dist12 < 51:
    classL = 51
elif dist12 >= 51 and dist12 < 52:
    classL = 52
```

```
    elif dist12 >= 52 and dist12 < 53:
        classL = 53
    elif dist12 >= 53 and dist12 < 54:
        classL = 54
    elif dist12 >= 54 and dist12 < 55:
        classL = 55
    elif dist12 >= 55 and dist12 < 56:
        classL = 56
    elif dist12 >= 56 and dist12 < 57:
        classL = 57
    elif dist12 >= 57 and dist12 < 1000:
        classL = 58

    return classL


def calDist(x1, y1, z1, x2, y2, z2):
    return math.sqrt((x2 - x1) ** 2 + (y2 - y1) ** 2 + (z2 - z1) ** 2)


for AminoAcidName in aa_list:
    duplicate = []
    Key_Dict_Total = ()
    DataFrame_Index = []
    Group_Information = []

    Output_Folder_Path =
f'/ddnB/work/wxx6941/TSR/code/code/psi_revision/9pdb/output_trp_tsr_a_b'
    outputFile3 =
open(f'{Output_Folder_Path}/Incomplete_Residue{AminoAcidName}.txt', 'w')
    #
outputFile4=open(f'{Output_Folder_Path}/sample_details_{AminoAcidName}.cs
v', 'w')
    for i in range(len(PDB_list)):
        PDB_ID = PDB_list[i]
        Chain_Name = Chain[i]
        rm_duplicate = f'{PDB_ID}_{Chain_Name}'
        if rm_duplicate not in duplicate:
            duplicate.append(rm_duplicate)

            PDB_File_Path =
"/ddnB/work/wxx6941/TSR/code/code/psi_revision/9pdb/PDB_datadir_HRemoved/
{}.pdb".format(
                PDB_ID)
            p = Bio.PDB.PDBParser()
            Structure = p.get_structure('PrimaryStructureChain',
PDB_File_Path)
            model = Structure[0]
            for chain in model:
                if chain.id == Chain_Name:
                    for residue in chain:
                        if str(residue)[9:12] == AminoAcidName.upper():
                            numeric_filter = filter(str.isdigit,
str(residue.id)[6:10])
```

```
                            Res_Id = "".join(numeric_filter)
                            for atom0 in residue:
                                Coord_end = atom0.get_vector()
                                X1_end = Coord_end[0]

                            X1_end = '{:.2f}'.format(X1_end)

                            outputFile1 = open(

f'{Output_Folder_Path}/{PDB_ID}_{Chain_Name.upper()}_{AminoAcidName}_{Res
_Id}_{X1_end}.triplets_theta29_dist17',
                                'w')
                            outputFile2 = open(

f'{Output_Folder_Path}/key_{PDB_ID}_{Chain_Name.upper()}_{AminoAcidName}_
{Res_Id}_{X1_end}.keys_theta29_dist17',
                                'w')
                            # header
                            outputFile1.writelines(
                                'Residue1   Residue2   Residue3   Edge1
Edge2  Edge3\t   Coor_R1\t           Coor_R2\t
CoorR3\tTheta\tmax_dist\td_3\tkey3D\n')
                            outputFile2.writelines('key\t\tfreq\n')

                            keyDict3D = {}
                            xCoord = {}
                            yCoord = {}
                            zCoord = {}
                            Atom = {}
                            counter2 = 0
                            for atom1 in residue:
                                atomCoord = atom1.get_vector()

                                Atom[counter2] = atom1.get_name()
                                xCoord[counter2] = atomCoord[0]
                                yCoord[counter2] = atomCoord[1]
                                zCoord[counter2] = atomCoord[2]

                                counter2 += 1
                            for i in range(len(xCoord)):
                                for j in range(i + 1, len(yCoord)):
                                    for k in range(j + 1, len(zCoord)):
                                        L1 = calDist(xCoord[i],
yCoord[i], zCoord[i], xCoord[j], yCoord[j], zCoord[j])
                                        L2 = calDist(xCoord[j],
yCoord[j], zCoord[j], xCoord[k], yCoord[k], zCoord[k])
                                        L3 = calDist(xCoord[i],
yCoord[i], zCoord[i], xCoord[k], yCoord[k], zCoord[k])

                                        l1 = atomSeq[Atom[j]]
                                        l2 = atomSeq[Atom[k]]
                                        l3 = atomSeq[Atom[i]]
```

```
Med1 = (1 / 2) * math.sqrt(2 * (L1 ** 2) + 2 * (L2 ** 2) - L3 ** 2)

Med2 = (1 / 2) * math.sqrt(2 * (L2 ** 2) + 2 * (L3 ** 2) - L1 ** 2)

Med3 = (1 / 2) * math.sqrt(2 * (L3 ** 2) + 2 * (L1 ** 2) - L2 ** 2)

Median = [Med1, Med2, Med3]
Label = [l1, l2, l3]
index1 = [L3, L1, L2]

# 1st Condition
if l1 != l2 != l3:
    X = [l1, l2, l3]
    b3 = Median[Label.index(min(l1, l2, l3))]

    d12 = index1[Label.index(min(l1, l2, l3))]

    if d12 == L3 and max(l1, l2, l3) == l2:

        d13 = L2
    elif d12 == L3 and max(l1, l2, l3) == l3:

        d13 = L1

    elif d12 == L2 and max(l1, l2, l3) == l1:

        d13 = L1
    elif d12 == L2 and max(l1, l2, l3) == l2:

        d13 = L3
    elif d12 == L1 and max(l1, l2, l3) == l1:

        d13 = L2
    elif d12 == L1 and max(l1, l2, l3) == l3:

        d13 = L3
    X.remove(max(X))
    X.remove(min(X))
    Label1 = max(l1, l2, l3)
    Label2 = X[0]
    Label3 = min(l1, l2, l3)

# 2nd condition
elif l1 > l2 == l3:
    Label1 = l1
    if L2 > L1:
        b3 = Med3
        d13 = L1
        d12 = L2
        Label2 = l2
        Label3 = l3
    else:
        b3 = Med2
        d13 = L2
```

```
                    d12 = L1
                    Label2 = l3
                    Label3 = l2

            elif l2 > l1 == l3:
                Label1 = l2
                if L3 > L2:
                    b3 = Med1
                    d13 = L2
                    d12 = L3
                    Label2 = l3
                    Label3 = l1
                else:
                    b3 = Med3
                    d13 = L3
                    d12 = L2
                    Label2 = l1
                    Label3 = l3

            elif l3 > l1 == l2:
                Label1 = l3
                if L1 > L3:
                    b3 = Med2
                    d13 = L3
                    d12 = L1
                    Label2 = l1
                    Label3 = l2
                else:
                    b3 = Med1
                    d13 = L1
                    d12 = L3
                    Label2 = l2
                    Label3 = l1
            # 3rd condition
            elif l1 == l2 > l3:
                b3 = Med3
                Label3 = l3
                if L1 > L3:
                    d13 = L1
                    d12 = L2
                    Label1 = l1
                    Label2 = l2
                else:
                    d13 = L3
                    d12 = L2
                    Label1 = l2
                    Label2 = l1

            elif l1 == l3 > l2:
                Label3 = l2
                b3 = Med2
                if L2 > L3:
                    d13 = L2
                    d12 = L1
```

```
            Label1 = l1
            Label2 = l3
        else:
            d13 = L3
            d12 = L1
            Label1 = l3
            Label2 = l1
    elif l2 == l3 > l1:
        Label3 = l1
        b3 = Med1
        if L2 > L1:
            d13 = L2
            d12 = L3
            Label1 = l2
            Label2 = l3
        else:
            d13 = L1
            d12 = L3
            Label1 = l3
            Label2 = l2

    # 4th condition
    if l1 == l2 == l3:
        if L2 >= max(L1, L2, L3):
            b3 = Med3
            d13 = L1
            d12 = L2
            Label1 = l1
            Label2 = l2
            Label3 = l3
        if L1 >= max(L1, L2, L3):
            b3 = Med2
            d13 = L2
            d12 = L1
            Label1 = l1
            Label2 = l3
            Label3 = l2

        if L3 >= max(L1, L2, L3):
            # b3=Med3
            # d13 =L1
            # d12 =L2
            # Corrected
            b3 = Med1
            d13 = L1
            d12 = L3
            Label1 = l3
            Label2 = l2
            Label3 = l1

    a = (d13 ** 2 - (d12 / 2) ** 2 -
b3 ** 2)
    b = (2 * (d12 / 2) * b3)
```

```
                                        Theta1 = (math.acos(a / b)) *
(180 / math.pi)

                                        if Theta1 <= 90:
                                            Theta = Theta1
                                        else:
                                            Theta = abs(180 - Theta1)
                                        maxDist = max(L1, L2, L3)
                                        ClassT1 = thetaClass_(Theta)
                                        ClassL1 = dist12Class_(maxDist)
                                        key3D = dLen * dTheta *
(numOfLabels ** 2) * (int(Label1) - 1) + \
                                                dLen * dTheta *
(numOfLabels) * (int(Label2) - 1) + \
                                                dLen * dTheta *
(int(Label3) - 1) + \
                                                dTheta * (ClassL1 - 1) +
\
                                                (ClassT1 - 1)

                                        if key3D in keyDict3D:
                                            keyDict3D[key3D] += 1
                                        else:
                                            keyDict3D[key3D] = 1

                                        outputFile1.write(
                                            "{}_{}_{}_{}
".format(AminoAcidName, Chain_Name, Res_Id, Atom[i]))
                                        outputFile1.write(
                                            "{}_{}_{}_{}
".format(AminoAcidName, Chain_Name, Res_Id, Atom[j]))
                                        outputFile1.write(
                                            "{}_{}_{}_{}
".format(AminoAcidName, Chain_Name, Res_Id, Atom[k]))
                                        outputFile1.write(" {:.2f}
{:.2f}  {:.2f} ".format(L1, L2, L3))
                                        outputFile1.write(
                                            "  {:.2f},{:.2f},{:.2f}
{:.2f},{:.2f},{:.2f}  ".format(xCoord[i],

yCoord[i],

zCoord[i],

xCoord[j],

yCoord[j],

zCoord[j]))

                                        outputFile1.write(
                                            " {:.2f},{:.2f},{:.2f}
".format(xCoord[k], yCoord[k], zCoord[k]))
                                        outputFile1.write(
```

```
                                        "{:.2f}   {:.2f}   {:.2f}
{:.0f}\n".format(Theta, maxDist, b3, key3D))

                            for value_ in keyDict3D:
                                outputFile2.writelines([str(value_),
'\t', str(keyDict3D[value_]), '\n'])
                            if sum(keyDict3D.values()) ==
Incomplete_residue[AminoAcidName]:
                                Key_Dict_Total += (keyDict3D,)

DataFrame_Index.append(f'{PDB_ID}_{Chain_Name}_{AminoAcidName}_{Res_Id}_{
X1_end}')
                                Group_Information.append(AminoAcidName)

                            else:

outputFile3.writelines(f'{PDB_ID}_{Chain_Name}_{AminoAcidName}_{Res_Id}_{
X1_end}\n')
                            outputFile1.close()
                            outputFile2.close()

    df = pd.DataFrame(Key_Dict_Total, index=Group_Information)
    df = df.rename_axis('group')
    df = df.fillna(0)
    df = df.astype('int')
    df.insert(0, 'protein', DataFrame_Index)
    df.to_csv(f"{Output_Folder_Path}/feature_map_with_header.csv",
header=True, index=True)

    df_Group = pd.DataFrame(columns=['group'], index=DataFrame_Index)
    df_Group = df_Group.rename_axis('protein')
    df_Group['group'] = Group_Information

df_Group.to_csv(f"{Output_Folder_Path}/sample_details_{AminoAcidName}.csv
", header=True, index=True)

    df_Group_2 = pd.DataFrame(columns=['group'], index=DataFrame_Index)
    df_Group_2 = df_Group_2.rename_axis('protein')
    df_Group_2['group'] = Group_Information

df_Group_2.to_csv(f"{Output_Folder_Path}/sample_details_2_{AminoAcidName}
.csv", header=True, index=True)

    df_Clustering = pd.DataFrame(Key_Dict_Total, index=DataFrame_Index)
    df_Clustering = df_Clustering.fillna(0)
    df_Clustering = df_Clustering.astype('int')

    first_column = list(df_Clustering.iloc[:, 0])
    DataFrame_Index_2 = []
    for i in range(len(df_Clustering)):
        DataFrame_Index_2.append(DataFrame_Index[i] + ";" +
str(int(first_column[i])))
    df_Clustering.iloc[:, 0] = DataFrame_Index_2
```

```
df_Clustering.to_csv(f"{Output_Folder_Path}/localFeatureVect_theta29_dist
35_NoFeatureSelection_keyCombine0.csv",
                         header=False, index=False)
    outputFile3.close()

print('completed Successfully')
```

(iv) USR code for proteins using C alpha atoms

#Python code: Calculate structure similarity of protein using CA atom using USR

```
#program to calculate similarity between molecules


import csv
import math
import Bio.PDB
from Bio.PDB import PDBParser
import pandas as pd
import os
from scipy.stats import skew
import numpy as np
from itertools import combinations

df=pd.read_csv('sample_details_psi_ab_mix1.csv')
PDB_list = df['protein'].to_list()
Chain = df['chain'].to_list()
#Drug_name = df['drug_name'].to_list()
#Drug_id = df['drug_id'].to_list()
#Group = df['group'].to_list()


def calDist(x1, y1, z1, x2, y2, z2):
    return math.sqrt((x2 - x1) ** 2 + (y2 - y1) ** 2 + (z2 - z1) ** 2)

def Cal_Centroid(chain):
    SumX = 0
    SumY = 0
    SumZ = 0
    counter = 0
    for residue in chain:
        for atom1 in residue:
            if atom1.get_name()=='CA':
               atomCoord = atom1.get_vector()
               SumX += atomCoord[0]
               SumY += atomCoord[1]
               SumZ += atomCoord[2]

               counter += 1

    Centroid_X_ = SumX / counter
    Centroid_Y_ = SumY / counter
    Centroid_Z_ = SumZ / counter
    return Centroid_X_,Centroid_Y_,Centroid_Z_
def Cal_NA_Centroid(*args):
    Cen_X=args[0]
    Cen_Y=args[1]
    Cen_Z=args[2]
    dist_dict={}
    for residue in chain:
        for atom1 in residue:
            if atom1.get_name()=='CA':
               atomCoord = atom1.get_vector()
```

```
                dist=calDist(Cen_X, Cen_Y, Cen_Z, atomCoord[0],
atomCoord[1], atomCoord[2])

dist_dict[dist]=f'{atomCoord[0]}_{atomCoord[1]}_{atomCoord[2]}'

    #print(dist_dict)
    #print(min(dist_dict.values()))
    NA_Coord=dist_dict[min(dist_dict.keys())].split("_")
    DA_Coord = dist_dict[max(dist_dict.keys())].split("_")
    NA_Centroid_X_=NA_Coord[0]
    NA_Centroid_Y_ = NA_Coord[1]
    NA_Centroid_Z_ = NA_Coord[2]

    DA_Centroid_X_=DA_Coord[0]
    DA_Centroid_Y_ = DA_Coord[1]
    DA_Centroid_Z_ = DA_Coord[2]

    CM_=np.mean([*dist_dict.keys()])
    CV_=np.var([*dist_dict.keys()])
    CS_=skew([*dist_dict.keys()], axis=0, bias=True)


    return
NA_Centroid_X_,NA_Centroid_Y_,NA_Centroid_Z_,DA_Centroid_X_,DA_Centroid_Y
_,DA_Centroid_Z_,CM_,CV_,CS_


    #print(dist_dict[min(dist_dict.values())])
def Cal_DPA_Centroid(*args):
    Cen_X=float(args[0])
    Cen_Y=float(args[1])
    Cen_Z=float(args[2])
    dist_dict={}
    for residue in chain:
        for atom1 in residue:
            if atom1.get_name()=='CA':
                atomCoord = atom1.get_vector()
                dist=calDist(Cen_X, Cen_Y, Cen_Z, atomCoord[0],
atomCoord[1], atomCoord[2])

dist_dict[dist]=f'{atomCoord[0]}_{atomCoord[1]}_{atomCoord[2]}'

    #print(dist_dict)
    #print(min(dist_dict.values()))
    DPA_Coord = dist_dict[max(dist_dict.keys())].split("_")

    DPA_Centroid_X_=DPA_Coord[0]
    DPA_Centroid_Y_ = DPA_Coord[1]
    DPA_Centroid_Z_ = DPA_Coord[2]

    DAM_=np.mean([*dist_dict.keys()])
    DAV_=np.var([*dist_dict.keys()])
    DAS_=skew([*dist_dict.keys()], axis=0, bias=True)
    return DPA_Centroid_X_,DPA_Centroid_Y_,DPA_Centroid_Z_,DAM_,DAV_,DAS_
```

```
def Cal_MVS(*args):
    Cen_X=int(float(args[0]))
    Cen_Y=int(float(args[1]))
    Cen_Z=int(float(args[2]))
    dist_dict={}
    for residue in chain:
        for atom1 in residue:
            if atom1.get_name()=='CA':
                atomCoord = atom1.get_vector()
                dist=calDist(Cen_X, Cen_Y, Cen_Z, atomCoord[0],
atomCoord[1], atomCoord[2])

dist_dict[dist]=f'{atomCoord[0]}_{atomCoord[1]}_{atomCoord[2]}'

    #print(dist_dict)
    #print(min(dist_dict.values()))

    M=np.mean([*dist_dict.keys()])
    V=np.var([*dist_dict.keys()])
    S=skew([*dist_dict.keys()], axis=0, bias=True)
    return M,V,S


Dict_drug={}
Output_Folder_Path="/ddnB/work/wxx6941/TSR/code/code/psi_revision/9pdb/ou
tput_ca_a_b_usr"
OutputFile=open(f'{Output_Folder_Path}/Similarity_drugs.txt','w')
for i in range(len(PDB_list)):
    PDB_ID=PDB_list[i]
    Chain_Name = Chain[i]
    #drug_Name = Drug_name[i]
    #drug_Id = Drug_id[i]
    #group=Group[i]

    #print(PDB_ID,Chain_Name,drug_Name,drug_Id)
    PDB_File_Path =
"/ddnB/work/wxx6941/TSR/code/code/psi_revision/9pdb/PDB_datadir_HRemoved/
{}.pdb".format(PDB_ID)
    p = Bio.PDB.PDBParser()
    Structure = p.get_structure('PrimaryStructureChain', PDB_File_Path)

    model = Structure[0]
    for chain in model:
        if chain.id == Chain_Name:
            PDB_chain=f'{PDB_ID}_{Chain_Name}'
            Centroid_X,Centroid_Y,Centroid_Z=Cal_Centroid(chain)

NA_Centroid_X,NA_Centroid_Y,NA_Centroid_Z,DA_Centroid_X,DA_Centroid_Y,DA_
Centroid_Z,CM,CV,CS=Cal_NA_Centroid(Centroid_X,Centroid_Y,Centroid_Z,chai
n)
```

```
DPA_Centroid_X,DPA_Centroid_Y,DPA_Centroid_Z,DAM,DAV,DAS=Cal_DPA_Centroid
(DA_Centroid_X,DA_Centroid_Y,DA_Centroid_Z,chain)

            NAM, NAV, NAS =
Cal_MVS(NA_Centroid_X,NA_Centroid_Y,NA_Centroid_Z, chain)
            DPAM, DPAV, DPAS =
Cal_MVS(DPA_Centroid_X,DPA_Centroid_Y,DPA_Centroid_Z, chain)

Dict_drug[PDB_chain]=f'{CM}_{CV}_{CS}_{NAM}_{NAV}_{NAS}_{DAM}_{DAV}_{DAS}
_{DPAM}_{DPAV}_{DPAS}'


print(Dict_drug)
comb_drug = combinations([*Dict_drug], 2)
for i in comb_drug:
    Drug1_moments=Dict_drug[i[0]].split("_")
    Drug2_moments = Dict_drug[i[1]].split("_")
    print(Drug1_moments)
    print(Drug2_moments)
    sum=0
    for k in range(len(Drug1_moments)):
        diff=abs(float(Drug1_moments[k])-float(Drug2_moments[k]))
        sum+=diff
    similarity=1/(1+(sum/12))
    OutputFile.write(f'{i[0]}\t{i[1]}\t{similarity}')
    OutputFile.write('\n')

OutputFile.close()
print('completed successfully')
```

(v) USR code for pigments using all atoms except hydrogen atoms

#Python code: Calculate the structural similarity for chl using USR

```
#program to calulate similarity between molecules


import csv
import math
import Bio.PDB
from Bio.PDB import PDBParser
import pandas as pd
import os
from scipy.stats import skew
import numpy as np
from itertools import combinations

df=pd.read_csv('sample_details_et_mix5_name_id_atom.csv')
PDB_list = df['protein'].to_list()
Chain = df['chain'].to_list()
Drug_name = df['drug_name'].to_list()
Drug_id = df['drug_id'].to_list()
Group = df['group'].to_list()
Atom_list=df['ligand_atom'].to_list()

def calDist(x1, y1, z1, x2, y2, z2):
    return math.sqrt((x2 - x1) ** 2 + (y2 - y1) ** 2 + (z2 - z1) ** 2)

def Cal_Centroid(res,Atoms):
    SumX = 0
    SumY = 0
    SumZ = 0
    counter = 0
    for atom1 in residue:
        if atom1.get_name() in Atoms:
            atomCoord = atom1.get_vector()
            SumX += atomCoord[0]
            SumY += atomCoord[1]
            SumZ += atomCoord[2]
            counter += 1

    Centroid_X_ = SumX / counter
    Centroid_Y_ = SumY / counter
    Centroid_Z_ = SumZ / counter
    return Centroid_X_,Centroid_Y_,Centroid_Z_
def Cal_NA_Centroid(*args):
    Cen_X=args[0]
    Cen_Y=args[1]
    Cen_Z=args[2]
    dist_dict={}
    for atom1 in residue:
        if atom1.get_name() in atoms:
            atomCoord = atom1.get_vector()
            dist=calDist(Cen_X, Cen_Y, Cen_Z, atomCoord[0], atomCoord[1],
atomCoord[2])
```

```
dist_dict[dist]=f'{atomCoord[0]}_{atomCoord[1]}_{atomCoord[2]}'

    #print(dist_dict)
    #print(min(dist_dict.values()))
    NA_Coord=dist_dict[min(dist_dict.keys())].split("_")
    DA_Coord = dist_dict[max(dist_dict.keys())].split("_")
    NA_Centroid_X_=NA_Coord[0]
    NA_Centroid_Y_ = NA_Coord[1]
    NA_Centroid_Z_ = NA_Coord[2]

    DA_Centroid_X_=DA_Coord[0]
    DA_Centroid_Y_ = DA_Coord[1]
    DA_Centroid_Z_ = DA_Coord[2]

    CM_=np.mean([*dist_dict.keys()])
    CV_=np.var([*dist_dict.keys()])
    CS_=skew([*dist_dict.keys()], axis=0, bias=True)


    return
NA_Centroid_X_,NA_Centroid_Y_,NA_Centroid_Z_,DA_Centroid_X_,DA_Centroid_Y
_,DA_Centroid_Z_,CM_,CV_,CS_


    #print(dist_dict[min(dist_dict.values())])
def Cal_DPA_Centroid(*args):
    Cen_X=float(args[0])
    Cen_Y=float(args[1])
    Cen_Z=float(args[2])
    dist_dict={}
    for atom1 in residue:
        if atom1.get_name() in atoms:
            atomCoord = atom1.get_vector()
            dist=calDist(Cen_X, Cen_Y, Cen_Z, atomCoord[0], atomCoord[1],
atomCoord[2])

dist_dict[dist]=f'{atomCoord[0]}_{atomCoord[1]}_{atomCoord[2]}'

    #print(dist_dict)
    #print(min(dist_dict.values()))
    DPA_Coord = dist_dict[max(dist_dict.keys())].split("_")

    DPA_Centroid_X_=DPA_Coord[0]
    DPA_Centroid_Y_ = DPA_Coord[1]
    DPA_Centroid_Z_ = DPA_Coord[2]

    DAM_=np.mean([*dist_dict.keys()])
    DAV_=np.var([*dist_dict.keys()])
    DAS_=skew([*dist_dict.keys()], axis=0, bias=True)
    return DPA_Centroid_X_,DPA_Centroid_Y_,DPA_Centroid_Z_,DAM_,DAV_,DAS_

def Cal_MVS(*args):
    Cen_X=int(float(args[0]))
```

```
    Cen_Y=int(float(args[1]))
    Cen_Z=int(float(args[2]))
    dist_dict={}
    for atom1 in residue:
        if atom1.get_name() in atoms:
            atomCoord = atom1.get_vector()
            dist=calDist(Cen_X, Cen_Y, Cen_Z, atomCoord[0], atomCoord[1],
atomCoord[2])

dist_dict[dist]=f'{atomCoord[0]}_{atomCoord[1]}_{atomCoord[2]}'

    #print(dist_dict)
    #print(min(dist_dict.values()))

    M=np.mean([*dist_dict.keys()])
    V=np.var([*dist_dict.keys()])
    S=skew([*dist_dict.keys()], axis=0, bias=True)
    return M,V,S


Dict_drug={}
Output_Folder_Path="/ddnB/work/wxx6941/TSR/code/code/psi_revision/5pdb/ou
tput_chl_atom_selection_usr"
OutputFile=open(f'{Output_Folder_Path}/Similarity_drugs.txt','w')
for i in range(len(PDB_list)):
    PDB_ID=PDB_list[i]
    Chain_Name = Chain[i]
    drug_Name = Drug_name[i]
    drug_Id = Drug_id[i]
    group=Group[i]
    atoms=Atom_list[i].split(";")
    Drug=f'{PDB_ID}_{Chain_Name}_{drug_Name}_{drug_Id}_{group}'
    #print(PDB_ID,Chain_Name,drug_Name,drug_Id)
    PDB_File_Path =
"/ddnB/work/wxx6941/TSR/code/code/psi_revision/5pdb/PDB_datadir_HRemoved/
{}.pdb".format(PDB_ID)
    p = Bio.PDB.PDBParser()
    Structure = p.get_structure('PrimaryStructureChain', PDB_File_Path)
    if drug_Name == 'CL0':
        #drug_Name='CL0'
        drug_Id=f'0{drug_Id}'
    #print(drug_Id)
    #print(drug_Name)

    model = Structure[0]
    for chain in model:
        if chain.id == Chain_Name:

            for residue in chain:

                drug_Identifier = str(residue)[17:18].strip()
                resName = str(residue)[9:12].strip()  # residue Name

                numeric_filter = filter(str.isdigit, str(residue.id))
```

```
                Res_Id = "".join(numeric_filter)  # Residue ID


                if drug_Identifier == 'H' and resName == drug_Name and
Res_Id == str(drug_Id):

Centroid_X,Centroid_Y,Centroid_Z=Cal_Centroid(residue,atoms)

NA_Centroid_X,NA_Centroid_Y,NA_Centroid_Z,DA_Centroid_X,DA_Centroid_Y,DA_
Centroid_Z,CM,CV,CS=Cal_NA_Centroid(Centroid_X,Centroid_Y,Centroid_Z,resi
due,atoms)


DPA_Centroid_X,DPA_Centroid_Y,DPA_Centroid_Z,DAM,DAV,DAS=Cal_DPA_Centroid
(DA_Centroid_X,DA_Centroid_Y,DA_Centroid_Z,residue,atoms)

                    NAM, NAV, NAS =
Cal_MVS(NA_Centroid_X,NA_Centroid_Y,NA_Centroid_Z, residue,atoms)
                    DPAM, DPAV, DPAS =
Cal_MVS(DPA_Centroid_X,DPA_Centroid_Y,DPA_Centroid_Z, residue,atoms)

Dict_drug[Drug]=f'{CM}_{CV}_{CS}_{NAM}_{NAV}_{NAS}_{DAM}_{DAV}_{DAS}_{DPA
M}_{DPAV}_{DPAS}'

print(Dict_drug)
comb_drug = combinations([*Dict_drug], 2)
for i in comb_drug:
    Drug1_moments=Dict_drug[i[0]].split("_")
    Drug2_moments = Dict_drug[i[1]].split("_")
    print(Drug1_moments)
    print(Drug2_moments)
    sum=0
    for k in range(len(Drug1_moments)):
        diff=abs(float(Drug1_moments[k])-float(Drug2_moments[k]))
        sum+=diff
    similarity=1/(1+(sum/12))
    OutputFile.write(f'{i[0]}\t{i[1]}\t{similarity}')
    OutputFile.write('\n')

OutputFile.close()
print('completed successfully')
```

(vi) USR code for tryptophan using all atoms except hydrogen atoms

#Python code: Calculate structure similarity of tryptophan using USR

```
#program to calulate similarity between molecules


import csv
import math
import Bio.PDB
from Bio.PDB import PDBParser
import pandas as pd
import os
from scipy.stats import skew
import numpy as np
from itertools import combinations

df=pd.read_csv('sample_details_psi_ab_mix1.csv')
PDB_list = df['protein'].to_list()
Chain = df['chain'].to_list()
#Drug_name = df['drug_name'].to_list()
#Drug_id = df['drug_id'].to_list()
#Group = df['group'].to_list()


def calDist(x1, y1, z1, x2, y2, z2):
    return math.sqrt((x2 - x1) ** 2 + (y2 - y1) ** 2 + (z2 - z1) ** 2)

def Cal_Centroid(res):
    SumX = 0
    SumY = 0
    SumZ = 0
    counter = 0
    for atom1 in residue:
        atomCoord = atom1.get_vector()
        SumX += atomCoord[0]
        SumY += atomCoord[1]
        SumZ += atomCoord[2]
        counter += 1
    Centroid_X_ = SumX / counter
    Centroid_Y_ = SumY / counter
    Centroid_Z_ = SumZ / counter
    return Centroid_X_,Centroid_Y_,Centroid_Z_
def Cal_NA_Centroid(*args):
    Cen_X=args[0]
    Cen_Y=args[1]
    Cen_Z=args[2]
    dist_dict={}
    for atom1 in residue:
        atomCoord = atom1.get_vector()
        dist=calDist(Cen_X, Cen_Y, Cen_Z, atomCoord[0], atomCoord[1],
atomCoord[2])
        dist_dict[dist]=f'{atomCoord[0]}_{atomCoord[1]}_{atomCoord[2]}'
    #print(dist_dict)
    #print(min(dist_dict.values()))
```

```
    NA_Coord=dist_dict[min(dist_dict.keys())].split("_")
    DA_Coord = dist_dict[max(dist_dict.keys())].split("_")
    NA_Centroid_X_=NA_Coord[0]
    NA_Centroid_Y_ = NA_Coord[1]
    NA_Centroid_Z_ = NA_Coord[2]

    DA_Centroid_X_=DA_Coord[0]
    DA_Centroid_Y_ = DA_Coord[1]
    DA_Centroid_Z_ = DA_Coord[2]

    CM_=np.mean([*dist_dict.keys()])
    CV_=np.var([*dist_dict.keys()])
    CS_=skew([*dist_dict.keys()], axis=0, bias=True)


    return
NA_Centroid_X_,NA_Centroid_Y_,NA_Centroid_Z_,DA_Centroid_X_,DA_Centroid_Y
_,DA_Centroid_Z_,CM_,CV_,CS_


    #print(dist_dict[min(dist_dict.values())])
def Cal_DPA_Centroid(*args):
    Cen_X=float(args[0])
    Cen_Y=float(args[1])
    Cen_Z=float(args[2])
    dist_dict={}
    for atom1 in residue:
        atomCoord = atom1.get_vector()
        dist=calDist(Cen_X, Cen_Y, Cen_Z, atomCoord[0], atomCoord[1],
atomCoord[2])
        dist_dict[dist]=f'{atomCoord[0]}_{atomCoord[1]}_{atomCoord[2]}'
    #print(dist_dict)
    #print(min(dist_dict.values()))
    DPA_Coord = dist_dict[max(dist_dict.keys())].split("_")

    DPA_Centroid_X_=DPA_Coord[0]
    DPA_Centroid_Y_ = DPA_Coord[1]
    DPA_Centroid_Z_ = DPA_Coord[2]

    DAM_=np.mean([*dist_dict.keys()])
    DAV_=np.var([*dist_dict.keys()])
    DAS_=skew([*dist_dict.keys()], axis=0, bias=True)
    return DPA_Centroid_X_,DPA_Centroid_Y_,DPA_Centroid_Z_,DAM_,DAV_,DAS_

def Cal_MVS(*args):
    Cen_X=int(float(args[0]))
    Cen_Y=int(float(args[1]))
    Cen_Z=int(float(args[2]))
    dist_dict={}
    for atom1 in residue:
        atomCoord = atom1.get_vector()
        dist=calDist(Cen_X, Cen_Y, Cen_Z, atomCoord[0], atomCoord[1],
atomCoord[2])
        dist_dict[dist]=f'{atomCoord[0]}_{atomCoord[1]}_{atomCoord[2]}'
```

```
    #print(dist_dict)
    #print(min(dist_dict.values()))

    M=np.mean([*dist_dict.keys()])
    V=np.var([*dist_dict.keys()])
    S=skew([*dist_dict.keys()], axis=0, bias=True)
    return M,V,S


Dict_drug={}
Output_Folder_Path="/ddnB/work/wxx6941/TSR/code/code/psi_revision/9pdb/ou
tput_trp_a_b"
OutputFile=open(f'{Output_Folder_Path}/Similarity_drugs.txt','w')
for i in range(len(PDB_list)):
    PDB_ID=PDB_list[i]
    Chain_Name = Chain[i]
    #drug_Name = Drug_name[i]
    #drug_Id = Drug_id[i]
    #group=Group[i]

    #print(PDB_ID,Chain_Name,drug_Name,drug_Id)
    PDB_File_Path =
"/ddnB/work/wxx6941/TSR/code/code/psi_revision/9pdb/PDB_datadir_HRemoved/
{}.pdb".format(PDB_ID)
    p = Bio.PDB.PDBParser()
    Structure = p.get_structure('PrimaryStructureChain', PDB_File_Path)

    model = Structure[0]
    for chain in model:
        if chain.id == Chain_Name:

            for residue in chain:

                drug_Identifier = str(residue)[17:18].strip()
                resName = str(residue)[9:12].strip()  # residue Name

                if  resName == 'TRP':
                    count=0
                    for atom1 in residue:
                        count+=1
                    if count==14:

                        numeric_filter = filter(str.isdigit,
str(residue.id))
                        Res_Id = "".join(numeric_filter)  # Residue ID
                        Drug=f'{PDB_ID}_{Chain_Name}_{resName}_{Res_Id}'

Centroid_X,Centroid_Y,Centroid_Z=Cal_Centroid(residue)

NA_Centroid_X,NA_Centroid_Y,NA_Centroid_Z,DA_Centroid_X,DA_Centroid_Y,DA_
Centroid_Z,CM,CV,CS=Cal_NA_Centroid(Centroid_X,Centroid_Y,Centroid_Z,resi
due)
```

```
DPA_Centroid_X,DPA_Centroid_Y,DPA_Centroid_Z,DAM,DAV,DAS=Cal_DPA_Centroid
(DA_Centroid_X,DA_Centroid_Y,DA_Centroid_Z,residue)

                        NAM, NAV, NAS =
Cal_MVS(NA_Centroid_X,NA_Centroid_Y,NA_Centroid_Z, residue)
                        DPAM, DPAV, DPAS =
Cal_MVS(DPA_Centroid_X,DPA_Centroid_Y,DPA_Centroid_Z, residue)

Dict_drug[Drug]=f'{CM}_{CV}_{CS}_{NAM}_{NAV}_{NAS}_{DAM}_{DAV}_{DAS}_{DPA
M}_{DPAV}_{DPAS}'


print(Dict_drug)
comb_drug = combinations([*Dict_drug], 2)
for i in comb_drug:
    Drug1_moments=Dict_drug[i[0]].split("_")
    Drug2_moments = Dict_drug[i[1]].split("_")
    print(Drug1_moments)
    print(Drug2_moments)
    sum=0
    for k in range(len(Drug1_moments)):
        diff=abs(float(Drug1_moments[k])-float(Drug2_moments[k]))
        sum+=diff
    similarity=1/(1+(sum/12))
    OutputFile.write(f'{i[0]}\t{i[1]}\t{similarity}')
    OutputFile.write('\n')

OutputFile.close()
print('completed successfully')
```

(vii) RMSD code for pigments using all atoms except hydrogen atoms

#Pyton code: calculate structural similarity for chl using RMSD

```
#program to calculate the similarity between structures using rmsd

import csv
import math
import Bio.PDB
from Bio.PDB import PDBParser
import pandas as pd
import os
from scipy.stats import skew
import numpy as np
from itertools import combinations

df=pd.read_csv('sample_details_et_mix5_name_id_atom.csv') #Change here
for different sample details file
PDB_list = df['protein'].to_list()
Chain = df['chain'].to_list()
Drug_name = df['drug_name'].to_list()
Drug_id = df['drug_id'].to_list()
Group = df['group'].to_list()
Atom_list=df['ligand_atom'].to_list()

def calDist(x1, y1, z1, x2, y2, z2): #Calculate distance between two
atoms
    return math.sqrt((x2 - x1) ** 2 + (y2 - y1) ** 2 + (z2 - z1) ** 2)

def Cal_Centroid(res): #Calculates the centriod of structures
    SumX = 0
    SumY = 0
    SumZ = 0
    counter = 0
    for atom1 in residue:
        atomCoord = atom1.get_vector()
        SumX += atomCoord[0]
        SumY += atomCoord[1]
        SumZ += atomCoord[2]
        counter += 1
    Centroid_X_ = SumX / counter
    Centroid_Y_ = SumY / counter
    Centroid_Z_ = SumZ / counter
    return Centroid_X_,Centroid_Y_,Centroid_Z_

def translation(*args): #translates the two structures
    CenX=args[0]
    CenY=args[1]
    CenZ=args[2]
    residue_coord_dict={}
    counter=0
    for atom in residue:
        atomCoord = atom.get_vector()
        X1_New=CenX-atomCoord[0]
        Y1_New=CenY-atomCoord[1]
        Z1_New =CenZ-atomCoord[2]
```

```
        residue_coord_dict[counter]=[X1_New,Y1_New,Z1_New]
        counter+=1
    return residue_coord_dict
Dataframe_Index=[]

def kabsch_umeyama(A, B): #the algoritms for alignment by roration and
scaling
    assert A.shape == B.shape
    n, m = A.shape

    EA = np.mean(A, axis=0)
    EB = np.mean(B, axis=0)
    VarA = np.mean(np.linalg.norm(A - EA, axis=1) ** 2)

    H = ((A - EA).T @ (B - EB)) / n
    U, D, VT = np.linalg.svd(H)
    d = np.sign(np.linalg.det(U) * np.linalg.det(VT))
    S = np.diag([1] * (m - 1) + [d])

    R = U @ S @ VT
    c = VarA / np.trace(np.diag(D) @ S)
    t = EA - c * R @ EB

    return R, c, t

def rmsd(A,B): #The rmsd method
    sum=0
    for i in range(len(A)):
        dist=calDist(A[i][0],A[i][1],A[i][2],B[i][0],B[i][1],B[i][2])
        sum+=dist**2
    return np.sqrt(sum/len(A))




counter=0
drug_coord_dict={}
Output_Folder_Path="/ddnB/work/wxx6941/TSR/code/code/psi_revision/5pdb/ou
tput_chl_atom_rmsd"
OutputFile=open(f'{Output_Folder_Path}/similarity_drugs_rmsd.txt','w')
for i in range(len(PDB_list)):
    PDB_ID=PDB_list[i]
    Chain_Name = Chain[i]
    drug_Name = Drug_name[i]
    drug_Id = Drug_id[i]
    atoms=Atom_list[i]
    if drug_Name == 'CL0':
        #drug_Name='CL0'
        drug_Id=f'0{drug_Id}'

    Drug=f'{PDB_ID}_{Chain_Name}_{drug_Name}_{drug_Id}'
    Dataframe_Index.append(Drug)
    #print(PDB_ID,Chain_Name,drug_Name,drug_Id)
```

```
    PDB_File_Path =
"/ddnB/work/wxx6941/TSR/code/code/psi_revision/5pdb/PDB_datadir_HRemoved/
{}.pdb".format(
        PDB_ID)
    p = Bio.PDB.PDBParser()
    Structure = p.get_structure('PrimaryStructureChain', PDB_File_Path)
    model = Structure[0]
    for chain in model:
        if chain.id == Chain_Name:
            for residue in chain:
                drug_Identifier = str(residue)[17:18].strip()
                resName = str(residue)[9:12].strip()  # residue Name

                numeric_filter = filter(str.isdigit, str(residue.id))
                Res_Id = "".join(numeric_filter)  # Residue ID

                if drug_Identifier == 'H' and resName == drug_Name and
Res_Id == str(drug_Id):
                    drug_coord = []
                    for atom in residue:
                        if atom.get_name() in atoms:
                            atomCoord = atom.get_vector()
                            drug_coord.append([atomCoord[0],
atomCoord[1], atomCoord[2]])


                    drug_coord_dict[Drug] = drug_coord
                    counter+= 1

#Centroid_X,Centroid_Y,Centroid_Z=Cal_Centroid(residue)
                    #print(Centroid_X,Centroid_Y,Centroid_Z)
                    #print(translation(Centroid_X,Centroid_Y,Centroid_Z))

comb_drug = combinations([*drug_coord_dict], 2)
for i in comb_drug:
    A=np.array(drug_coord_dict[i[0]])
    B=np.array(drug_coord_dict[i[1]])
    R, c, t = kabsch_umeyama(A, B)

    B = np.array([t + c * R @ b for b in B])
    rmsd_similarity=rmsd(A,B)
    print(rmsd_similarity)
    OutputFile.write(f'{i[0]}\t{i[1]}\t{rmsd_similarity}')
    OutputFile.write('\n')

#df = pd.DataFrame(Total_drug_dict_features, index=Dataframe_Index)
#df = df.astype('int')
#df.to_csv(f"feature_map_with_header.csv", header=True, index=True)
OutputFile.close()
print('completed successfully')
```

(viii) RMSD code for tryptophan using all atoms except hydrogen atoms

#Python code: Calculate the structural similarity for tryptophan using RMSD

```
#program to calculate the similarity between structures using rmsd

import csv
import math
import Bio.PDB
from Bio.PDB import PDBParser
import pandas as pd
import os
from scipy.stats import skew
import numpy as np
from itertools import combinations

df=pd.read_csv('sample_details_psi_ab_mix1.csv') #Change here for
different sample details file
PDB_list = df['protein'].to_list()
Chain = df['chain'].to_list()
#Drug_name = df['drug_name'].to_list()
#Drug_id = df['drug_id'].to_list()
#Group = df['group'].to_list()

def calDist(x1, y1, z1, x2, y2, z2): #Calculate distance between two
atoms
    return math.sqrt((x2 - x1) ** 2 + (y2 - y1) ** 2 + (z2 - z1) ** 2)

def Cal_Centroid(res): #Calculates the centriod of structures
    SumX = 0
    SumY = 0
    SumZ = 0
    counter = 0
    for atom1 in residue:
        atomCoord = atom1.get_vector()
        SumX += atomCoord[0]
        SumY += atomCoord[1]
        SumZ += atomCoord[2]
        counter += 1
    Centroid_X_ = SumX / counter
    Centroid_Y_ = SumY / counter
    Centroid_Z_ = SumZ / counter
    return Centroid_X_,Centroid_Y_,Centroid_Z_

def translation(*args): #translates the two structures
    CenX=args[0]
    CenY=args[1]
    CenZ=args[2]
    residue_coord_dict={}
    counter=0
    for atom in residue:
        atomCoord = atom.get_vector()
        X1_New=CenX-atomCoord[0]
        Y1_New=CenY-atomCoord[1]
        Z1_New =CenZ-atomCoord[2]
        residue_coord_dict[counter]=[X1_New,Y1_New,Z1_New]
```

```
        counter+=1
    return residue_coord_dict
#Dataframe_Index=[]

def kabsch_umeyama(A, B): #the algoritms for alignment by roration and 
scaling
    assert A.shape == B.shape
    n, m = A.shape

    EA = np.mean(A, axis=0)
    EB = np.mean(B, axis=0)
    VarA = np.mean(np.linalg.norm(A - EA, axis=1) ** 2)

    H = ((A - EA).T @ (B - EB)) / n
    U, D, VT = np.linalg.svd(H)
    d = np.sign(np.linalg.det(U) * np.linalg.det(VT))
    S = np.diag([1] * (m - 1) + [d])

    R = U @ S @ VT
    c = VarA / np.trace(np.diag(D) @ S)
    t = EA - c * R @ EB

    return R, c, t

def rmsd(A,B): #The rmsd method
    sum=0
    for i in range(len(A)):
        dist=calDist(A[i][0],A[i][1],A[i][2],B[i][0],B[i][1],B[i][2])
        sum+=dist**2
    return np.sqrt(sum/len(A))




counter=0
drug_coord_dict={}
Output_Folder_Path="/ddnB/work/wxx6941/TSR/code/code/psi_revision/9pdb/ou
tput_trp_a_b_rmsd"
OutputFile=open(f'{Output_Folder_Path}/similarity_drugs_rmsd.txt','w')
for i in range(len(PDB_list)):
    PDB_ID=PDB_list[i]
    Chain_Name = Chain[i]
    #drug_Name = Drug_name[i]
    #drug_Id = Drug_id[i]

    #Dataframe_Index.append(Drug)
    #print(PDB_ID,Chain_Name,drug_Name,drug_Id)
    PDB_File_Path = 
"/ddnB/work/wxx6941/TSR/code/code/psi_revision/9pdb/PDB_datadir_HRemoved/
{}.pdb".format(
        PDB_ID)
    p = Bio.PDB.PDBParser()
    Structure = p.get_structure('PrimaryStructureChain', PDB_File_Path)
    model = Structure[0]
```

```
    for chain in model:
        if chain.id == Chain_Name:
            for residue in chain:
                drug_Identifier = str(residue)[17:18].strip()
                resName = str(residue)[9:12].strip()  # residue Name

                numeric_filter = filter(str.isdigit, str(residue.id))
                Res_Id = "".join(numeric_filter)  # Residue ID

                if resName == 'TRP':
                    count=0
                    for atom1 in residue:
                        count+=1
                    if count==14:
                        Drug=f'{PDB_ID}_{Chain_Name}_{resName}_{Res_Id}'

                        drug_coord = []
                        for atom in residue:
                            atomCoord = atom.get_vector()
                            drug_coord.append([atomCoord[0],
atomCoord[1], atomCoord[2]])
                        drug_coord_dict[Drug] = drug_coord
                        counter += 1

#Centroid_X,Centroid_Y,Centroid_Z=Cal_Centroid(residue)
                    #print(Centroid_X,Centroid_Y,Centroid_Z)
                    #print(translation(Centroid_X,Centroid_Y,Centroid_Z))

comb_drug = combinations([*drug_coord_dict], 2)
for i in comb_drug:
    A=np.array(drug_coord_dict[i[0]])
    B=np.array(drug_coord_dict[i[1]])
    R, c, t = kabsch_umeyama(A, B)

    B = np.array([t + c * R @ b for b in B])
    rmsd_similarity=rmsd(A,B)
    print(rmsd_similarity)
    OutputFile.write(f'{i[0]}\t{i[1]}\t{rmsd_similarity}')
    OutputFile.write('\n')

#df = pd.DataFrame(Total_drug_dict_features, index=Dataframe_Index)
#df = df.astype('int')
#df.to_csv(f"feature_map_with_header.csv", header=True, index=True)
OutputFile.close()
print('completed successfully')
```

(ix) aminoAcidCode_lexicographic_new.txt

PHE	4
CYS	5
GLN	6
GLU	7
LEU	8
HIS	9
ILE	10
GLY	11
LYS	12
MET	13
ASP	14
PRO	15
SER	16
THR	17
TRP	18
TYR	19
VAL	20
ALA	21
ARG	22
ASN	23
TPO	24
SEP	25
PTR	26
MSE	13
NIY	27
M3L	28
TRF	29
CSO	30
LLP	31
CME	32
CSD	33

(x) drug_atom_lexical_txt.csv

atom	seq	ATOM
Ac	4	AC
Ag	5	AG
Al	6	AL
Am	7	AM
Ar	8	AR
As	9	AS
At	10	AT
Au	11	AU
B	12	B
Ba	13	BA
Be	14	BE
Bh	15	BH
Bi	16	BI
Bk	17	BK
Br	94	BR
BR	94	BR
C	106	C
Ca	99	CA
CA	106	CA
CAA	106	CAA
CAB	106	CAB
CAC	106	CAC
CAD	106	CAD
CAE	106	CAE
CAF	106	CAF
CAG	106	CAG
CAH	106	CAH
CAI	106	CAI
CAJ	106	CAJ
CAK	106	CAK
CAL	106	CAL
CAM	106	CAM
CAN	106	CAN
CAO	106	CAO
CAP	106	CAP
CAQ	106	CAQ
CAR	106	CAR
CAS	106	CAS
CAT	106	CAT
CAU	106	CAU
CAV	106	CAV
CAW	106	CAW
CAX	106	CAX

CAY	106 CAY
CAZ	106 CAZ
CB	106 CB
CBA	106 CBA
CBB	106 CBB
CBC	106 CBC
CBD	106 CBD
CBE	106 CBE
CBF	106 CBF
CBG	106 CBG
CBH	106 CBH
CBI	106 CBI
CBJ	106 CBJ
CBK	106 CBK
CBL	106 CBL
CBM	106 CBM
CBN	106 CBN
CBO	106 CBO
CBP	106 CBP
C5'	106 C5'
C4'	106 C4'
C3'	106 C3'
C2'	106 C2'
C1'	106 C1'
C1	106 C1
C2	106 C2
C3	106 C3
C4	106 C4
C5	106 C5
C6	106 C6
C7	106 C7
C8	106 C8
C02	106 C02
C05	106 C05
C07	106 C07
C09	106 C09
C82	106 C82
C83	106 C83
C85	106 C85
C86	106 C86
C87	106 C87
C97	106 C97
C4S	106 C4S
C3S	106 C3S

C5S	106	C5S
C6S	106	C6S
C9	106	C9
C10	106	C10
C11	106	C11
C12	106	C12
C13	106	C13
C14	106	C14
C15	106	C15
C16	106	C16
C17	106	C17
C18	106	C18
C19	106	C19
C20	106	C20
C21	106	C21
C22	106	C22
C23	106	C23
C24	106	C24
C25	106	C25
C25	106	C25
C26	106	C26
C27	106	C27
C28	106	C28
C29	106	C29
C30	106	C30
C31	106	C31
C32	106	C32
C33	106	C33
C34	106	C34
C35	106	C35
C36	106	C36
C37	106	C37
C38	106	C38
C39	106	C39
C40	106	C40
C41	106	C41
C42	106	C42
C43	106	C43
C44	106	C44
C45	106	C45
C46	106	C46
C47	106	C47
C48	106	C48
C49	106	C49

C50	106	C50
C51	106	C51
C52	106	C52
C53	106	C53
C54	106	C54
C61	106	C61
C62	106	C62
C63	106	C63
C64	106	C64
C65	106	C65
C66	106	C66
C67	106	C67
C70	106	C70
C71	106	C71
C72	106	C72
C73	106	C73
C74	106	C74
C75	106	C75
C76	106	C76
C77	106	C77
C78	106	C78
C55	106	C55
C56	106	C56
C57	106	C57
C58	106	C58
C59	106	C59
CBQ	106	CBQ
CBR	106	CBR
CBS	106	CBS
CBT	106	CBT
CBV	106	CBV
CBY	106	CBY
CC	106	CC
Cd	18	CD
CD	106	CD
CD1	106	CD1
CD2	106	CD2
CD3	106	CD3
CD4	106	CD4
CDO	106	CDO
Ce	19	CE
CE	106	CE
CE1	106	CE1
CE2	106	CE2

CE3	106	CE3
CE4	106	CE4
CE5	106	CE5
CED	106	CED
CH2	106	CH2
CZ1	106	CZ1
CZ2	106	CZ2
CZ3	106	CZ3
Cf	20	CF
CG2	106	CG2
CG1	106	CG1
CG	106	CG
CGA	106	CGA
CGD	106	CGD
CGO	106	CGO
CH	106	CH
CHA	106	CHA
CHB	106	CHB
CHC	106	CHC
CHD	106	CHD
CI	106	CI
CL	93	CL
CLE	93	CLE
Cm	21	CM
CM	106	CM
CMA	106	CMA
CMB	106	CMB
CMC	106	CMC
CMD	106	CMD
Co	102	CO
CO	106	CO
CP	106	CP
Cr	22	CR
Cs	23	CS
CT	106	CT
Cu	104	CU
CZ	106	CZ
D11	24	D11
D12	24	D12
D13	24	D13
D21	24	D21
D22	24	D22
D23	24	D23
Db	24	DB

Dy	25	DY
E21	26	E21
E22	26	E22
Er	26	ER
Es	27	ES
Eu	28	EU
F	92	F10
F	92	F9
F	92	F8
F	92	F11
F	92	F
F1	92	F1
F2	92	F2
F3	92	F3
F39	92	F39
F40	92	F40
F61	92	F61
F62	92	F62
FAA	92	FAA
FAB	92	FAB
FAD	92	FAD
FAE	92	FAE
FAF	92	FAF
Fe	101	FE
Fm	29	FM
Fr	30	FR
Ga	31	GA
Gd	32	GD
G11	33	G11
G12	33	G12
G13	33	G13
G21	33	G21
G22	33	G22
G23	33	G23
Ge	33	GE
H	107	H
D	107	D
H6	107	H6
H7	107	H7
H18	107	H18
H19	107	H19
H20	107	H20
H17	107	H17
H16	107	H16

H15	107	H15
H14	107	H14
H10	107	H10
H8	107	H8
H9	107	H9
H13	107	H13
H5	107	H5
H28	107	H28
H29	107	H29
H30	107	H30
H31	107	H31
H32	107	H32
H33	107	H33
H34	107	H34
H35	107	H35
H36	107	H36
H39	107	H39
H40	107	H40
H38	107	H38
H41	107	H41
H42	107	H42
H43	107	H43
H59	107	H59
H60	107	H60
H61	107	H61
H62	107	H62
H63	107	H63
H64	107	H64
H65	107	H65
H66	107	H66
H67	107	H67
H68	107	H68
H69	107	H69
H70	107	H70
H77	107	H77
H78	107	H78
H79	107	H79
H80	107	H80
H88	107	H88
H89	107	H89
H90	107	H90
H91	107	H91
H92	107	H92
H93	107	H93

H94	107	H94
H95	107	H95
H96	107	H96
H71	107	H71
H72	107	H72
H73	107	H73
H74	107	H74
H75	107	H75
H76	107	H76
H4	107	H4
H2	107	H2
H3	107	H3
HA	107	HA
HB	107	HB
HB2	107	HB2
HB3	107	HB3
HD2	107	HD2
HD3	107	HD3
H1	107	H1
HH11	107	HH11
HH12	107	HH12
HH21	107	HH21
HH22	107	HH22
H11	107	H11
H12	107	H12
H21	107	H21
H22	107	H22
HC	107	HC
HZ1	107	HZ1
HE3	107	HE3
HZ2	107	HZ2
HZ3	107	HZ3
HH2	107	HH2
He	34	HE
HE1	107	HE1
HE2	107	HE2
HE21	107	HE21
HE22	107	HE22
HZ	107	HZ
HB1	107	HB1
HA2	107	HA2
HA3	107	HA3
HD1	107	HD1
HD11	107	HD11

HD12	107	HD12
HD13	107	HD13
HD22	107	HD22
HD23	107	HD23
HD21	107	HD21
HE	107	HE
Hf	35	HF
Hg	36	HG
HG3	36	HG3
HG11	36	HG11
HG12	36	HG12
HG13	36	HG13
HG21	36	HG21
HG22	36	HG22
HG23	36	HG23
HG1	36	HG1
HG2	36	HG2
HH	107	HH
HN	107	HN
HNC	107	HNC
HNM	107	HNM
Ho	37	HO
HO	107	HO
HOA	107	HOA
HOB	107	HOB
HOC	107	HOC
HOD	107	HOD
HOL	107	HOL
Hs	38	HS
I	95	I
In	39	IN
Ir	40	IR
K	97	K
Kr	41	KR
La	42	LA
Li	43	LI
Lr	44	LR
Lu	45	LU
Md	46	MD
Mg	98	MG
MG	98	MG
Mn	100	MN
Mo	47	MO
Mt	48	MT

N	108	N
N1	108	N1
N03	108	N03
N06	108	N06
N81	108	N81
N99	108	N99
NAK	108	NAK
NAE	108	NAE
Na	96	NA
NA	108	NA
NAA	108	NAA
NAB	108	NAB
NAD	108	NAD
NAF	108	NAF
NAJ	108	NAJ
NAL	108	NAL
NAM	108	NAM
NAH	108	NAH
NAI	108	NAI
NAN	108	NAN
NAO	108	NAO
NAP	108	NAP
NAQ	108	NAQ
NAR	108	NAR
NAS	108	NAS
NAT	108	NAT
NAU	108	NAU
NAV	108	NAV
NAW	108	NAW
Nb	49	NB
NB	108	NB
NBA	108	NBA
NBB	108	NBB
NBD	108	NBD
NBE	108	NBE
NBF	108	NBF
NBG	108	NBG
NBH	108	NBH
NBI	108	NBI
NBT	108	NBT
NBU	108	NBU
NBV	108	NBV
NBW	108	NBW
NBX	108	NBX

NC	108	NC
N1	108	N1
N2	108	N2
N3	108	N3
N4	108	N4
N5	108	N5
N6	108	N6
N7	108	N7
N7	108	N7
N8	108	N8
N9	108	N9
N10	108	N10
N11	108	N11
N12	108	N12
N13	108	N13
N14	108	N14
N15	108	N15
N16	108	N16
N17	108	N17
N18	108	N18
N19	108	N19
N20	108	N20
N21	108	N21
N22	108	N22
N23	108	N23
N24	108	N24
N25	108	N25
N26	108	N26
N27	108	N27
N28	108	N28
N29	108	N29
N30	108	N30
N31	108	N31
N32	108	N32
N33	108	N33
N34	108	N34
N35	108	N35
N36	108	N36
N37	108	N37
N38	108	N38
N39	108	N39
N40	108	N40
N41	108	N41
N42	108	N42

N43	108	N43
N44	108	N44
N45	108	N45
N46	108	N46
N47	108	N47
N48	108	N48
N49	108	N49
N50	108	N50
N51	108	N51
N52	108	N52
N53	108	N53
N54	108	N54
N55	108	N55
N56	108	N56
N57	108	N57
N58	108	N58
N59	108	N59
Nd	50	ND
ND2	108	ND2
ND1	108	ND1
ND	108	ND
Ne	51	NE
NE	108	NE
NE1	108	NE1
NE2	108	NE2
NE3	108	NE3
NE4	108	NE4
NE5	108	NE5
NE6	108	NE6
NEO	108	NEO
NF	108	NF
NH	108	NH
NH1	108	NH1
NH2	108	NH2
Ni	103	NI
NN	108	NN
No	52	NO
NO	108	NO
NOA	108	NOA
Np	53	NP
NXT	108	NXT
O	109	O
O53	109	O53
O54	109	O54

O55	109	O55
O56	109	O56
O57	109	O57
O58	109	O58
O61	109	O61
O68	109	O68
O01	109	O01
O04	109	O04
O08	109	O08
O84	109	O84
O98	109	O98
O4S	109	O4S
O3S	109	O3S
OA	109	OA
OAA	109	OAA
OAB	109	OAB
OAC	109	OAC
OAD	109	OAD
OAZ	109	OAZ
OBA	109	OBA
OAE	109	OAE
OAF	109	OAF
OAG	109	OAG
OAH	109	OAH
OAI	109	OAI
OAJ	109	OAJ
OAK	109	OAK
OAL	109	OAL
OAM	109	OAM
OAN	109	OAN
OAO	109	OAO
OAP	109	OAP
OAQ	109	OAQ
OAR	109	OAR
OAS	109	OAS
OAT	109	OAT
OAU	109	OAU
OAV	109	OAV
OAW	109	OAW
OB	109	OB
OBC	109	OBC
OBD	109	OBD
OBE	109	OBE
OBG	109	OBG

OBH	109	OBH
OBI	109	OBI
OBJ	109	OBJ
OD	109	OD
OD1	109	OD1
OD2	109	OD2
OD3	109	OD3
OD4	109	OD4
OD5	109	OD5
OD6	109	OD6
OD7	109	OD7
OD8	109	OD8
OD9	109	OD9
OD10	109	OD10
OD11	109	OD11
OD12	109	OD12
OD13	109	OD13
OE	109	OE
OE1	109	OE1
OE2	109	OE2
OG2	109	OG2
OG1	109	OG1
OG	109	OG
OH	109	OH
OP1	109	OP1
OP2	109	OP2
OP	109	OP
Os	54	OS
OS	109	OS
OT	109	OT
OXT	109	OXT
O5'	109	O5'
O4'	109	O4'
O3'	109	O3'
O2'	109	O2'
O1'	109	O1'
O1	109	O1
P	110	P
Pa	55	PA
Pb	56	PB
Pd	57	PD
Pm	58	PM
Po	59	PO
Pr	60	PR

Pt	61	PT
Pu	62	PU
Ra	63	RA
Rb	64	RB
Re	65	RE
Rf	66	RF
Rh	67	RH
Rn	68	RN
Ru	112	RU
S	111	S
S1	111	S1
S2	111	S2
S3	111	S3
S5'	111	S5'
S23	111	S23
S51	111	S51
S6	111	S6
S7	111	S7
S8	111	S8
S9	111	S9
S31	111	S31
S29	111	S29
S79	111	S79
S67	111	S67
S24	111	S24
S12	111	S12
S13	111	S13
S26	111	S26
S62	111	S62
S63	111	S63
S64	111	S64
S15	111	S15
S17	111	S17
S5S	111	S5S
SAZ	111	SAZ
SAO	111	SAO
SAT	111	SAT
Sb	69	SB
Sc	70	SC
Se	71	SE
Sg	72	SG
Si	73	SI
Sm	74	SM
Sn	75	SN

Sr	76	SR
Ta	77	TA
Tb	78	TB
Tc	79	TC
Te	80	TE
Th	81	TH
Ti	82	TI
Tl	83	TL
Tm	84	TM
U	85	U
V	86	V
W	87	W
Xe	88	XE
Y	89	Y
Yb	90	YB
Zn	105	ZN
Zr	91	ZR
TE	80	TE
S	111	SD
N	108	NZ
CL	93	CL11
O	109	O2
O	109	O3
O	109	O4
O	109	O5
O	109	O6
O	109	O7
O	109	O8
O	109	O9
O	109	O10
O	109	O11
O	109	O12
O	109	O13
O	109	O14
O	109	O15
O	109	O16
O	109	O17
O	109	O18
O	109	O19
O	109	O20
O	109	O21
O	109	O22
O	109	O23
O	109	O24

O	109	O25
O	109	O26
O	109	O27
O	109	O28
O	109	O29
O	109	O30
O	109	O31
O	109	O32
O	109	O33
O	109	O34
O	109	O35
O	109	O36
O	109	O37
O	109	O38
O	109	O39
O	109	O40
O	109	O41
O	109	O42
O	109	O43
O	109	O44
O	109	O45
O	109	O46
O	109	O47
O	109	O48
O	109	O49
O	109	O50
CL11	93	CL1
CL11	93	L11
N	108	N63
H	107	HN1
H	107	N1A
H	107	12A
H	107	14A
H	107	15A
H	107	16A
H	107	18A
H	107	18B
H	107	20A
H	107	20B
H	107	H24
H	107	H25
H	107	25A
H	107	H27
H	107	29A

H	107	30A
H	107	32A
H	107	H37
C	106	C68
N	108	N74
O	109	O76
C	106	C80
S	111	S81
N	108	N83
C	106	C90
C	106	C95
N	108	NBO
O	109	OBV
S	111	SAB
N	108	NBQ
CL	93	L0
C	106	C6'
C	106	C7'
C	106	C8'
CL	93	L1
CL	93	L2
S	111	S4
F	92	F18
F	92	F20
O	109	O81
N	108	N82
C	106	C88
C	106	C08
C	106	C04
C	106	C06
C	106	C01
N	108	N4'
C	106	C79
N	108	N76
N	108	N77
N	108	N02
C	106	C03
O	109	O59
N	108	N60
C	106	C69
N	108	N64
C	106	C2A
C	106	C3A
O	109	O3A

C	106	C1A
C	106	C4A
C	106	C5A
S	111	S5A
H	107	14B
S	111	SAY
S	111	SBK
O	109	OAX
N	108	N8Q
N	108	N1'
CL	93	L04
CL	93	L22
F	92	F21
F	92	F22
F	92	F33
N	108	N04
O	109	O06
N	108	NP6
F	92	F28
F	92	F29
C	106	C9'
N	108	N3'
N	108	N2'
BR	94	R
F	92	F59
F	92	F60
CL	93	L6
F	92	F5
BR	94	R4
F	92	F02
F	92	F03
F	92	F01
F	92	F38
F	92	F14
F	92	F12
F	92	F13
BR	94	R1
F	92	F30
CL	93	L25
CL	93	L18
S	111	S22
F	92	F35
F	92	F36
O	109	O1A

O	109	O2A
O	109	O3B
O	109	O4B
O	109	O02
C	106	C1X
C	106	C2X
C	106	C3X
O	109	O3X
C	106	C4X
O	109	O4X
C	106	C5X
O	109	O5X
C	106	C6X
N	108	NV2
C	106	CV1
C	106	CV6
C	106	CV2
C	106	CV7
C	106	CV3
C	106	CV8
C	106	CV4
C	106	CV9
C	106	CV5
O	109	OV1
O	109	OV2
N	108	NL
C	106	CGL
C	106	CL2
O	109	OT1
O	109	OT2
H	107	HV2
H	107	V12
H	107	HV6
H	107	V33
H	107	V83
H	107	V42
H	107	V93
H	107	H1A
H	107	CB1
H	107	CB2
H	107	HL
H	107	HAL
H	107	BL2
H	107	HGL

H	107	L12	
H	107	L21	
H	107	HC5	
H	107	H7A	
H	107	H7B	
H	107	H7C	
O	109	OP4	
O	109	OP3	
C	106	CA1	
C	106	E11	
C	106	CA2	
C	106	CA3	
C	106	CB3	
C	106	CG3	
C	106	E12	
P	110	P4	
P	110	P8	
O	109	O1P	
O	109	O2P	
O	109	O3P	
F	92	F43	
C	106	C8A	
C	106	C7A	
C	106	C81	
C	106	CM2	
N	108	N4A	
C	106	CM4	
N	108	N3A	
C	106	C6A	
N	108	NA2	
N	108	NA4	
F	92	F4	
F	92	F8Q	
C	106	CS5	
CL	93	L28	
H	107	O1I	
H	107		101
H	107		102
H	107		111
H	107		112
H	107		161
H	107		171
H	107		191
H	107		201

H	107		231
H	107		241
H	107		261
H	107		271
H	107		251
H	107		252
H	107		262
H	107		281
H	107		282
H	107		291
H	107		292
H	107		232
H	107		121
H	107		122
H	107		123
H	107		321
H	107		322
H	107		323
H	107		311
H	107		312
H	107		313
S	111	SAM	
S	111	SAQ	
H	107	HAP	
H	107	HAO	
H	107	HAN	
H	107	HAM	
H	107	HAH	
H	107	HAD	
H	107	HAC	
H	107	HAA	
H	107	HAF	
H	107	HAV	
H	107	HAT	
S	111	S16	
F	92	F17	
S	111	S27	
O	109	O60	
S	111	S32	
H	107	HO6	
H	107	HO1	
H	107	HO3	
O	109	O66	
O	109	O1B	

C	106	C91
N	108	NP7
F	92	F7
S	111	S14
S	111	S6'
N	108	N79
P	110	P1
N	108	N09
S	111	S11
N	108	NP2
BR	94	RAI
N	108	N08
N	108	NBC
H	107	H23
H	107	H26
CL	93	L17
F	92	F23
F	92	F24
O	109	O51
C	106	C60
O	109	O77
F	92	FAH
F	92	FAI
F	92	FAK
F	92	FAL
H	107	HAB
H	107	HAE
S	111	S74
C	106	CA4
C	106	CA5
C	106	CA6
N	108	NB1
C	106	CB4
C	106	CB5
C	106	CB6
N	108	NC1
C	106	CC2
N	108	NC3
C	106	CC4
C	106	CC5
C	106	CD5
C	106	CD6
F	92	FD3
P	110	PBY

C	106	CBX
O	109	OBK
C	106	CBU
O	109	O03
S	111	S42
S	111	S28
CL	93	L5
S	111	S25
O	109	O83
O	109	O97
N	108	N1B
S	111	S1B
C	106	C2B
N	108	N2B
C	106	C3B
C	106	C4B
N	108	N5'
C	106	C5B
C	106	C6B
C	106	C7B
C	106	C8B
C	106	C9B
O	109	OCB
BR	94	R44
S	111	S49
I	95	I7
C	106	CC1
C	106	CF1
F	92	F34
O	109	O6B
O	109	O2B
C	106	C1B
C	106	C2D
C	106	C4D
O	109	OC
C	106	C1C
C	106	C2C
C	106	C3C
C	106	C4C
C	106	C5C
C	106	C6C
H	107	H51
H	107	H52
H	107	131

H	107		141
O	109	OA2	
O	109	OA3	
O	109	OA6	
C	106	CG4	
C	106	CZ4	
C	106	CZ5	
C	106	CZ6	
C	106	CZ7	
N	108	NZ7	
S	111	S10	
C	106	CP1	
C	106	CP2	
C	106	CP3	
C	106	CP4	
C	106	CP5	
C	106	CP6	
C	106	CR1	
C	106	CR2	
C	106	CR3	
C	106	CR4	
C	106	CR5	
C	106	CR6	
O	109	O67	
S	111	S36	
F	92	F31	
F	92	F32	
F	92	F16	
S	111	S38	
C	106	CDB	
C	106	CDL	
C	106	CEB	
C	106	CEL	
P	110	P3	
C	106	CDE	
C	106	CEE	
C	106	CEO	
H	107	H57	
H	107	H58	
H	107	H47	
H	107	H48	
H	107	H49	
H	107	H50	
H	107	H55	

H	107	H54
H	107	H53
H	107	H56
H	107	H81
N	108	N0
C	106	CA0
C	106	CB0
C	106	CE0
C	106	C0
O	109	O0
N	108	N'
C	106	CA'
C	106	CB'
C	106	CG'
C	106	CD'
C	106	CE'
C	106	CGB
O	109	OL
C	106	CN
N	108	NBK
O	109	OBL
S	111	SBM
O	109	OBO
O	109	OBP
C	106	CBW
C	106	CBZ
C	106	CCA
N	108	NAG
CL	93	LB
C	106	C8Q
N	108	N07
O	109	O09
S	111	S33
H	107	HAI
H	107	HAQ
H	107	HAK
H	107	H44
H	107	H45
H	107	H46
N	108	NAY
O	109	O63
BR	94	R2
H	107	H1B
H	107	H6A

H	107	H6B
H	107	H9A
H	107	HN3
H	107	23A
H	107	23B
H	107	24A
H	107	24B
H	107	HN5
H	107	27A
H	107	30B
S	111	SAG
F	92	F44
F	92	F45
N	108	NAC
S	111	SAN
O	109	OBB
O	109	OBM
N	108	NBP
O	109	OBR
O	109	OBT
N	108	NBZ
C	106	CCB
O	109	O52
O	109	O65
C	106	C1F
C	106	C1E
C	106	C1D
C	106	C1H
C	106	C1I
C	106	C1J
C	106	C1K
C	106	C1L
C	106	C1M
C	106	C1G
O	109	O1G
O	109	O3F
C	106	C3G
N	108	N3H
C	106	C3D
C	106	C3E
C	106	C3I
C	106	C3N
C	106	C3M
C	106	C3L

C	106	C3K	
C	106	C3J	
H	107	HO4	
H	107	HO2	
F	92	F47	
CL	93	L3	
N	108	NAX	
N	108	NAZ	
S	111	S30	
S	111	S53	
H	107		151
H	107		202
H	107		211
H	107		212
H	107		221
H	107		12
H	107		11
H	107		21
H	107		31
H	107		32
H	107		41
H	107		42
H	107		51
H	107		61
H	107		72
H	107		71
H	107		81
H	107		142
H	107		192
H	107		331
H	107		342
H	107		341
H	107		351
H	107		352
H	107		412
H	107		413
H	107		411
H	107		422
H	107		423
H	107		421
H	107		433
H	107		432
H	107		431
H	107		461

H	107	471
H	107	481
H	107	491
H	107	521
H	107	361
H	107	541
O	109 OAY	
F	92 FBI	
F	92 FBJ	
O	109 OBN	
S	111 SBR	
F	92 F26	
H	107	132
H	107	133
H	107 91C	
H	107 92C	
H	107 81C	
H	107 82C	
H	107 71C	
H	107 72C	
CL	93 L36	
O	109 O'	
F	92 2'1	
F	92 3'1	
F	92 4'1	
F	92 5'1	
F	92 6'1	
F	92 2'2	
F	92 3'2	
F	92 4'2	
F	92 5'2	
F	92 6'2	
F	92 6'3	
O	109 O1S	
O	109 O2S	
N	108 N3S	
P	110 P22	
S	111 S45	
S	111 S50	
S	111 S19	
P	110 P2	
O	109 O05	
H	107 11A	
H	107 11B	

H	107	22A	
H	107	22B	
H	107	26A	
H	107	28A	
H	107	28B	
CL	93	L68	
O	109	OBF	
F	92	F15	
N	108	N05	
F	92	F19	
P	110	PAX	
F	92	F25	
CL	93	L7	
H	107		181
H	107		182
F	92	F49	
F	92	F50	
F	92	F51	
F	92	F52	
N	108	N8'	
CL	93	L24	
CL	93	L19	
F	92	5'3	
O	109	O64	
O	109	O4E	
O	109	O8E	
S	111	SBN	
S	111	S04	
C	106	CJ	
C	106	CK	
N	108	NM	
C	106	CQ	
S	111	SAW	
S	111	SAX	
S	111	SAL	
H	107	HAJ	
H	107	HAR	
CL	93	L33	
CL	93	L13	
C	106	CVX	
C	106	CWX	
O	109	OXX	
O	109	OYX	
C	106	C7X	

O	109	O8X	
O	109	O9X	
O	109	O6'	
N	108	N7A	
N	108	N2A	
S	111	S4A	
N	108	N7B	
O	109	O8B	
O	109	O9B	
H	107	H82	
H	107		152
H	107		301
H	107		302
H	107		332
H	107		172
H	107		222
H	107		13
H	107		22
H	107		162
H	107		492
H	107		493
H	107		501
H	107		511
CL	93	L23	
S	111	S61	
CL	93	L43	
F	92	F41	
F	92	FAS	
F	92	FBM	
C	106	C9A	
CL	93	L15	
CL	93	L16	
S	111	S9B	
H	107	H99	
C	106	C8C	
S	111	S21	
S	111	S20	
F	92	4'3	
N	108	NBY	
F	92	F27	
CL	93	LD	
O	109	OA1	
C	106	CGM	
C	106	CDD	

C	106	CDN	
C	106	CEN	
C	106	CGF	
C	106	CGG	
C	106	CG6	
C	106	CDG	
C	106	CEG	
C	106	CEQ	
C	106	CDQ	
H	107	HN2	
H	107	HN4	
S	111	SAU	
C	106	CH3	
S	111	S46	
CL	93	L50	
O	109	OC2	
O	109	O5G	
S	111	S18	
H	107		62
H	107		63
H	107		82
H	107		92
H	107		91
H	107		173
H	107		243
H	107		242
H	107		253
C	106	CE6	
C	106	CG5	
H	107	HN7	
H	107	17A	
H	107	17B	
H	107	HN9	
P	110	PAI	
O	109	O82	
H	107		83
H	107		403
H	107		402
H	107		401
H	107		451
H	107		483
H	107		482
H	107		532
H	107		531

H	107		533
S	111	S54	
N	108	N01	
P	110	P18	
P	110	P27	
BR	94	R3	
BR	94	R5	
CL	93	L31	
S	111	S34	
BR	94	R19	
O	109	O91	
O	109	O92	
F	92	FAJ	
I	95	IAE	
S	111	SAJ	
C	106	C92	
C	106	C93	
C	106	C94	
C	106	C96	
N	108	N97	
C	106	C98	
H	107		371
CL	93	L27	
I	95	IAG	
I	95	IAH	
P	110	PG	
O	109	O2G	
O	109	O3G	
CL	93	L9	
H	107	H1'	
H	107	H2'	
H	107	H3'	
H	107	H6'	
BR	94	R23	
P	110	PAW	
H	107		223
H	107		293
H	107		523
H	107		522
F	92	FAC	
P	110	PV	
P	110	PY	
P	110	PZ	
N	108	N1C	

N	108	N1I	
N	108	N1N	
O	109	O1V	
O	109	O1Y	
O	109	O1Z	
O	109	O2D	
C	106	C2I	
C	106	C2N	
N	108	N2N	
O	109	O2V	
O	109	O2Y	
O	109	O2Z	
O	109	O3C	
O	109	O3D	
O	109	O3E	
N	108	N3I	
N	108	N3N	
O	109	O4D	
C	106	C4I	
C	106	C4N	
O	109	O5B	
O	109	O5C	
C	106	C5D	
O	109	O5D	
C	106	C5I	
C	106	C5N	
C	106	C6D	
C	106	C6I	
N	108	N6I	
C	106	C6N	
O	109	O6N	
C	106	C7C	
N	108	N7I	
N	108	N7N	
C	106	C8I	
C	106	C8N	
C	106	C9I	
N	108	N9N	
S	111	S40	
H	107		372
H	107		382
H	107		381
H	107		391
H	107		393

H	107		392
H	107		441
N	108	NXZ	
H	107		203
H	107		52
H	107		333
H	107		343
N	108	NA3	
F	92	FB7	
N	108	NC5	
C	106	CC6	
N	108	NC7	
N	108	ND3	
O	109	O7'	
S	111	S02	
F	92	3'3	
C	106	C84	
N	108	NBJ	
N	108	NCA	
N	108	NBM	
C	106	CCC	
H	107	HC2	
H	107	HC3	
H	107	HC6	
CL	93	L30	
H	107		303
S	111	SAR	
S	111	SAP	
F	92	FL1	
F	92	FL2	
O	109	OC3	
O	109	OC1	
H	107	H8A	
H	107		153
H	107		213
N	108	NBL	
P	110	PBB	
P	110	PBC	
H	107	H03	
H	107	13A	
H	107	H04	
H	107	H05	
H	107	H07	
S	111	SG2	

O	109	OF2	
O	109	OZ1	
C	106	CM1	
O	109	O1N	
O	109	O2N	
O	109	O4P	
O	109	O4A	
O	109	O6A	
H	107	H2A	
H	107	H3A	
H	107	H4A	
H	107	H5A	
H	107	6A1	
H	107	6A2	
H	107	7A1	
H	107	7A2	
H	107	OA4	
CL	93	L32	
CL	93	L29	
S	111	SAS	
C	106	CN4	
H	107	H4'	
H	107	H2B	
H	107	H3B	
H	107	H4B	
H	107	H5B	
H	107		233
H	107		193
O	109	OH1	
O	109	OH2	
S	111	SAV	
I	95	I24	
S	111	S5C	
O	109	O5E	
N	108	N5F	
O	109	O07	
CL	93	LAG	
S	111	SAC	
S	111	S05	
H	107		73
H	107		93
H	107		353
H	107		362
H	107	HAS	

N	108	NA'	
N	108	NB'	
O	109	O62	
P	110	P6	
O	109	O71	
O	109	O72	
P	110	P9	
N	108	N71	
C	106	CCD	
C	106	CCE	
C	106	CCF	
C	106	CCG	
C	106	CCH	
C	106	CCI	
C	106	CCJ	
C	106	CCK	
C	106	CCL	
C	106	CCM	
C	106	CCN	
C	106	CCO	
C	106	CCP	
C	106	CCQ	
C	106	CCR	
C	106	CCS	
H	107		113
H	107		114
H	107		115
O	109	OX2	
O	109	OX1	
O	109	O5A	
N	108	N9A	
N	108	N6A	
O	109	O1D	
N	108	N1T	
C	106	C6T	
C	106	C2T	
O	109	O2T	
N	108	N3T	
C	106	C4T	
O	109	O4T	
C	106	C5T	
C	106	CM5	
F	92	2'3	
P	110	P08	

S	111	S07	
N	108	N95	
C	106	C99	
N	108	N62	
H	107		163
H	107		183
S	111	SBA	
P	110	PAG	
CL	93	L10	
H	107		811
H	107		821
H	107		831
H	107		911
H	107		921
H	107		931
C	106	C1S	
C	106	C2S	
C	106	C2P	
C	106	C3P	
C	106	C4P	
C	106	C5P	
C	106	C6F	
N	108	N1F	
C	106	C2F	
N	108	N2F	
N	108	N3F	
C	106	C4F	
O	109	O4F	
C	106	C8F	
C	106	C7F	
N	108	N3B	
C	106	C5E	
C	106	C4E	
C	106	C2E	
O	109	O2X	
P	110	PC	
O	109	O1C	
O	109	O2C	
P	110	PE	
O	109	O1E	
O	109	O2E	
O	109	O5F	
C	106	C5F	
O	109	O2F	

C	106	C3F	
F	92	F07	
S	111	SBB	
S	111	S39	
H	107	HN6	
N	108	NA1	
S	111	SB2	
H	107	HA1	
H	107		272
H	107	HNW	
H	107	HOX	
N	108	NBN	
F	92	FAG	
BR	94	R7	
BR	94	RAE	
BR	94	RAF	
F	92	F42	
C	106	C1P	
N	108	N1U	
C	106	C6U	
C	106	C2U	
O	109	O2U	
N	108	N3U	
C	106	C4U	
O	109	O4U	
C	106	C5U	
S	111	SAH	
P	110	PBA	
BR	94	RAG	
C	106	B11	
O	109	OB1	
CL	93	L26	
H	107	H5'	
H	107	5''	
C	106	CXQ	
C	106	CXV	
C	106	CXW	
C	106	CXP	
C	106	CXR	
C	106	CXA	
C	106	CXY	
C	106	CXZ	
C	106	CXT	
S	111	SAI	

C	106	CXG
C	106	CM7
H	107	B12
C	106	C5M
F	92	F08
C	106	C7T
N	108	N3C
O	109	OPA
O	109	OPB
O	109	O5P
O	109	O6P
O	109	OPC
O	109	O7P
O	109	O8P
O	109	OPD
O	109	O9P
F	92	F37
P	110	P7
S	111	S2P
C	106	C7M
C	106	C8M
F	92	F04
H	107	61C
H	107	62C
H	107	51C
H	107	52C
H	107	41C
H	107	42C
H	107	31C
H	107	32C
P	110	P5
O	109	OPF
O	109	OPG
O	109	OPH
P	110	PA1
BR	94	R9
BR	94	R10
BR	94	R11
H	107	2C1
H	107	2C2
H	107	6C1
H	107	6C2
H	107	7C1
H	107	7C2

H	107	1C1
H	107	1C2
CL	93	L20
S	111	S09
BR	94	R4'
C	106	CV'
BR	94	R5'
H	107	32'
H	107	33'
H	107	34'
S	111	S01
O	109	O5N
N	108	N4N
C	106	C1N
O	109	O3N
CL	93	L8
P	110	P13
C	106	CNN
P	110	P12
S	111	S47
BR	94	R14
N	108	N1P
H	107	AA2
H	107	AA1
H	107	AB2
H	107	AB1
H	107	AC1
H	107	AG1
H	107	AF1
H	107	AE1
H	107	AI1
H	107	AJ1
H	107	AM2
H	107	AM1
H	107	AN1
H	107	AN2
H	107	AO1
H	107	AO2
H	107	AQ3
H	107	AQ1
H	107	AQ2
H	107	AR1
H	107	AR2
H	107	AR3

H	107	AS3
H	107	AS1
H	107	AS2
H	107	AT2
H	107	AT3
H	107	AT1
H	107	AU3
H	107	AU1
H	107	AU2
BR	94	RG
H	107	H01
H	107	H02
H	107	H09
H	107	H08
H	107	H06
H	107	AD1
H	107	AH1
H	107	AV1
H	107	AK1
H	107	AK2
S	111	S1G
O	109	O7B
BR	94	R12
BR	94	R13
F	92	F6
P	110	P16
P	110	P02
F	92	FBK
S	111	SBE
S	111	SAK
N	108	N4C
C	106	C2H
C	106	C3H
C	106	C4H
C	106	C5H
C	106	C6H
C	106	C7H
O	109	O2H
O	109	O3H
O	109	O4H
O	109	O7H
C	106	C2G
C	106	C4G
C	106	C5G

C	106	C6G
O	109	O4G
O	109	O6G
F	92	F7B
F	92	F7C
F	92	F7A
H	107	AAA
H	107	AAB
H	107	ACA
H	107	AMA
H	107	ANA
H	107	AOA
H	107	AOB
H	107	APA
H	107	AQA
H	107	ARA
H	107	ASA
H	107	01A
H	107	01B
I	95	ILA
S	111	SBQ
P	110	PCL
P	110	PCK
P	110	PCJ
F	92	FAR
H	107	HAG
BR	94	RAX
P	110	P14
O	109	1P1
O	109	2P1
C	106	1'1
N	108	N91
N	108	N61
P	110	P21
H	107	H83
P	110	P33
N	108	N70
O	109	O80
O	109	O75
H	107	HC9
H	107	C'2
H	107	C'3
H	107	C'4
P	110	P11

I	95	I5	
O	109	O4C	
S	111	SBD	
S	111	SBC	
H	107	NB3	
S	111	S5	
S	111	S37	
H	107	10A	
H	107	10B	
H	107	30C	
H	107	40A	
H	107	40B	
H	107	40C	
S	111	SA1	
S	111	SA2	
H	107	H84	
H	107	H85	
H	107	H87	
H	107	H86	
C	106	C6P	
H	107	HO7	
H	107	HO8	
H	107	HO9	
H	107	B21	
H	107	B22	
H	107	B31	
H	107	1'2	
C	106	CW'	
N	108	NX'	
H	107	31A	
H	107	31B	
H	107	32B	
H	107	33A	
H	107	33B	
H	107	33C	
H	107		103
P	110	PBE	
P	110	PBG	
P	110	PBF	
CL	93	LE	
CL	93	LC	
P	110	PBK	
BR	94	RAV	
N	108	N1E	

N	108	N1Y
C	106	C1Q
N	108	N1W
C	106	C1Z
O	109	O1F
N	108	N1X
C	106	C1R
C	106	C2L
N	108	N1V
N	108	N2K
C	106	C1O
F	92	F2B
H	107	42A
H	107	42B
P	110	P2'
P	110	PBS
P	110	PAM
P	110	P44
C	106	CH4
C	106	CH5
CL	93	LAH
O	109	ON1
I	95	I9
N	108	NP3
C	106	A21
P	110	P09
BR	94	R05
O	109	O5J
C	106	C5J
C	106	C4J
O	109	O4J
O	109	O3J
C	106	C2J
O	109	O2J
N	108	N9B
N	108	N6B
O	109	O1Q
F	92	FBC
F	92	FBD
F	92	FBE
O	109	O93
C	106	E13
H	107	143
H	107	HC4

H	107	OB3
H	107	HC7
O	109	OX'
O	109	OY'
P	110	PN
C	106	C7N
O	109	O7N
S	111	S44
BR	94	R01
S	111	SE1
CL	93	LAD
CL	93	LAE
CL	93	LAF
F	92	FAW
F	92	FBB
F	92	FBF
F	92	FBG
I	95	I2
I	95	I1
I	95	I4
C	106	CT1
C	106	CT2
O	109	XT1
N	108	NP0
CL	93	LAC
H	107	AA3
H	107	AC2
H	107	AO3
H	107	AP1
H	107	AP2
H	107	HAU
H	107	HAZ
N	108	N9R
C	106	C8R
N	108	N7R
C	106	C5R
C	106	C6R
N	108	N6R
N	108	N1R
C	106	C2R
N	108	N3R
C	106	C4R
P	110	P01
S	111	SAA

P	110	PAN
P	110	PAO
O	109	OE3
O	109	OE4
O	109	OE5
O	109	OE6
S	111	SG5
O	109	OE7
O	109	OE8
CL	93	LH
C	106	CS1
CL	93	L14
N	108	NG1
N	108	NG2
C	106	CME
H	107	ME1
H	107	ME2
H	107	ME3
H	107	AP3
P	110	P1A
O	109	O1M
P	110	P2A
C	106	C2M
P	110	P3X
C	106	C4M
C	106	C6M
O	109	O7A
O	109	O8A
O	109	O9A
C	106	C9M
O	109	OI
N	108	NV
C	106	CV
O	109	OV
C	106	CZ8
C	106	CZ9
C	106	CZA
C	106	CZB
C	106	CZC
C	106	CZD
C	106	CZE
C	106	CZF
O	109	OZ2
O	109	OZ3

N	108	NZ1
S	111	SZ1
S	111	SZ2
CL	93	L34
H	107	AZ2
H	107	AZ1
H	107	AV2
H	107	BD1
H	107	BD2
H	107	BB1
H	107	BB2
H	107	BC1
H	107	AX1
H	107	AX2
H	107	AW1
H	107	AW2
BR	94	R8
H	107	43C
H	107	83C
S	111	S06
O	109	OCA
O	109	OBU
P	110	PAZ
P	110	PAT
C	106	C'
S	111	S'
C	106	CW2
C	106	CW3
C	106	CW7
C	106	CK2
C	106	CW9
C	106	CK1
N	108	NW2
C	106	CW8
N	108	NW1
C	106	CW4
C	106	CW5
C	106	CW6
C	106	CW1
O	109	OW1
F	92	FAZ
N	108	N90
O	109	OP5
O	109	OP6

H	107	HAY	
H	107	HAX	
H	107	AL1	
H	107	AL2	
O	109	O8C	
O	109	O9C	
C	106	C7D	
S	111	SG1	
S	111	SBH	
N	108	NO1	
P	110	PAP	
BR	94	RAB	
N	108	N1G	
S	111	S1H	
CL	93	L08	
P	110	PAQ	
P	110	P17	
CL	93	LAB	
CL	93	LAQ	
BR	94	RAC	
H	107	HAW	
H	107	HBA	
H	107	HBC	
H	107	HBB	
H	107	HBE	
H	107	HBD	
H	107	HBF	
H	107	HBG	
O	109	OM1	
O	109	OM3	
O	109	OM2	
O	109	OM4	
S	111	SBO	
P	110	PCI	
P	110	PCH	
P	110	PCG	
H	107		733
H	107		731
H	107		732
H	107		643
H	107		642
H	107		641
H	107		512
H	107		513

H	107	HO5	
CL	93	LAA	
P	110	P06	
F	92	F89	
N	108	N96	
P	110	PAJ	
H	107	HE4	
H	107	HE5	
H	107	HKL	
H	107	HCA	
H	107	21A	
H	107	21B	
H	107	29B	
P	110	PAS	
S	111	S08	
P	110	PBH	
I	95	IAQ	
I	95	I3	
I	95	I3'	
I	95	I5'	
C	106	CM3	
F	92	F2'	
H	107		263
C	106	CO1	
C	106	CO2	
C	106	CO3	
C	106	CO4	
C	106	CO5	
C	106	CO6	
H	107	1AA	
H	107	1AB	
H	107	1AC	
P	110	P2B	
O	109	O1X	
C	106	CY1	
C	106	CY2	
C	106	CY3	
C	106	CY4	
C	106	CY5	
C	106	CY6	
C	106	CY0	
C	106	CX2	
C	106	CX3	
C	106	CX4	

C	106	CX5	
C	106	CX6	
C	106	CX1	
C	106	CX0	
O	109	OY1	
O	109	OY2	
C	106	CH1	
C	106	CI1	
C	106	CI2	
O	109	OF3	
S	111	S03	
C	106	CPB	
C	106	CPA	
C	106	CP9	
C	106	CP8	
C	106	CP7	
N	108	NP1	
O	109	OS1	
C	106	CS2	
C	106	CS3	
O	109	OS5	
O	109	OS4	
C	106	CK3	
C	106	CK4	
C	106	CK5	
C	106	CK6	
C	106	CK7	
C	106	CK8	
C	106	CK9	
C	106	CKA	
C	106	CKB	
C	106	CKC	
O	109	OK1	
O	109	OK2	
H	107		273
H	107		283
BR	94	RAT	
BR	94	RAR	
O	109	OBX	
O	109	OBW	
F	92	F3'	
N	108	N5B	
N	108	N5A	
N	108	N4B	

O	109	O6C	
O	109	O6D	
C	106	C6E	
O	109	O6E	
S	111	SAE	
S	111	SCE	
S	111	SCC	
S	111	SCF	
S	111	SCD	
H	107	A31	
H	107	A32	
H	107	A41	
H	107	A42	
H	107	HA5	
H	107	B51	
H	107	B52	
H	107	HA6	
H	107	B61	
H	107	B62	
O	109	OM	
I	95	I19	
I	95	I01	
H	107	AB3	
H	107	H5O	
H	107	4N1	
S	111	S58	
H	107		33
S	111	SBG	
H	107	H2C	
N	108	N72	
N	108	N92	
S	111	S5R	
O	109	O4R	
C	106	C3R	
O	109	O3R	
O	109	O2R	
C	106	CGC	
O	109	OB2	
O	109	OHB	
H	107	12C	
H	107	11C	
C	106	C7,	
C	106	C5,	
C	106	C6,	

C	106	C4,	
N	108	N4,	
N	108	N3,	
C	106	C2,	
N	108	N1,	
O	109	OA7	
N	108	NA5	
O	109	OB4	
O	109	OB5	
O	109	OB6	
C	106	CD7	
F	92	F3A	
H	107	ABA	
H	107	ABB	
H	107	ACB	
F	92	F09	
F	92	F5A	
P	110	P26	
H	107		314
H	107		214
H	107	HOH	
H	107	41O	
H	107		611
H	107		612
H	107	52O	
H	107	23O	
H	107	43O	
H	107		631
H	107		632
H	107	63O	
P	110	P2D	
P	110	P1B	
D	107	DE2	
D	107	DD1	
D	107	DG	
D	107	DG1	
D	107	DZ1	
D	107	DZ2	
D	107	DZ3	
D	107	DE	
D	107	DH	
D	107	DE1	
H	107	13B	
D	107	DN1	

F	92	F9F
O	109	ON
N	108	N4T
H	107	H6C
H	107	H5C
H	107	H4C
H	107	H3C
H	107	H6D
H	107	H5D
H	107	H4D
H	107	H3D
H	107	H2D
P	110	P10
P	110	PAY
P	110	PAE
BR	94	R15
O	109	OS2
O	109	OS3
I	95	I15
I	95	I17
N	108	NT
N	108	N1D
N	108	N3D
S	111	SAD
P	110	PAU
P	110	PAV
P	110	PAK
BR	94	RAW
O	109	OH3
O	109	OH4
O	109	OH5
O	109	OH6
C	106	CA7
C	106	CA8
C	106	CB7
C	106	CB8
P	110	P'
P	110	PAR
N	108	N1K
C	106	C7S
N	108	N1S
N	108	N4S
CL	93	L07
P	110	P28

CL	93	L4
CL	93	L
H	107	H12A
H	107	H14A
H	107	H15A
H	107	H16A
H	107	H18A
H	107	H18B
H	107	HO18
H	107	H20A
H	107	H20B
H	107	HN20
H	107	H25A
H	107	H29A
H	107	H30A
H	107	H32A
CL	93	CL0
H	107	H14B
CL	93	CL04
CL	93	CL22
CL	93	CL6
BR	94	BR4
BR	94	BR1
CL	93	CL25
CL	93	CL18
H	107	HV12
H	107	HV33
H	107	HV83
H	107	HV42
H	107	HV93
H	107	HC21
H	107	HC22
H	107	HCB1
H	107	HCB2
H	107	HBL2
H	107	HL12
H	107	HL21
H	107	HC61
H	107	HC62
C	106	CD11
C	106	CD21
C	106	CE11
C	106	CE21
C	106	CD12

C	106	CD22
C	106	CE12
C	106	CE22
CL	93	CL28
H	107	HO1I
H	107	HN21
H	107	HC51
H	107	HC52
H	107	HC71
H	107	HC72
H	107	HC81
H	107	HC82
H	107	H101
H	107	H102
H	107	H111
H	107	H112
H	107	H161
H	107	H171
H	107	H191
H	107	H201
H	107	H231
H	107	H241
H	107	H261
H	107	H271
H	107	H251
H	107	H252
H	107	H262
H	107	H281
H	107	H282
H	107	H291
H	107	H292
H	107	H232
H	107	H121
H	107	H122
H	107	H123
H	107	H321
H	107	H322
H	107	H323
H	107	H311
H	107	H312
H	107	H313
BR	94	BRAI
CL	93	CL17
CL	93	CL5

BR	94	BR44
H	107	H131
H	107	H141
CL	93	CLB
BR	94	BR2
H	107	H23A
H	107	H23B
H	107	H24A
H	107	H24B
H	107	H27A
H	107	H30B
H	107	HN30
H	107	HN31
CL	93	CL3
H	107	H151
H	107	H202
H	107	H211
H	107	H212
H	107	H221
H	107	H012
H	107	H011
H	107	H021
H	107	H031
H	107	H032
H	107	H041
H	107	H042
H	107	H051
H	107	H061
H	107	H072
H	107	H071
H	107	H081
H	107	H142
H	107	H192
H	107	H331
H	107	H342
H	107	H341
H	107	H351
H	107	H352
H	107	H412
H	107	H413
H	107	H411
H	107	H422
H	107	H423
H	107	H421

H	107	H433
H	107	H432
H	107	H431
H	107	H461
H	107	H471
H	107	H481
H	107	H491
H	107	H521
H	107	H361
H	107	H541
H	107	H132
H	107	H133
H	107	H91C
H	107	H92C
H	107	H81C
H	107	H82C
H	107	H71C
H	107	H72C
CL	93	CL36
F	92	F2'1
F	92	F3'1
F	92	F4'1
F	92	F5'1
F	92	F6'1
F	92	F2'2
F	92	F3'2
F	92	F4'2
F	92	F5'2
F	92	F6'2
F	92	F6'3
H	107	HN51
H	107	H11A
H	107	H11B
H	107	H22A
H	107	H22B
H	107	H26A
H	107	H28A
H	107	H28B
CL	93	CL68
CL	93	CL7
H	107	H181
H	107	H182
CL	93	CL24
CL	93	CL19

F	92	F5'3
CL	93	CL33
CL	93	CL13
H	107	H152
H	107	H301
H	107	H302
H	107	H332
H	107	H172
H	107	H222
H	107	H013
H	107	H022
H	107	H162
H	107	H492
H	107	H493
H	107	H501
H	107	H511
CL	93	CL23
CL	93	CL43
CL	93	CL15
CL	93	CL16
F	92	F4'3
C	106	CG11
C	106	CG21
C	106	CG12
C	106	CG22
CL	93	CLD
H	107	HO3'
CL	93	CL50
H	107	H062
H	107	H063
H	107	H082
H	107	H092
H	107	H091
H	107	H173
H	107	H243
H	107	H242
H	107	H253
H	107	H17A
H	107	H17B
H	107	H083
H	107	H403
H	107	H402
H	107	H401
H	107	H451

H	107	H483
H	107	H482
H	107	H532
H	107	H531
H	107	H533
BR	94	BR3
BR	94	BR5
CL	93	CL31
BR	94	BR19
H	107	H371
CL	93	CL27
CL	93	CL9
H	107	HN3'
BR	94	BR23
H	107	H223
H	107	H293
H	107	H523
H	107	H522
H	107	H372
H	107	H382
H	107	H381
H	107	H391
H	107	H393
H	107	H392
H	107	H441
H	107	H203
H	107	H052
H	107	H333
H	107	H343
F	92	F3'3
H	107	HC3'
H	107	HC4'
H	107	HC5'
CL	93	CL30
H	107	H303
CL	93	CLA
H	107	H153
H	107	H213
H	107	HN12
H	107	HN23
H	107	H13A
H	107	H6A1
H	107	H6A2
H	107	H7A1

H	107	H7A2
H	107	HOA2
H	107	HOA3
H	107	HOA4
H	107	HOA6
H	107	HN41
H	107	HN42
CL	93	CL32
CL	93	CL29
H	107	H233
H	107	H193
CL	93	CLAG
H	107	H073
H	107	H093
H	107	H353
H	107	H362
H	107	H113
H	107	H114
H	107	H115
F	92	F2'3
H	107	H163
H	107	H183
CL	93	CL10
H	107	H811
H	107	H821
H	107	H831
H	107	H911
H	107	H921
H	107	H931
H	107	HCN4
H	107	HC2'
C	106	CG13
C	106	CG23
H	107	HN22
H	107	H2'1
H	107	H2'2
H	107	HN4'
H	107	H272
BR	94	BR7
BR	94	BRAE
BR	94	BRAF
BR	94	BRAG
C	106	CB11
CL	93	CL26

H	107	HOG2
H	107	H5''
H	107	HO2'
H	107	HB11
H	107	HB12
H	107	HN11
H	107	HO17
H	107	H61C
H	107	H62C
H	107	H51C
H	107	H52C
H	107	H41C
H	107	H42C
H	107	H31C
H	107	H32C
H	107	HN26
H	107	HN24
BR	94	BR9
BR	94	BR10
BR	94	BR11
H	107	H2C1
H	107	H2C2
H	107	H6C1
H	107	H6C2
H	107	H7C1
H	107	H7C2
H	107	H1C1
H	107	H1C2
CL	93	CL20
BR	94	BR4'
BR	94	BR5'
H	107	H32'
H	107	H33'
H	107	H34'
CL	93	CL8
BR	94	BR14
H	107	HAA2
H	107	HAA1
H	107	HAB2
H	107	HAB1
H	107	HAC1
H	107	HAG1
H	107	HAF1
H	107	HAE1

H	107	HAI1
H	107	HAJ1
H	107	HAM2
H	107	HAM1
H	107	HAN1
H	107	HAN2
H	107	HAO1
H	107	HAO2
H	107	HAQ3
H	107	HAQ1
H	107	HAQ2
H	107	HAR1
H	107	HAR2
H	107	HAR3
H	107	HAS3
H	107	HAS1
H	107	HAS2
H	107	HAT2
H	107	HAT3
H	107	HAT1
H	107	HAU3
H	107	HAU1
H	107	HAU2
BR	94	BRG
BR	94	BRB
BR	94	BRA
H	107	HAD1
H	107	HAH1
H	107	HAV1
H	107	HAK1
H	107	HAK2
BR	94	BR12
BR	94	BR13
H	107	HAAA
H	107	HAAB
H	107	HACA
H	107	HAMA
H	107	HANA
H	107	HAOA
H	107	HAOB
H	107	HAPA
H	107	HAQA
H	107	HARA
H	107	HASA

H	107	HNAU
H	107	H01A
H	107	H01B
H	107	HNAN
BR	94	BRAX
O	109	O1P1
O	109	O2P1
O	109	O5'1
C	106	C5'1
C	106	C4'1
O	109	O4'1
C	106	C3'1
O	109	O3'1
C	106	C2'1
O	109	O2'1
C	106	C1'1
H	107	HN4A
H	107	HC'2
H	107	HC'3
H	107	HC'4
H	107	HNB3
H	107	H5'2
H	107	H5'1
H	107	H10A
H	107	H10B
H	107	H30C
H	107	H40A
H	107	H40B
H	107	H40C
H	107	HB21
H	107	HB22
H	107	HB31
H	107	H1'1
H	107	H1'2
H	107	H31A
H	107	H31B
H	107	H32B
H	107	H33A
H	107	H33B
H	107	H33C
H	107	H103
CL	93	CLC
BR	94	BRAV
H	107	H42A

H	107	H42B
CL	93	CL21
CL	93	CLAH
C	106	CA21
C	106	CB21
BR	94	BR05
C	106	CD23
C	106	CE13
H	107	H143
H	107	HO2B
H	107	HCB3
H	107	HOB3
H	107	HO6B
BR	94	BR01
CL	93	CLAD
CL	93	CLAE
CL	93	CLAF
C	106	C131
O	109	OXT1
CL	93	CLAC
H	107	HAA3
H	107	HAC2
H	107	HAO3
H	107	HAP1
H	107	HAP2
H	107	HN61
H	107	HN62
CL	93	CLI
CL	93	CLH
CL	93	CL14
H	107	HME1
H	107	HME2
H	107	HME3
H	107	HAP3
CL	93	CL34
H	107	HAZ2
H	107	HAZ1
H	107	HAV2
H	107	HBD1
H	107	HBD2
H	107	HBB1
H	107	HBB2
H	107	HBC1
H	107	HAX1

H	107	HAX2
H	107	HAW1
H	107	HAW2
BR	94	BR8
H	107	H43C
H	107	H83C
H	107	HOP2
H	107	HN32
H	107	HN2A
H	107	HN2B
H	107	HAL1
H	107	HAL2
H	107	HO1A
BR	94	BRAB
CL	93	CL08
CL	93	CLAB
CL	93	CLAQ
BR	94	BRAC
H	107	H733
H	107	H731
H	107	H732
H	107	H643
H	107	H642
H	107	H641
H	107	H512
H	107	H513
CL	93	CLAA
H	107	H21A
H	107	H21B
H	107	H29B
H	107	HO91
H	107	HO11
H	107	H263
H	107	HO51
H	107	HO61
H	107	H1AA
H	107	H1AB
H	107	H1AC
C	106	C101
C	106	C102
S	111	SS4
H	107	H273
H	107	H283
BR	94	BRAT

BR	94	BRAR
H	107	HA31
H	107	HA32
H	107	HA41
H	107	HA42
H	107	HB51
H	107	HB52
H	107	HB61
H	107	HB62
H	107	HAB3
H	107	H4N1
H	107	HN5'
H	107	H033
H	107	H12C
H	107	H11C
H	107	HABA
H	107	HABB
H	107	HACB
H	107	HNAR
H	107	HO10
H	107	HO13
H	107	HO14
H	107	HO15
H	107	H314
H	107	H214
H	107	H41O
H	107	H611
H	107	H612
H	107	H52O
H	107	H23O
H	107	H43O
H	107	H631
H	107	H632
H	107	H63O
D	107	DD21
D	107	DD22
D	107	DH11
D	107	DH12
D	107	DH21
D	107	DH22
D	107	DE21
D	107	DE22
H	107	H13B
H	107	HO5'

BR	94	BR15
BR	94	BRAW
CL	93	CL12
CL	93	CL07
CL	93	CL4
H	107	HN1A
P	110	P42
O	109	OBS
N	108	NCK
N	108	NCL
N	108	NCJ
O	109	OBY
S	111	S3'
CL	93	CLAI
O	109	O9'

(xi) sample_details_psi_ab_mix1.csv

protein	chain	group	group1	group1	group1	Organism
5OY0	A	PsaA	PsaA	PsaA	PsaA	Synechocystis_sp_PCC6803
5OY0	B	PsaB	PsaB	PsaB	PsaB	Synechocystis_sp_PCC6803
1JB0	A	PsaA	PsaA	PsaA	PsaA	Synechococcus_elongatus
1JB0	B	PsaB	PsaB	PsaB	PsaB	Synechococcus_elongatus
5ZJI	A	PsaA	PsaA	PsaA	PsaA	Zea_mays
5ZJI	B	PsaB	PsaB	PsaB	PsaB	Zea_mays
6HQB	A	PsaA	PsaA	PsaA	PsaA	Synechocystis_sp_PCC6803
6HQB	B	PsaB	PsaB	PsaB	PsaB	Synechocystis_sp_PCC6803
6JO6	A	PsaA	PsaA	PsaA	PsaA	Chlamydomonas_reinhardtii
6JO6	B	PsaB	PsaB	PsaB	PsaB	Chlamydomonas_reinhardtii
6KMW	A	PsaA	PsaA	PsaA	PsaA	Halomicronema_hongdechloris
6KMW	B	PsaB	PsaB	PsaB	PsaB	Halomicronema_hongdechloris
6KMX	A	PsaA	PsaA	PsaA	PsaA	Halomicronema_hongdechloris
6KMX	B	PsaB	PsaB	PsaB	PsaB	Halomicronema_hongdechloris
6PNJ	A	PsaA	PsaA	PsaA	PsaA	Fischerella_thermalis
6PNJ	B	PsaB	PsaB	PsaB	PsaB	Fischerella_thermalis
7COY	A	PsaA	PsaA	PsaA	PsaA	Acaryochloris_marina
7COY	B	PsaB	PsaB	PsaB	PsaB	Acaryochloris_marina

(xii) sample_details_et_mix5_name_id_atom.csv

prote	chain	drug_	drug_	grouı	grouı	ligand_atom
1JB0	A	CLA	1013	A0	A0A	MG;CHA;CHB;CHC;CHD;NA;C1A;C2A;C3A;C4A;CMA;CAA;CBA;CGA;O1A;O2A;NB;C1B;C2B;C3B;C4B;CMB;CAB;CBB;NC;C1C;C2C;C3C;C4C;CMC;CAC;CBC;ND;C1D;C2D;C3D;C4D;CMD;CAD;OBD;CBD;CGD;O1D;O2D;CED;C1;C2;C3;C4;C5
1JB0	B	CLA	1023	A0	A0B	MG;CHA;CHB;CHC;CHD;NA;C1A;C2A;C3A;C4A;CMA;CAA;CBA;CGA;O1A;O2A;NB;C1B;C2B;C3B;C4B;CMB;CAB;CBB;NC;C1C;C2C;C3C;C4C;CMC;CAC;CBC;ND;C1D;C2D;C3D;C4D;CMD;CAD;OBD;CBD;CGD;O1D;O2D;CED;C1;C2;C3;C4;C5
1JB0	B	CLA	1012	AA	AAA	MG;CHA;CHB;CHC;CHD;NA;C1A;C2A;C3A;C4A;CMA;CAA;CBA;CGA;O1A;O2A;NB;C1B;C2B;C3B;C4B;CMB;CAB;CBB;NC;C1C;C2C;C3C;C4C;CMC;CAC;CBC;ND;C1D;C2D;C3D;C4D;CMD;CAD;OBD;CBD;CGD;O1D;O2D;CED;C1;C2;C3;C4;C5
1JB0	A	CLA	1022	AA	AAB	MG;CHA;CHB;CHC;CHD;NA;C1A;C2A;C3A;C4A;CMA;CAA;CBA;CGA;O1A;O2A;NB;C1B;C2B;C3B;C4B;CMB;CAB;CBB;NC;C1C;C2C;C3C;C4C;CMC;CAC;CBC;ND;C1D;C2D;C3D;C4D;CMD;CAD;OBD;CBD;CGD;O1D;O2D;CED;C1;C2;C3;C4;C5
1JB0	A	CLA	1011	P700	P700	MG;CHA;CHB;CHC;CHD;NA;C1A;C2A;C3A;C4A;CMA;CAA;CBA;CGA;O1A;O2A;NB;C1B;C2B;C3B;C4B;CMB;CAB;CBB;NC;C1C;C2C;C3C;C4C;CMC;CAC;CBC;ND;C1D;C2D;C3D;C4D;CMD;CAD;OBD;CBD;CGD;O1D;O2D;CED;C1;C2;C3;C4;C5
1JB0	B	CLA	1021	P700	P700	MG;CHA;CHB;CHC;CHD;NA;C1A;C2A;C3A;C4A;CMA;CAA;CBA;CGA;O1A;O2A;NB;C1B;C2B;C3B;C4B;CMB;CAB;CBB;NC;C1C;C2C;C3C;C4C;CMC;CAC;CBC;ND;C1D;C2D;C3D;C4D;CMD;CAD;OBD;CBD;CGD;O1D;O2D;CED;C1;C2;C3;C4;C5
5OY(	A	CLA	1013	A0	A0A	MG;CHA;CHB;CHC;CHD;NA;C1A;C2A;C3A;C4A;CMA;CAA;CBA;CGA;O1A;O2A;NB;C1B;C2B;C3B;C4B;CMB;CAB;CBB;NC;C1C;C2C;C3C;C4C;CMC;CAC;CBC;ND;C1D;C2D;C3D;C4D;CMD;CAD;OBD;CBD;CGD;O1D;O2D;CED;C1;C2;C3;C4;C5
5OY(	B	CLA	1023	A0	A0B	MG;CHA;CHB;CHC;CHD;NA;C1A;C2A;C3A;C4A;CMA;CAA;CBA;CGA;O1A;O2A;NB;C1B;C2B;C3B;C4B;CMB;CAB;CBB;NC;C1C;C2C;C3C;C4C;CMC;CAC;CBC;ND;C1D;C2D;C3D;C4D;CMD;CAD;OBD;CBD;CGD;O1D;O2D;CED;C1;C2;C3;C4;C5
5OY(	A	CLA	1012	AA	AAA	MG;CHA;CHB;CHC;CHD;NA;C1A;C2A;C3A;C4A;CMA;CAA;CBA;CGA;O1A;O2A;NB;C1B;C2B;C3B;C4B;CMB;CAB;CBB;NC;C1C;C2C;C3C;C4C;CMC;CAC;CBC;ND;C1D;C2D;C3D;C4D;CMD;CAD;OBD;CBD;CGD;O1D;O2D;CED;C1;C2;C3;C4;C5
5OY(	B	CLA	1022	AA	AAB	MG;CHA;CHB;CHC;CHD;NA;C1A;C2A;C3A;C4A;CMA;CAA;CBA;CGA;O1A;O2A;NB;C1B;C2B;C3B;C4B;CMB;CAB;CBB;NC;C1C;C2C;C3C;C4C;CMC;CAC;CBC;ND;C1D;C2D;C3D;C4D;CMD;CAD;OBD;CBD;CGD;O1D;O2D;CED;C1;C2;C3;C4;C5
5OY(	A	CLA	1011	P700	P700	MG;CHA;CHB;CHC;CHD;NA;C1A;C2A;C3A;C4A;CMA;CAA;CBA;CGA;O1A;O2A;NB;C1B;C2B;C3B;C4B;CMB;CAB;CBB;NC;C1C;C2C;C3C;C4C;CMC;CAC;CBC;ND;C1D;C2D;C3D;C4D;CMD;CAD;OBD;CBD;CGD;O1D;O2D;CED;C1;C2;C3;C4;C5
5OY(	B	CLA	1021	P700	P700	MG;CHA;CHB;CHC;CHD;NA;C1A;C2A;C3A;C4A;CMA;CAA;CBA;CGA;O1A;O2A;NB;C1B;C2B;C3B;C4B;CMB;CAB;CBB;NC;C1C;C2C;C3C;C4C;CMC;CAC;CBC;ND;C1D;C2D;C3D;C4D;CMD;CAD;OBD;CBD;CGD;O1D;O2D;CED;C1;C2;C3;C4;C5
5ZJI	A	CLA	802	A0	A0A	MG;CHA;CHB;CHC;CHD;NA;C1A;C2A;C3A;C4A;CMA;CAA;CBA;CGA;O1A;O2A;NB;C1B;C2B;C3B;C4B;CMB;CAB;CBB;NC;C1C;C2C;C3C;C4C;CMC;CAC;CBC;ND;C1D;C2D;C3D;C4D;CMD;CAD;OBD;CBD;CGD;O1D;O2D;CED;C1;C2;C3;C4;C5
5ZJI	B	CLA	803	A0	A0B	MG;CHA;CHB;CHC;CHD;NA;C1A;C2A;C3A;C4A;CMA;CAA;CBA;CGA;O1A;O2A;NB;C1B;C2B;C3B;C4B;CMB;CAB;CBB;NC;C1C;C2C;C3C;C4C;CMC;CAC;CBC;ND;C1D;C2D;C3D;C4D;CMD;CAD;OBD;CBD;CGD;O1D;O2D;CED;C1;C2;C3;C4;C5
5ZJI	A	CLA	803	AA	AAA	MG;CHA;CHB;CHC;CHD;NA;C1A;C2A;C3A;C4A;CMA;CAA;CBA;CGA;O1A;O2A;NB;C1B;C2B;C3B;C4B;CMB;CAB;CBB;NC;C1C;C2C;C3C;C4C;CMC;CAC;CBC;ND;C1D;C2D;C3D;C4D;CMD;CAD;OBD;CBD;CGD;O1D;O2D;CED;C1;C2;C3;C4;C5
5ZJI	A	CLA	854	AA	AAB	MG;CHA;CHB;CHC;CHD;NA;C1A;C2A;C3A;C4A;CMA;CAA;CBA;CGA;O1A;O2A;NB;C1B;C2B;C3B;C4B;CMB;CAB;CBB;NC;C1C;C2C;C3C;C4C;CMC;CAC;CBC;ND;C1D;C2D;C3D;C4D;CMD;CAD;OBD;CBD;CGD;O1D;O2D;CED;C1;C2;C3;C4;C5
5ZJI	A	CL0	801	P700	P700	MG;CHA;CHB;CHC;CHD;NA;C1A;C2A;C3A;C4A;CMA;CAA;CBA;CGA;O1A;O2A;NB;C1B;C2B;C3B;C4B;CMB;CAB;CBB;NC;C1C;C2C;C3C;C4C;CMC;CAC;CBC;ND;C1D;C2D;C3D;C4D;CMD;CAD;OBD;CBD;CGD;O1D;O2D;CED;C1;C2;C3;C4;C5
5ZJI	B	CLA	802	P700	P700	MG;CHA;CHB;CHC;CHD;NA;C1A;C2A;C3A;C4A;CMA;CAA;CBA;CGA;O1A;O2A;NB;C1B;C2B;C3B;C4B;CMB;CAB;CBB;NC;C1C;C2C;C3C;C4C;CMC;CAC;CBC;ND;C1D;C2D;C3D;C4D;CMD;CAD;OBD;CBD;CGD;O1D;O2D;CED;C1;C2;C3;C4;C5
6JO6	A	CLA	802	A0	A0A	MG;CHA;CHB;CHC;CHD;NA;C1A;C2A;C3A;C4A;CMA;CAA;CBA;CGA;O1A;O2A;NB;C1B;C2B;C3B;C4B;CMB;CAB;CBB;NC;C1C;C2C;C3C;C4C;CMC;CAC;CBC;ND;C1D;C2D;C3D;C4D;CMD;CAD;OBD;CBD;CGD;O1D;O2D;CED;C1;C2;C3;C4;C5
6JO6	B	CLA	803	A0	A0B	MG;CHA;CHB;CHC;CHD;NA;C1A;C2A;C3A;C4A;CMA;CAA;CBA;CGA;O1A;O2A;NB;C1B;C2B;C3B;C4B;CMB;CAB;CBB;NC;C1C;C2C;C3C;C4C;CMC;CAC;CBC;ND;C1D;C2D;C3D;C4D;CMD;CAD;OBD;CBD;CGD;O1D;O2D;CED;C1;C2;C3;C4;C5
6JO6	A	CLA	803	AA	AAA	MG;CHA;CHB;CHC;CHD;NA;C1A;C2A;C3A;C4A;CMA;CAA;CBA;CGA;O1A;O2A;NB;C1B;C2B;C3B;C4B;CMB;CAB;CBB;NC;C1C;C2C;C3C;C4C;CMC;CAC;CBC;ND;C1D;C2D;C3D;C4D;CMD;CAD;OBD;CBD;CGD;O1D;O2D;CED;C1;C2;C3;C4;C5
6JO6	A	CLA	854	AA	AAB	MG;CHA;CHB;CHC;CHD;NA;C1A;C2A;C3A;C4A;CMA;CAA;CBA;CGA;O1A;O2A;NB;C1B;C2B;C3B;C4B;CMB;CAB;CBB;NC;C1C;C2C;C3C;C4C;CMC;CAC;CBC;ND;C1D;C2D;C3D;C4D;CMD;CAD;OBD;CBD;CGD;O1D;O2D;CED;C1;C2;C3;C4;C5
6JO6	A	CL0	801	P700	P700	MG;CHA;CHB;CHC;CHD;NA;C1A;C2A;C3A;C4A;CMA;CAA;CBA;CGA;O1A;O2A;NB;C1B;C2B;C3B;C4B;CMB;CAB;CBB;NC;C1C;C2C;C3C;C4C;CMC;CAC;CBC;ND;C1D;C2D;C3D;C4D;CMD;CAD;OBD;CBD;CGD;O1D;O2D;CED;C1;C2;C3;C4;C5
6JO6	B	CLA	802	P700	P700	MG;CHA;CHB;CHC;CHD;NA;C1A;C2A;C3A;C4A;CMA;CAA;CBA;CGA;O1A;O2A;NB;C1B;C2B;C3B;C4B;CMB;CAB;CBB;NC;C1C;C2C;C3C;C4C;CMC;CAC;CBC;ND;C1D;C2D;C3D;C4D;CMD;CAD;OBD;CBD;CGD;O1D;O2D;CED;C1;C2;C3;C4;C5
6PNJ	A	CLA	1013	A0	A0A	MG;CHA;CHB;CHC;CHD;NA;C1A;C2A;C3A;C4A;CMA;CAA;CBA;CGA;O1A;O2A;NB;C1B;C2B;C3B;C4B;CMB;CAB;CBB;NC;C1C;C2C;C3C;C4C;CMC;CAC;CBC;ND;C1D;C2D;C3D;C4D;CMD;CAD;OBD;CBD;CGD;O1D;O2D;CED;C1;C2;C3;C4;C5
6PNJ	B	CLA	1023	A0	A0B	MG;CHA;CHB;CHC;CHD;NA;C1A;C2A;C3A;C4A;CMA;CAA;CBA;CGA;O1A;O2A;NB;C1B;C2B;C3B;C4B;CMB;CAB;CBB;NC;C1C;C2C;C3C;C4C;CMC;CAC;CBC;ND;C1D;C2D;C3D;C4D;CMD;CAD;OBD;CBD;CGD;O1D;O2D;CED;C1;C2;C3;C4;C5
6PNJ	A	CLA	1012	AA	AAA	MG;CHA;CHB;CHC;CHD;NA;C1A;C2A;C3A;C4A;CMA;CAA;CBA;CGA;O1A;O2A;NB;C1B;C2B;C3B;C4B;CMB;CAB;CBB;NC;C1C;C2C;C3C;C4C;CMC;CAC;CBC;ND;C1D;C2D;C3D;C4D;CMD;CAD;OBD;CBD;CGD;O1D;O2D;CED;C1;C2;C3;C4;C5
6PNJ	B	CLA	1022	AA	AAB	MG;CHA;CHB;CHC;CHD;NA;C1A;C2A;C3A;C4A;CMA;CAA;CBA;CGA;O1A;O2A;NB;C1B;C2B;C3B;C4B;CMB;CAB;CBB;NC;C1C;C2C;C3C;C4C;CMC;CAC;CBC;ND;C1D;C2D;C3D;C4D;CMD;CAD;OBD;CBD;CGD;O1D;O2D;CED;C1;C2;C3;C4;C5
6PNJ	A	CL0	1011	P700	P700	MG;CHA;CHB;CHC;CHD;NA;C1A;C2A;C3A;C4A;CMA;CAA;CBA;CGA;O1A;O2A;NB;C1B;C2B;C3B;C4B;CMB;CAB;CBB;NC;C1C;C2C;C3C;C4C;CMC;CAC;CBC;ND;C1D;C2D;C3D;C4D;CMD;CAD;OBD;CBD;CGD;O1D;O2D;CED;C1;C2;C3;C4;C5
6PNJ	B	CLA	1021	P700	P700	MG;CHA;CHB;CHC;CHD;NA;C1A;C2A;C3A;C4A;CMA;CAA;CBA;CGA;O1A;O2A;NB;C1B;C2B;C3B;C4B;CMB;CAB;CBB;NC;C1C;C2C;C3C;C4C;CMC;CAC;CBC;ND;C1D;C2D;C3D;C4D;CMD;CAD;OBD;CBD;CGD;O1D;O2D;CED;C1;C2;C3;C4;C5
